# Supplementary figures and images for: Anthropogenic reverberations on the gut microbiome of dwarf chameleons (Bradypodion)
Source: PeerJ. 2025 Feb 28;13:e18811. doi: 10.7717/peerj.18811 (PMC11874949; doi:10.7717/peerj.18811)

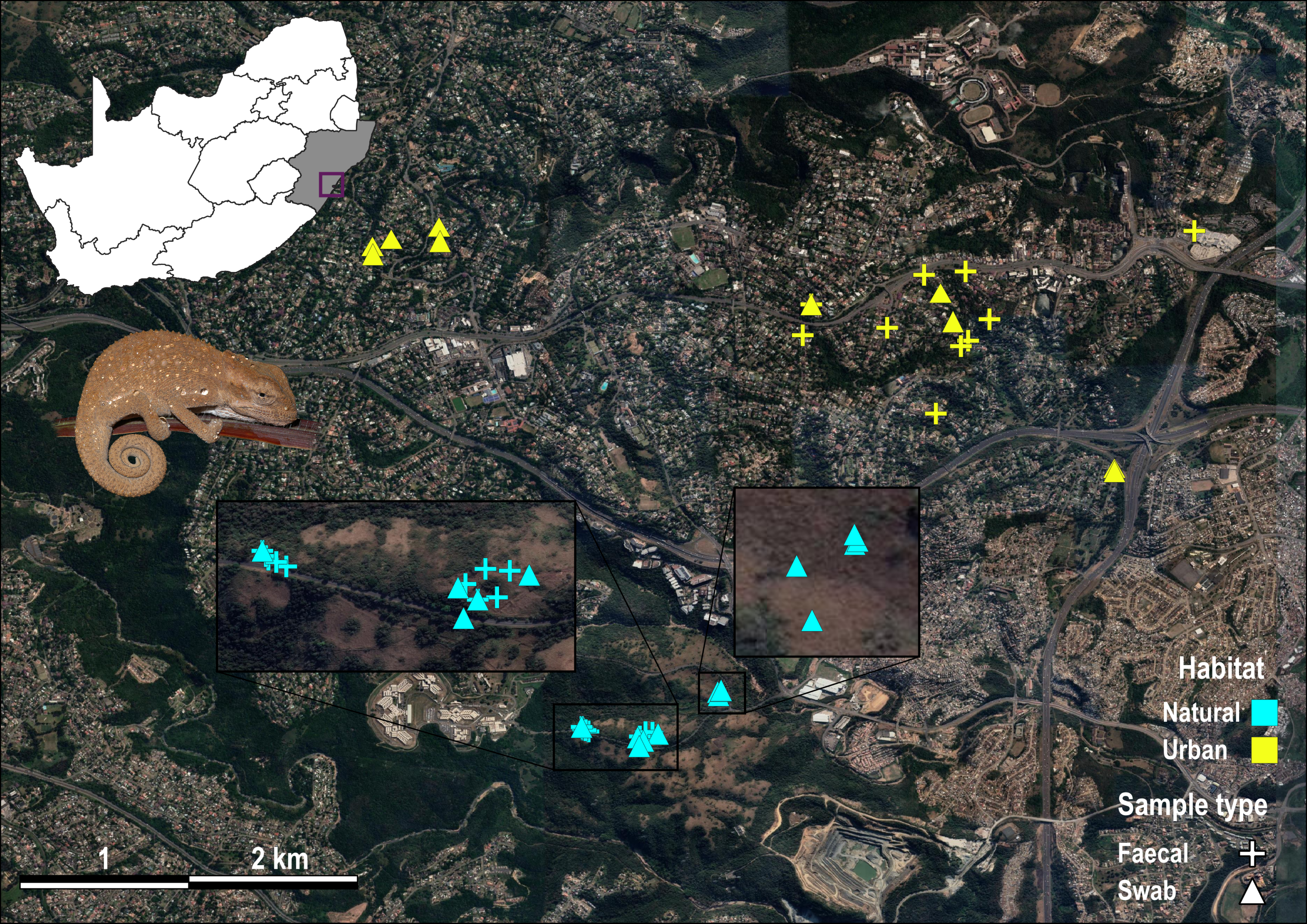

Supplement: Supplemental Information 1 — Localities are coloured yellow from urban habitat and light blue from natural habitat. Faecal samples are indicated by crosses and buccal swab samples are indicated by triangles. [file peerj-13-18811-s001.png]

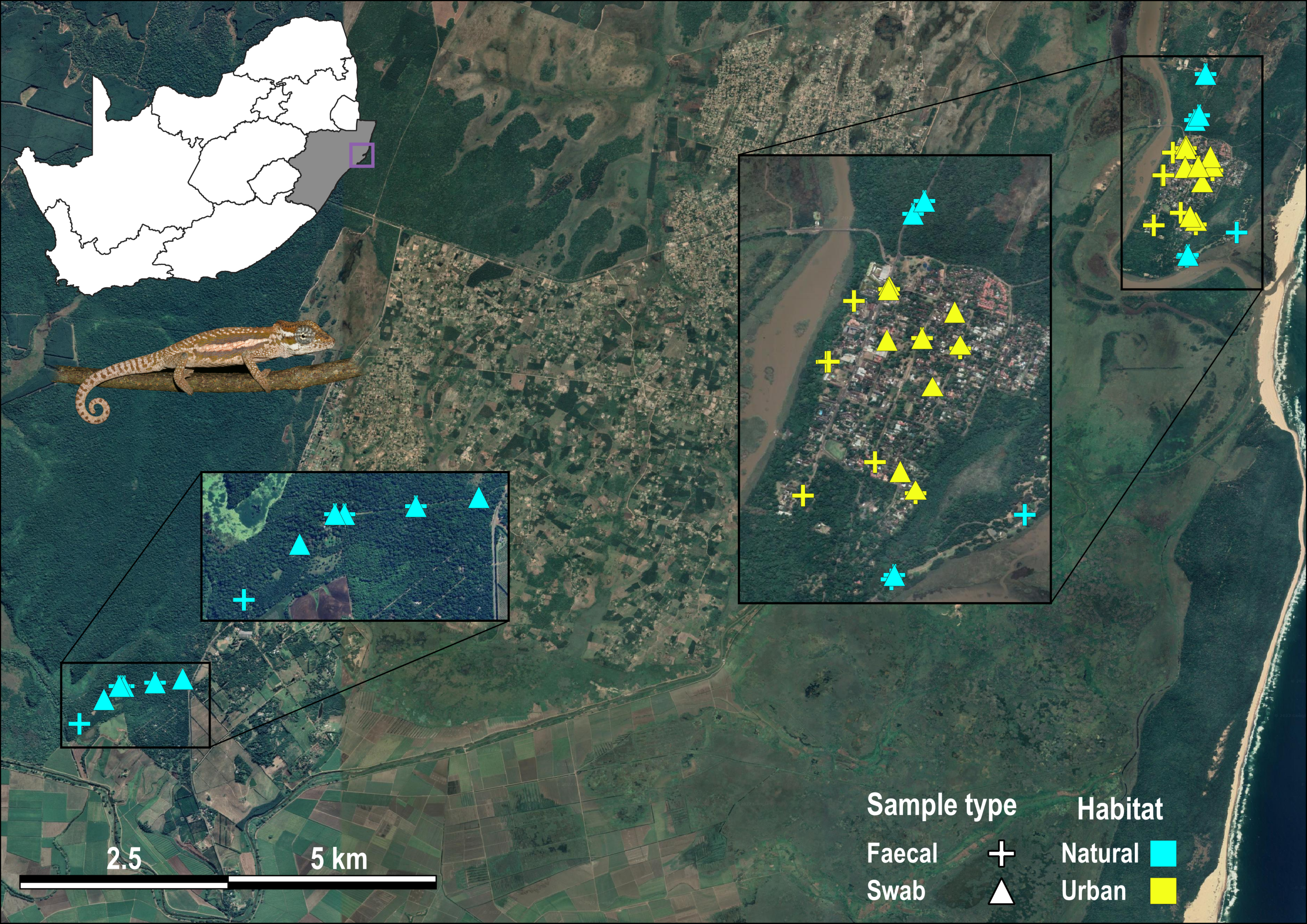

Supplement: Supplemental Information 2 — Localities are coloured yellow from urban habitat and light blue from natural habitat. Faecal samples are indicated by crosses and buccal swab samples are indicated by triangles. [file peerj-13-18811-s002.png]

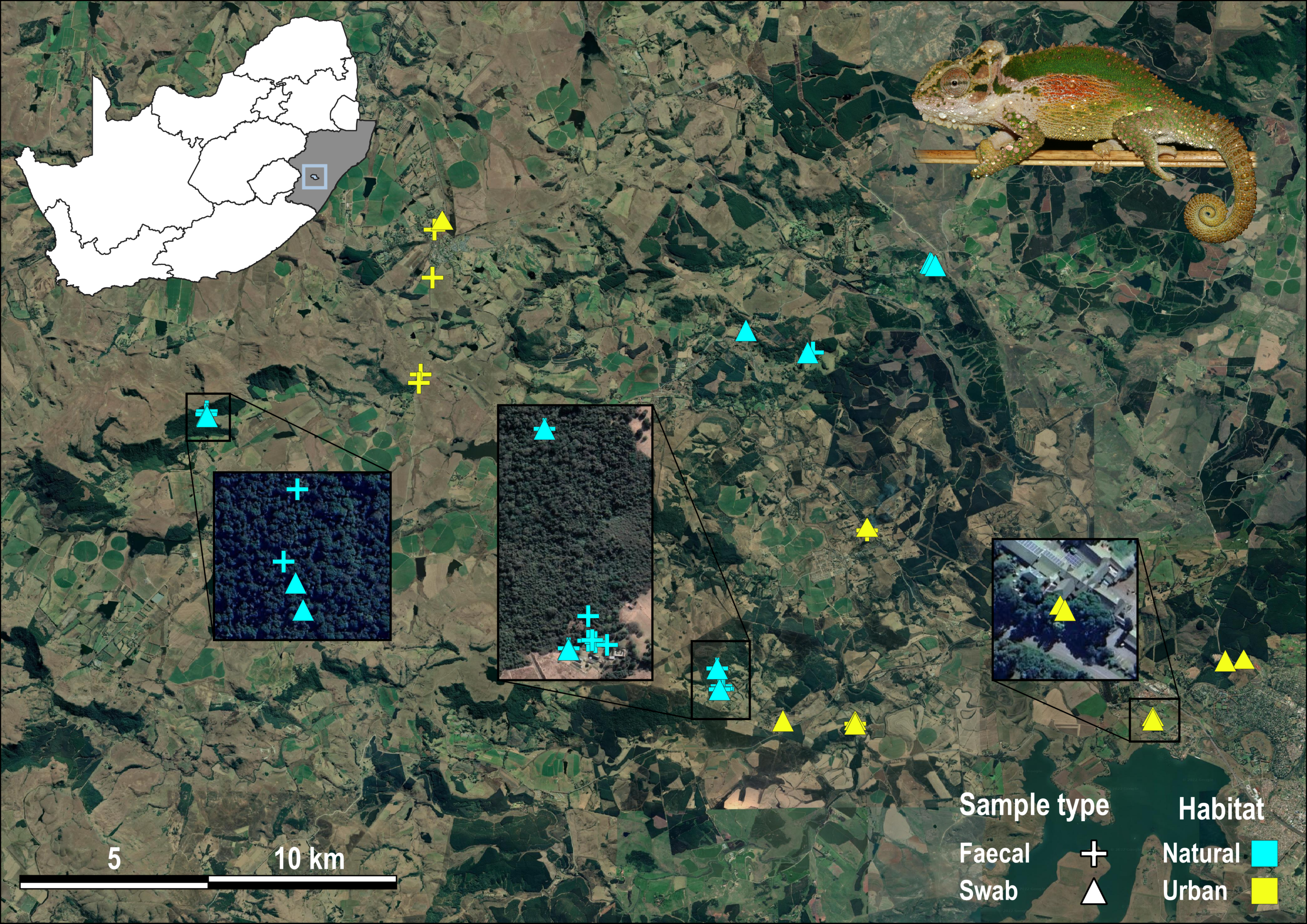

Supplement: Supplemental Information 3 — Localities are coloured yellow from urban habitat and light blue from natural habitat. Faecal samples are indicated by crosses and buccal swab samples are indicated by triangles. [file peerj-13-18811-s003.png]

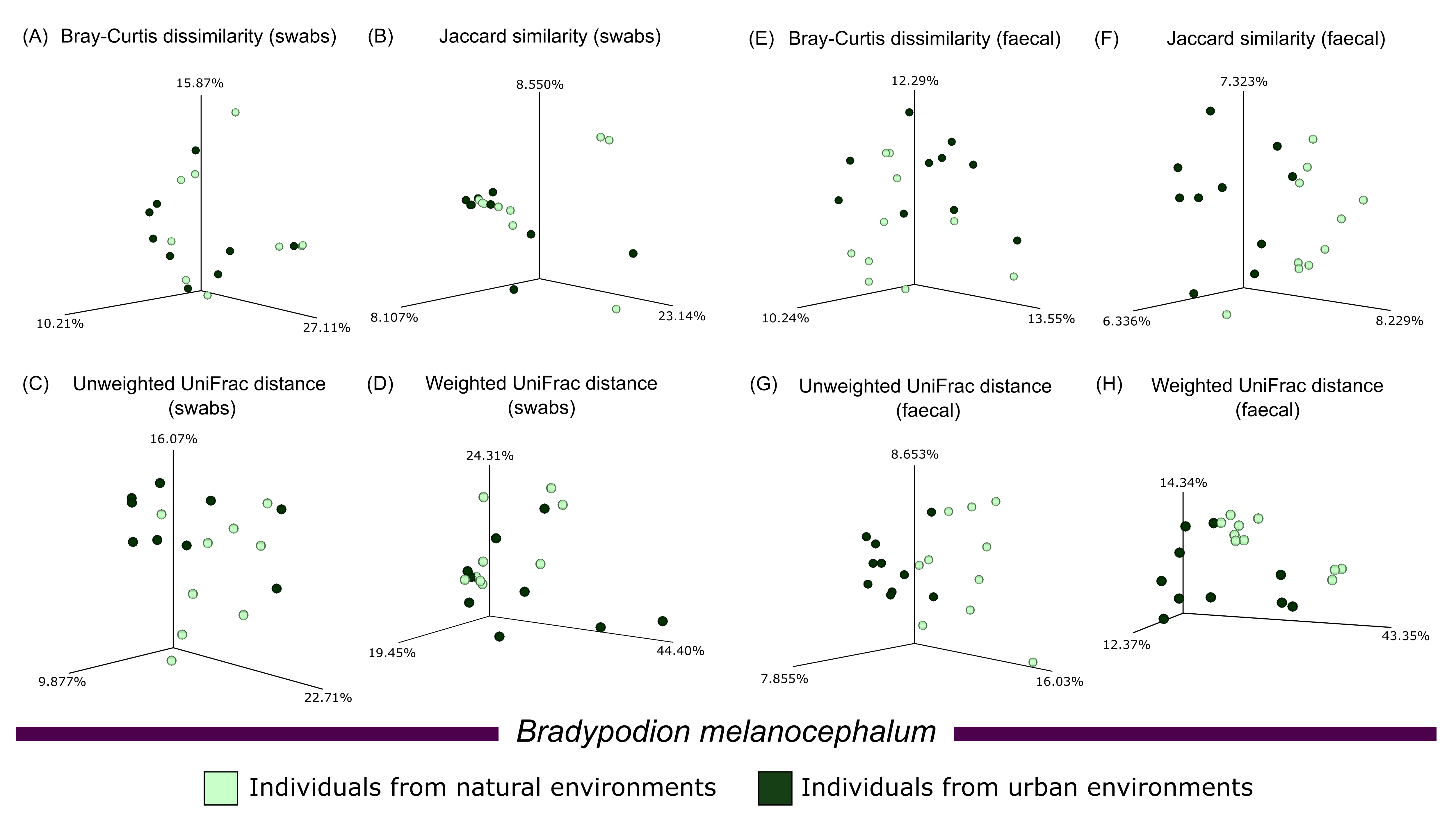

Supplement: Supplemental Information 4 — Principal Coordinate Analysis (PCoA) for calculated beta diversity metrics (Bray-Curtis dissimilarity index, Jaccard similarity index, Unweighted UniFrac distance, and Weighted UniFrac distance) across buccal swab samples (left) and faecal material samples (right) characterised by natural and urban populations from Bradypodion melanocephalum. [file peerj-13-18811-s004.png]

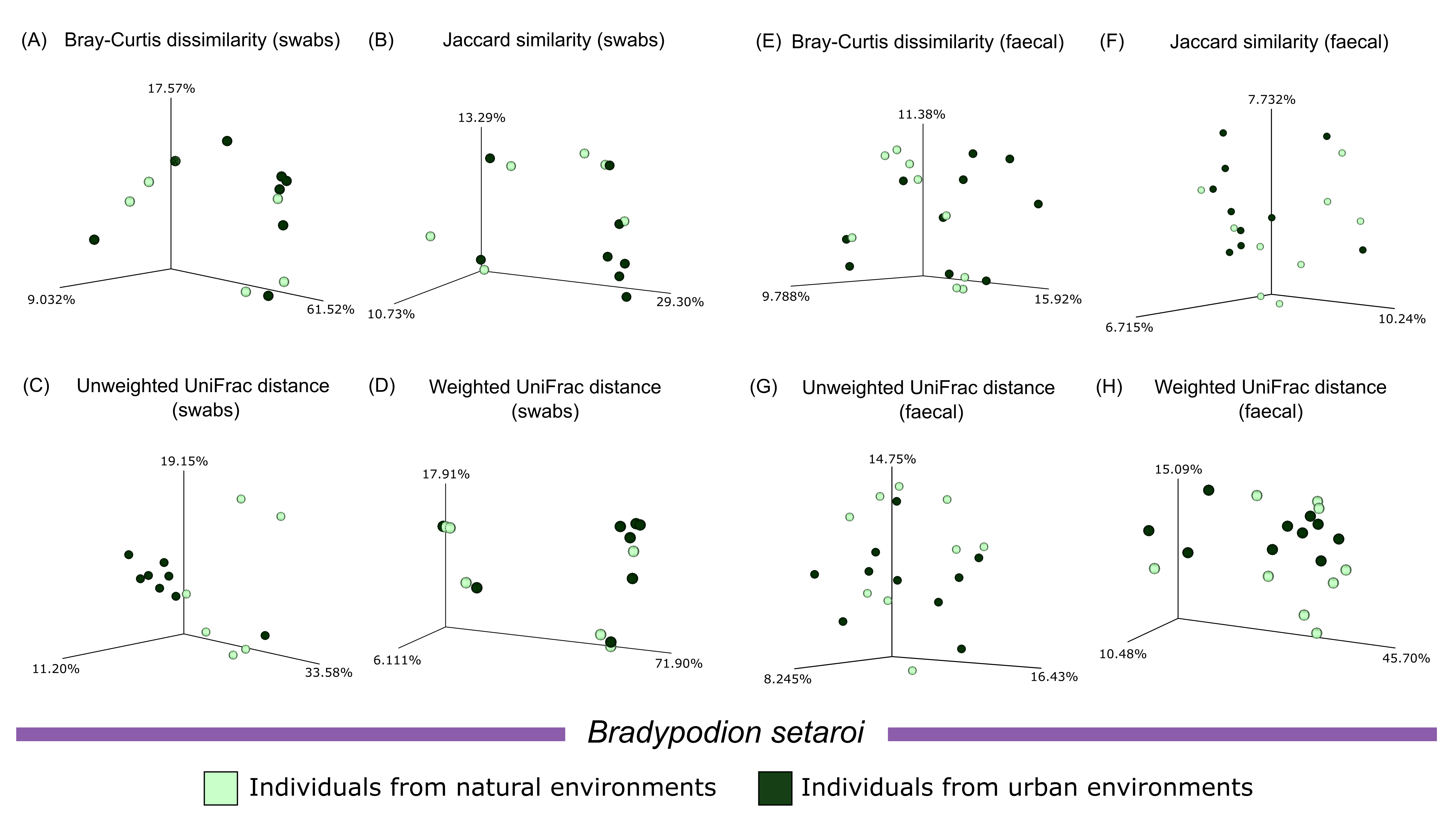

Supplement: Supplemental Information 5 — Principal Coordinate Analysis (PCoA) for calculated beta diversity metrics (Bray-Curtis dissimilarity index, Jaccard similarity index, Unweighted UniFrac distance, and Weighted UniFrac distance) across buccal swab samples (left) and faecal material samples (right) characterised by natural and urban populations from Bradypodion setaroi. [file peerj-13-18811-s005.png]

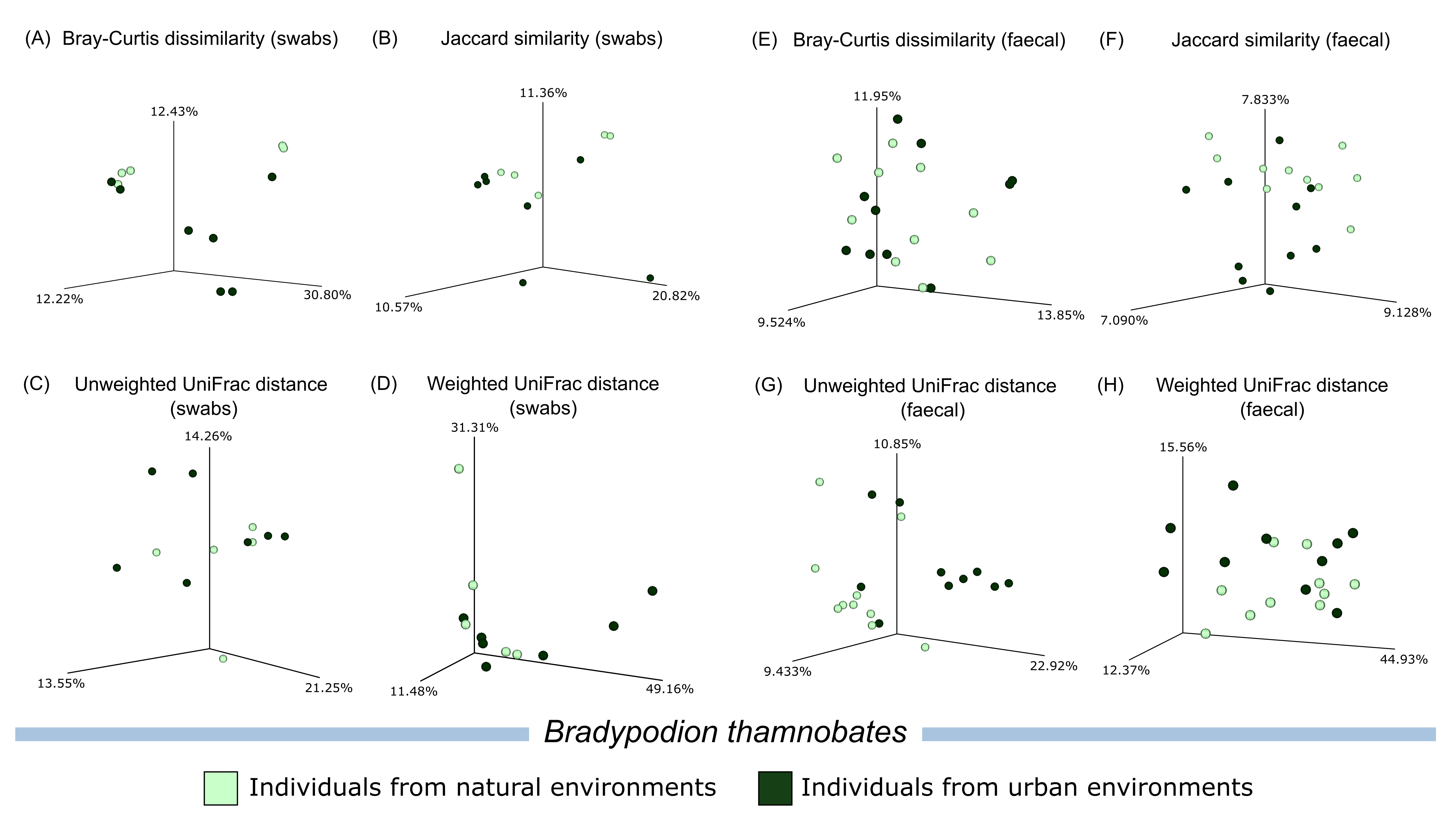

Supplement: Supplemental Information 6 — Principal Coordinate Analysis (PCoA) for calculated beta diversity metrics (Bray-Curtis dissimilarity index, Jaccard similarity index, Unweighted UniFrac distance, and Weighted UniFrac distance) across buccal swab samples (left) and faecal material samples (right) characterised by natural and urban populations from Bradypodion thamnobates. [file peerj-13-18811-s006.png]
