# Supplementary material for: Anthropogenic reverberations on the gut microbiome of dwarf chameleons (Bradypodion)
Source: PeerJ. 2025 Feb 28;13:e18811. doi: 10.7717/peerj.18811 (PMC11874949; doi:10.7717/peerj.18811)
Supplement: Supplemental Information 7 — Shown are taxonomic rank, taxonomic nomenclature of identification, the confidence of identification, and the unique sequence ASV identifier assigned. Any uncultured or unidentified classifications were assigned to the next highest taxonomic rank that was certain. [file peerj-13-18811-s007.docx]

| **Taxonomic rank** | **Taxon nomenclature** | **Confidence** | **Assigned sequence ID** |
| --- | --- | --- | --- |
| Phylum | Firmicutes | 99.91% | 253da3f2003c66ffda7b498233410fff |
| Phylum | Firmicutes | 96.10% | 7fbe53c790cbcd631feb2385259f3d7c |
| Phylum | Firmicutes | 70.32% | 7a33a753e13714f569153a2aea6ba0da |
| Phylum | Firmicutes | 71.46% | f3e123f539d5e059ea469966e93ee1d2 |
| Phylum | Firmicutes | 85.43% | 1ea10acc964b92acd02d2cc710c7f1a9 |
| Phylum | Planctomycetota | 99.75% | 11dbebac87effb545c46f12fc9bf1bdf |
| Class | Actinobacteria | 100.00% | efa9ce315ac589dc6ade7b9ebac4369c |
| Class | Actinobacteria | 100.00% | 6b17b05ca45be96f2b55bdf906a5add9 |
| Class | Actinobacteria | 100.00% | 65e535c36d6521a4ea0fe901286b7576 |
| Class | Actinobacteria | 100.00% | 9eda2a27ded06ad4b4d06f2f711f0994 |
| Class | Actinobacteria | 100.00% | a263c18d4a4de4e0cf135867d44f2690 |
| Class | Alphaproteobacteria | 100.00% | 4a0568cf762a6322dd5a616966a1a087 |
| Class | Alphaproteobacteria | 72.15% | 71dc96303b95e7accb97a47b67bc5121 |
| Class | Bacilli | 73.30% | 29c6eed065ea39b8cdbadabc9c313ce7 |
| Class | Bacilli | 89.69% | fc7b56c6486b86a0bdcd06d9411a2104 |
| Class | Bacilli | 99.95% | 3530bdff4eb5e9a58f504d0aabc09a9c |
| Class | Bacilli | 99.51% | 9cc4f199a38f66b5f99ea9bcf7e826df |
| Class | Bacteroidia | 76.46% | 3a22f07584cb527c88dad2699b3ccd7f |
| Class | Bacteroidia | 93.05% | 501e399249cdc78810dd1295a01d8283 |
| Class | Bacteroidia | 99.91% | b0acedf596553995dd3f29698a1b43f6 |
| Class | Bacteroidia | 95.99% | 396fa5cfca955a6fe00c4e159b3a28ff |
| Class | Clostridia | 71.06% | e54925a3e2a45f33f4786ea409e6f944 |
| Class | Clostridia | 99.98% | 960bee4448d441982de56dd40dc27e61 |
| Class | Clostridia | 99.86% | aa9f612cc2f06078f6546645e0c75d35 |
| Order | Bacillales | 99.99% | 146c009a9b43159681cab218664cd33a |
| Order | Bacteroidales | 90.47% | f10f746b408c288c444287c82f1065fd |
| Order | Bacteroidales | 99.96% | 1b5e41869c65fa5b95e774b1181b24a5 |
| Order | Bacteroidales | 100.00% | 484f5d212e192cba5a911d7d58049c91 |
| Order | Bacteroidales | 98.10% | 5903d79f355344d6791225ea8db53d51 |
| Order | Bacteroidales | 99.99% | 909d61d2bb4f182fce9de92daa6f7f6e |
| Order | Bacteroidales | 88.70% | 99d77514fa3ad18fc3d0f8b80c6c8381 |
| Order | Bacteroidales | 100.00% | 647cd5675b6d460a1cb5f9bdd95c4ddb |
| Order | Bacteroidales | 93.44% | d3f8b1fd896eeebbe33eaeed7652c026 |
| Order | Burkholderiales | 91.66% | 0d7dd08feac8112c2f4b62848259d7ba |
| Order | Burkholderiales | 99.91% | 4b9907d0d7360ba0e165a57f821efb5a |
| Order | Burkholderiales | 82.37% | 18e647f8b0de1de1ec4c72c4e246e7fd |
| Order | Burkholderiales | 95.96% | 28772d0fbad7418208c159c8d079c4b2 |
| Order | Burkholderiales | 95.33% | e88798c66675796257cf451be8e31f37 |
| Order | Burkholderiales | 80.80% | 606b9e5879fd864e9239ab608b357a99 |
| Order | Corynebacteriales | 100.00% | 7e699578d9780020f65ecda24cf0fdd2 |
| Order | Corynebacteriales | 100.00% | 383bc92c177f0122ab732ed319c22a8f |
| Order | Corynebacteriales | 100.00% | 71836fdd193fe28dead40a8a142c14f9 |
| Order | Cyanobacteriales | 87.67% | 93f1a18a772918bd3d213d1512a35c79 |
| Order | Enterobacterales | 77.00% | 4d4d335a27350cfd004b50251de14285 |
| Order | Oscillospirales | 99.98% | 9cc4c4f184fd4c19e45477eb274ee1ad |
| Order | Oscillospirales | 99.98% | 198f5ede0084932df126c791f08c110d |
| Order | Oscillospirales | 99.99% | 8f2e1acf33d07bc3c1102a212d1ed84f |
| Order | Peptostreptococcales-Tissierellales | 99.42% | ede15ea5a91f1735ced6743546a12bcb |
| Order | Peptostreptococcales-Tissierellales | 99.38% | 2a47ead13657081c95cdf81226400cca |
| Order | Peptostreptococcales-Tissierellales | 99.53% | 1d5caaf93d7110be8beecc91174ccce2 |
| Order | Peptostreptococcales-Tissierellales | 90.25% | 815e19694c88b2d2579bdb3af3fe90f4 |
| Order | Peptostreptococcales-Tissierellales | 92.27% | 88708b317fdc27c32ef95bdac29d67ce |
| Order | Pseudomonadales | 99.99% | 706e47aa32d531e34ef4f935a1063ad4 |
| Order | Pseudomonadales | 99.99% | 5763850e98314c5982ed1035b6336026 |
| Order | Pseudomonadales | 99.94% | da4570babff7068127046567d6369a15 |
| Order | Pseudomonadales | 99.99% | e7eada8a3cabceca4e74a9f423806877 |
| Order | Rhizobiales | 100.00% | 7790628b89f1ecd074db2fe82ddee2c5 |
| Order | Rhizobiales | 100.00% | 87d392c2fde21132952563813a96c460 |
| Order | Saccharimonadales | 79.26% | a28932b4d963b654ebe06e3152ad935c |
| Order | Veillonellales-Selenomonadales | 99.99% | cc203b329f1d32b65ce6fcd0595efa6c |
| Family | Acetobacteraceae | 99.99% | 7a132550afcc1cde92f6efb622f0ae94 |
| Family | Acetobacteraceae | 99.99% | c667c37d92b5535a5ed8c83fbe7bff84 |
| Family | Acetobacteraceae | 100.00% | 18fb27931a7b9ce56bd00f72882093a4 |
| Family | Anaerovoracaceae | 99.13% | 222c97f2c4304944dff5c5f23090e19c |
| Family | Anaerovoracaceae | 87.65% | e7b5c9127d038fb62014fec7f7f0ad67 |
| Family | Bacillaceae | 92.84% | 64bf96af3d9ce29f6ff964c61b44aaeb |
| Family | Bacillaceae | 80.09% | 3d4feeaa282af9c22113f495ce4fda1c |
| Family | Beijerinckiaceae | 84.34% | 8a9b3caa4db1cf39b21510aa1c7c52f8 |
| Family | Beijerinckiaceae | 98.06% | 3150f5f6c7cf0b3b2200bf138a85d7e4 |
| Family | Beijerinckiaceae | 89.19% | bfc18a8bdbfd91db176188aa53fcdc75 |
| Family | Beijerinckiaceae | 74.17% | ae478c9810e0e3e77b697bf3864dd44f |
| Family | Chitinophagaceae | 100.00% | 690df2a02aeb7ea9eaaef59a86daca50 |
| Family | Chitinophagaceae | 98.99% | c40e14e3704d29d1340405272d5d9300 |
| Family | Clostridiaceae | 90.23% | e70c0a260c2701906f60e18716f56548 |
| Family | Comamonadaceae | 99.80% | 257a1243bdd05af46c7ee13c115c6434 |
| Family | Comamonadaceae | 98.60% | 32df36ad88aad6935a9a9755642ccf76 |
| Family | Dysgonomonadaceae | 77.20% | 1801eeb5e5b51f925728d2e8c4fa7879 |
| Family | Dysgonomonadaceae | 75.39% | 49cde44a92721d7b01d40f3e096b4b1c |
| Family | Dysgonomonadaceae | 73.50% | 32c3ff2e3375315c4be9eec811880a23 |
| Family | Lachnospiraceae | 100.00% | 6d8843cc13b2c7df43ef15fead3e993a |
| Family | Lachnospiraceae | 99.99% | 00fedfdbdd1deb6f2d1a0875e588f4d4 |
| Family | Lachnospiraceae | 100.00% | 373ab3522292a3f4d24fbffd6c574be9 |
| Family | Lachnospiraceae | 98.09% | a2338591cbc926fee73cdcba125e1ca7 |
| Family | Lachnospiraceae | 100.00% | f34c4987b03016ed8bd9a8722777b3c6 |
| Family | Lachnospiraceae | 99.97% | f3b748b5b184b6b5ee63916374c7843a |
| Family | Methyloligellaceae | 90.73% | 5aeeedcc346d51a5864777810c3366de |
| Family | Microbacteriaceae | 97.16% | e2dd8a22765600f618d6a225c02ca550 |
| Family | Microbacteriaceae | 93.37% | fe0afa86ff31aef0112b6b15981c3a9d |
| Family | Microbacteriaceae | 85.43% | 352c07a8447f9719a7c4a458f4f67bf2 |
| Family | Microbacteriaceae | 95.42% | 9d91a387896e7d64733a6a730d09d73d |
| Family | Microbacteriaceae | 87.66% | fee488f23b5a9456394b8035f511b796 |
| Family | Microbacteriaceae | 97.54% | b1f85723da8ba81075e07597d57cc8fa |
| Family | Microbacteriaceae | 76.43% | 69762327dde8d91ed7521f52baf63ea3 |
| Family | Microbacteriaceae | 74.25% | 0268e70b929405a02e50d026609a8373 |
| Family | Microbacteriaceae | 96.37% | 06274452709107af1e637eff724e5db1 |
| Family | Microbacteriaceae | 96.08% | 97cd98e1fd4fd91d33cfe82f4e8317dc |
| Family | Micrococcaceae | 97.00% | f844f7557efbabb489aa51ae6658ca4d |
| Family | Neisseriaceae | 99.47% | 3b942627249a38b6cd63640cd9b93ef5 |
| Family | Neisseriaceae | 99.23% | a0edab59f2a8c9bf65805794f5483cd6 |
| Family | Neisseriaceae | 99.69% | 91b24afbcc8c6f2131124b3c1f5afa75 |
| Family | Orbaceae | 99.71% | f5b6d0c1d9b774c9e724f3a2838bad76 |
| Family | Orbaceae | 99.71% | 7e76798fd0739209c546df4bb3d9d16a |
| Family | Orbaceae | 73.43% | b74043a198c51bf850e71ed4ff5d9782 |
| Family | Oscillospiraceae | 77.57% | 7865765e93e7ad9ef770967e37c773ca |
| Family | Pasteurellaceae | 99.98% | b39b26b8f2c9e77d7fface3175a97a94 |
| Family | Peptostreptococcaceae | 100.00% | 0648a9113ab225823e27ba206b3dfe32 |
| Family | Rhizobiaceae | 100.00% | 2ddc2ecff882ad2074de7bffe8071c69 |
| Family | Rhodocyclaceae | 76.25% | 14f3fcb2ce38e4cdda8df1078ae02db6 |
| Family | Rhodocyclaceae | 80.78% | 54bb585ec883aeff630f449f66c0f573 |
| Family | Ruminococcaceae | 93.39% | e3bff8d84c49163ed4759982a5dbe56e |
| Family | Ruminococcaceae | 99.56% | 0c286d525e1c4620dad1b09eb6eef64c |
| Family | Ruminococcaceae | 81.95% | 55b2e23d7d141891085e10297ddf573f |
| Family | Ruminococcaceae | 99.58% | 578ef62a530d2d281693c563330a8923 |
| Family | Ruminococcaceae | 97.88% | c9af8d1b236646c43d4f08a10f0a7660 |
| Family | Saccharimonadaceae | 81.91% | 9fce97e6139db1f0c0932e01a5d972ca |
| Family | Sandaracinaceae | 74.86% | f292658fb7e2524cca771808c3062abc |
| Family | Sphingomonadaceae | 82.60% | ca1e70bcf21190ca7924886e9b0ddcc0 |
| Family | Sutterellaceae | 72.04% | d0ebe3a949ee33a863de4cd928fc7909 |
| Family | Sutterellaceae | 74.23% | 44bb9ec517942ed8c82ce24e3890f111 |
| Family | Tannerellaceae | 99.99% | e5c61a1521d944dc477589e9ce5a2236 |
| Family | Weeksellaceae | 92.10% | 964ea87c411631553e928a2406b1bb89 |
| Family | Weeksellaceae | 92.10% | 90acce5030dc9fb4130b80e0d538347b |
| Family | Weeksellaceae | 100.00% | ed22fd63f4c809514b80cd549ba0515e |
| Family | Weeksellaceae | 99.99% | 32bf7d0c4687d594c369853e8fadc54f |
| Family | Weeksellaceae | 73.82% | f22c01a5efcbfbd3b0c882e1c70ec47d |
| Family | Weeksellaceae | 92.58% | 20d43041362dcad8949e18f870e2f79d |
| Family | Weeksellaceae | 90.48% | 34fa726650e74f6ef85823568061ff32 |
| Family | Weeksellaceae | 89.81% | 0592028b5f9e7551f23ceb7f2fce601d |
| Family | Williamwhitmaniaceae | 99.28% | 05c76092a706fbf3b8dba19de355faf5 |
| Family | Wohlfahrtiimonadaceae | 100.00% | 680b454926edae42a844729b12bdbd78 |
| Family | Wohlfahrtiimonadaceae | 100.00% | d910e2c373fc2fcff28b6d554de6f46f |
| Family | Wohlfahrtiimonadaceae | 100.00% | b06e1f8e14ceb64ec7d8b056f7cd1e51 |
| Family | Wohlfahrtiimonadaceae | 100.00% | 465fe775b0a01383fe5e4c8a8de6e230 |
| Family | Xanthobacteraceae | 99.54% | 1588ef3f5b0996361691ccbf13a9a0a0 |
| Family | Xanthomonadaceae | 87.93% | 12e16b0083b3698ef9c6bd2b902e21cf |
| Genus | Clostridium innocuum group | 99.96% | 414b92eba277238e76b12c687fc90cbd |
| Genus | Clostridium innocuum group | 99.85% | a2abb2730495e527c36c3a18c2f9ccdb |
| Genus | Abditibacterium | 99.79% | a8b1c40d5e7b6612d50d9e9eddc21581 |
| Genus | Abditibacterium | 79.54% | 42e510570ccfbdb27713acfe4fc66c6d |
| Genus | Abditibacterium | 100.00% | 32056940e4c843906baf38572bef891c |
| Genus | Abditibacterium | 99.92% | b9506b516407519482da7a7f814f8774 |
| Genus | Acholeplasma | 74.07% | 9dd6b6cf482a592935e53cfd33993f25 |
| Genus | Achromobacter | 73.27% | 8cb783bb8a374250cad8f28889b158c9 |
| Genus | Acinetobacter | 99.09% | 7bd362a445b5a7ef8c24818b0963c7e4 |
| Genus | Acinetobacter | 98.47% | 18387e9cf1f471d949814462f1f3e43b |
| Genus | Acinetobacter | 98.94% | 0882ed2f3b40de8d8c1319b94074fd73 |
| Genus | Acinetobacter | 99.11% | 8f28784533cde436c39673c5b55cefd5 |
| Genus | Acinetobacter | 100.00% | 1b01b7a711ee4a484960b5d189521b28 |
| Genus | Akkermansia | 99.67% | 051c594e7ca64f7414a75016500ef30a |
| Genus | Alistipes | 100.00% | a76a007d7c92707d153c4433606ae339 |
| Genus | Alistipes | 100.00% | 758e4d0cb9b9de8e32b12e6228c1ee99 |
| Genus | Alkanindiges | 96.99% | e0ed4f477d969e8c6a20b4d6da36ce58 |
| Genus | Anaerolinea | 94.86% | 4bcee44d376eb2c03d5a201c83b4f506 |
| Genus | Arthrobacter | 77.72% | 4cee0c7e19503e767114a210e4abd6e6 |
| Genus | Aurantisolimonas | 71.48% | 4b110641915c04315f236af262550974 |
| Genus | Bacteroides | 99.99% | 5e188cd382c47fd8a7abadf2f47c9b99 |
| Genus | Bacteroides | 100.00% | c41290aa09743e6c4f369ae7f1cfee19 |
| Genus | Bacteroides | 100.00% | d96ee4ebcd458f092546c5b97755bb3b |
| Genus | Bacteroides | 99.53% | a6c13e16024131345b05007716792f50 |
| Genus | Bacteroides | 100.00% | 933f5136a5cca1b98fae46e5c5d99683 |
| Genus | Bacteroides | 100.00% | 18eda008fc98bd54a9b9bd10a18b4fea |
| Genus | Bacteroides | 99.99% | a7e992401860b4b0c37491ffb0976f79 |
| Genus | Bacteroides | 100.00% | 1db1711d9a1d7e897ee874fff05fbc3d |
| Genus | Bacteroides | 100.00% | cdf13cca19d9f8175b066b8846ae6eda |
| Genus | Bacteroides | 100.00% | 5cba65b9b9d17bb7f130073ded039473 |
| Genus | Bacteroides | 100.00% | 23611d31ad49cffe0b20b2fa9931aca4 |
| Genus | Bacteroides | 99.99% | c8d9df2433f193acc7f752c205f35614 |
| Genus | Bacteroides | 98.81% | 22db4b09d645312b71a5107f53e71be8 |
| Genus | Bacteroides | 99.74% | 86dfc62fc898213cb03251d110ffc226 |
| Genus | Bacteroides | 100.00% | d5a2371fac20c903880c092a4aabae06 |
| Genus | Bacteroides | 99.53% | 292bd05b6cbcb3ed898c552c2e7eec26 |
| Genus | Bacteroides | 99.69% | 287449441f2df1d221e571eeb4635578 |
| Genus | Bacteroides | 100.00% | eda905e5483c24a1bc3b4157d5284225 |
| Genus | Bacteroides | 99.54% | b9dc4a70dd2cccba4a054abfff63b1a2 |
| Genus | Bacteroides | 100.00% | a095ad1f8a168d65e5b35ed1d31a6c73 |
| Genus | Bacteroides | 100.00% | d3510f58db56383e0c60b55f0374ff4e |
| Genus | Bacteroides | 99.64% | 926e6c460261607f5ed84d208ecc431c |
| Genus | Bacteroides | 99.54% | 8340355293c3b3db2e3796f867f14613 |
| Genus | Bacteroides | 100.00% | 158f8a976142c01d0613d520c266e26c |
| Genus | Bacteroides | 99.99% | c8d6611db6c36a061e7eaceef6630fed |
| Genus | Bacteroides | 100.00% | 7b64b71541e360493144cf77aa00e44d |
| Genus | Bacteroides | 100.00% | ab1d20253205afc4550550b509be7e27 |
| Genus | Bacteroides | 100.00% | acf9fc36df7011a88f5dcf52ec5932dc |
| Genus | Bacteroides | 100.00% | bd72ffb15bce38631319ee1136310731 |
| Genus | Bacteroides | 100.00% | d3deeb017a9596b51b5cf88f34f014c9 |
| Genus | Bacteroides | 99.97% | bf383e6f51525e128f953bc29be72f70 |
| Genus | Bacteroides | 100.00% | 114a27fc1bd54ac6eee0b0e588ade19f |
| Genus | Bacteroides | 99.60% | a21ef53629bfb746f7d1439df0b2f4a7 |
| Genus | Bacteroides | 99.74% | 9ea1561a0632fc6ac8077aa0abf9cc18 |
| Genus | Bacteroides | 98.77% | 310792c5a7a0db4922cb1ee7f050165a |
| Genus | Bdellovibrio | 98.34% | 66f2b76c265f049ebf0766766fe7fce0 |
| Genus | Belnapia | 81.66% | 7a8f821d11ff91d3e921ff73ccab97b7 |
| Genus | Brachybacterium | 99.58% | 032fb7be4a3b9bcd2f5129a3d6914601 |
| Genus | Brachybacterium | 99.66% | f0b76d3dc006662c65c77ba934a3ae21 |
| Genus | Brachybacterium | 99.65% | 7596489a8f7cf7913e0e379658cc8fd2 |
| Genus | Brevundimonas | 99.98% | c5692042e40b7c07ffdf88d1b34e2444 |
| Genus | Brevundimonas | 99.34% | 2f781c54d85b54711c326d8de4698c0d |
| Genus | Butyricimonas | 88.92% | 6cb8d0aa2bd317b56a60d9956dc4871e |
| Genus | Cerasicoccus | 97.89% | 84c3d3b75a03636048a79413fa608178 |
| Genus | Chitinophaga | 98.05% | a772833519ccea08ee25dd0245c978d0 |
| Genus | Christensenellaceae R-7 group | 80.44% | 21e9bbb33812178f353b8b0055d4b41e |
| Genus | Christensenellaceae R-7 group | 92.67% | f5cbb6744f6e0a7e4dd22914fa739522 |
| Genus | Chryseobacterium | 99.99% | 16910584212df2dda3c68970a6af9d96 |
| Genus | Chthoniobacter | 99.68% | e42283c39c6ad4ead261cfa1f50c4c86 |
| Genus | Comamonas | 99.80% | f3fa144a2da7a593ff95c7d76de90e66 |
| Genus | Comamonas | 99.52% | fb9e987bede20b32e6c5ec64faa60947 |
| Genus | Comamonas | 99.90% | d16dc578f0226ea2cad1a86fdec3bfc3 |
| Genus | Comamonas | 98.09% | f2fd12da9bb31a70242fb2f7717426f0 |
| Genus | Comamonas | 99.15% | 2e51dc22cda8d25c1e26f06911709988 |
| Genus | Comamonas | 95.69% | 3bce9c35a120acdb24adaafafa5e83e0 |
| Genus | Comamonas | 99.72% | 4b43b948c89fc07c3c7d4c185a2b5cf0 |
| Genus | Comamonas | 94.85% | 2f4884f17d0d89983aae13eb2725b347 |
| Genus | Cutibacterium | 99.89% | f4f44aed827e8f00132c866312e51673 |
| Genus | Cutibacterium | 96.78% | a3b16882af684210c244602e7414de41 |
| Genus | Cutibacterium | 96.94% | 13d05bd3516320874529c76aba55a25d |
| Genus | Cutibacterium | 96.78% | 1ceb8120b377df8daec6164b58c85d19 |
| Genus | Deinococcus | 100.00% | 9162ba36abe7b0974378cb73a1591c9e |
| Genus | Deinococcus | 100.00% | 104054faa0fdcaf8b823d12327a6d062 |
| Genus | Desulfomonile | 93.27% | be54d8139445f3102db25822c137d0d1 |
| Genus | Desulfovibrio | 70.58% | 81baa8bd7dd144b6a66acc0ab2544ad0 |
| Genus | Desulfovibrio | 82.93% | 4f1fc441a0a4d1e1c981055de7ad98f2 |
| Genus | Desulfovibrio | 97.28% | e550d2bef5bcd847433f4a3ecc16ee28 |
| Genus | Desulfovibrio | 93.60% | ae29f03f5e6bd5ca6acfcb52376440fa |
| Genus | Desulfovibrio | 96.98% | c36758e304004381f4eb31ee775610ae |
| Genus | Desulfovibrio | 97.18% | 3ea9ab8ca78c2a1bf51f00e6864022a5 |
| Genus | Desulfovibrio | 97.89% | 567b3ad5d63585acfddea51cbc83c3dd |
| Genus | Desulfovibrio | 76.23% | 13a5e5a7bb05aef08c74a39d4aae3679 |
| Genus | Desulfovibrio | 99.96% | efe2f098fac9ae9c7a642b1def6e43cd |
| Genus | Desulfovibrio | 99.99% | 12d78a003c34256dc06198e306ccaff3 |
| Genus | Desulfovibrio | 99.37% | 89c236e1f1b50df3eaa0e3a36263eea0 |
| Genus | Dickeya | 99.17% | f7bbc998592423dd9904210b6774b0d4 |
| Genus | Dickeya | 98.92% | 12e9e4f6fb389c4290f0ee863f8c113c |
| Genus | Dickeya | 98.97% | 5816a5a31b0721f3d9182a4b46bf0233 |
| Genus | Dickeya | 99.18% | 1a1b0b41f134baf3d27c04430449587e |
| Genus | Dickeya | 99.07% | b190b0eeb47f70ff5bd5015f8b7e0325 |
| Genus | Dickeya | 98.98% | 63b6c1b4693668853e1c49bba7dc633d |
| Genus | Dickeya | 98.92% | 5c6288e670d73c94afab9f276cd9d10d |
| Genus | Dickeya | 98.80% | ed1cea3f2bff616a394a529cf51ef7d1 |
| Genus | Dickeya | 98.97% | 7aebc79c7d4b918604b94658655985b1 |
| Genus | Dickeya | 99.04% | b82f08b60b9c647f180d146400d10d6d |
| Genus | Dickeya | 98.85% | 09c826143d80b4df453f3d9edb89504a |
| Genus | Dickeya | 99.21% | b4258c5f3b98e0cbb7b8f14e199de9ee |
| Genus | Dickeya | 98.98% | d2e8a901416ed47c060c35eeb55fb524 |
| Genus | Dickeya | 98.85% | 46be1f393740e225b54dea4a56deab41 |
| Genus | Dickeya | 98.98% | 932c27e80104c0b6a1c710de59a00509 |
| Genus | Dickeya | 99.04% | 10c5b6ced4704399dd1b6cb6ac5b2d4c |
| Genus | Dickeya | 98.91% | ce5b1709104079bd5f848ab09f91f94d |
| Genus | Dickeya | 99.21% | f5feaba919eaf5cd786c6eb810f93c74 |
| Genus | Dickeya | 98.98% | 8b0a60ffbec2356a2b6c58d7442f1ff5 |
| Genus | Dickeya | 99.73% | 90bb43cdff07599e144986641e6d87ef |
| Genus | Dickeya | 97.38% | 8c7daa31f83011d21e741ed42524dfc0 |
| Genus | Dickeya | 96.35% | 87b6549fdc0d1539a9c0925137df2227 |
| Genus | Dickeya | 95.66% | d5824a7364ff6f1b27afa0abdcce917a |
| Genus | Dickeya | 95.68% | aaae3b8a244183500532a64530f9981d |
| Genus | Dickeya | 96.88% | 1fcd83f168af6be5b53af14ae1109076 |
| Genus | Dickeya | 99.94% | 2c5a44525aa567d58309bc325e1a2271 |
| Genus | Dickeya | 96.90% | 42f09af5b5656a4e9368caf96105939b |
| Genus | Dickeya | 97.37% | 156b73af5133ce1ea970cdb65d9c4ef0 |
| Genus | Dickeya | 96.86% | 9c9bca39f28dfe379725805dbf9ca5bf |
| Genus | Dickeya | 99.93% | 854c80c931bb31efad21179d6e906f73 |
| Genus | Dickeya | 99.92% | b5243d9b27c7e3342ccabf896e8e294e |
| Genus | Dickeya | 93.12% | 9f05bc4e4842d4044330b4f0a31c9ad4 |
| Genus | Dickeya | 93.05% | e897b63e724576b15e8fae9997375ac6 |
| Genus | Dickeya | 99.80% | 902af8e753f3d1e173877ec5dbfb8b9f |
| Genus | Dickeya | 91.89% | 6b5fb8a72a275ba8ca4433e61401da51 |
| Genus | Dickeya | 99.76% | 2af3ad172274a5518fa2aa9752f59208 |
| Genus | Dickeya | 99.76% | 0b6ce48800006c5a2ed4de1bef97809d |
| Genus | Dickeya | 99.94% | 32982489e1f4ff57a4f9c0db426e946f |
| Genus | Dickeya | 99.92% | 6e46dd59ddccb8f4627e7514d9c0c94d |
| Genus | Dickeya | 99.93% | 540ea6e7e293f7a98b70db095a6fd69e |
| Genus | Dickeya | 99.92% | 07a4ad46bfa93021c7daba56558f8c6b |
| Genus | Dickeya | 99.93% | 548d86de11be7c91b82c09764c550afb |
| Genus | Dickeya | 92.87% | 5905eb4d97dc3ff63b965ae7d0032f78 |
| Genus | Dickeya | 91.81% | 14e22462351a6b4e05e07862bdaac947 |
| Genus | Dickeya | 99.94% | 845c9a08c4eae050c4794cf87a2cf36e |
| Genus | Dickeya | 96.84% | 52b57562beacab3bb982462a1dfcaa9c |
| Genus | Dickeya | 96.26% | b07240161241df091dd76c975a8a5ed8 |
| Genus | Dickeya | 99.37% | 25bb14137555f3dc23d279956d9439b0 |
| Genus | Dickeya | 99.80% | 90cac311949869433916d61d4d003739 |
| Genus | Dickeya | 99.76% | 0f613af3332c5de5f134b14cd98ccc7f |
| Genus | Dickeya | 99.92% | 6d272c4001912a0282b5f9edfd42074f |
| Genus | Dickeya | 99.76% | df612ff411ce76797011fd22ea3e9241 |
| Genus | Dickeya | 99.87% | fd1eb73a40601a60ec806b0ff3886166 |
| Genus | Dickeya | 99.93% | b7ebd78530f2b7daf9a7c1c5bb2e7601 |
| Genus | Dickeya | 99.85% | 76e0ec95ae6608285a9f3f46f354b135 |
| Genus | Dickeya | 99.85% | 1b88bb19520d48a36a4a04658103ce8f |
| Genus | Dickeya | 99.99% | 17926028f316f4c77aab0fee503b0c63 |
| Genus | Dickeya | 91.59% | a55a52e092abe231d2a454d3f68b08e3 |
| Genus | Dickeya | 99.99% | 08a68ff746f1ee276ddd0bcb74957364 |
| Genus | Dickeya | 99.99% | 8d41f3efdaf29cdb350313cde9607646 |
| Genus | Dickeya | 97.38% | ea6df2ec8cf6ffc36f6893487c492de9 |
| Genus | Dickeya | 96.18% | d2036f2af04334c6cf94cb97ca7e8fdb |
| Genus | Dickeya | 91.21% | fb036c0d8e8ea8296b2e123858865f21 |
| Genus | Dickeya | 96.87% | b463c525f6a2e706a3a19565b077a1ee |
| Genus | Dickeya | 99.82% | 55331187a1fb7343d3f4495c13e45581 |
| Genus | Dickeya | 99.87% | 9e61f53d4c32ccb9020a1daee6665e0f |
| Genus | Dickeya | 91.17% | 63eb165dafac73fd4750e39226a393f4 |
| Genus | Dickeya | 98.91% | 9785604d6199b1cb1347abfc6464bbff |
| Genus | Dickeya | 99.99% | f428b1f1e564445823e193f90949d091 |
| Genus | Dickeya | 99.67% | 8ef50fa0ba0ee6d9118bc0561cd66637 |
| Genus | Dickeya | 87.57% | 82d5f6ad08b6938a30748284091ac35f |
| Genus | Dickeya | 99.94% | d339e115feaf2b837f4ba0a3e9a1e32a |
| Genus | Dickeya | 93.89% | 1b9d4f5b2938c4b9f812d42afa12f3ac |
| Genus | Dickeya | 99.99% | 40f0b8c0047b8c8258cd924507650994 |
| Genus | Dickeya | 99.92% | fe478a32ae3fbe2212dad2b502502faa |
| Genus | Dickeya | 99.90% | 30dc6d45b26e3c86097dfd235e38cbde |
| Genus | Dickeya | 99.99% | f0a94907d1d4d2cecdd8b08941ff669f |
| Genus | Dickeya | 99.99% | b6194c213c7b779f3cc2381c4b69e1c3 |
| Genus | Dickeya | 100.00% | 58345435d271c27090c083db42367c91 |
| Genus | Dickeya | 99.98% | 6d736cd66aa2acad82643f896da50167 |
| Genus | Dickeya | 100.00% | 2628e119b47a62bdaf33eaea665a8171 |
| Genus | Dickeya | 99.98% | 3c990133e5b0b6459a95d34170c06a70 |
| Genus | Dickeya | 99.40% | 540b1740144a9fff00175addf5ed51d4 |
| Genus | Dickeya | 99.87% | cded731407a224d8cc8c21d1152ae9da |
| Genus | Dickeya | 99.13% | 946a52b8fdedaa97f250758e1db8271f |
| Genus | Dickeya | 77.57% | a57527e60d2f98a445d35f016ed693b5 |
| Genus | Dickeya | 99.96% | ffd10dd4c9f6a114e31cc03905830be8 |
| Genus | Dickeya | 94.56% | 1b3a2cda2cb9a9f3e0aae72ada8bcdec |
| Genus | Dickeya | 98.89% | 290f44a62cbdf705bbbb6fdb966445d2 |
| Genus | Dickeya | 96.28% | afa0319db7005e25e21705fbfbe71668 |
| Genus | Dickeya | 100.00% | 482dc504c7b8ff26e1981809f8bb1f61 |
| Genus | Dickeya | 80.61% | 1ad36a4051f1a061f65466fcb4c4cba9 |
| Genus | Dickeya | 99.99% | 034ed39696a687a222f91a0e1be6b4ba |
| Genus | Dickeya | 97.26% | d3e965bb362126d9efd56e19ea1434f5 |
| Genus | Dickeya | 99.91% | 69688da499575504bf0c726830dbff93 |
| Genus | Dickeya | 87.49% | 86311bfd829465a95088390e3d62a8b1 |
| Genus | Dickeya | 99.99% | 7a492fda9abfaff823552f1d084dbbe6 |
| Genus | Dickeya | 99.99% | 37b195eaa85fd703eff534f08b5e8471 |
| Genus | Dickeya | 100.00% | 6c010da9ce64e034bd40e1e7628518b0 |
| Genus | Dickeya | 99.94% | 0f9d4aaa763df0aa3f03968c44fefd36 |
| Genus | Dickeya | 99.97% | db5af9c181de396bba77c377597a1cb2 |
| Genus | Dickeya | 99.95% | 8575566e4a6f723077859a0d00812eba |
| Genus | Dickeya | 99.95% | d35df9ee2e375dfe2b25d103ece32358 |
| Genus | Dickeya | 100.00% | 1b9ecbe3d31ba57488c8eb1f9c5372ec |
| Genus | Dickeya | 99.92% | 1e2d1fd258b6a821b6d106b63c5830ef |
| Genus | Dickeya | 99.99% | c82398777359ab3f9a51eb8e4c45622a |
| Genus | Dickeya | 100.00% | 836f679b7ab891dca7ade67f4570ff53 |
| Genus | Dickeya | 99.94% | b8f108499bde412cb5f1fd899b9bb452 |
| Genus | Dickeya | 99.99% | 0b952aa8d9ca9248983a848e15effa1c |
| Genus | Dickeya | 96.33% | afc194e05db06a14af390cf8e8fbc3c6 |
| Genus | Dickeya | 99.83% | d19ac461827be06b714fa05e9385be2a |
| Genus | Dickeya | 99.95% | 37c252b36d89c9bd582af5c677135bee |
| Genus | Dickeya | 99.99% | 3e4b54ec0093c86d06c143159a7a1559 |
| Genus | Dickeya | 99.93% | f034b02e19abe7dfdb5ad60e56c7bf19 |
| Genus | Dickeya | 99.99% | a5b2c147f8ba68d4458cb165bbc93665 |
| Genus | Dickeya | 99.99% | 5adf958f8fad900bbde5533d9de2cb63 |
| Genus | Dickeya | 89.38% | ae6c892a775a08eec4edf1f5d2999cd9 |
| Genus | Dickeya | 95.68% | 2967d96439f2edf0a6dc8388b5e15ff3 |
| Genus | Dickeya | 99.93% | da3166926f29c2b05cf8d6fe936557b6 |
| Genus | Dickeya | 89.91% | 568f29400b76d7da2406fbd4746cf95b |
| Genus | Dickeya | 99.83% | 20148c7120914eafa1e2f5be7c734822 |
| Genus | Dickeya | 100.00% | cb34ebaf6632d0ad08023e80d35b0bc0 |
| Genus | Dickeya | 99.80% | b913ae4679333f768c06fbab1ccb4fd8 |
| Genus | Dickeya | 91.44% | 480646d1fd120ea6c82c134e236b0a23 |
| Genus | Dickeya | 97.03% | a1cb7bf9d3750f7b24a8a0d85799e285 |
| Genus | Dickeya | 99.95% | 976ea920b2ad406f27592d5210c4c970 |
| Genus | Dickeya | 100.00% | 9b68f4273510302c1e9749bd6acbcb70 |
| Genus | Dickeya | 99.86% | cc5adf47460a13300ab19210b372088c |
| Genus | Dickeya | 90.03% | 55201ec39b46ee1b05b56e4a9ec052e0 |
| Genus | Dickeya | 99.96% | bc6a3a2678a57905fdfd664b82adda53 |
| Genus | Dickeya | 99.07% | 2271683f629adac0c579f81f4dda6b3e |
| Genus | Dickeya | 91.24% | 65bd9a21cc59e6a2cee4daef85c1cd8b |
| Genus | Dickeya | 99.86% | 5f80cc01dee3e5cc34ae23b91fe7bd70 |
| Genus | Dickeya | 84.03% | 2556b345067ec6f2e68ab09fffe85504 |
| Genus | Dickeya | 99.95% | 796fb7e3a757fec50dbd230b95da7b56 |
| Genus | Dickeya | 99.92% | c4d287b969fc960fd42fadc52d67f6cb |
| Genus | Dickeya | 89.38% | f4a04fd1916c08a640034e89df1e1711 |
| Genus | Dickeya | 99.85% | c0e6338eb7c2b5ff5f2895a141fb264f |
| Genus | Dickeya | 99.88% | 67b53da336eadaca3f2ada34b9466ccb |
| Genus | Dickeya | 78.67% | 9e0fe3d51bc3385d4372b5bf3b403c2f |
| Genus | Dickeya | 99.87% | 24412eb4b5283e883a98069d276c31d1 |
| Genus | Dickeya | 99.94% | be6a8fcc52fa4fa2b7afad968c201acb |
| Genus | Dickeya | 100.00% | 4b6edd4820f3c1be1f64e70dccee91ba |
| Genus | Dickeya | 99.92% | d624b488b65e773c27a08ca5746015f8 |
| Genus | Dickeya | 99.92% | 24ff919b7db9a07690aa8b14c461039f |
| Genus | Dickeya | 94.56% | 9153026097a648e1d7c14eec52430fb7 |
| Genus | Dickeya | 99.97% | 8da6115fe79d14407a75f3285aeac701 |
| Genus | Dickeya | 99.92% | 5cb7a2779bcb8ceefaf66f15f33740ec |
| Genus | Dickeya | 99.97% | 1121752e0d63b5cee91c1d3aff5e4016 |
| Genus | Dickeya | 98.85% | e5f4669610c292d9667a3e1cd44dc35e |
| Genus | Dickeya | 80.83% | 8126cbd8e1b9d8d87c5f5067a3444ee9 |
| Genus | Dickeya | 99.99% | 219720600c1e8825d6a56cc630e5b391 |
| Genus | Dickeya | 99.75% | e302fc6fd9f8e9d4c765b10b4409754f |
| Genus | Dickeya | 100.00% | 9aca997e9fcd322d151f2f6f52e46af9 |
| Genus | Dickeya | 100.00% | 83b3df022e52e1e448051f9f903f21a6 |
| Genus | Dickeya | 99.98% | 1f6692e575e37c00f84e14cee3de3836 |
| Genus | Dickeya | 99.95% | 5b688e755786ed7c4cac051b713c7a51 |
| Genus | Dickeya | 99.80% | 22de124d008c2d430a42c2d28c41d016 |
| Genus | Dickeya | 99.95% | 2dd5313974ee5f4004ecd86f138ea058 |
| Genus | Dickeya | 100.00% | c0971af48c5261eb25944e98aaaf1003 |
| Genus | Dickeya | 99.90% | bc97469a98baf9eb473813abb80420b6 |
| Genus | Dickeya | 100.00% | a8a85fa73112d9efd2a782cd64a6b59c |
| Genus | Dickeya | 99.97% | 77c9a00957423fa6757b9e4e061b6ed8 |
| Genus | Dickeya | 99.98% | f8abcf1221dcdebacd42e34f66f496a9 |
| Genus | Dickeya | 99.86% | a4e9628bdab1ebf58a03596b26583bed |
| Genus | Dickeya | 99.91% | 3a7640643c69919a0354113b8f60aa8f |
| Genus | Dickeya | 93.01% | 3e6c822458eaab57a18d18d53ba79e1e |
| Genus | Dickeya | 99.99% | 0d66c4d12d7594a64f425dee610fd7c0 |
| Genus | Dickeya | 100.00% | 8c36b50866699eec72d0583c4198139e |
| Genus | Dickeya | 99.97% | c9163419fc9be508fd7377f03470435b |
| Genus | Dickeya | 99.93% | b7df8702ed1a005e695e2e23fc3fada3 |
| Genus | Dickeya | 91.28% | 18be5fee78efbb765c86efc17ba66921 |
| Genus | Dickeya | 99.97% | 93bb04b2255ac04c4453352b577003d4 |
| Genus | Dickeya | 99.96% | 20930ab094a656f1bee7333810bff995 |
| Genus | Dickeya | 99.95% | 726280bd24860da68d562e4820e19498 |
| Genus | Dickeya | 99.94% | 906cb268c4d48724ba52cb05a356b9dd |
| Genus | Dickeya | 99.95% | 134bf467c9e90259b4d13be7a3dae2cf |
| Genus | Dickeya | 92.47% | 09acf9580d5b7eaecbee380638fa8f15 |
| Genus | Dickeya | 97.41% | 10578b62310d8f10a38c8b022b1bb250 |
| Genus | Dickeya | 99.93% | e2a0eaae836b0e22fb81b118e576bf39 |
| Genus | Dickeya | 99.99% | d0408cac0a17796187c02a0c1fb06035 |
| Genus | Dickeya | 94.56% | 0bf9a93ee36151c492e0fc52dc0fbf17 |
| Genus | Dickeya | 99.83% | ad43b8f9ad4e78c2ee3d1e6ddfaaf66a |
| Genus | Dickeya | 99.92% | 21fc63fae94700c3dca44f297d0ba281 |
| Genus | Dickeya | 89.59% | 7314be00c61696a949ed5fc1b0cafc85 |
| Genus | Dickeya | 99.92% | 6205544386dbfb9dcf31e47888f9ea7b |
| Genus | Dickeya | 99.90% | 8bb2dd157546875939d05f48f0db8572 |
| Genus | Dickeya | 99.99% | 981c791af9c1cde052a25d03fe7581bc |
| Genus | Dickeya | 99.89% | 4c0b08ea9e38a57fa39285bbe8fbf8f4 |
| Genus | Dickeya | 99.99% | f5a8e66e1131e75278f79081b8dee562 |
| Genus | Dickeya | 99.88% | e0f6d9137e44da02f4719fa725d4d85b |
| Genus | Dickeya | 99.98% | a04a068654a4eb203acb3fd5102b1c2b |
| Genus | Dickeya | 99.74% | 3abd1fdeac27770f9fd41ecd378a5a36 |
| Genus | Dickeya | 95.41% | dca39e0f09bde098ea2713b7a86fc4e4 |
| Genus | Dickeya | 99.96% | 4da1965ea616b4a957611fa42b329060 |
| Genus | Dickeya | 99.92% | c7d08c7077d04a70d70ae59dd81d61f4 |
| Genus | Dickeya | 98.73% | 5f9d5b9abb9b0f4f9a0355ea8378f34d |
| Genus | Dickeya | 100.00% | d92d74c420a8004643182987df6dcba4 |
| Genus | Dickeya | 99.59% | 8fb241fa7f0086816cd6df96d9df0a64 |
| Genus | Dickeya | 100.00% | d49dc6018fd0652f2b38abf50bffbf20 |
| Genus | Dickeya | 99.87% | 3895f5e785528717e1269abbfc55fe62 |
| Genus | Dickeya | 99.90% | 9c2d0e123489e8cba045913716cb31d3 |
| Genus | Dickeya | 97.74% | 8f08e103347fb2c2c2956a2968a28552 |
| Genus | Dickeya | 99.99% | 851838d3ad344b85118853a08e368012 |
| Genus | Dickeya | 100.00% | dd325e46dd5e323edbd7a255a1f8ad8b |
| Genus | Dickeya | 99.70% | ecf11db855a0b8a86bc4b76d664a625c |
| Genus | Dickeya | 99.75% | c09dbbde80fa953a95ff989d9a98ac6d |
| Genus | Dickeya | 99.86% | 54c9038e6fca86c7fe5b0c6e7b9086bc |
| Genus | Dickeya | 99.80% | 2b092a764f9b91a166641335a7f78425 |
| Genus | Dickeya | 99.80% | 67feec896ffe5ecc49d2201e8d49d3c9 |
| Genus | Dickeya | 99.83% | 56935a7841b116267cbe2b6eec30150a |
| Genus | Dickeya | 99.50% | 9ec4e00bdca2c03a59e75961c409582a |
| Genus | Dickeya | 100.00% | 468ccc88626371fafa2c3b55f2841b3c |
| Genus | Dickeya | 100.00% | d106f10297ee56b8b824c6655e7ee777 |
| Genus | Dickeya | 99.80% | cc5832e39858a1aee7d9e41f153f1da0 |
| Genus | Dickeya | 99.93% | 5987edf79aac41d88b9fbd3dd4b06312 |
| Genus | Dickeya | 99.99% | 8671f8c6c129d335f8e90441c70e585b |
| Genus | Dickeya | 99.95% | 72bb9959bf4ab8f8268542850cbb4db4 |
| Genus | Dickeya | 99.97% | ea62f7865a4528c4da3346cf117a159a |
| Genus | Dickeya | 100.00% | f1d7d20b410d0c7faed44781cd4be0fb |
| Genus | Dickeya | 90.20% | eadf0f06fcd00584c561932237df18a2 |
| Genus | Dickeya | 99.94% | f21376ca252a44b98126370aa90e4a89 |
| Genus | Dickeya | 99.96% | 0057f898c9b2be6104e9bbdc7391d906 |
| Genus | Dickeya | 100.00% | d7e93323ec4b51845dfde2029bbfa652 |
| Genus | Dickeya | 99.92% | 1280efc3a833fedf1c825ee64f9a4352 |
| Genus | Dickeya | 99.97% | a1fac3af0e70908f0531ecae2ce2cd08 |
| Genus | Dickeya | 100.00% | dd922bce2ad1fcb9725c5910b6ac5d06 |
| Genus | Dickeya | 98.59% | 50ded981f6f6ee7e90d1d9698b7e7856 |
| Genus | Dickeya | 98.32% | 132ee9a62bfea39ed7682f2a0d76a03c |
| Genus | Dickeya | 99.98% | 84fe0d94a883eec69e02f510e2dabf66 |
| Genus | Dickeya | 99.97% | f2b48b1f50eb992c87337bf66b4e00ab |
| Genus | Dickeya | 99.91% | b8b775adb739a5202f25799a0ae11751 |
| Genus | Dickeya | 99.92% | 1fdb225ee43fe8d8e519f24ea5393bbb |
| Genus | Dickeya | 99.86% | 6fc73926144b8f14ece282c0b4c290e9 |
| Genus | Dickeya | 98.19% | e9116479bcc4c0b1704d2b78088281ba |
| Genus | Dickeya | 100.00% | 28e597bb4ae539e50425b2f677376677 |
| Genus | Dickeya | 71.02% | 46ca262d7383c9310827898ebe1262c0 |
| Genus | Dickeya | 98.90% | 59f3b41e35e3517e4a23433696cb4f9e |
| Genus | Dickeya | 97.91% | 3d16fd257fe0cc052b9adb51ac7ad7ab |
| Genus | Dickeya | 99.98% | 2b1871b0de13e643ffe4f928f7376d0d |
| Genus | Dickeya | 94.18% | 29fb866371329293fe542fca01ae4e2a |
| Genus | Dickeya | 99.99% | d69a22b95a96480981bf3de5125bc730 |
| Genus | Dickeya | 98.81% | b364cb5cdd6ca4515be42d5dad9d1133 |
| Genus | Dickeya | 85.16% | 822ece07f6fc633fe3b8e2f91d4f3002 |
| Genus | Dickeya | 99.96% | 881882cd7e3f8e991dd3c262ded68eda |
| Genus | Dickeya | 99.94% | c46262744d3758b696457b3f992513bd |
| Genus | Dickeya | 99.96% | 6acfebdaf3aa6eefe16b69b72b807ba5 |
| Genus | Dickeya | 99.97% | 28798fd6480a210d1f12752fa0ceaea9 |
| Genus | Dickeya | 95.95% | 2b71f1d78cc73aeb59b71a492ed65e0a |
| Genus | Dickeya | 99.77% | 9ba8630944964156e026a1c35a084f54 |
| Genus | Dickeya | 99.33% | 9f7da9a147d27e111f16aa3a3aa6f3f9 |
| Genus | Dickeya | 100.00% | fc20f2408d96948f2f8ceec4b4c3d8a8 |
| Genus | Dickeya | 77.40% | 387a298b37315afd1dc299ea249d9068 |
| Genus | Dickeya | 99.96% | d8c3d2c5b5a4b11e4556c5c1dcd8cb8b |
| Genus | Dickeya | 94.85% | 61d0dde81752d419b28d780b50bb5bc0 |
| Genus | Dickeya | 97.67% | 04905942d2cb6967fe2b639ac07575d6 |
| Genus | Dickeya | 97.90% | 9619665ed4739a655237b7679c9571e1 |
| Genus | Dickeya | 97.88% | e25327ca3926e2129b07119f0395bb1c |
| Genus | Dickeya | 97.93% | d7d3ee8c00cbb60f9e782d6544da1487 |
| Genus | Dickeya | 98.68% | 1870fde9255f0f07ac2e1f1841f05df4 |
| Genus | Dickeya | 99.93% | dce884fb6c68cfe37f49e7c19a33a14f |
| Genus | Dickeya | 90.59% | de2f051cbee340d98e8a6331a05adca2 |
| Genus | Dickeya | 97.97% | fe13e1f1dca6413c709c3c43d84a382d |
| Genus | Dickeya | 85.92% | b7f93079338737cdf3edc927f1a7da85 |
| Genus | Dickeya | 98.75% | f3184245604f437ed6acbccbc696ed6d |
| Genus | Dickeya | 99.89% | b61ec58eab6b019f8fa2f2c684ea5a24 |
| Genus | Dickeya | 99.89% | 39ac916eea2d4ba3bbdd6b9dfa513a05 |
| Genus | Duganella | 97.08% | f4e40369f5329aaf0f4657a71f5b72aa |
| Genus | Dysgonomonadaceae | 99.98% | f32bc1276481c00317ea446841c9c1c1 |
| Genus | Dysgonomonadaceae | 99.92% | a58a5676864ef541fd709afccd2da700 |
| Genus | Dysgonomonas | 72.78% | 7c3a11d94d8e884cc4f81e4d077d3a06 |
| Genus | Dysgonomonas | 96.54% | 073dafa7ac10668669aef84557e28408 |
| Genus | Dysgonomonas | 99.89% | a97854dbaf7e50381532f153178a02ff |
| Genus | Dysgonomonas | 97.56% | a0ed91ce73911275c3e563ec5e71c048 |
| Genus | Dysgonomonas | 71.56% | 17003bda04897c7708ece03da0bf3a72 |
| Genus | Dysgonomonas | 90.12% | 59a4dc39cb7943b574f37c9e01e784be |
| Genus | Enterococcus | 99.09% | 199837dfbc969128a5356c896cc188fd |
| Genus | Enterococcus | 96.62% | 9f94b5065f41b5d16571ecc4b6160083 |
| Genus | Enterococcus | 87.54% | 8a96ea4d4e901a03cc6d11dfdb022301 |
| Genus | Enterococcus | 96.73% | 938b4c15e7a286a3888af9af1eeb8e17 |
| Genus | Enterococcus | 96.66% | 0d0a33d21e09d83e4099b2c87b9f9def |
| Genus | Enterococcus | 96.51% | 65b91a923ca1c53f399bcfb3858e4e33 |
| Genus | Enterococcus | 96.46% | 3f01aeccbd92c1305593af043afeeead |
| Genus | Enterococcus | 99.08% | b6e8242a62bad762c1f00e78fcb21b85 |
| Genus | Enterococcus | 97.03% | d2b5cab3d5f3f69cfc9b0efb05e9e99d |
| Genus | Enterococcus | 99.14% | 6851d0df97039d1a69d6652e744e4dd6 |
| Genus | Enterococcus | 99.15% | 730e79b87611add37f746ae6b10d8887 |
| Genus | Enterococcus | 96.62% | a42f225d259885ec2a655fcd2c76517d |
| Genus | Enterococcus | 98.12% | a41826073f966772c3da62c4fef6bb05 |
| Genus | Enterococcus | 99.18% | 441d02bad57b8f8ce75c531c4f1076d3 |
| Genus | Enterococcus | 86.69% | a623d46842a0fb8daa7a450325041c70 |
| Genus | Enterococcus | 96.94% | 1186974e6f12400931d731722dab6396 |
| Genus | Enterococcus | 96.70% | bf4e822b5cbad1528c56753f9028cba2 |
| Genus | Erysipelothrix | 79.13% | 39c689425ce1fa80244d9f1a917b38a0 |
| Genus | Erysipelothrix | 75.77% | c484d59e3bd2f49133322b3bd6e93230 |
| Genus | Erysipelothrix | 75.69% | 109757324af150ae73272f83e3f92fec |
| Genus | Erysipelothrix | 72.83% | df5aa14309c52af70ce772972f2dea25 |
| Genus | Fusobacterium | 100.00% | d8d18fcd2755ef7ee6732a4cf80518ab |
| Genus | Fusobacterium | 100.00% | 18bd3f294f8e05c6624bf70f32a8b2fa |
| Genus | Fusobacterium | 99.81% | 01e5ce19471349234481709ade192476 |
| Genus | Fusobacterium | 99.73% | ca4a24cfc21810cd7fd154b57e121232 |
| Genus | Fusobacterium | 100.00% | d1b6e59a665f83abce1aae8639ac471f |
| Genus | Fusobacterium | 99.84% | 44c3c4bb3e7506f83737ff8242a8f6ff |
| Genus | Fusobacterium | 100.00% | 9fc10607c800d7d0e2e76a79095355c7 |
| Genus | Fusobacterium | 100.00% | a618338c08d51dbd4d36529b5826b105 |
| Genus | Fusobacterium | 99.98% | bf9f408f5e596843b03f7db2e9d0d6ac |
| Genus | Fusobacterium | 77.75% | b0ac52a1af7c7e6791eb728682803640 |
| Genus | Halomonas | 80.14% | f2885a4af47dbf047bddeba62cefcaa4 |
| Genus | Halomonas | 80.05% | 2aec735001d67636017d54cce4560b99 |
| Genus | Halomonas | 80.14% | e4dd77e2dc6224b1181b2a436de0a1d0 |
| Genus | Halomonas | 81.28% | 4b4c087cb5c3d3f59c149d7bb833332b |
| Genus | Halomonas | 85.72% | 4b1144d1d3227bfbf5d5f18c8a8e3360 |
| Genus | Halomonas | 85.73% | 15c711bf391c646d21821134b06f34c9 |
| Genus | Halomonas | 80.01% | fe0dd2ac7168a45141b715e9aa5637a8 |
| Genus | Halomonas | 80.03% | b9026efe7ee754994ed400770bd093dc |
| Genus | Halomonas | 95.49% | 69e9026a1387c26e29b7a951aa662111 |
| Genus | Halomonas | 76.23% | 92395a6ba4b4df5120bc6af34fa55db2 |
| Genus | Halomonas | 85.72% | 60f07087e973378fd332a4fbe67ca188 |
| Genus | Halomonas | 80.08% | 4181df311dfbef48b5356d92e4f8c429 |
| Genus | Halomonas | 85.71% | 5401c677ebb34ee57eeed2ad200b7ef9 |
| Genus | Halomonas | 82.49% | 325de694f4ead8e5f2e5fc7383009087 |
| Genus | Halomonas | 76.14% | 176a0861d0ff72280001be4f5ec0848e |
| Genus | Hungatella | 74.75% | 6b1a990ebb6c54c8988ea4402a17e7bd |
| Genus | Hymenobacter | 100.00% | aa3fce77ee5be0d8db418accc84e6f66 |
| Genus | Hymenobacter | 100.00% | a48a1788a348a68b6b357c734fc2e39b |
| Genus | Hymenobacter | 100.00% | 603054bc1ce41ae95c8c7d72cb83e983 |
| Genus | Hymenobacter | 100.00% | aff517d66380fd46745322f1c28da51f |
| Genus | Hymenobacter | 99.63% | cfd235b0294cda484c7facc3332baab4 |
| Genus | Ignatzschineria | 100.00% | 6eeb19ad217e44a1d9f1c3672d8574a3 |
| Genus | Ignatzschineria | 100.00% | 3dce234105be5d54cc7debf7a042b8b7 |
| Genus | Lachnoclostridium | 88.96% | ac26d8d57d7654c96f74205dc420af0f |
| Genus | Lachnoclostridium | 98.74% | 4ab33b955c716644aabf94626958578e |
| Genus | Larkinella | 99.99% | 1e1818e5a691045150ce2891094343c1 |
| Genus | Leifsonia | 75.95% | 20f07cce575680b11d6eab72bec556ab |
| Genus | Leifsonia | 76.76% | 18138f98122247583038ce20bca21bdb |
| Genus | Leifsonia | 76.02% | b0272b459b5cca183f773cd664a797aa |
| Genus | Leifsonia | 75.99% | 33c2a753fdebe4b775d8b6a559c9096d |
| Genus | Massilia | 99.93% | 5d40daebcf82458d96910b832ed36d72 |
| Genus | Massilia | 99.94% | 388fe08cf2d177a3c9461415654e6e13 |
| Genus | Massilia | 99.52% | ee8a6db5fa4bcd4ebd4bc80fa4d36f5e |
| Genus | Massilia | 99.93% | 00d427ac8a16adac492f4dc9b9fbe3f5 |
| Genus | Massilia | 99.93% | 37842e73bd8807bfb35697716ed24d1a |
| Genus | Massilia | 99.94% | 43d97df6025dab34da5e0008d05d2124 |
| Genus | Massilia | 93.92% | 2216387acc4003ac0e3a822ed1dd3161 |
| Genus | Massilia | 99.73% | aadd73ad2159adc5c994e4a24e063c51 |
| Genus | Massilia | 99.93% | 611c8255a915554c488ea8ae85d3c189 |
| Genus | Massilia | 99.93% | f330c64120a6eeacf01f84b15f011793 |
| Genus | Methylobacterium-Methylorubrum | 99.99% | 14a7bf8c7f97b442f5e1d17c13008cd5 |
| Genus | Methylobacterium-Methylorubrum | 99.99% | 9591e5d7df6bf4bcfd339190d4b96b9f |
| Genus | Methylobacterium-Methylorubrum | 99.98% | 137f90af9f988d4c445bbb486c233455 |
| Genus | Methylobacterium-Methylorubrum | 99.99% | 7a54c8e445b842218a489b7d5e309d66 |
| Genus | Methylobacterium-Methylorubrum | 99.72% | fe07e7e889d8550f185e555d053f4e89 |
| Genus | Methylobacterium-Methylorubrum | 99.99% | b6b0d5873ba8e72d2da89bd6e1b4fbe3 |
| Genus | Methylobacterium-Methylorubrum | 89.09% | 06e1d2b37c8d7d93e56b0c03609ba282 |
| Genus | Microbacterium | 90.76% | d0aa71b351fe588bb6afd318d7b55929 |
| Genus | Muribaculaceae | 98.45% | 03e56adf77dbb807dd7a3233f14d67f8 |
| Genus | Mycoplasma | 100.00% | 0ab15634508b35ab615a9a783d73a0ff |
| Genus | Mycoplasma | 100.00% | 52aba174e3196ef122a1634627f6fccf |
| Genus | Mycoplasma | 100.00% | f99c3247705c1575e5d82aa650d69e71 |
| Genus | Mycoplasma | 100.00% | e072d37ef26b63b4c42ae94fda6f0e75 |
| Genus | Mycoplasma | 84.47% | 8658bb42034e86f201706654854b2e7e |
| Genus | Mycoplasma | 100.00% | 32a9c4656b7347e020ebcbbd7f4eb7ea |
| Genus | Mycoplasma | 97.82% | 384e51fb942135e27f3619684602e583 |
| Genus | Mycoplasma | 73.50% | c599dbe0a9ec5a6b518d9783bcd8ae3d |
| Genus | Mycoplasma | 98.63% | 7ac14211b9ef9a04443cd102424def3a |
| Genus | Mycoplasma | 100.00% | b2875945263efeb4e5d718fd7f9d1891 |
| Genus | Mycoplasma | 100.00% | de911a9f2476987336ea1cf57291cafb |
| Genus | Mycoplasma | 100.00% | a7ee3c34ab63af470019a6d074b3b469 |
| Genus | Mycoplasma | 100.00% | d79ee5904d6e739fdd0f690a2b6a74d6 |
| Genus | Mycoplasma | 96.28% | 192e6a05b4a5bdbe5beff1981ac4f4cd |
| Genus | Mycoplasma | 100.00% | b544d241645b3389f04cc34d11ceb87c |
| Genus | Mycoplasma | 100.00% | 74237c2d49233df86a17287b3f374634 |
| Genus | Myroides | 96.82% | c25bc05550bb917dd748db44c709d000 |
| Genus | Nocardioides | 82.93% | dfbae5e8db097cbff8a74f13bc4d1d1d |
| Genus | Nocardioides | 74.69% | c891127854d195e317617c0895ea9099 |
| Genus | Nocardioides | 97.39% | 143f7dc8f7dcff4a96f4215b970cd515 |
| Genus | Novosphingobium | 96.35% | 925945142c3b81fd5acbeaf25c2e4e2d |
| Genus | Ochrobactrum | 74.42% | 85230871537c0ac0f3830a6fd8f0ac4e |
| Genus | Orbus | 73.16% | 455a29fccff0feb1028123d91049bb2b |
| Genus | Parabacteroides | 79.89% | fdf6dcca91d76fd126fbf2596f83e44b |
| Genus | Parabacteroides | 100.00% | 18079a1b9bf9912bb00df2dd0278ca4e |
| Genus | Parabacteroides | 98.77% | 0e2d9449dbac054e26be5d0a90f4bb44 |
| Genus | Parabacteroides | 76.79% | 0308a8b7d5e490b32b830494f8d1aa75 |
| Genus | Parabacteroides | 74.94% | 9c1eab4bfef33ed26dda2af48eb4936e |
| Genus | Parabacteroides | 99.56% | 9d61059f98f80b6a7846a8f91c6799a3 |
| Genus | Parabacteroides | 99.97% | 5298714075c9f104bc2fb4a206d1b4c0 |
| Genus | Parabacteroides | 99.99% | aed2b1076a0b54a82e6d99c4a934120f |
| Genus | Paracoccus | 76.06% | fe4a71a0eb5d6313020de2ce9a40b6e0 |
| Genus | Pedobacter | 97.35% | f69df7e15b2b39cca43c1827e690cb6e |
| Genus | Pedobacter | 97.36% | e94874a901c66cc2c6fd462fa5917e43 |
| Genus | Peptoniphilus | 99.00% | 7b9e74c11b16b47bbe61014bd88e2e5f |
| Genus | Phascolarctobacterium | 71.00% | afd363d43bc712be45fef64eda13c90a |
| Genus | Phascolarctobacterium | 71.09% | 47854c3d41ff687b172d936b506b2cce |
| Genus | Proteiniphilum | 98.48% | b1e750d53d3885024286a761dd7dba5f |
| Genus | Pseudomonas | 91.97% | cc756225c8492a3d79c5e89e5fff2471 |
| Genus | Pseudomonas | 95.93% | e2ff5b16430ae09e7dc0d5153136b10a |
| Genus | Pseudomonas | 94.16% | b680cd497192af0ea33d2e8fc3819826 |
| Genus | Pseudomonas | 94.21% | 85cb3c2a3f213b6ceb0ee8de01c5e0cd |
| Genus | Pseudomonas | 88.75% | 078c918e119457a547826ab073a1d26c |
| Genus | Pseudomonas | 83.44% | 2cbb81553aeb8e5a48f01e66d0c9c525 |
| Genus | Pseudomonas | 99.74% | 1372622ea06ef763ddc0d5e8fd3d3baa |
| Genus | Pseudomonas | 96.98% | defd221ecacc2d712fe430cf36dcd11b |
| Genus | Pseudomonas | 99.33% | 44c60a05e738384017a1ca2e794193c2 |
| Genus | Pseudomonas | 83.14% | 021526c443deaa5473532f913c0d43a2 |
| Genus | Pseudomonas | 97.64% | c9cfbf65b7fa97ed7793a74113e4ffe8 |
| Genus | Pseudomonas | 99.28% | 3969b9981cf74bee3c2c25c29d018b97 |
| Genus | Pseudomonas | 86.44% | 81c67751d12c75b1abdd7c1cd9d4e703 |
| Genus | Pseudomonas | 99.38% | dc7843b15857015c23e9d4c9692ae099 |
| Genus | Pseudomonas | 84.26% | 26bb12e1123a26f057dffed705b71c10 |
| Genus | Pseudomonas | 90.37% | ce9021f7e18d6be23e490ecd2baf5010 |
| Genus | Pseudomonas | 99.15% | 245d9882b797d1135e5411999435fb9b |
| Genus | Psychrobacter | 78.14% | e9bd5988ee46e7ec3086b4f289711a47 |
| Genus | Quadrisphaera | 70.02% | 882a7b37ed41c9ffee77d0cb77188e45 |
| Genus | Quadrisphaera | 74.26% | 84d7faf5a45d65a188ae22b2543858b5 |
| Genus | Raoultibacter | 100.00% | dfe423b354081242abab139e680e11ce |
| Genus | Rickettsia | 95.66% | db569ba071fa72bbbf5144aeed358e31 |
| Genus | Rickettsiella | 99.40% | 7597608464ebcbbae223e774cee2e5cb |
| Genus | Rikenellaceae RC9 gut group | 71.57% | d8c3753d0570fde45b2da69703144200 |
| Genus | Rikenellaceae RC9 gut group | 70.64% | abd575ced35313bf5719f46b9a20ff88 |
| Genus | Rs-K70 termite group | 98.21% | 0a28577d488c120007d7420f41c635bf |
| Genus | Segetibacter | 75.42% | 24f36b254921d9ed2eb835fa5e6b7956 |
| Genus | Sphingobium | 97.23% | e92a8a6c81cfad6d8b172d83b4f15fe7 |
| Genus | Sphingomonas | 99.14% | 6581f2d5e43d72db1159f34180e4e38f |
| Genus | Sphingomonas | 98.92% | 722c5a3e58aef125b5228f82c93a4a78 |
| Genus | Sphingomonas | 97.68% | ef7a610e2fd06583f8e06eea9c838656 |
| Genus | Sphingomonas | 98.80% | 5f62f98b0f63a30d74ca16e10d2ac747 |
| Genus | Sphingomonas | 99.11% | a2aecc9bebf3eeb50ed24c76abc14ebb |
| Genus | Sphingomonas | 99.00% | 33b857811e238cfb419e32319cde15e3 |
| Genus | Sphingomonas | 99.37% | b680ed3b7845117d84d5a48baca96467 |
| Genus | Sphingomonas | 94.64% | 846e6e659197b4b61f9384eee84b03a4 |
| Genus | Sphingomonas | 97.45% | df6f4ec33db8c91759b275e4616cc58b |
| Genus | Sphingomonas | 99.32% | 046c19a3f3b77ec3ca4fcff2ff1f0122 |
| Genus | Sphingomonas | 99.67% | 1e925e3cb374d5f3f713921546d9a9b8 |
| Genus | Sphingomonas | 95.78% | f92ccc633c2ae9e143bc8ab5fd24f925 |
| Genus | Sphingomonas | 96.95% | 78172c3cfd995f25dc7973c7ce6a18c0 |
| Genus | Sphingomonas | 94.13% | d9ea9edfef835f4a423c3ac8372916a1 |
| Genus | Sphingomonas | 97.99% | 98b5d49d5280ce2d4170c8eca9957730 |
| Genus | Sphingomonas | 98.64% | b3937d174b147a918dab2e0c7110e132 |
| Genus | Sphingomonas | 98.98% | a577b1bd0da6db79dc9c6f884f0c21ad |
| Genus | Sphingomonas | 98.79% | 0ee12308b78a6055cfad57ca4a9387d5 |
| Genus | Sphingomonas | 98.99% | 841f9c9fb165504c5901476c99f1b1f2 |
| Genus | Stenotrophomonas | 99.75% | 995ae13c4a44ad1587e90e6a98a43b4d |
| Genus | Stenotrophomonas | 79.40% | 3fa1963f5948e0adf6c63b598327aca6 |
| Genus | Stenotrophomonas | 92.96% | 758ba8f879ed18aa6b0482dda3edd1d8 |
| Genus | Streptococcus | 73.40% | df4071ef4063d5281fb77a39eb5adade |
| Genus | Subdoligranulum | 71.50% | 25fb46275aa72ac83b2224977db58844 |
| Genus | Subdoligranulum | 79.77% | 15de4605965aa110667be31f12c46807 |
| Genus | Sutterella | 81.09% | 53957d34e541d94cfad66cd808072d1f |
| Genus | Sutterella | 92.33% | 0b02ec4c4a84ae3f397c67549aa9fef5 |
| Genus | Tepidibacter | 90.83% | 8e1f5234121901a30fb2b60178f1c5db |
| Genus | Tepidibacter | 99.31% | 6b19cb93f276b4ea1dfa5abe4499c6b4 |
| Genus | Tepidibacter | 92.08% | 759af98cbffc00513d478d0ba626f0e1 |
| Genus | Tepidibacter | 99.89% | a6ee2b7b11fa2b20b0dff02a865ea3ac |
| Genus | Tepidibacter | 99.61% | 651a8fcce302d9cf706a3d2bf51ee056 |
| Genus | Terrimicrobium | 76.05% | 2c37a9aed679101f7e9e4e7ba2ce41af |
| Genus | Terrimicrobium | 85.32% | 7cde17aeb3ec849e7998860a83007103 |
| Genus | Tissierella | 98.89% | 2abb797cf686cd283db3dbb158cf38f3 |
| Genus | Tissierella | 98.22% | 316b30b1c638c43cb64a2f9ffcee838a |
| Genus | Tissierella | 98.56% | bde6f4615633a3fba0a9aaa34c89cd8a |
| Genus | Ureaplasma | 99.60% | e399bdf2b31b8c4329f5f0de91cac663 |
| Genus | Vagococcus | 77.40% | e2c691d384947d5a9a5d252a8365f815 |
| Genus | Vagococcus | 96.05% | 50a3aa3d6ed5d8c20a9b93ccd65776c9 |
| Genus | Vagococcus | 96.04% | dbcb3b62741eaf5ea1976fa1004ff28a |
| Genus | Vagococcus | 81.52% | 88b03d8a564be644c04b662c08b6c73c |
| Genus | Vagococcus | 78.09% | 3c709ff2361b972cfc3e7eabcd8505d8 |
| Genus | Vagococcus | 81.52% | 251809f182ebccec382ec903f77f80ed |
| Genus | Vibrio | 74.75% | 06a55bd9e16cc555cb6bc6593f005888 |
| Genus | Weissella | 100.00% | 0679e095cfba30268ade72503333b97e |
| Genus | Zymobacter | 91.58% | 6083285ba95147a7dfd1d6874215aca9 |
| Genus | Zymobacter | 91.49% | 817b083b30b59a2bae9a2210c6b47479 |
| Genus | Zymobacter | 91.61% | fd4eef00430f9c23e329d0369f79acbe |
| Species | *Clostridium colinum* | 90.82% | 4a1022127250417f1dc068e4fa0706c7 |
| Species | *Pseudomonas geniculata* | 100.00% | a6c7d7013c0b7746d7dc0aee6d790d75 |
| Species | *Pseudomonas geniculata* | 84.27% | d373494891190e2f0a15b59577b85a74 |
| Species | *Pseudomonas geniculata* | 95.39% | 500a304512fc42d696945d732402e5a4 |
| Species | *Pseudomonas geniculata* | 78.81% | d08a2fab1f257917d7607a2ac9a48571 |
| Species | *Pseudomonas geniculata* | 96.20% | 9d7613fd6079d49a36370760d770d073 |
| Species | *Pseudomonas geniculata* | 98.26% | 225f9057832ffc48a7edfd9bc56ad79c |
| Species | *Pseudomonas geniculata* | 99.99% | 352533bd5d8174a34be0de814f2aa048 |
| Species | *Pseudomonas geniculata* | 100.00% | 15be7db67bce9df6ccc3a80c6b159d50 |
| Species | *Pseudomonas geniculata* | 99.98% | 3a8e0dd0cb90030dd187932141bdce73 |
| Species | *Pseudomonas geniculata* | 97.03% | 1d3a5d213690a05a8efd246fa00bfa1c |
| Species | *Acidibacter ferrireducens* | 84.48% | 1993c26531463e52fb6e0a886aa7535e |
| Species | *Acidobacteria bacterium* | 71.00% | b5cbd05e82621a94a33ad29663c21720 |
| Species | *Acinetobacter indicus* | 75.25% | 673684063906ad7b66687748bfddca9b |
| Species | *Acinetobacter towneri* | 71.53% | f9d57a301e88538d48cecc989504809e |
| Species | *Acinetobacter towneri* | 70.50% | 7c1ff39ca09125c431127cf90ddfa748 |
| Species | *Aerococcus sp.* | 99.88% | 2a43bb21878f18d4f9d48af175cebd6a |
| Species | *Akkermansia sp.* | 96.94% | bd04d37aa807e55e40d50413e68dbc0b |
| Species | *Alistipes finegoldii* | 97.80% | b1f10bc3618c8846527530eed578a2d8 |
| Species | *Alistipes finegoldii* | 99.82% | ac441333d6657e0ca0cabe718fed2bce |
| Species | *Alistipes finegoldii* | 99.82% | 816d84070ba4669c88e67397e13459c8 |
| Species | *Alistipes finegoldii* | 96.76% | f921b410630a1bbb698f00336987cd09 |
| Species | *Alistipes finegoldii* | 85.22% | db2e67d19139ff81fdead54cb267f67a |
| Species | *Alistipes finegoldii* | 95.45% | 71e84cfa2c715370ecd9159c4bde1ee5 |
| Species | *Alistipes finegoldii* | 91.59% | f092d34fa67b2a1d54af887e43185aa9 |
| Species | *Alistipes finegoldii* | 99.85% | 186fdd7365c0441cfb31e2c563fb143c |
| Species | *Alistipes finegoldii* | 81.42% | 0f8caf0dbe6e7c3a7fa68f03e72eb6c7 |
| Species | *Alosa alosa* | 70.80% | 0ef2dda873865841cb6f66d235e8719d |
| Species | *Anaerococcus provencensis* | 98.09% | f785f17975aab570186cc46a4383d96a |
| Species | *Apibacter sp.* | 96.81% | d33824ffc0dcf9798c9685867ae1e16a |
| Species | *Arachis hypogaea* | 82.40% | 2383d49241941eadb2df119d26c95c59 |
| Species | *Asterionellopsis glacialis* | 85.45% | 188ca78d0aa1b87be6827209d89559fb |
| Species | *Bacteroides acidifaciens* | 81.59% | ab6fbea079385cca432d72958cb1d145 |
| Species | *Bacteroides acidifaciens* | 81.57% | c9a7f72e762cf0d5d66b1e104c176370 |
| Species | *Bacteroides acidifaciens* | 81.57% | 793d6c799f748781300362ecdeb3c0fc |
| Species | *Bacteroides acidifaciens* | 81.75% | b30c4194a9076646bfb8b0426199f3d6 |
| Species | *Bacteroides acidifaciens* | 81.62% | 198922977e8f5bff56e6a4519ebefb3b |
| Species | *Bacteroides acidifaciens* | 85.01% | a62beb8c113721b0362097c476ddf049 |
| Species | *Bacteroides acidifaciens* | 80.40% | c37054e2a145e9c1c86c2c56f44bc535 |
| Species | *Bacteroides acidifaciens* | 84.93% | f81aced774d43b235403a18ad69c0a1b |
| Species | *Bacteroides acidifaciens* | 80.23% | 71cfff45411941c0d590d55d5f117b37 |
| Species | *Bacteroides acidifaciens* | 87.12% | f9a59ee5bc92e02c6a4ca912d83dee10 |
| Species | *Bacteroides caccae* | 92.64% | d850fbe2521f7556b7986f166eade5b0 |
| Species | *Bacteroides caccae* | 92.51% | 1be415d932c000799145d96a417efe74 |
| Species | *Bacteroides cellulosilyticus* | 71.10% | 4e53c1fff423dccdfed9a19ce62cb2aa |
| Species | *Bacteroides cellulosilyticus* | 73.96% | 2fa5e71e021d230e5c931e61cae1df3a |
| Species | *Bacteroides cellulosilyticus* | 90.74% | 6a07dfe6101042ff335160ff345240e0 |
| Species | *Bacteroides coprosuis* | 100.00% | 5c1e210a30d163215d5b46c8c4856d57 |
| Species | *Bacteroides coprosuis* | 100.00% | cd30eaf05513011519af593732a113dc |
| Species | *Bacteroides coprosuis* | 100.00% | 66094e0f7adb205367785086d82455da |
| Species | *Bacteroides coprosuis* | 91.43% | f1df06b97039304bfbe6b60db7476ed2 |
| Species | *Bacteroides coprosuis* | 98.54% | e785e49cc1c2d07fe4a5b0899c810652 |
| Species | *Bacteroides coprosuis* | 100.00% | 0a2cfd7fbdad05a700722e169effa2ae |
| Species | *Bacteroides coprosuis* | 100.00% | 319010b5fd47897dbbfbb04bcf6c0431 |
| Species | *Bacteroides massiliensis* | 99.96% | b389ad0295416a01c53e03783cb7fb7a |
| Species | *Bacteroides oleiciplenus* | 78.23% | 76d423038ac860b92f703372d3c0eb7e |
| Species | *Bacteroides propionicifaciens* | 93.36% | fe0964272d3b6b751ebf7fddb6d65121 |
| Species | *Bacteroides sp.* | 70.89% | 1a7fe9f20f242128320c67fff5ca3e3f |
| Species | *Bacteroides sp.* | 70.75% | 665a5063e21bdaf3ee449c6b5749ff28 |
| Species | *Bacteroides sp.* | 70.16% | 84515c0744a56d264e09b877484ebbaa |
| Species | *Bacteroides sp.* | 73.91% | c78d2e53b6696f90a8de6ca7b818a9c6 |
| Species | *Bacteroides sp.* | 72.29% | f73fee2936db6fb5ec5ef8a4783634c2 |
| Species | *Bacteroides sp.* | 77.33% | 372bc20413cfbbd7395f37a33c831229 |
| Species | *Bacteroides sp.* | 74.44% | f849b0b1e3e22e1676eba6fc2cbb884d |
| Species | *Bacteroides sp.* | 74.00% | 2ba44c718850998dc9b7a412644956ea |
| Species | *Bacteroides sp.* | 73.31% | d11df811cd73ea3afff2d4bbf45bad06 |
| Species | *Bacteroides stercoris* | 97.28% | 964ae73dd60ab555978837afbb0ec189 |
| Species | *Bacteroides thetaiotaomicron* | 86.77% | cc7ab79329a59043e3aed5db8202bdb1 |
| Species | *Bacteroides thetaiotaomicron* | 88.67% | 0821e7745e37471884595d5ac44f6a90 |
| Species | *Bacteroides thetaiotaomicron* | 88.63% | 30188e081e77769a3484aeded92bfc47 |
| Species | *Bacteroides thetaiotaomicron* | 88.63% | 268c68ccbb2c00037682711f21520534 |
| Species | *Bacteroides thetaiotaomicron* | 84.93% | 6907f1d90712abb06b18fcbb7b81497e |
| Species | *Bacteroides uniformis* | 97.90% | 3de114d89f5ef3586cb45c0613b6f782 |
| Species | *Bacteroides uniformis* | 99.49% | 7a270269524e86429f7f0cdb2a8af19c |
| Species | *Bacteroides uniformis* | 97.90% | 0bfc73e3c5110eb7e2536c8bbffdf4b4 |
| Species | *Bacteroides uniformis* | 99.48% | 7231d6d332b3dcf6547bf28bcb6bb089 |
| Species | *Bacteroides vulgatus* | 80.83% | 59361bb35a047b3d011b44e31235aaea |
| Species | *Bacteroides vulgatus* | 80.97% | 916005967616bfb93e9d94728c7d4d67 |
| Species | *Bacteroides vulgatus* | 73.13% | 898176c93314421708734269cfbae087 |
| Species | *Bacteroides vulgatus* | 77.75% | f5d642a427b449de21a21302802704d6 |
| Species | *Bacteroides vulgatus* | 70.79% | eb3f00ce00b491e7608c719f68204a8b |
| Species | *Bactoderma rosea* | 99.66% | 9369c8553e1cfe42d1839147e6551704 |
| Species | *Bergeyella sp.* | 78.78% | 08397ad00dba5d204c75b94aed9fa0fd |
| Species | *Bilophila wadsworthia* | 92.46% | fab196834ee96c75cfdd5893624e6454 |
| Species | *Brachyspira sp.* | 96.45% | 56341489588cb4ed0175406821b3a0ab |
| Species | *Brevibacterium permense* | 99.46% | 7806288847c366b64fe93472480afb41 |
| Species | *Brevundimonas naejangsanensis* | 81.07% | 4208f2da56bf2625b0937679de9358fb |
| Species | *Brevundimonas naejangsanensis* | 78.67% | 41f8b15a2cf5cbe2f79ef3a767962c9a |
| Species | *Brevundimonas naejangsanensis* | 79.34% | c8fb4526335b41514efeaa5772121ac7 |
| Species | *Breznakia pachnodae* | 83.99% | 1cf0ad1783ee2048a62bc59c8b985e57 |
| Species | *Burkholderia sp.* | 70.90% | 8d0c7a1ae73b693e333a84eb7d3df16b |
| Species | *Candidatus Fritschea* | 99.67% | 318926299badb10196e7a251afd63757 |
| Species | *Candidatus Rhabdochlamydia* | 71.63% | 49a9a52b925692e7aa40234c6db7cd86 |
| Species | *Cardiobacterium valvarum* | 93.88% | d0d8f356bff5768ac3b56ca8242f09f9 |
| Species | *Cardiobacterium valvarum* | 92.18% | ab23de616146157c1b93004d2567c05b |
| Species | *Cardiobacterium valvarum* | 88.40% | daa4096c2f378516f34a3aed8ef01075 |
| Species | *Cardiobacterium valvarum* | 86.68% | 7dee2db8c745def63ff6317ee9a9d1d0 |
| Species | *Cardiobacterium valvarum* | 97.73% | 0fa0c26e2c481fffa464b7500977761f |
| Species | *Cardiobacterium valvarum* | 96.55% | 92567f85302ca5eb29432343f002f997 |
| Species | *Cardiobacterium valvarum* | 98.53% | 807486d7d1ee0bf0f47c2e6d8595e06d |
| Species | *Cardiobacterium valvarum* | 97.26% | dacdc210831675f408612fd26a672bd6 |
| Species | *Carnimonas nigrificans* | 76.48% | d9fdd6f7cbb93dfd9936ba02e624023e |
| Species | *Cenchrus americanus* | 96.81% | b60252eb76e3ba7e39df03bc3744da9f |
| Species | *Cenchrus americanus* | 86.75% | 6818764be5110bb8859d4f719ee1e2fe |
| Species | *Chryseobacterium hispalense* | 76.41% | 45436d72e4cc68e25522cfffd20ea278 |
| Species | *Chryseobacterium hispalense* | 79.63% | 07f4df513dd4b55ca037b0d20b2dfae3 |
| Species | *Chryseobacterium hispalense* | 79.41% | b140eb1ec2b873b2449bcb92022c70be |
| Species | *Chryseobacterium piscium* | 93.58% | a6290c26e6a6f044578fbebfc9fdf17c |
| Species | *Clostridioides difficile* | 99.98% | 17309c08d5300e4cb88a5e2b74b8a302 |
| Species | *Colobanthus quitensis* | 99.95% | 85c2213172a0bb333c4072a4e6640578 |
| Species | *Colobanthus quitensis* | 99.94% | e5a1ff68b192557b713757b2bdbd89e2 |
| Species | *Coptotermes gestroi* | 82.61% | 7e31d2fb30ca079608fd95762ababf65 |
| Species | *Coptotermes gestroi* | 99.70% | 8514064bce5f4a5429ca1dcb3af696f6 |
| Species | *Coptotermes gestroi* | 99.71% | b75dad7538fc3ed45baeed07344ac314 |
| Species | *Corynebacterium callunae* | 80.89% | dc7af4373d9eb88abf0febcb2075ea86 |
| Species | *Corynebacterium tuberculostearicum* | 89.14% | 1d0f1b6267509f545f4ef98cb541e744 |
| Species | *Daucus carota* | 99.67% | 515bb92406e66eac10c9293fef495ca8 |
| Species | *Daucus carota* | 99.94% | 5b7d35eaf6b871fcc5732dd80d559238 |
| Species | *Daucus carota* | 99.67% | b18ccc34b36511d065fca9053b306dee |
| Species | *Daucus carota* | 99.65% | 307a9f756a72ef19df4920e74b130417 |
| Species | *Daucus carota* | 99.67% | 0e11e8ee3090848f13d95442eeba812b |
| Species | *Daucus carota* | 99.99% | 5a2d242a5599dd5bfb2d57825e0b0fe6 |
| Species | *Daucus carota* | 99.90% | 5a31106ecc8656748927098449c0a74f |
| Species | *Daucus carota* | 99.99% | c128ef79d5fd78af2f3bcc841bda3946 |
| Species | *Daucus carota* | 99.99% | 6d90c25a35824cb9bf8547cdb9bf411d |
| Species | *Daucus carota* | 99.99% | 9797cca18013069ad09ad0d52b51bf24 |
| Species | *Deinococcus radiomollis* | 79.36% | 34f93cb0ad3c09e4bdcdf7e632e53dec |
| Species | *Dolosigranulum pigrum* | 92.91% | 79ec83414a5dd668295b09e332b12843 |
| Species | *Dysgonomonas alginatilytica* | 88.51% | a1f77493f91d561e132ff4ae4d73f87d |
| Species | *Dysgonomonas alginatilytica* | 99.95% | 15c22030c37b8bc5a45d844f45c015aa |
| Species | *Dysgonomonas macrotermitis* | 80.85% | 01bd02069cedce9d19940e1ac880938b |
| Species | *Dysgonomonas macrotermitis* | 81.95% | e5c77bf7fb94cc24b85bac93d6b2f5ca |
| Species | *Dysgonomonas mossii* | 71.54% | 7f183e518f7b1082334610c045c2e7d8 |
| Species | *Dysgonomonas mossii* | 81.57% | 91aa23d445d5689f924e527a7429dc0b |
| Species | *Dysgonomonas mossii* | 83.34% | 201f61c6e20c96515613863531f97380 |
| Species | *Dysgonomonas mossii* | 99.76% | 6a10ddd08426a6560e8f7d06c3bb1e8a |
| Species | *Dysgonomonas mossii* | 92.73% | b47aa613250a0a96f95c1a6fc71fe84e |
| Species | *Edaphochlorella mirabilis* | 73.70% | 8c0a4e6509f38df362bbb6eeec050022 |
| Species | *Edaphochlorella mirabilis* | 73.71% | 148b20b04def44b97bbb3185425a8751 |
| Species | *Edaphochlorella mirabilis* | 73.02% | 66ed1044c1e0da663587381934a9306e |
| Species | *Enterococcus flavescens* | 71.61% | de658bed4810d2bf6fd8e584ba25a775 |
| Species | *Enterococcus flavescens* | 96.24% | a2e46f566ff73361079ba719d5954051 |
| Species | *Enterococcus flavescens* | 96.24% | c35084b2ef74f14e59b44146b8402123 |
| Species | *Enterococcus flavescens* | 71.22% | 3157b3131e434adefcd6753acacae5ac |
| Species | *Enterococcus flavescens* | 70.97% | af35fef875a0953eac28e0e6c4d75ae4 |
| Species | *Enterococcus flavescens* | 71.30% | e5e70e59164317ff9c35feeb73956d4b |
| Species | *Enterococcus flavescens* | 71.69% | 029427fe0d93a4c1458a293861ffd731 |
| Species | *Enterococcus flavescens* | 71.59% | d4dda34b25bb40ea08e98c0c1c2bfba8 |
| Species | *Enterococcus flavescens* | 96.23% | 2839059f8683e52e99b059fa50f27c2c |
| Species | *Enterococcus flavescens* | 71.27% | 55c3624f234d38428d7d878b016153ca |
| Species | *Enterococcus flavescens* | 71.28% | accf1f851a9ba775ef70f70343ac4576 |
| Species | *Enterococcus flavescens* | 96.34% | b0ee421bab4925e4d294ed16dbb4860c |
| Species | *Enterococcus flavescens* | 70.20% | 9f2a7d3000b2bcc33e2ccba09100c899 |
| Species | *Enterococcus flavescens* | 73.28% | 3363c35d4e375908b235ae2bf17faaa3 |
| Species | *Enterococcus flavescens* | 70.92% | 3c80b0e44195b9bbd3f6ddd69a2da1ef |
| Species | *Enterococcus flavescens* | 71.28% | ef84f30a783f47b7ff6e9519f148072e |
| Species | *Enterococcus flavescens* | 70.68% | 62d3cf43194780bf3949eb13cdedc2b9 |
| Species | *Enterococcus flavescens* | 71.69% | 7129aa96be7fd020fb3618f539b9652c |
| Species | *Erysipelatoclostridium ramosum* | 81.73% | e86da07f37dfef91c130030d71c4e743 |
| Species | *Erysipelothrix inopinata* | 88.89% | 10eeb5bcdfbfb79c70e1cd3ddcd23f53 |
| Species | *Ferruginibacter lapsinanis* | 97.66% | 39a500ed383046987e3da65e144b59c7 |
| Species | *Finegoldia magna* | 86.55% | 5a5730e7206aad6f93f22bdf81ef4b18 |
| Species | *Flavobacteriaceae bacterium* | 96.47% | aca488f4cc434d53ac6217c9d3edbc4b |
| Species | *Fusobacterium equinum* | 85.87% | c537d1ea07c153f6e8b6b2808a213fc8 |
| Species | *Fusobacterium ulcerans* | 90.66% | 63146d2fb9df0878ed6a125151b2cea1 |
| Species | *Fusobacterium varium* | 92.12% | 3c70d457cdf65a18142fa2385f2fa91f |
| Species | *Gemella sp.* | 77.19% | 5a42f4ab2967b8bb9c6e2f1c4ff31bd7 |
| Species | *Gryllus firmus* | 99.70% | 40c8a38cf0f61fed23b7e9313de966db |
| Species | *Gryllus firmus* | 99.62% | db008f7a3f15698ac6b686da0e1a5006 |
| Species | *Gryllus firmus* | 99.55% | 5c8e8a98b571b54232dc6c175b8dff2a |
| Species | *Halomonas xiaochaidanensis* | 82.08% | 06afdf77bab6622389036b6457bdeeaf |
| Species | *Heliconius cydno* | 99.97% | b91d910a06ca5d497f3bada6029ea983 |
| Species | *Heliconius cydno* | 99.98% | 3ec83fbe38b7b1740f9473c63994c86c |
| Species | *Heliconius numata* | 99.50% | 7f61aa69ee04f2735186b382b9904257 |
| Species | *Heliconius numata* | 99.46% | b2f6ff6d4dcfffe3d0cf331774b979a8 |
| Species | *Heliconius numata* | 99.56% | 455fc931d03c066e23b9683599edfc51 |
| Species | *Heliconius numata* | 99.51% | 4a3658c4df1423a6d2a90ab6b81afa8b |
| Species | *Heliconius numata* | 99.51% | 42d5596d0deba80553f8456391200ccd |
| Species | *Heliconius timareta* | 99.96% | d59e59438f92dbbbcfe9664db17fdde2 |
| Species | *Huanghella arctica* | 99.29% | 8641251cbce6f4ee96a9bdc5c9240151 |
| Species | *Hymenobacter rivuli* | 98.44% | 96e278925eba70aaba34458d500dd34e |
| Species | *Hymenobacter rivuli* | 95.64% | cf9a33e877dcf53fc1a6f0a8227957d2 |
| Species | *Hymenobacter rivuli* | 95.59% | c30e2aafd1902823b0bef3ca3bf39132 |
| Species | *Hymenobacter rivuli* | 83.01% | c04675ccf6ec58d36a00ecb1905cc1c6 |
| Species | *Hymenobacter rivuli* | 74.51% | 51bd2eb430a10bd9240889c69c4d0178 |
| Species | *Lachnoclostridium sp.* | 84.60% | 9e69606bf8685a23ada2bd9ca6503149 |
| Species | *Lactobacillus spicheri* | 94.77% | c88a4ebaf6d4f005dbc465e0a6487fdd |
| Species | *Lawsonella clevelandensis* | 94.49% | b2712cc646bcaebb8eae5345ece18f67 |
| Species | *Leptotrichia goodfellowii* | 99.98% | 7405563b23d5b8a48ace18a211c3c823 |
| Species | *Leuconostocaceae bacterium* | 70.60% | c3101c872b4204675ad788e718d5fb7f |
| Species | *Loriellopsis cavernicola* | 97.74% | f62fd48227ce646ba1a8b70cbf56e5d3 |
| Species | *Luteibacter rhizovicinus* | 85.45% | 454c7e509db3d49cf75d6d469d9832ff |
| Species | *Mannheimia granulomatis* | 70.73% | 1e8f89ce32e320a143c54916442e5b54 |
| Species | *Mannheimia granulomatis* | 73.67% | 7fee6864a1c5c21193a504e87a1eff7d |
| Species | *Mannheimia granulomatis* | 75.34% | df199ef2d2d9ed1b1f55b43a63cc7617 |
| Species | *Mannheimia granulomatis* | 72.20% | 4de93055b8d899d10e4a2bc2c33b72ae |
| Species | *Mannheimia granulomatis* | 72.87% | 8d976cd1b75ecbd0b55e98de3c1a04e8 |
| Species | *Mannheimia granulomatis* | 90.41% | 789af18968faecfc33f373b54a54a2d6 |
| Species | *Mannheimia granulomatis* | 90.72% | 5918c8052194b5e7de281a91d7ad2dc6 |
| Species | *Methylobacterium adhaesivum* | 99.28% | 8019285b0bc60327e30dd97a6151c0fd |
| Species | *Methylobacterium adhaesivum* | 99.24% | 3e1e7b76162d81595b3af625a90eb8c0 |
| Species | *Methylobacterium adhaesivum* | 79.67% | 403483a6695a2fb2bd1b52fbe724b6c1 |
| Species | *Methylobacterium aerolatum* | 99.34% | 1c0d36231f6a2fca4a975142af302a9e |
| Species | *Methylobacterium aerolatum* | 99.33% | 8fd55105ad4294e826b48b24769423d7 |
| Species | *Methylobacterium cerastii* | 83.83% | bd121066d2393e3a2677f44ffc5303e8 |
| Species | *Methylobacterium sp.* | 74.11% | 208bf8422d69807df28e10ce691862b4 |
| Species | *Mycoplasma iguanae* | 87.48% | 157bd650e654e8e01b53c70ed70fb65a |
| Species | *Mycoplasma iguanae* | 87.48% | bdb25c65282256c5418c4fff28f0c194 |
| Species | *Mycoplasma iguanae* | 87.37% | 3d32523b2a9adfeb255c807fcdde7f1c |
| Species | *Mycoplasma iguanae* | 87.48% | fdd0560020a12d8b47ed357f5c88cb33 |
| Species | *Mycoplasma iguanae* | 89.43% | cebf0d550968a49b4ddb393303797740 |
| Species | *Mycoplasma iguanae* | 87.47% | e0f8324cab2e6284ca34020e0539883f |
| Species | *Mycoplasma iguanae* | 87.47% | 780938a29af5ac7ce7ef2db30ac2c1d9 |
| Species | *Mycoplasma iguanae* | 87.36% | 5b5440eb3e03649837fb4c9f092e89a0 |
| Species | *Mycoplasma iguanae* | 87.47% | 70b42feeccd33a350c76b3eb94356de8 |
| Species | *Mycoplasma iguanae* | 87.26% | e848cf16579d76be3d08196e3a721b1e |
| Species | *Mycoplasma iguanae* | 87.36% | f10ed559858e4266fa7d6f9bf4b91d10 |
| Species | *Mycoplasma iguanae* | 87.32% | 804959db9ed87a62bdcaa3de1abf7a46 |
| Species | *Mycoplasma iguanae* | 87.26% | 503b41cf2d46e85c8bfa4f30631b3192 |
| Species | *Mycoplasma iguanae* | 89.62% | 99e756333f8e4c86e7723e50773df230 |
| Species | *Mycoplasma iguanae* | 96.16% | 6db94a89445524ae7e6af22cdf0a10e6 |
| Species | *Mycoplasma iguanae* | 90.74% | d1469b5658f75f431fab9af19696dbee |
| Species | *Mycoplasma iguanae* | 93.81% | 7d4a01af35412fd1e8d366e97aef0b37 |
| Species | *Mycoplasma iguanae* | 92.53% | 9b588e523e44204e00140bc27af6ecd6 |
| Species | *Mycoplasma iguanae* | 93.81% | 8a75081e7eb86592f258882de87d7438 |
| Species | *Mycoplasma iguanae* | 84.98% | b3fdaf3462c6f27143823c68c8034b92 |
| Species | *Mycoplasma iguanae* | 82.38% | db21e5457a513343eb693581b769f832 |
| Species | *Mycoplasma iguanae* | 85.10% | 929dc17ebdd9524864c4b9d5db8083f3 |
| Species | *Mycoplasmopsis columboralis* | 99.82% | e3b25bbe677b2d35ba949473a68c1dfe |
| Species | *Mycoplasmopsis columboralis* | 99.87% | 01d96ba67c21d6eaa4d01381b141b12f |
| Species | *Mycoplasmopsis columboralis* | 99.89% | 5c1af6372edfb35b5e2d070a226be7dd |
| Species | *Mycoplasmopsis columboralis* | 99.87% | bea7fe025b41276519672d98096cf65f |
| Species | *Mycoplasmopsis columboralis* | 99.91% | 4f6611d6a4db92f18258afe3231de62d |
| Species | *Mycoplasmopsis columboralis* | 99.88% | 38cb02922fb43babf221789cbb4bc1a5 |
| Species | *Mycoplasmopsis columboralis* | 99.87% | eab668dddad29526a8733c99c1bfcc98 |
| Species | *Myroides odoratus* | 99.28% | efb896c7d0f319067a1648327586b292 |
| Species | *Nocardioides halotolerans* | 99.02% | 438aee17c9765c4a91ec8721540f80c3 |
| Species | *Novosphingobium nitrogenifigens* | 90.95% | 7bba9813b3332552cfe1fac7f0041bcd |
| Species | *Novosphingobium nitrogenifigens* | 90.81% | 7e5fc29b2551d52c703d27d99c53242e |
| Species | *Novosphingobium resinovorum* | 77.58% | bebc638b41571fd9ab752f6a2ac3accc |
| Species | *Orbus sasakiae* | 72.11% | b29c0d1f0e0a1f11f47f8b77a7e5a433 |
| Species | *Orbus sasakiae* | 74.48% | a1e5ecf56988e99596b8f7ef8c984190 |
| Species | *Orbus sasakiae* | 72.91% | 16b1f6d8fc9dff2f8fa41269797c5a1f |
| Species | *Orbus sasakiae* | 83.51% | 9b975647c565972b3a53ed9c6184e27a |
| Species | *Parabacteroides goldsteinii* | 98.92% | d0d9cc298f3f4cff6c8da85426e58ae7 |
| Species | *Parabacteroides goldsteinii* | 89.25% | fd986a851fc5a0ba21127ca57e5ad00d |
| Species | *Parabacteroides goldsteinii* | 98.95% | e8c197d2d747433e74fe05c0fbdaef6c |
| Species | *Parabacteroides goldsteinii* | 89.34% | 7ab4c67b09e084e38c025b58d92cc278 |
| Species | *Parabacteroides goldsteinii* | 99.49% | 6f459d5bccd5d1798f101ce8eb5b68c2 |
| Species | *Parabacteroides merdae* | 74.68% | b57409e5539402149cada796c4313a51 |
| Species | *Paracoccus marcusii* | 70.81% | 03c10400f997e45e60a516888c528af1 |
| Species | *Pararhodospirillum sulfurexigens* | 80.12% | a79b2b635d92f927551014b0b065937d |
| Species | *Pararhodospirillum sulfurexigens* | 80.63% | dac8f991cfba8e3394c7348ebe2a0030 |
| Species | *Peptoniphilaceae bacterium* | 93.81% | 80370902244ca8e86e3c6ff499af0f32 |
| Species | *Peptoniphilus urinimassiliensis* | 71.66% | 290bb6f69035003dcbd2b77ff7cc5e3f |
| Species | *Peptostreptococcus russellii* | 99.95% | 9ec73bdf1ba1b99dcd0c2f136ce19cea |
| Species | *Peptostreptococcus russellii* | 99.95% | 6cdddb95ec64633e1a416cddeabae69f |
| Species | *Petrimonas sp.* | 98.65% | e046054a2f0c2ed72e149281599460fa |
| Species | *Petrimonas sp.* | 98.65% | 38e09d5f0970941b69e5d46c94112678 |
| Species | *Petrimonas sp.* | 98.63% | 6b3a1bf55671372d6c829c58875fd45c |
| Species | *Petrimonas sp.* | 97.94% | ec9aa5ae80b7d0fd849578fb4f0e06a0 |
| Species | *Porphyromonadaceae bacterium* | 99.12% | cb05ce8f232424dc1342dba283003640 |
| Species | *Porphyromonadaceae bacterium* | 99.14% | 889651aa157ee090d4298ed86ae8efdb |
| Species | *proteobacterium symbiont* | 99.47% | 56340783778cfd33f9b4509cccbbcf21 |
| Species | *Pseudokineococcus lusitanus* | 84.34% | 740ce49698c87c8f4b77c6c348803cd0 |
| Species | *Pseudomonas fragi* | 96.38% | 800d29dc295b5f04cbbb3adeec2ba8b9 |
| Species | *Pseudomonas fragi* | 92.59% | 6e1b1078c83b4a501e5d1e0c15949acb |
| Species | *Pseudomonas fragi* | 95.54% | 883b9022f2538140bd34640d03cd8327 |
| Species | *Pseudomonas fragi* | 94.97% | aad74b763f37a9acd6c30c74ec5f36c5 |
| Species | *Pseudomonas fragi* | 94.03% | 5688266ef6648614063558a0ddd48bcf |
| Species | *Pseudomonas psychrotolerans* | 87.32% | bdec2d5b488593069b84a5cbb6a52e09 |
| Species | *Pseudomonas psychrotolerans* | 87.17% | 1b844b220769216e1abc8519d020fcaa |
| Species | *Pseudomonas psychrotolerans* | 87.85% | 05053227687ef8355f75c663c58822db |
| Species | *Pseudomonas psychrotolerans* | 87.97% | 0429ec3794902197f1f0a2a88503aa57 |
| Species | *Pseudomonas psychrotolerans* | 86.80% | 7e1baf28106ae70e3f904262f977d627 |
| Species | *Pygmaiobacter massiliensis* | 99.44% | 787b8322d2162de5f2e80cfefb9a3d2a |
| Species | *Ralstonia pickettii* | 94.92% | 30bfb6f99468f66abfbd40228576bb77 |
| Species | *Raoultibacter massiliensis* | 90.98% | 2b510fefc55e55432923030df11e1429 |
| Species | *Rickettsia asemboensis* | 90.06% | 426458fa5ae5c7ff10c3fc796a4b6ca8 |
| Species | *Rickettsia asemboensis* | 96.72% | 6400d1319716f9f9c0cb314b7b0a324c |
| Species | *Robinsoniella peoriensis* | 86.28% | 136a7eae571a0bc117f3ab348daf5fdc |
| Species | *Robinsoniella peoriensis* | 83.11% | dbda0ced23ed218a314b5be90eb25a98 |
| Species | *Sanguibacter gelidistatuariae* | 96.21% | 08c65b12b7de343ecd04d59240cf9233 |
| Species | *Siccationidurans occulans* | 82.08% | 9ea572c37796d4090c72a2fb2db94a1c |
| Species | *Siccationidurans soli* | 91.09% | df8846bf2603c944a7cac5f1dd3f0658 |
| Species | *Solirubrobacterales bacterium* | 99.45% | ff9693817969af8a3f631f50b056ae34 |
| Species | *Sphingobacterium kyonggiense* | 80.64% | 51ee7ae813f58d64577f3a256b525ad5 |
| Species | *Sphingobacterium rhinocerotis* | 75.41% | 7c4eb996be03294d331ccb0f6d1ebb87 |
| Species | *Sphingomonas crusticola* | 98.99% | e8a242b0975f90535cd666cc0bbe7e78 |
| Species | *Sphingomonas cynarae* | 70.30% | 7ea8900eb5062ca2ee1690ee6ac15a82 |
| Species | *Sphingomonas faeni* | 99.67% | 04600fe81990d48f673efbc393ea135f |
| Species | *Sphingomonas faeni* | 99.67% | 449091f976fbf8f679e67afd43174f50 |
| Species | *Sphingomonas faeni* | 99.66% | 67a6371d31630b03d9c5c3ad420e4841 |
| Species | *Sphingomonas phyllosphaerae* | 97.87% | 3abb34a99979df470633462fc38694d8 |
| Species | *Sphingomonas phyllosphaerae* | 97.72% | cd1875f207326ed83ca759234dde89b3 |
| Species | *Sphingomonas phyllosphaerae* | 98.43% | 00c6cde5df4907ca46225655f2e1075f |
| Species | *Sphingomonas roseiflava* | 75.12% | f52e9a18bdc8987e249168639480af3f |
| Species | *Sphingomonas roseiflava* | 75.12% | ce7c2acacca5bbc4a4a2245660fc973d |
| Species | *Sphingomonas roseiflava* | 74.93% | 624aa4ef52bef2f2c7ea5f09f902ad8a |
| Species | *Sphingomonas roseiflava* | 73.45% | 4aa0a28ac99e10d30a478b66b31a9134 |
| Species | *Spirosoma oryzae* | 99.95% | 4389ddcec9797073c6c1423cba7fc274 |
| Species | *Spirosoma oryzae* | 99.99% | 1079a501a5d220f30fef25cf5763e548 |
| Species | *Spirosoma oryzae* | 99.95% | 61ac6c4a4af9c8db519b130a270cc2a8 |
| Species | *Spirosoma pomorum* | 95.57% | 0c02277564f701af9642b5a7c407bc07 |
| Species | *Spirosoma pomorum* | 99.91% | a231650d6b41c07fa9f06f37c0a9087a |
| Species | *Staphylococcus carnosus* | 99.99% | baf51b24d5f98baa3d7494e8e8ddd6e8 |
| Species | *Staphylococcus carnosus* | 100.00% | 9442d373256297a1ea403679d7343740 |
| Species | *Staphylococcus carnosus* | 99.99% | 900789544f80d18d0dd8bbd265d18e38 |
| Species | *Staphylococcus carnosus* | 100.00% | 2089418b0fab403c8c51fb991816dc2e |
| Species | *Staphylococcus carnosus* | 99.86% | 590d2a40eb1d8e5bed490c2dfe58fbd5 |
| Species | *Staphylococcus carnosus* | 99.89% | e0a6a6968342f743c34cb757358ce72e |
| Species | *Staphylococcus carnosus* | 100.00% | ed1536b39bfbe8f360e2be051ba75b46 |
| Species | *Staphylococcus carnosus* | 100.00% | 475da4e9d4013ffa6bc00aa0aa8ca003 |
| Species | *Staphylococcus carnosus* | 99.99% | 4c9a4701836f20d6a206a5d468001cfe |
| Species | *Streptococcus pneumoniae* | 100.00% | de01fa220eb44aa99898ddd3316631a6 |
| Species | *Streptococcus pneumoniae* | 100.00% | 9ccbb9bd79a98dea8392197f937b2337 |
| Species | *Streptococcus pneumoniae* | 100.00% | db395ccd0114ec25a2aafe6c05296ded |
| Species | *Streptomyces sp.* | 88.00% | 7a7e8519e6a1789d5d9bda760e81b4c5 |
| Species | *Streptomyces sp.* | 91.16% | b4b71e4b5d6c6f219adc364cd47968ff |
| Species | *Streptomyces sp.* | 72.37% | 52904d58b891dfcb8f2de8478cae239f |
| Species | *Streptomyces sp.* | 82.20% | ffd76d0625f3d3a07ab3f00a31b3427c |
| Species | *Streptomyces sp.* | 79.66% | 2745c98f3a68d28f2c7c03d6c6672834 |
| Species | *Streptomyces sp.* | 99.99% | b5a008dd5d0b336538d6b5283401694f |
| Species | *Streptomyces sp.* | 99.72% | a70fc8072077bf6a008747c2755344b2 |
| Species | *Streptomyces sp.* | 97.97% | d24f531f6b59cf8c1fb69115929d237c |
| Species | *Streptomyces sp.* | 70.34% | 5a2c4763f3487ab0344bb817e805838f |
| Species | *Streptomyces sp.* | 77.26% | c6359f6d8ccedd53bd79998ec06c9da8 |
| Species | *Streptomyces sp.* | 82.36% | ae185fc37080ced41f2a6e8ac78f5e29 |
| Species | *Streptomyces sp.* | 99.97% | 7e21e000b1e6699290b61d34ca912ea0 |
| Species | *Streptomyces sp.* | 99.80% | 0d41ba775d0e31aa686de3c804f84144 |
| Species | *Streptomyces sp.* | 98.01% | 398e3c2d6c892ea5998dde7c71b33578 |
| Species | *Streptomyces sp.* | 99.98% | b075302b85c69a2db8c812f5fc058766 |
| Species | *Streptomyces sp.* | 99.97% | 9ed18a499418e8df75a3d6d2f37085e2 |
| Species | *Streptomyces sp.* | 100.00% | 6189bd389031938493319ef6a0c39fd1 |
| Species | *Streptomyces sp.* | 99.94% | 4d572f5295efefc34e73a3d42c7c9879 |
| Species | *Streptomyces sp.* | 100.00% | 6104ba50eaa7a7ea770e94e6555e2038 |
| Species | *Streptomyces sp.* | 97.36% | 302005b7b652f9536d12a02666ca28f7 |
| Species | *Streptomyces sp.* | 98.11% | 1093e21c662b010634d45f4d5dbe90da |
| Species | *Streptomyces sp.* | 100.00% | 01ee2b460f22ad6c4ea8448f377caac0 |
| Species | *Streptomyces sp.* | 100.00% | ddc1971d97f78401e99b4ffebb8f2da1 |
| Species | *Streptomyces sp.* | 99.99% | 5ee8a99d3fa4ef8da82d1212de8e9aa1 |
| Species | *Streptomyces sp.* | 99.99% | 623aefac464bf0c6730b6e7daaeac8fb |
| Species | *Streptomyces sp.* | 98.66% | 3581b7e06f1b69953de42323915958e8 |
| Species | *Streptomyces sp.* | 100.00% | 3519b70afb36075fd5cd3a2f0bc926dc |
| Species | *Streptomyces sp.* | 100.00% | 69c9afbb4ce81fb05e0c8c9a8f0e700e |
| Species | *Streptomyces sp.* | 95.11% | 3be50c401e27f8ebabb1c69f942118b2 |
| Species | *Streptomyces sp.* | 100.00% | 3b4fb9f1fbd01a808b2f450668e9afe4 |
| Species | *Streptomyces sp.* | 82.13% | 92df125c9c5a0b5bdd7d82b3de554907 |
| Species | *Streptomyces sp.* | 100.00% | 448c80da073c195e6960c6ed9ef0a31d |
| Species | *Streptomyces sp.* | 88.85% | 6a5ce6969434213b1cd8a01145add9ee |
| Species | *Streptomyces sp.* | 95.94% | 76045d140c7152ba0614f117321e8f79 |
| Species | *Terriglobus aquaticus* | 99.75% | 532ba12d310477c4390fd23ff7a7b77c |
| Species | *Vagococcus fluvialis* | 74.94% | 9e30822c297122ea922d039d2f180801 |
| Species | *Vagococcus fluvialis* | 74.33% | ab411eed4d386ec6e941e9a0cdba49ef |
| Species | *Vagococcus fluvialis* | 75.79% | 5c7f9e8c96e69c06c948656063d05ad8 |
| Species | *Vagococcus fluvialis* | 75.79% | 4f6ac13400d5347e5796106f6eecaa6f |
| Species | *Vagococcus teuberi* | 98.57% | 99d5da78d681dd40043e21a5beec83ba |
| Species | *Vagococcus teuberi* | 98.57% | 2682ab4e9b11c65c84d48138645daa2d |
| Species | *Vagococcus teuberi* | 98.57% | f354e872116988230db53261cfc7bb0d |
| Species | *Vagococcus teuberi* | 95.66% | 774c5fa442ff8b5a66255e3c352cccbf |
| Species | *Vagococcus teuberi* | 98.60% | b92d24ae252f3ffa4add8bf1141d1898 |
| Species | *Vibrio penaeicida* | 86.16% | 0a13430bfb746ac289152603436bdfb1 |
| Species | *Wohlfahrtiimonas chitiniclastica* | 92.71% | 38ce0e996edb8432921a6dedaaf627c4 |
| Species | *Xanthomonas albilineans* | 93.92% | 89c46ac3879cf76f91312624e6917e9b |
