# Supplementary material for: Anthropogenic reverberations on the gut microbiome of dwarf chameleons (Bradypodion)
Source: PeerJ. 2025 Feb 28;13:e18811. doi: 10.7717/peerj.18811 (PMC11874949; doi:10.7717/peerj.18811)
Supplement: Supplemental Information 8 — Shown are taxonomic rank, taxonomic nomenclature of identification, the confidence of identification, and the unique sequence ASV identifier assigned. Any uncultured or unidentified classifications were assigned to the next highest taxonomic rank that was certain. [file peerj-13-18811-s008.docx]

| **Taxonomic rank** | **Taxon nomenclature** | **Confidence** | **Assigned sequence ID** |
| --- | --- | --- | --- |
| Phylum | Firmicutes | 99.92% | b44c1896f7bd720242dc0bb134cfe555 |
| Phylum | Firmicutes | 99.91% | 05780e154991c0a10200b432c9830a15 |
| Phylum | Firmicutes | 95.24% | 8516e81615e96e9fcfcf1486de4cc0cd |
| Phylum | Firmicutes | 100.00% | 1db1218ee8bb72939e4fae13ca320440 |
| Phylum | Firmicutes | 100.00% | c714af4fd335b38c68dd95e65abb3862 |
| Phylum | Firmicutes | 100.00% | e5fc21d85496a256809175f36acd8254 |
| Phylum | Firmicutes | 100.00% | 72068fdbc3e9f64dc2cbb85d8e8f6d49 |
| Phylum | Firmicutes | 99.13% | 27d6d74f706ab474af4261194d2a1440 |
| Phylum | Firmicutes | 100.00% | 5f8b6c5688c6742993d840ab30080879 |
| Phylum | Firmicutes | 100.00% | fc2427a7462c72fe15d74d121c8ed907 |
| Phylum | Rs-K70 termite group | 94.95% | f0520745dddeebc206683d952c0572c6 |
| Phylum | Rs-K70 termite group | 99.98% | 70dc4419b47f7794d5efdcb1428f3527 |
| Phylum | Rs-K70 termite group | 97.80% | 523e4f30880b3833631efa06e3359b36 |
| Phylum | Rs-K70 termite group | 99.98% | cba5c97cfbfeb660a1a749dd72add2ee |
| Phylum | Rs-K70 termite group | 99.42% | 961011767afa17ba32f8b3643e5e3101 |
| Phylum | Rs-K70 termite group | 99.97% | 82254aae85524bfae19febda0e87e2d4 |
| Class | Actinobacteria | 100.00% | b141d4b0b7e87828e88546ca214b8e1b |
| Class | Actinobacteria | 100.00% | bc6f865ddc2c684f3f45e5b63e2336c5 |
| Class | Actinobacteria | 100.00% | 705e4b74deb3b05549c0aff420064072 |
| Class | Actinobacteria | 100.00% | b3aa88cd9555300b40549b34278e0654 |
| Class | Actinobacteria | 100.00% | 61fd61fbbbe7de3c9a6c88ce63fa86ff |
| Class | Actinobacteria | 100.00% | d20a618f51168f562a318297921ec081 |
| Class | Actinobacteria | 100.00% | 62ab936556ecfcd716c2a2bcf97d483b |
| Class | Actinobacteria | 100.00% | 9b9741f20de351adb23113990ac09ea5 |
| Class | Actinobacteria | 100.00% | e53aa7856671f9356f7e0b8925ee933a |
| Class | Actinobacteria | 100.00% | 67a1dbc7e1e289b11dabb24280c63627 |
| Class | Actinobacteria | 100.00% | ed3ca8cddd8148d3a6be31386359d3b0 |
| Class | Actinobacteria | 100.00% | 5e3a294bf4dfd0f803953d93eb32ea41 |
| Class | Actinobacteria | 100.00% | c58e1674ad0983d941507ab49c71dacf |
| Class | Bacilli | 100.00% | 32c5be129a67498c94ff5080d0c95d40 |
| Class | Bacilli | 100.00% | 83533eccfab181c1f7021bf65250f215 |
| Class | Bacilli | 77.45% | e8b7f9af072ff5cb90fdfa6536b62ae8 |
| Class | Bacilli | 100.00% | f98b8f73ab22cb8e59c4bd76d1e34fcc |
| Class | Bacilli | 99.41% | fdf69aaed977dcd6c0e09bc6d937ab86 |
| Class | Bacilli | 99.54% | b8f5f059e26b896b7431d363b031a120 |
| Class | Bacilli | 99.98% | 0eb68cccf310b3c4250b55c13f45233c |
| Class | Bacilli | 85.62% | c37286940b677e44a37afe543a9d70b9 |
| Class | Bacilli | 99.99% | b9ca71a198375936e643e7084ff9f14f |
| Class | Bacilli | 99.72% | 4e3e33b42fa0b3bd9e1d7b35adda8993 |
| Class | Bacilli | 99.51% | 61b85a0a828031dbb6466a669b85f23a |
| Class | Bacilli | 91.04% | bedf32daa9bd41ff40d3a7d287d06082 |
| Class | Bacilli | 98.65% | 7d4671448b32d93586303eeb3bbdec92 |
| Class | Bacilli | 70.64% | 7799e803a5205fe28e99bb8a9103195e |
| Class | Clostridia | 100.00% | f686f364fd825bdcd915f0e9635a17a9 |
| Class | Clostridia | 100.00% | 93bb7022ab5d01e6ef5d6d62a03fc0d5 |
| Class | Clostridia | 99.99% | 58e5f8762a441e13d3406562b2398a66 |
| Class | Clostridia | 99.89% | 7e5688edfaf0cb5169127f7fa4ae408e |
| Class | Clostridia | 99.98% | 08e8581d79a4245445085647ff4e3268 |
| Class | Clostridia | 99.06% | 0c03c5a5be50c105e452e88529e68bda |
| Class | Clostridia | 99.95% | feed32ea15a38cf30a53baa64fb11f68 |
| Class | Clostridia | 99.99% | 7e5acecdb6ddcef7ccf0cc29dea9909e |
| Class | Clostridia | 99.87% | a2f0e8a67c2eb9f881de341777eceeb8 |
| Class | Clostridia | 99.02% | 94f558b0d1803dbad1a775d61f7e0d54 |
| Class | Clostridia | 99.99% | 5225303d8b9c8df6771b907868d21de0 |
| Class | Clostridia | 99.50% | fb2e3bb1351462181b5be8b6379d896f |
| Class | Clostridia | 99.77% | 82c4d0fbdf912d825cb48a8f8317ff5b |
| Class | Clostridia | 99.60% | 8ab14726b579179994e28c3d52bf8433 |
| Class | Clostridia | 99.68% | 8943030ad060bd6863d8a2c058bd6446 |
| Class | Clostridia | 99.60% | 7414c2686f7edea431a58849956d8fbb |
| Class | Clostridia | 99.98% | e7bcdcedce36fe0f84848237b373159e |
| Class | Clostridia | 99.59% | f4b1eaf2a8a26f16dd30ef64b8b87dde |
| Class | Clostridia | 86.75% | 784147ba7c5b86fe5ffe9a08d0fb56c5 |
| Class | Clostridia | 99.99% | bb5c7e49fee88c9dd11038ae98bde9b4 |
| Class | Clostridia | 99.91% | 2b6f6741da54dcfd08de6d13f996f0dc |
| Class | Clostridia | 99.71% | 7a14f6ca866f9c952c1336375485b9bb |
| Class | Clostridia | 99.95% | 335a5d07b90421020ffafb1dfbd8b862 |
| Class | Clostridia | 99.72% | e9c09226504c24e9e026282bda9bcfde |
| Class | Clostridia | 99.02% | c5e228aa91d35a24e909ba3cf62288ec |
| Class | Clostridia | 99.93% | 639246f297430bbb7d65e3e62fd55988 |
| Class | Clostridia | 99.99% | cffc11a9eedc82ca92f89434d5b9759a |
| Class | Clostridia | 99.49% | 3dda1ab3e2cbbd8fc5ff875aa763ff1b |
| Class | Clostridia | 99.97% | e88723d1edae717ac7076fb496d50cd2 |
| Class | Clostridia | 99.99% | ac7fbe95469d87257f0cf7f1c9810742 |
| Class | Clostridia | 99.17% | ec9d90351834090f5694366b1007fb33 |
| Class | Clostridia | 92.10% | 746ee38f4f04389be9f58ac0e760e907 |
| Class | Clostridia | 97.66% | 58397e10097f35ccd261b2c57b115edf |
| Class | Clostridia | 99.07% | 4da3dd283b7d9e460eb5c035924949ae |
| Class | Clostridia | 99.71% | 905114b5f8565cbecb339c58faa4a1d0 |
| Class | Clostridia | 93.54% | af9d6d4d2d741e9c222d3ebefe4c7274 |
| Class | Clostridia | 99.98% | dc43f115977c6e16c21919eb526f004f |
| Class | Clostridia | 91.40% | 1820734af728d4a5c6677814348f413d |
| Class | Clostridia | 99.98% | 2347adb8596b81fd84bc8ac856231105 |
| Class | Clostridia | 99.89% | bdd186e3023300b5b31f96ad89275da5 |
| Class | Clostridia | 99.99% | e530601b1400dbab5d6d4937b2038514 |
| Class | Clostridia | 97.66% | c3558353e83bb8f1485b706106861832 |
| Class | Clostridia | 99.34% | c39c37d451fa4beccc7255367d970e85 |
| Class | Clostridia | 99.97% | 95290a1cd2248e21cd74831911223d7a |
| Class | Clostridia | 99.95% | 811a7c5e6f5c6089a35e2aaba7c78375 |
| Class | Clostridia | 99.99% | 05702cd5373dbbf9af2b4f67131ad036 |
| Class | Clostridia | 99.96% | fe309b6e5b99f67c1033c3c87c517cc7 |
| Class | Clostridia | 93.25% | 39200ecd08263ec04f985d40b994debc |
| Class | Clostridia | 89.50% | 527d510a4286f62dc18b1da7174f1892 |
| Class | Clostridia | 99.99% | 94f5b0984a85ea58817f9bc5a9a4c84a |
| Class | Clostridia | 99.81% | abc31502c98ebf4b631884eb41f7a74f |
| Class | Clostridia | 99.51% | 4967e264feb3eabeec0c08e977fb16fd |
| Class | Clostridia | 99.63% | c2128c9463ed3b8c5283a1f103f882f8 |
| Class | Clostridia | 99.59% | 5db7647eab145f4b8d0d2b21ae449e8a |
| Class | Clostridia | 99.98% | 93b44861602a3dde61f167f2792f4cfe |
| Class | Clostridia | 100.00% | f2d8425c3fc55b321f4a012a36d844ec |
| Class | Clostridia | 99.61% | c3bff4a3e29c2edb0dc2a848a55c620b |
| Class | Clostridia | 99.87% | 26031a2c2a30cb65dda0507ab8a5491e |
| Class | Clostridia | 99.95% | 9754001b8c84399eaa4ac0df14408b35 |
| Class | Clostridia | 97.67% | 4e5e2c2672c169446eec22d78c164367 |
| Class | Clostridia | 73.24% | ffa284eacd160daf4271041dc79f8b4d |
| Class | Clostridia | 99.95% | 10a4ce578de044e15a78f188ccedb4f2 |
| Class | Clostridia | 98.83% | 503a3bef700d3758ecb170a0b1325bcb |
| Class | Clostridia | 100.00% | 17d88e48436be1677b480bf4e152f339 |
| Class | Clostridia | 99.96% | 56ebffa5b8e4fc59c7163c2c2188a27c |
| Class | Gammaproteobacteria | 99.81% | 34320d0ee62c70c703a1f0a6dc060e82 |
| Order | Bacillales | 98.55% | bbc8de89fb13074dcf19c7af1f0f5f9c |
| Order | Bacteroidales | 98.23% | cc36bffe204d2b6bec699f3381b432a6 |
| Order | Bacteroidales | 99.88% | 6e6cb8e8c9edd83227f3e9f754fb6b7d |
| Order | Bacteroidales | 99.75% | be17595d914191b85df7380ca2587bcc |
| Order | Bacteroidales | 99.40% | ff34501137f618a85617ef92b9f1f1f9 |
| Order | Bacteroidales | 99.17% | 978ecb88fe9bcd8996ec5f064728ed30 |
| Order | Bacteroidales | 96.90% | 6fe9fe2e5ced31894c512bcf8251350d |
| Order | Bacteroidales | 96.72% | 0ae0c5b4e8804024b9e3aa409305c52c |
| Order | Bacteroidales | 97.48% | 1de3191e5892e104191fd96c661eaf5e |
| Order | Bacteroidales | 97.37% | 717a1cef017136f79bde2c049b77f5a6 |
| Order | Bacteroidales | 97.88% | ad414534855e0de71429ec03fd49fdd0 |
| Order | Bacteroidales | 97.31% | 991d1bb2c39d6128378351d766ae1950 |
| Order | Bacteroidales | 98.14% | c77e16ddd75ded5324e52d350390b1f2 |
| Order | Bacteroidales | 99.18% | 3c8e82a9fb700306e8fb9d7bce6fe286 |
| Order | Bacteroidales | 96.90% | 8b24ee212fc2ff0410ef1d12fbb272d7 |
| Order | Bacteroidales | 99.18% | 996cf30c888c68652d588d65c390cae2 |
| Order | Bacteroidales | 99.98% | 44081508462ea0583762aa7006010b49 |
| Order | Bacteroidales | 96.14% | 4f051fe51c30f456014d79dcb721eb01 |
| Order | Bacteroidales | 99.95% | 8d22fb351627511f6694d6e9dc738e4d |
| Order | Bacteroidales | 98.06% | a8d7dd08cbdc7e2746a03a21f59405a2 |
| Order | Bacteroidales | 95.90% | 4b876f2c9d7a9094a30241198197d63e |
| Order | Bacteroidales | 99.96% | 1b5e41869c65fa5b95e774b1181b24a5 |
| Order | Bacteroidales | 95.74% | 5be6e49936430bd553c7ea454bc9e523 |
| Order | Bacteroidales | 99.99% | ce0da3fdf6bd46bc36f734aa79ae27d9 |
| Order | Bacteroidales | 100.00% | 63f5f36b8e3e7a3a3a999389ce3e8646 |
| Order | Bacteroidales | 99.95% | d112dbbcccc803ac775676b46d09a0c8 |
| Order | Bacteroidales | 100.00% | 2a5f738003eb0a35c77d63d25a1e4939 |
| Order | Bacteroidales | 99.96% | f736e25f412186d16a220c89eddcba43 |
| Order | Bacteroidales | 99.07% | 6496856e8dbf2c1a5e5ca1c5a2b55202 |
| Order | Bacteroidales | 97.35% | 3d8ee989b35d469e75f0d754b32ca42a |
| Order | Bacteroidales | 100.00% | fef491ba280d643af9feda732efbc274 |
| Order | Bacteroidales | 99.99% | 65fc108ec35e1a7517c952e48e660768 |
| Order | Bacteroidales | 99.99% | 112fa9446cf11e4d99941927febf1ff7 |
| Order | Bacteroidales | 96.32% | 6e20261412eac29f0ef8800280d0e84d |
| Order | Bacteroidales | 96.30% | 764a08bd9b265bd899e3326c0241724b |
| Order | Bacteroidales | 96.26% | afcfd5dd3f542123e064ec31cc9dfdb4 |
| Order | Bacteroidales | 99.59% | 001ee1089b5c14acaafe64721680bc9b |
| Order | Bacteroidales | 96.31% | 98940c7ab248f428179165dbd99a69bb |
| Order | Bacteroidales | 100.00% | bc3d322dd65ce51a657a6d2d9548a135 |
| Order | Bacteroidales | 90.96% | aff36eeca719d21c3c6e8a59422ac621 |
| Order | Bacteroidales | 99.99% | 924e34c382f0dc899e465841603b2c30 |
| Order | Bacteroidales | 99.88% | a9310f146fca1d3c13851d39a7737a3b |
| Order | Bacteroidales | 99.84% | 7c325e3256258d2da63a908744c75001 |
| Order | Bacteroidales | 99.99% | 61121071ee39dfb1119ef04e23e6194b |
| Order | Bacteroidales | 100.00% | e9ccd941251f402d717791a800e378c1 |
| Order | Bacteroidales | 99.98% | 7d521475238af356fcb6e4149ce86997 |
| Order | Bacteroidales | 96.72% | 31b1f863875580eef0edf6ca7239065c |
| Order | Bacteroidales | 99.98% | 85e4a77163420d2352c4cc934580b67a |
| Order | Bacteroidales | 99.98% | 8839786e92344760ef6f134bb675b3b8 |
| Order | Bacteroidales | 99.75% | 8f7c1e4082854cddd9bfe877137d07a9 |
| Order | Bacteroidales | 84.68% | 519f8a3015f848b9ebbb3ddace243499 |
| Order | Bacteroidales | 98.62% | 0846f6283bf25477473a7c7d4856e683 |
| Order | Bacteroidales | 99.89% | d1d34e413f014abb2919f7e665ec0beb |
| Order | Bacteroidales | 95.92% | 8718f229424f05f6aad2895c3a1b3933 |
| Order | Bacteroidales | 70.18% | 4721e1ae40ac3a07f84437a31e83af69 |
| Order | Bacteroidales | 84.34% | d9063c38119f041b0c36b3d58e0251a6 |
| Order | Bacteroidales | 99.94% | 99c449d5db5a4de4ad872ecc0aa08c83 |
| Order | Bacteroidales | 85.33% | 065b8a6603ef2eff41ee9413aff8542d |
| Order | Bacteroidales | 98.24% | 4f36c070b2e3d2b38372ff1377e29bca |
| Order | Bacteroidales | 99.32% | c3be4c3228ec7228a5281154a9957acd |
| Order | Bacteroidales | 99.43% | d9a87f8f97efe56e04691ea041e24785 |
| Order | Bacteroidales | 98.75% | 6a92ee2dfebe10074603745fa5e91064 |
| Order | Burkholderiales | 99.27% | df4818cb346084d53c3ac16fc7cefb44 |
| Order | Burkholderiales | 99.98% | 862d6ee9e00d3e7a718a411f2f2e235b |
| Order | Burkholderiales | 99.85% | cb0b006fe6959fdd9154ffe1f222be3c |
| Order | Burkholderiales | 98.68% | 38363cd320308a9acce6f213b36288a4 |
| Order | Clostridia UCG-014 | 70.06% | 6279d90cb2d2f5dfb30694465f6599f2 |
| Order | Clostridia UCG-014 | 89.78% | d7c98ae8ef0e3469aced6fa2617e0056 |
| Order | Clostridia UCG-014 | 91.56% | b67dcf9a6f701b0765f1e833cecbb36a |
| Order | Clostridia UCG-014 | 74.82% | 1f97b1d7aa782cd766b3d8732ec65603 |
| Order | Clostridia UCG-014 | 77.49% | 3280e8c9c76910dd4f0ed01ac7ecaffb |
| Order | Clostridia vadinBB60 group | 70.59% | 60bffc2c54baa45da5fd06dd31532a2c |
| Order | Clostridia vadinBB60 group | 84.84% | b67b8c94c3c5b881973526b886ce9e1b |
| Order | Clostridia vadinBB60 group | 99.48% | 61271479bc81cbdf3cd457dcbe177676 |
| Order | Clostridia vadinBB60 group | 100.00% | d82f72c464db5d2b2a9ace65d89c4dc5 |
| Order | Clostridia vadinBB60 group | 79.80% | e8ebf93e8ba1c77a8ba51634f5ced35f |
| Order | Clostridia vadinBB60 group | 79.37% | 0b3576e2713bd19a788f82937d0c87f7 |
| Order | Clostridia vadinBB60 group | 73.86% | 4d44229a8b4145b75681176f2cd79539 |
| Order | Clostridiales | 89.87% | 2ff07c11530311c674951f431906381b |
| Order | Clostridiales | 86.04% | 081f12992154815c88d4a370bc0120c4 |
| Order | Corynebacteriales | 100.00% | d490c9056fc5498ab51375d9ab2ff3ac |
| Order | Desulfobacterales | 99.96% | b78776191246946108b108e81f157ad1 |
| Order | Desulfobacterales | 100.00% | 74650c62a517900c92344b74f1d7fb14 |
| Order | Desulfobacterales | 100.00% | df5a8dc26b178d63c4c678c9592cfa7f |
| Order | Desulfobacterales | 99.98% | e14f15dbda88282cca8f62fbae1df233 |
| Order | Desulfobacterales | 100.00% | e4ee9ddcbcca194ce0654a91ef697d39 |
| Order | Enterobacterales | 99.97% | c3291eac944f22837e3f6dcf0a79e7a1 |
| Order | Enterobacterales | 99.97% | c94883555b0e8fff5aa2ff2709074cbf |
| Order | Enterobacterales | 100.00% | 19f043e9a4ef6f6174416ae33430f1d9 |
| Order | Enterobacterales | 100.00% | a32f0c9e5acb2d9909e7a8f1110879bf |
| Order | Enterobacterales | 99.21% | 7cea45d84d19dbf5bd9129f6af28ecb3 |
| Order | Enterobacterales | 99.92% | d320599b2763c699710c2217fdc5c6f5 |
| Order | Enterobacterales | 99.97% | 923ab40968bdf12ce6beb6dd2aa32747 |
| Order | Enterobacterales | 99.98% | 6c1a63b79fa2708a21aa01f02a3a21e1 |
| Order | Enterobacterales | 100.00% | 87887a45b937e44afda07652c0d7d6ca |
| Order | Lactobacillales | 99.83% | f53ff14fef90e36daaa2024a85babc2f |
| Order | Lactobacillales | 71.77% | 78105b388e596466c1179e3e8d737797 |
| Order | Lactobacillales | 98.75% | db8ac32cf15394041a7e2d7c62fe15c3 |
| Order | Lactobacillales | 98.04% | 2c0816bfde5fc69d0e22f650394eafb2 |
| Order | Micrococcales | 91.28% | 0d1c40324b3afc21470c10ae9a395b32 |
| Order | Micrococcales | 92.55% | 2d956ce1261e44b0cc0adc38a703b93b |
| Order | Micrococcales | 89.95% | 9ca7ec7174c278f84a5f09c173c58858 |
| Order | Oscillospirales | 98.55% | dc9729fadd329a7dcfcda6712faa99fc |
| Order | Oscillospirales | 92.74% | eb1bd96970c61b59cdfbdf176eaf623c |
| Order | Oscillospirales | 88.16% | 6b29a009d85862652324cef5ac1d71df |
| Order | Oscillospirales | 98.74% | c1d78a54333879864c2992c267beb894 |
| Order | Oscillospirales | 93.16% | ee85b1617f059326a7a3f63bb8f9025a |
| Order | Oscillospirales | 99.98% | 18ca1f39d0996f2145fc0c99ffbe96d7 |
| Order | Oscillospirales | 96.96% | 265fae9a982af93c6dd284dac967ebd7 |
| Order | Oscillospirales | 99.98% | b6b2b00abfcb305076cf0c5ec1e2965b |
| Order | Oscillospirales | 99.98% | 52cb34b8fbd35cff30750c0d83a5507d |
| Order | Oscillospirales | 99.99% | 3f80e28fd50750baff8b65b0cce01e26 |
| Order | Oscillospirales | 96.53% | 34b7ca94a7049fc264373b06fbd71120 |
| Order | Oscillospirales | 100.00% | 84e201dfd1c6dafe210ae4fabe3f8261 |
| Order | Oscillospirales | 100.00% | ad950c4640710d8095cbf77bad05c417 |
| Order | Oscillospirales | 99.99% | 6d505420c0156b288b4ab7224b2cfca5 |
| Order | Oscillospirales | 99.98% | 9cc4c4f184fd4c19e45477eb274ee1ad |
| Order | Oscillospirales | 99.95% | 843676441fe434157f8226baff1282aa |
| Order | Oscillospirales | 99.98% | 91b37eca4194e0c8a006167c11acc22d |
| Order | Oscillospirales | 100.00% | 8af44dc00cf9bcef52d6aad3d331e5cc |
| Order | Oscillospirales | 99.26% | a0963d9c4c2d45bdeadefc768daf64cf |
| Order | Oscillospirales | 99.99% | 07aabe1a4a7f87c3c3110bdd515c0bd8 |
| Order | Oscillospirales | 99.92% | 28fe318da5eec639359798a1afa5ca64 |
| Order | Oscillospirales | 99.98% | 7a7761f270e4c72aa81a5e9c06745e64 |
| Order | Oscillospirales | 99.98% | 8ca4ca375376c9ff606b46cbb3757ded |
| Order | Oscillospirales | 99.99% | 02d17d778b1eddc722b7691ab7655984 |
| Order | Oscillospirales | 99.97% | 5941272332e48ec7c3435ae47cc67666 |
| Order | Oscillospirales | 99.94% | c19d5b8798ad9b746abe497d542db1f5 |
| Order | Oscillospirales | 99.96% | ab2bcf34cb85f46bdcefd3eff839ae3f |
| Order | Oscillospirales | 99.17% | c3106aea6861c9860fc0a32ffde8531f |
| Order | Oscillospirales | 100.00% | f5a261470eb1b192952e3f40121ee277 |
| Order | Oscillospirales | 99.45% | 54345c0e27a68763825a8a0153d0a626 |
| Order | Oscillospirales | 99.81% | c72c3423060c46bb11c5eea265cbdcc5 |
| Order | Oscillospirales | 100.00% | a8ff3b616d574b6bff8c2939add8aff2 |
| Order | Oscillospirales | 99.86% | 13ebf0a852a8d5081d86601e54d1f9b8 |
| Order | Oscillospirales | 99.85% | 5e3c3cd908f789babc8841d799e4ebe2 |
| Order | Oscillospirales | 99.92% | 7d731f1dc29d76596d4ec52a0a419350 |
| Order | Oscillospirales | 99.98% | 198f5ede0084932df126c791f08c110d |
| Order | Oscillospirales | 99.74% | 83ff2b2f2d52b09e8811507583e46002 |
| Order | Oscillospirales | 99.99% | a044d3fd00f997288d8be94d43f48d10 |
| Order | Oscillospirales | 100.00% | f7d00b5160a638d53455bb3fdd60f2b3 |
| Order | Oscillospirales | 99.96% | 74a758ed69a686ea2a0873ad3a1b984d |
| Order | Oscillospirales | 86.63% | c038cb7d0bb6f60c96d33e47f2c2826d |
| Order | Oscillospirales | 76.38% | 9daecb72180b770214e9d0cdab679783 |
| Order | Oscillospirales | 88.74% | 184b884c1c91d964de258ac9ce0166bd |
| Order | Oscillospirales | 100.00% | dbecad8a1b18819826620f1ab8f07ef3 |
| Order | Oscillospirales | 87.74% | 90eedd246b88a60e7b782c422d474caf |
| Order | Oscillospirales | 98.85% | c63c0431805f3edc06bec8090af84db0 |
| Order | Peptostreptococcales-Tissierellales | 99.86% | 40e830654e545a94a017468cafa1afd4 |
| Order | Peptostreptococcales-Tissierellales | 99.98% | 0d0b4b22748e2849430a5adf47fff9e3 |
| Order | Peptostreptococcales-Tissierellales | 99.96% | eafa1b73b89f2b4ab9dec1c8a2cea247 |
| Order | Peptostreptococcales-Tissierellales | 99.85% | 523fa6889151500383d8853edb8256bd |
| Order | Peptostreptococcales-Tissierellales | 99.88% | bef7baed9ba700f78a1f11da83652e20 |
| Order | Peptostreptococcales-Tissierellales | 99.87% | f80e42433d792e2d458febe99e36b452 |
| Order | Peptostreptococcales-Tissierellales | 99.87% | d7438fffe7f6528496933bc8af768a93 |
| Order | Peptostreptococcales-Tissierellales | 99.96% | 82af4c26eb8361df82000e31ccc0e428 |
| Order | Peptostreptococcales-Tissierellales | 99.90% | 7d992e70be82e5ff2f5fbb75b3ede9f1 |
| Order | Peptostreptococcales-Tissierellales | 99.84% | 394f3128aae312c182e342fd59ee5355 |
| Order | Peptostreptococcales-Tissierellales | 99.98% | 27203324b5400ab9e1f7813b6dbaf0ae |
| Order | Peptostreptococcales-Tissierellales | 99.49% | 884403d9a31ae5354766411656c39cca |
| Order | Peptostreptococcales-Tissierellales | 99.88% | d8b7da502692beb0812baeec5bd3f31c |
| Order | Rhodospirillales | 99.99% | 02d7dd696c037e4179a1c1fce76acd3a |
| Order | Rhodospirillales | 100.00% | ff14d4b22c93772542e336f10db151a3 |
| Order | Rhodospirillales | 92.56% | 0bac4c29cf5231deddbe78695ceeebef |
| Order | Rhodospirillales | 99.90% | 2a64660d49ae67c4e721d306703de838 |
| Order | Veillonellales-Selenomonadales | 99.95% | e628ca83e9b5ce41a37de71d9856e195 |
| Order | Veillonellales-Selenomonadales | 100.00% | d4131c0ce02d3403f7f32768ff7d180e |
| Order | Veillonellales-Selenomonadales | 99.94% | 203aa43219c3251b4321eb20ff14b57d |
| Order | Veillonellales-Selenomonadales | 100.00% | 516c1cf21455ace1aa66717b28298602 |
| Order | Veillonellales-Selenomonadales | 99.99% | b52dee5cc5646110f2a4a0add3f81109 |
| Order | Veillonellales-Selenomonadales | 99.99% | 639355caab89dfb462ab9ae71564923d |
| Order | Veillonellales-Selenomonadales | 99.99% | cc203b329f1d32b65ce6fcd0595efa6c |
| Order | Veillonellales-Selenomonadales | 98.19% | 2c578abc93a37db8d7b8d704503c5789 |
| Order | Veillonellales-Selenomonadales | 98.20% | c8ee0ec6ce4842549d5831462a13a067 |
| Order | Veillonellales-Selenomonadales | 99.99% | 641d60235b3428de1f171acc0470d7d1 |
| Order | Victivallales | 99.99% | c48e088000f3298fc6dd4c89172f5cfa |
| Order | Victivallales | 100.00% | eb1d578bdc18cccc3bcf8b75f04d21b0 |
| Order | Victivallales | 99.99% | 2839ed97ab61d7945e2e5441721b28a1 |
| Order | Victivallales | 100.00% | 4669973deed034782542a2dbfb997a70 |
| Order | Victivallales | 99.99% | 4e35d2f1c85ae19ef9269b80769866a7 |
| Family | Acetobacteraceae | 100.00% | e5d053249f519c2f1affcd2cea9745b4 |
| Family | Acetobacteraceae | 99.99% | 10fd01ff4e07913e4836449bf8ef6b57 |
| Family | Acidaminococcaceae | 98.81% | ec3c45b7806b26e579b2b732a3bcdff7 |
| Family | Acidaminococcaceae | 87.21% | e945004bf5c08832123c96f909ad5728 |
| Family | Acidaminococcaceae | 98.50% | 587a87d886fa29c3fe0a5fff5b71d8cd |
| Family | Acidaminococcaceae | 98.26% | b918008216e4721d8af66dc0d11febaf |
| Family | Acidaminococcaceae | 94.56% | c543c5ee67db506274f4a56d08962c6f |
| Family | Acidaminococcaceae | 94.05% | c226192ad2bef3b2b2bd10763db9e15d |
| Family | Acidaminococcaceae | 94.59% | f6c27d8afd8ef8bf39cb44f9eda658ad |
| Family | Anaerovoracaceae | 82.42% | 5c10c9c7fba4ae2a60037ae903a95ac9 |
| Family | Anaerovoracaceae | 99.10% | 6ccba58f87f3ceff9b55d25e77e62225 |
| Family | Anaerovoracaceae | 99.97% | 8cfc9829da13648f3f247a7e8820e66c |
| Family | Anaerovoracaceae | 74.38% | 5f7e9423ed0a6e932857616b5f1027ee |
| Family | Anaerovoracaceae | 80.94% | 195a5fe3ebb65da89885b8f69ee0d38b |
| Family | Anaerovoracaceae | 82.74% | 56e84f0aa40141072af133cfc94a219c |
| Family | Anaerovoracaceae | 80.98% | 2b53ed02dda2edd40d5b82975b8a6d69 |
| Family | Anaerovoracaceae | 99.48% | 809ab7d6bc79007885afe48a56b214f3 |
| Family | Anaerovoracaceae | 99.91% | 6041bf73ad7a6b65dca9771b5f1e61ee |
| Family | Anaerovoracaceae | 76.14% | 6a338d20b57d1d0942a1240375fce3b3 |
| Family | Anaerovoracaceae | 86.74% | 8007789dec7adbbeee5d7b9cdac763f3 |
| Family | Anaerovoracaceae | 80.96% | a24c32df85148f051e46347d61aef90f |
| Family | Anaerovoracaceae | 83.26% | 2350a40412d4e80d2b0ed95dbc576cdb |
| Family | Anaerovoracaceae | 80.89% | c6f3e359a628f61e66db444c541316d3 |
| Family | Anaerovoracaceae | 99.92% | c576974d4bd86935f68ecafa1d0c4cfd |
| Family | Anaerovoracaceae | 70.30% | 0e6e3091acdb8b6db107f972c5e08b89 |
| Family | Anaerovoracaceae | 77.75% | 65d6f8a7ced51d43de5d74dfd7c1cab1 |
| Family | Anaerovoracaceae | 80.57% | aa06fda15099c34b5b8999f774d58297 |
| Family | Anaerovoracaceae | 98.36% | f82b9e00a06b3ee83324e4eb77e97399 |
| Family | Anaerovoracaceae | 86.86% | 1bcd365a622444a52713dec52ec5cfe6 |
| Family | Anaerovoracaceae | 86.82% | f900af81e05016a9896dc66bf6fae92f |
| Family | Anaerovoracaceae | 86.89% | 8b37029eb3247b1362eee9428a78ac0e |
| Family | Anaerovoracaceae | 75.85% | 8fb86ea201a9cdeb3ff0c31a76815a22 |
| Family | Anaerovoracaceae | 85.93% | eb1051825c705653f5d522d16767a665 |
| Family | Anaerovoracaceae | 78.31% | 0c679207d91096d91d2917dd57a3117f |
| Family | Anaerovoracaceae | 82.04% | a6a7af1c9039429f3f63eb6a705433a0 |
| Family | Anaerovoracaceae | 72.70% | c8991723a8645d0b3c94e8c180361530 |
| Family | Anaerovoracaceae | 85.74% | e85a8c5368835e512a77868670ef6640 |
| Family | Anaerovoracaceae | 77.47% | d075b50974b304e78e95f7db8c2d084b |
| Family | Anaerovoracaceae | 91.89% | 25a5daa0071bda6c66fecf2aa5a90179 |
| Family | Anaerovoracaceae | 95.14% | e55357d85646731569d30e5d816beae2 |
| Family | Anaerovoracaceae | 98.94% | ac038088fd3d5dba7d4ae06331ee3045 |
| Family | Anaerovoracaceae | 98.75% | a2553df551d36e46a764c07e5ec2fd54 |
| Family | Anaerovoracaceae | 80.08% | 9595589bbfffa86740a44d16613d0b0f |
| Family | Anaerovoracaceae | 98.76% | 52a1310bbc7f2472a5c48b901dfe8565 |
| Family | Anaerovoracaceae | 98.76% | 670fcb70026b3a60c7346fa69b8d9233 |
| Family | Anaerovoracaceae | 70.30% | a0ba9dd8a41be506db087a0e0c318773 |
| Family | Atopobiaceae | 100.00% | 60f0dcc9ed64dcfad765840002cc3b6e |
| Family | Bacillaceae | 92.70% | 47d3453e35352eae05d84294b1a1f6fe |
| Family | Bacillaceae | 79.23% | 5e6e823f56d1361b77e99f76fa3640dd |
| Family | Beijerinckiaceae | 88.40% | f982312e3c51f3f017a10bba68feb99c |
| Family | Beijerinckiaceae | 88.23% | 56267169b1b006c83527536b30fb38a9 |
| Family | Beijerinckiaceae | 99.51% | f4370396b9f4b71d20a50ecfd11a48e2 |
| Family | Beijerinckiaceae | 99.59% | 0acffd86c4af93cb44bccc642db1b983 |
| Family | Beijerinckiaceae | 99.67% | 411a621aadb54ac2c683b660ae8c2d1a |
| Family | Beijerinckiaceae | 87.99% | a8ec806263c4707bacb4a7e327a186de |
| Family | Burkholderiaceae | 95.75% | 15d543c3d76d9527471247dfd42201ae |
| Family | Butyricicoccaceae | 99.95% | 396827fe87fe85985122efba3913395f |
| Family | Butyricicoccaceae | 99.96% | a5f202f133d49bd0d81f899e5371457d |
| Family | Butyricicoccaceae | 99.96% | d342b9038f7a682a0a1bb748d65bfcf4 |
| Family | Butyricicoccaceae | 99.95% | 8e925d8c26c5c590a605594bf39831ff |
| Family | Butyricicoccaceae | 99.95% | b00d2b2e4d3b32fda70766329a56b76e |
| Family | Butyricicoccaceae | 99.96% | c70a6bb2ac0ee4f44a1b9107bc790cd9 |
| Family | Butyricicoccaceae | 99.96% | 2415213178bd06b8ecebfa5f4ace8af2 |
| Family | Butyricicoccaceae | 99.95% | 6da441be59ab1a84a410b5c2d7591e1d |
| Family | Butyricicoccaceae | 99.97% | 9a021798961c6db6feb0f69d83a30058 |
| Family | Butyricicoccaceae | 71.19% | 2a18db737fbeb3001bf2057cb355231c |
| Family | Butyricicoccaceae | 96.21% | 41a55fad4b279aa612c2f9f8d4d028f7 |
| Family | Butyricicoccaceae | 78.75% | a0227ab8b27ca06b285ffe1db5936095 |
| Family | Butyricicoccaceae | 88.88% | 0e128a09dff98c3d8788f511cf8e4ceb |
| Family | Butyricicoccaceae | 86.37% | 4fd9acf4dff1852646d793c8579e9b0b |
| Family | Butyricicoccaceae | 87.18% | 43903aa6a97991b3b74280939e4b4997 |
| Family | Butyricicoccaceae | 70.63% | 243c501f8f88200ac3b76c6635f46d19 |
| Family | Butyricicoccaceae | 74.43% | 20905907adebfc4209f830e42cf1419a |
| Family | Christensenellaceae | 100.00% | 210dc71b04b46b6421f07b901d5513ed |
| Family | Christensenellaceae | 90.27% | 82e60ec5033fccf53f66cd3a810e2a14 |
| Family | Christensenellaceae | 100.00% | 987a5dddca87c0b0d9d40bb8f6550e8f |
| Family | Christensenellaceae | 100.00% | 044858b8d9eefcf122728cd3d99a00d5 |
| Family | Christensenellaceae | 74.58% | 7bf9d5d86a4296a0020a1bf6126befc9 |
| Family | Clostridiaceae | 99.93% | 2db3d1de42380b2895cb3100aca57770 |
| Family | Clostridiaceae | 99.98% | 3bd1e269fac8b1d43ca729c91d21cdfa |
| Family | Clostridiaceae | 99.98% | 4f9ddab4a98f19f737088418e88c4283 |
| Family | Clostridiaceae | 100.00% | afb44b560ff1a5168077e5a46316551e |
| Family | Clostridiaceae | 72.90% | 7a626c56b5f3768094cb02df9329caa6 |
| Family | Clostridiaceae | 100.00% | dd0418741f628365e9590f9ff9b00960 |
| Family | Clostridiaceae | 92.56% | a8cbe86a11f5a61bfdb521f74b258d81 |
| Family | Clostridiaceae | 99.60% | cc0e6cacb38711d87547bdd5bb4a00e2 |
| Family | Clostridiaceae | 75.87% | 17e6f399246734e3d44aff5b35e063b8 |
| Family | Clostridiaceae | 99.95% | 7225ac99d8c259f073da135d9595ffd3 |
| Family | Clostridiaceae | 99.60% | e1db9c50933c82c85f663ffa72e0a610 |
| Family | Clostridiaceae | 99.64% | b7c1912e0025c01e88821780dbd696b9 |
| Family | Clostridiaceae | 99.60% | 44f48c04bee185b03ae747c1fc9eb3ee |
| Family | Clostridiaceae | 99.66% | c39860f72a385e1c233e047886cbe109 |
| Family | Clostridiaceae | 99.99% | a47c64dd25c3b068a7297e5030c8f55c |
| Family | Clostridiaceae | 99.99% | 109298ed55986b502307325360f50a07 |
| Family | Clostridiaceae | 72.91% | 507eae55aac11df7d5ebbcb5d7973ea1 |
| Family | Clostridiaceae | 73.07% | 9085f379e1a68484a12e35b24a504d63 |
| Family | Clostridiaceae | 75.83% | a55135b7c06ba5b2931a708a650742c0 |
| Family | Clostridiaceae | 72.51% | 7bad531fc273bef0b9065f6fd8179360 |
| Family | Clostridiaceae | 73.95% | ea5a9ce13a488fdff77b5a53ed863005 |
| Family | Clostridiaceae | 73.88% | c91da8582781583ed28a26a56aeb6fdb |
| Family | Clostridiaceae | 98.23% | 9ca13f8be7712d1f68c1a31836e3ca0a |
| Family | Clostridiaceae | 72.06% | 76bc9b71b015bab5944598efc163292e |
| Family | Clostridiaceae | 74.41% | 835da531c94e2c883ebd7b989a4a8f2e |
| Family | Clostridiaceae | 96.26% | cc5da2c44e67af8d792b2a59ee7408b2 |
| Family | Clostridiaceae | 96.26% | ecb2d234e4985fd772ca8dbfc526ad75 |
| Family | Clostridiaceae | 93.99% | 1b8f27dc29a72cf1915aa1101486d450 |
| Family | Clostridiaceae | 90.26% | 3d21c9ca09947f11593a403b66b29da3 |
| Family | Clostridiaceae | 94.06% | 2018f8b89d0634e6312f52dfe92c1307 |
| Family | Clostridiaceae | 94.00% | 61d370be1bd54ecc9b39c842c60ad99a |
| Family | Clostridiaceae | 96.26% | 2ff2b05d9f01cb360e9bdebc2279e42d |
| Family | Clostridiaceae | 95.25% | b88684ea028b5fcc4004ea8a0834dffa |
| Family | Clostridiaceae | 90.16% | ba3a4e08471a567c30b34ad9599ac1a1 |
| Family | Clostridiaceae | 94.86% | 1c6a86a7b1ddaaf1d448b33cc5c8b7c7 |
| Family | Clostridiaceae | 99.98% | b6c08f29d3654dc513741dd2aae6c245 |
| Family | Clostridiaceae | 81.93% | b1da40608ae5504a09bcdba2f70cfaf7 |
| Family | Clostridiaceae | 87.87% | b63a9dd7fa2aa65bbe79f23a76942ba1 |
| Family | COB P4-1 termite group | 99.92% | fd5098376260348766eaf54754134021 |
| Family | Coriobacteriales Incertae Sedis | 99.99% | 0fd04b3d03e3005552371fc59922a63a |
| Family | Desulfovibrionaceae | 99.96% | 017a2bbfc7ee439bbd36c500bc014411 |
| Family | Desulfovibrionaceae | 99.95% | ef20787f144854b2b6864b277ae02ac4 |
| Family | Desulfovibrionaceae | 99.69% | b05236ad606ae30a4637c4a78fff8cfa |
| Family | Desulfovibrionaceae | 88.82% | 693af67b528b1363fb3e440715f2b092 |
| Family | Desulfovibrionaceae | 88.89% | 277d61e81710fc1e60f99330514ae5bc |
| Family | Desulfovibrionaceae | 94.67% | 9f1cce910b35608a4a60486e00593ca5 |
| Family | Desulfovibrionaceae | 99.07% | 72c78378af3fd5000d4d7cc77e1199ed |
| Family | Desulfovibrionaceae | 96.71% | c893e0b629577d77b5892942979ea80d |
| Family | Desulfovibrionaceae | 99.16% | e1730501db09e11557505bf34f0ec856 |
| Family | Desulfovibrionaceae | 99.63% | 90c7ad79bc647a41f71d400ff6b3a17b |
| Family | Desulfovibrionaceae | 99.69% | 916148a7d27f66c7fb173bd4781fb4ff |
| Family | Dysgonomonadaceae | 77.20% | 1801eeb5e5b51f925728d2e8c4fa7879 |
| Family | Eggerthellaceae | 95.61% | ffb6aad975b10875cba95483976af687 |
| Family | Enterobacteriaceae | 97.73% | 0c2c4e040dd7476325f379e8736be99d |
| Family | Enterobacteriaceae | 96.60% | d5c2b98c0dbca45148f5d67f43f91f84 |
| Family | Enterobacteriaceae | 81.20% | 245d790f9ae7188e003d094667516564 |
| Family | Enterobacteriaceae | 98.06% | 5dfdd682a39026d5babfa021117d2d03 |
| Family | Enterobacteriaceae | 80.22% | 3b50ef3b1dfaf341975e08b74080d98a |
| Family | Enterobacteriaceae | 96.33% | 401473eafdaa7e9af3917d629c723105 |
| Family | Enterobacteriaceae | 95.76% | 2b0d4c75571c5747bf46fe81b779340b |
| Family | Erysipelatoclostridiaceae | 100.00% | 38b89e24fa8abfbdc5e68beadca34989 |
| Family | Erysipelatoclostridiaceae | 100.00% | 2be5519f6dab487ba61217c8804d19b0 |
| Family | Erysipelatoclostridiaceae | 100.00% | 42175048641deb8d28e327462a891e04 |
| Family | Erysipelatoclostridiaceae | 100.00% | 1c32cd264b5aea2927ee0f3716c9eb50 |
| Family | Erysipelatoclostridiaceae | 100.00% | 6272f57705c1d0093e5b7872b38c776e |
| Family | Erysipelatoclostridiaceae | 100.00% | 23743cd07933144bac3a0c5ab38bc587 |
| Family | Erysipelatoclostridiaceae | 100.00% | d149366ad1fbdef476a01e9a29a18108 |
| Family | Erysipelatoclostridiaceae | 100.00% | ed178b1160055a4a08016b0c734df564 |
| Family | Erysipelatoclostridiaceae | 100.00% | cbb7ef1b8c04ebc09f9b61596641294d |
| Family | Erysipelatoclostridiaceae | 100.00% | c06088451a57d159eba7266b57f62f1c |
| Family | Erysipelatoclostridiaceae | 100.00% | 9b82beaa592a44a24e5c7071cf9e6cbb |
| Family | Erysipelatoclostridiaceae | 100.00% | fab25453dc6601280fdc375187983739 |
| Family | Erysipelatoclostridiaceae | 96.74% | c3367383451fe69972b0bb445efe0c9c |
| Family | Erysipelatoclostridiaceae | 99.81% | 919e992549378eeab954fef2fdb33bd5 |
| Family | Erysipelatoclostridiaceae | 100.00% | f304620462108129e3a605ed7938187f |
| Family | Erysipelatoclostridiaceae | 100.00% | 35746af9604a785fa85230ecc403d5ce |
| Family | Erysipelatoclostridiaceae | 100.00% | ef0baa610811fecd30e082a80a9036f3 |
| Family | Erysipelotrichaceae | 99.85% | 5182e06f63f39768d5bd59b9dd4740e5 |
| Family | Erysipelotrichaceae | 99.96% | 2777edcb8520e0c443400c8662cfa30a |
| Family | Erysipelotrichaceae | 99.84% | b01dd90d2f7aa365b0ab66c5c52604d6 |
| Family | Erysipelotrichaceae | 99.54% | 454968bc9675a77400553a01f288b1e9 |
| Family | Erysipelotrichaceae | 99.84% | 7597d0ab2cadf1d410f7be205e5b5f2b |
| Family | Erysipelotrichaceae | 99.94% | b417a01d8abf24f1ecbb47e99c1558ac |
| Family | Erysipelotrichaceae | 99.94% | 9530c05a348d41f5d22007d3323044ff |
| Family | Erysipelotrichaceae | 99.75% | e9e5b1d34f736d48ff99a52087ddb0ee |
| Family | Erysipelotrichaceae | 99.81% | 7e4514473e7b3e2786d89598b974a5fd |
| Family | Erysipelotrichaceae | 96.97% | 592519ca4c75a64ca38181a8b84f4056 |
| Family | Erysipelotrichaceae | 99.95% | ff10655ad68218036b2d06d789c440c3 |
| Family | Erysipelotrichaceae | 99.61% | ac9890d9e3adcfe4252f1c71dd5773a8 |
| Family | Erysipelotrichaceae | 99.97% | 4cd5c51a3b1b30941104f30bf9f1ca76 |
| Family | Erysipelotrichaceae | 99.85% | 5c8878040fb628427a9f2738965caf71 |
| Family | Erysipelotrichaceae | 99.94% | 08ad60e55d6a6e43b887727d9a89c601 |
| Family | Erysipelotrichaceae | 99.86% | aa32f57e02a85df092b6960cb5f8469e |
| Family | Erysipelotrichaceae | 99.45% | 5029b20fb83007ce52fb96804a3d6e87 |
| Family | Erysipelotrichaceae | 99.83% | 97f0801feb2bfdfb0b5d4d714795bff4 |
| Family | Erysipelotrichaceae | 99.31% | bc40d9d9ca35b80cd276b05421565270 |
| Family | Erysipelotrichaceae | 99.94% | a9f66dd955e1ea9d0b9c5c3d09a8b962 |
| Family | Erysipelotrichaceae | 93.89% | 35f9b51c23231d8f5391d7af1de4d3db |
| Family | Erysipelotrichaceae | 70.11% | 74b711d869b64e8c75f9fc18a976dfbb |
| Family | Erysipelotrichaceae | 87.58% | eb5cda66b9c96e1deefda3efca797237 |
| Family | Erysipelotrichaceae | 71.97% | a02afc6413139150b07e4183d2ed8a18 |
| Family | Erysipelotrichaceae | 89.95% | 5ff48f980ae842758502b938c0080fc5 |
| Family | Erysipelotrichaceae | 87.11% | 190c884cf6a868f297e94f73398c58a1 |
| Family | Erysipelotrichaceae | 70.89% | 84db1bec972c51112ed5b68acbfef48f |
| Family | Ethanoligenenaceae | 77.69% | 7bc4d9bed60d6718ff00299412a2f984 |
| Family | Ethanoligenenaceae | 77.73% | 6d8012c2a00f00748df30826d793eda5 |
| Family | Flavobacteriaceae | 100.00% | 2df0a7643c24560af1cef480a92ae79f |
| Family | Flavobacteriaceae | 100.00% | 41a6af0f864650b53db4153915e73fa4 |
| Family | Flavobacteriaceae | 100.00% | eb47d283406279972b470c004ed9fc63 |
| Family | Flavobacteriaceae | 100.00% | e2d40ec09ca865cbe74b52f16d1224ce |
| Family | Flavobacteriaceae | 100.00% | 7ee1dd6de8b0cdac7a19c299dfb7850d |
| Family | Flavobacteriaceae | 100.00% | 8489e8f84ac0fea41f7bb26beb398883 |
| Family | Flavobacteriaceae | 100.00% | b8f16674dce01a34ad1ab83953c34de3 |
| Family | Hungateiclostridiaceae | 81.19% | aaa26df3d86af0c0e65d3fefb21221b7 |
| Family | Lachnospiraceae | 100.00% | 70a36c40ae08b8836cd792d5e7d750ac |
| Family | Lachnospiraceae | 100.00% | c9b7cc76757eff1d7d4eec00295231cb |
| Family | Lachnospiraceae | 100.00% | b0e6ded51aa3909443ce52a43ec3e4ed |
| Family | Lachnospiraceae | 100.00% | 03326c41b7a51a0ae072dc4f9d56e0f2 |
| Family | Lachnospiraceae | 100.00% | ed37e8c207d50884b963b9dc341a6c31 |
| Family | Lachnospiraceae | 100.00% | 9e5a324f9d2643d23722542fd870e728 |
| Family | Lachnospiraceae | 100.00% | 71b650126bbf3de1ecd11a7026ba0b92 |
| Family | Lachnospiraceae | 100.00% | 1b772c39491ae158e2dc8986a4d00f1a |
| Family | Lachnospiraceae | 100.00% | 25fd7d022cfe74fbc32213077a6fbaa4 |
| Family | Lachnospiraceae | 100.00% | 0e8e51109876a036bc0589f726e7486c |
| Family | Lachnospiraceae | 100.00% | 9f582dd43fa9bce86f70bb656beab2a0 |
| Family | Lachnospiraceae | 100.00% | f527d4c3c3eee225fed036f26fcb5b3d |
| Family | Lachnospiraceae | 100.00% | 69ff18972de4a909f5af8103170ec1d8 |
| Family | Lachnospiraceae | 100.00% | 9f02d0e7e6f9e5c3dd5bbaa9191e0296 |
| Family | Lachnospiraceae | 97.83% | 44fa7dee875af673270d85f01e180694 |
| Family | Lachnospiraceae | 100.00% | ba74bf01244fdd86b3f4461989a5a611 |
| Family | Lachnospiraceae | 97.77% | 565cd8b74bba0cf0d086dcdb54a2d422 |
| Family | Lachnospiraceae | 100.00% | f8bfdf252c5b70a79f839574e0cf66fb |
| Family | Lachnospiraceae | 100.00% | 2f1b53845f509841c7f8efea08595eb4 |
| Family | Lachnospiraceae | 100.00% | b46a12b1a9cb8b02ab220b9a29c70560 |
| Family | Lachnospiraceae | 100.00% | 06ea9217cc0dcb5be51c72468a1adb8b |
| Family | Lachnospiraceae | 100.00% | 6b0237cc8d94e9a414d59f674b772db6 |
| Family | Lachnospiraceae | 100.00% | c43b650e5e6e4eb0b2745947756cdc4b |
| Family | Lachnospiraceae | 100.00% | 60c687f67b45a67e42b3864647e309b7 |
| Family | Lachnospiraceae | 97.77% | cd1c3ea8e74420d1f3db89c27c76635e |
| Family | Lachnospiraceae | 100.00% | 7a4e306921006104594515716909d53b |
| Family | Lachnospiraceae | 100.00% | 8704e53d2741e6a7e445f2c342b5ff69 |
| Family | Lachnospiraceae | 100.00% | aa6b14197bb6bb49b1af51522bfa8c7b |
| Family | Lachnospiraceae | 100.00% | 1dc85a860a4798b6fe5f5df0fc0eda27 |
| Family | Lachnospiraceae | 100.00% | 52f521f32be3e9d32e512105ec0b8539 |
| Family | Lachnospiraceae | 99.75% | 4b751f6667600163d4073345cf53d26d |
| Family | Lachnospiraceae | 100.00% | 97dec9e532fd5eb6a1b2426a0dfde762 |
| Family | Lachnospiraceae | 100.00% | 8073fc46f2784f129754e1214709df9a |
| Family | Lachnospiraceae | 100.00% | 724d8c7864f48ba6ae2613e36347dd82 |
| Family | Lachnospiraceae | 100.00% | ab688a40931b24c16b4b2f89c64199e2 |
| Family | Lachnospiraceae | 100.00% | 65a95dd178aa9113077af1633a57ee4e |
| Family | Lachnospiraceae | 100.00% | a656277fcf5e43c2bbb9cb7c10aeae69 |
| Family | Lachnospiraceae | 100.00% | c8187245993a11ec7545c9e9cd676faf |
| Family | Lachnospiraceae | 88.52% | abfafe14152456ad15f19f658c8ef95c |
| Family | Lachnospiraceae | 100.00% | 604818f1d28771d788df66b1e7c7bd50 |
| Family | Lachnospiraceae | 100.00% | 007f16a44b0824701b63a06cff0c0581 |
| Family | Lachnospiraceae | 100.00% | b2578bc078e44790c7c69d5ed61382d9 |
| Family | Lachnospiraceae | 100.00% | f23c6702e7daa130cbdaff0f42c0868c |
| Family | Lachnospiraceae | 100.00% | ca69d1d9a49d1474f0b3eaefcc230422 |
| Family | Lachnospiraceae | 100.00% | 39ba5e4991526a38438560e46841fcae |
| Family | Lachnospiraceae | 100.00% | 8849de35920df2884a82861e615d8d95 |
| Family | Lachnospiraceae | 100.00% | b1c7e2f45c731b617e51d5a524b5bcd3 |
| Family | Lachnospiraceae | 100.00% | 38cfc7ad1255c9088d86ba0d74c1e2c1 |
| Family | Lachnospiraceae | 100.00% | 92f2fa273b81efeb225c34d73c76fd3e |
| Family | Lachnospiraceae | 100.00% | 83f7995fdb5172268715c14e107952ea |
| Family | Lachnospiraceae | 100.00% | 91cdb6cb8da81acf31f99beb0f4d2169 |
| Family | Lachnospiraceae | 100.00% | b7429732fe69eb8d477a92115d2550e6 |
| Family | Lachnospiraceae | 99.99% | 04d1e667de159d11ef51d49c21243d53 |
| Family | Lachnospiraceae | 100.00% | b723a6dde89c35e9a14225a9af19db64 |
| Family | Lachnospiraceae | 100.00% | 5358c7e94bcdcf01fffd3b31470cabc5 |
| Family | Lachnospiraceae | 100.00% | 54d458a3139c632bb3b49d2d0b04521f |
| Family | Lachnospiraceae | 100.00% | 5cc7fb50b81398dc7277b60f8f909200 |
| Family | Lachnospiraceae | 100.00% | 2bacf4fab7ca5646965e822736768eeb |
| Family | Lachnospiraceae | 99.76% | 8abf8ce98e75ac5255d00279e3cc6643 |
| Family | Lachnospiraceae | 100.00% | 5af4b92c9ccdc3254d40638d4cb9a025 |
| Family | Lachnospiraceae | 100.00% | 1a7a5dfd3e05ab6b2819bf30db301f4c |
| Family | Lachnospiraceae | 100.00% | 2b2ddf822da95c452dcb674a502c6571 |
| Family | Lachnospiraceae | 100.00% | cb0e89325d7c861a79f5cd31345a7654 |
| Family | Lachnospiraceae | 100.00% | b56e95b9a87fc010f16c132234c3c951 |
| Family | Lachnospiraceae | 99.98% | 98dba720f07caaa19562ea28ee845cc6 |
| Family | Lachnospiraceae | 100.00% | b5bbb45a34eeba7359829f741e9e199a |
| Family | Lachnospiraceae | 99.71% | 9a69d3d98f598c7205d2c4775ac8a4b6 |
| Family | Lachnospiraceae | 100.00% | 5e0ebc167b213aafd444131063d81151 |
| Family | Lachnospiraceae | 100.00% | 42eb2c7a2acbc9ff1ebe1c264f14799c |
| Family | Lachnospiraceae | 100.00% | f03b5c9fb9b81cc8eaa397bc4d655f5d |
| Family | Lachnospiraceae | 100.00% | 50d0f364641e9bfa4146f017a1459065 |
| Family | Lachnospiraceae | 100.00% | f662bea3813073d6864a4a121c5bf7c0 |
| Family | Lachnospiraceae | 100.00% | 6c72db165992a162082c005998b90f53 |
| Family | Lachnospiraceae | 100.00% | b7381cd20c35d1a273dee7ed96e0c41e |
| Family | Lachnospiraceae | 100.00% | 5db31a93a8281c49f966c3f80df81506 |
| Family | Lachnospiraceae | 100.00% | eb22400ab5e7dc500311e53420e8155f |
| Family | Lachnospiraceae | 100.00% | 55da4ed866ede7e7af98dd81d46a0a75 |
| Family | Lachnospiraceae | 99.95% | 309a1ec807196e34fb200cc0b886edc0 |
| Family | Lachnospiraceae | 100.00% | bc6c1710dea3181b936f40af57fc0ce0 |
| Family | Lachnospiraceae | 100.00% | 4e234354fcfb02b89a3e8924287a6d20 |
| Family | Lachnospiraceae | 100.00% | f34c4987b03016ed8bd9a8722777b3c6 |
| Family | Lachnospiraceae | 100.00% | 801d89a8f77af229d68a9248f2314594 |
| Family | Lachnospiraceae | 100.00% | adfd67fa102e9024fc16a8c9c18fbd0a |
| Family | Lachnospiraceae | 100.00% | 14e8ebd8ab54ef42f7aed29a0ff35d79 |
| Family | Lachnospiraceae | 100.00% | 3e8a5dc05ba2dee117c2619b7a1a043f |
| Family | Lachnospiraceae | 100.00% | 76ee9b747e571b06970c2e6305ed814b |
| Family | Lachnospiraceae | 98.04% | f4854e389c9f5777ad29f63d672cf90f |
| Family | Lachnospiraceae | 100.00% | 3927a970f80fa761bcf71e01618a13cf |
| Family | Lachnospiraceae | 99.99% | 2191ca7741b6af2e7b8b5e3b3df96d27 |
| Family | Lachnospiraceae | 100.00% | bc2cb17dde41433cc649bd94596ef349 |
| Family | Lachnospiraceae | 100.00% | 663557dc5add4ebe30875173809aa7fd |
| Family | Lachnospiraceae | 100.00% | 73c5aebbe46d8bd07ed8c42c6229cf0d |
| Family | Lachnospiraceae | 100.00% | 15c3fdd59c546e1b095d3b17c4a88392 |
| Family | Lachnospiraceae | 100.00% | a1b91b12a991ca625eb9969ca0d3f465 |
| Family | Lachnospiraceae | 99.98% | a62c358e307144b18cc8b4e355fd168d |
| Family | Lachnospiraceae | 100.00% | d85c2412f5a354f4c2881e1655212512 |
| Family | Lachnospiraceae | 100.00% | 5013055e67be56f99eff6a462eb19b73 |
| Family | Lachnospiraceae | 99.35% | 31c05eebecc415faefbf23025baaede7 |
| Family | Lachnospiraceae | 100.00% | 1df06b9b23797234fed16e94d2fc70ef |
| Family | Lachnospiraceae | 100.00% | 1b85eb193db964b7d4732d56cdfb4d39 |
| Family | Lachnospiraceae | 100.00% | 9874b970155d9de34fea0671df5ae505 |
| Family | Lachnospiraceae | 100.00% | 94141cd1a17b0f47f663817507d7cd94 |
| Family | Lachnospiraceae | 100.00% | a270138f99442fb39a2f7a8290190f88 |
| Family | Lachnospiraceae | 99.96% | 8fd260b5bae953d012de108430b1b782 |
| Family | Lachnospiraceae | 100.00% | f3f165b573e8fa091c3983f218e6d025 |
| Family | Lachnospiraceae | 74.15% | 334c767b0b457aa40f9e2c53c7eb4e5d |
| Family | Lachnospiraceae | 99.99% | a5a12d88c6104db75ab362a659d7a839 |
| Family | Lachnospiraceae | 99.99% | c29d62c516ac56b57e2ae54b30ba3a35 |
| Family | Lachnospiraceae | 99.97% | c40dcadd8a4750936ed1b66b242ac0f2 |
| Family | Lachnospiraceae | 100.00% | c942bdee6583552dd43a9e6e149a4d9c |
| Family | Lachnospiraceae | 100.00% | 16b30f25b238bf943c9a08255ddf57b3 |
| Family | Lachnospiraceae | 99.95% | 190432b8f2000ffa0cd8a62684abc01a |
| Family | Lachnospiraceae | 100.00% | ae28a392c97b872074931ab8a2a74d2c |
| Family | Lachnospiraceae | 100.00% | 5083605980e683579da7348155c8bd6c |
| Family | Lachnospiraceae | 99.99% | 7c61a1a76998ee6b52b006a0884630f1 |
| Family | Lachnospiraceae | 100.00% | 02507c39c4add2b07d541b4075cb6423 |
| Family | Lachnospiraceae | 100.00% | b4070dbbc353f53ef83f81c094374e66 |
| Family | Lachnospiraceae | 100.00% | 10c7670d508d17ae0155e985083c94ee |
| Family | Lachnospiraceae | 100.00% | 81ba5ed218a2257d99fca5cafb64ef5a |
| Family | Lachnospiraceae | 100.00% | de10348e8fde5e2a701e61624f5dcac3 |
| Family | Lachnospiraceae | 100.00% | 9f17f3b2b4cec9c11388b7db6b77e659 |
| Family | Lachnospiraceae | 100.00% | 3f391cc69238ac186df6493810f77dd3 |
| Family | Lachnospiraceae | 100.00% | c25c968ec56193c2eac93a738242b657 |
| Family | Lachnospiraceae | 99.98% | aed23a55894be6d2d5cc8fa62771d4d4 |
| Family | Lachnospiraceae | 100.00% | a9a348ccc86598e2dd867ea7e2a6ede5 |
| Family | Lachnospiraceae | 99.90% | e52a61c6117a1e7789053bedaa8178bc |
| Family | Lachnospiraceae | 100.00% | d947d881f1b0657bbaba9a6976695067 |
| Family | Lachnospiraceae | 100.00% | 25f9499f00afe05aecaa0386f1b0c8ab |
| Family | Lachnospiraceae | 100.00% | a463d306a6019a4c3a5f8e485a797950 |
| Family | Lachnospiraceae | 100.00% | f4b054a53db3a5a9e2f242cd2086fff5 |
| Family | Lachnospiraceae | 99.98% | 44d96fe56ce65194289c1c8d8515ef84 |
| Family | Lachnospiraceae | 100.00% | 3c26818cc726687e7b9fb8922f930993 |
| Family | Lachnospiraceae | 99.85% | 947e819f9f5ca5374c00a1b5558fe3e9 |
| Family | Lachnospiraceae | 100.00% | cfe45c07c498fe971f3f5afa4069a373 |
| Family | Lachnospiraceae | 100.00% | 009d4d6a638843b2f1b4f7e63971ea7a |
| Family | Lachnospiraceae | 99.98% | e2fb349d0e894c7393c71de650509110 |
| Family | Lachnospiraceae | 100.00% | 204f02ee8ffa45a56c694f677d65b4e0 |
| Family | Lachnospiraceae | 99.98% | 603fd2d5a90c3cf86febe3da7496cdae |
| Family | Lachnospiraceae | 100.00% | 88fb463412a6f5a7742e1db9b1986679 |
| Family | Lachnospiraceae | 100.00% | 04988107ee1cf9fe9bc49e7e57185a94 |
| Family | Lachnospiraceae | 100.00% | 247789403f01618ae666a98f4db99e9d |
| Family | Lachnospiraceae | 100.00% | 295761d06ca600a4db88a2643482b229 |
| Family | Lachnospiraceae | 99.75% | 64f8074dc6b8a7a5657a93362b089fcc |
| Family | Lachnospiraceae | 100.00% | c497578ab08b2708f11acca3ba1f3222 |
| Family | Lachnospiraceae | 100.00% | 260546296997f55097175a5f79b9b619 |
| Family | Lachnospiraceae | 99.99% | f5989bb924cfedacc7ee18c093243e96 |
| Family | Lachnospiraceae | 100.00% | 9d79a9e520ad61498778ea0faceaa8bf |
| Family | Lachnospiraceae | 100.00% | 5fff84e2156dbce1330fe7bd9aadab09 |
| Family | Lachnospiraceae | 100.00% | 3cd819241f3ea23ff9c9699bd6ca5a21 |
| Family | Lachnospiraceae | 100.00% | 37795bb943b26493101232c0f8058d0f |
| Family | Lachnospiraceae | 100.00% | 21ca306b8a4852349761b55ae839e4f5 |
| Family | Lachnospiraceae | 99.99% | e3658799c700e6ae395ca95960b608e9 |
| Family | Lachnospiraceae | 100.00% | 0c4476ee286a62924f6d20a9c2a0dc8f |
| Family | Lachnospiraceae | 100.00% | 62858e55ed50a6da0ee076271e229f0a |
| Family | Lachnospiraceae | 100.00% | d9eef805ca3b10fb5a7ea79a9727aa7a |
| Family | Lachnospiraceae | 100.00% | 74dbce1422292a2d572da2bf1acbc5ba |
| Family | Lachnospiraceae | 99.99% | a949183ab823b2c4d4e558eeaf0f6f65 |
| Family | Lachnospiraceae | 100.00% | 5eebdd9008582709ad5fb520120a1160 |
| Family | Lachnospiraceae | 100.00% | 5f5a5584a73a57d0fa05314ead8123f8 |
| Family | Lachnospiraceae | 97.13% | e547a00e23ec747841a9da61af6a5d39 |
| Family | Lachnospiraceae | 100.00% | b7e50732e1d33878badccd079fb3382d |
| Family | Lachnospiraceae | 100.00% | 33393ecc7c4c40cba075c6c6077e6701 |
| Family | Lachnospiraceae | 100.00% | 2f3364d58012fdd2af1a2f497c17c0d5 |
| Family | Lachnospiraceae | 98.29% | f2d10b540553c9f8c8452f98740d50cd |
| Family | Lachnospiraceae | 99.93% | d29077ad718b59d8e59c78736403c213 |
| Family | Lachnospiraceae | 99.99% | cf3136f0453291815e0d7a81e2eba8dc |
| Family | Lachnospiraceae | 100.00% | 93c93acf7cb300ed853442e20aab29ec |
| Family | Lachnospiraceae | 100.00% | c77072b1e0fca139ff5523564d44ca9f |
| Family | Lachnospiraceae | 99.99% | 00fedfdbdd1deb6f2d1a0875e588f4d4 |
| Family | Lachnospiraceae | 100.00% | b900235d2765e748c9d6bb38efd8e3d1 |
| Family | Lachnospiraceae | 93.90% | 0db8b3a1f75fd1d17297c402aea5ed9b |
| Family | Lachnospiraceae | 100.00% | b225d7cfbe75c9b90388ea2334d1b8aa |
| Family | Lachnospiraceae | 100.00% | 55f5cf3c40432d33868be30232e9c84e |
| Family | Lachnospiraceae | 100.00% | a46b0edfcd8ed7ef45ab93e724af9502 |
| Family | Lachnospiraceae | 100.00% | 164ca7dae13f2715de08dd979a33684d |
| Family | Lachnospiraceae | 100.00% | 71ec617a144a7e2e6d87be4c2cf19412 |
| Family | Lachnospiraceae | 100.00% | 2216a44611e50ebc8528492adc817397 |
| Family | Lachnospiraceae | 100.00% | 61a15160bc8a6a3dba6533c7b8ca40d2 |
| Family | Lachnospiraceae | 99.98% | 36c4218532ceff8aad9ce2168b6a6faa |
| Family | Lachnospiraceae | 100.00% | 084c771f1da7284ed056e57905fa7b2c |
| Family | Lachnospiraceae | 100.00% | f5c4327cbc483d4abb1c86104854c02f |
| Family | Lachnospiraceae | 100.00% | 490c57256f5bc6baeb900097e69e4389 |
| Family | Lachnospiraceae | 100.00% | 11e507cb34c556d7e1580d16b86a49d9 |
| Family | Lachnospiraceae | 100.00% | a31968a079c9c6f64507d7c3623d9eee |
| Family | Lachnospiraceae | 100.00% | 37b982b678532bf4b7f896607a2cd2fd |
| Family | Lachnospiraceae | 100.00% | 246529a300fd443349b3fe0b056e1452 |
| Family | Lachnospiraceae | 100.00% | 764c61051a501db560095a04e64ad6bb |
| Family | Lachnospiraceae | 100.00% | 2ec89ef9624f10b2d0f27423c2ce3630 |
| Family | Lachnospiraceae | 99.99% | 8f0f4494fe46a29c7a52d8ca47b09532 |
| Family | Lachnospiraceae | 100.00% | 137e0f90f70e48685c04f13fd6f00e33 |
| Family | Lachnospiraceae | 100.00% | cf78a142297b689d91928143c014710a |
| Family | Lachnospiraceae | 100.00% | 377c31878537cbd486563cdbfa4dc983 |
| Family | Lachnospiraceae | 100.00% | bc289d48f2d06b78f4cb95f2ef9fabd5 |
| Family | Lachnospiraceae | 100.00% | 9fd8609da2ff0cb681d2dc28d85b9fa1 |
| Family | Lachnospiraceae | 100.00% | 4ce485c06afccb39628eecdb642a97d6 |
| Family | Lachnospiraceae | 99.96% | c98ec45618b3645f64a4d13fe95f926f |
| Family | Lachnospiraceae | 99.99% | ad15b702afec4b4af7c3c1dddcde1102 |
| Family | Lachnospiraceae | 100.00% | c42c9415b07784ad49a7914dc01d766c |
| Family | Lachnospiraceae | 100.00% | cc5810371d17180d6f8d789f2bd54af4 |
| Family | Lachnospiraceae | 88.49% | b72700047c3adb6ddd2d1f02b18065c4 |
| Family | Lachnospiraceae | 100.00% | e317f9888f074ff9d4716933fd90438e |
| Family | Lachnospiraceae | 100.00% | 857625efebd1ac403aadd622c9b80707 |
| Family | Lachnospiraceae | 99.99% | 0a5abe7d111fa0576c0805ea8175aa37 |
| Family | Lachnospiraceae | 99.99% | a2e9e59b34e2b35a3d694c6b676bc1f3 |
| Family | Lachnospiraceae | 91.67% | 7e06703de4f3321f4b91660b0090e5cf |
| Family | Lachnospiraceae | 100.00% | 1b23404e631049ab29032f3dcfd69d45 |
| Family | Lachnospiraceae | 100.00% | ce0cb30e8e1b4a717a4781460033fb8a |
| Family | Lachnospiraceae | 100.00% | a6d4d31185959feb4ba2281980e31d99 |
| Family | Lachnospiraceae | 100.00% | b9b23fd639df1ad83c2710f4eba949a8 |
| Family | Lachnospiraceae | 100.00% | 0d9a07f9f8b80a1a463d1377900462fa |
| Family | Lachnospiraceae | 100.00% | c7e3ae273851b22c1b32f33859016f21 |
| Family | Lachnospiraceae | 97.44% | 1cabe4a654fce5c2978a712223384d12 |
| Family | Lachnospiraceae | 70.58% | 6bec45e5cf87da64a79de6daf3979564 |
| Family | Lachnospiraceae | 100.00% | 3baa4c74643692ff351905f774993cca |
| Family | Lachnospiraceae | 100.00% | 605e65a836bff4a459c98ef1187d1859 |
| Family | Lachnospiraceae | 100.00% | 2416947924d5cde50a14eacfe11799a4 |
| Family | Lachnospiraceae | 100.00% | 2e817b856eba4704b9019d0079759477 |
| Family | Lachnospiraceae | 100.00% | 8c0d64d021ba9bfe492aaea0b9a2742c |
| Family | Lachnospiraceae | 100.00% | b5184c7cd468373de089e46b99158cef |
| Family | Lachnospiraceae | 100.00% | a41168da848bcd75cffcdfd16c7e606f |
| Family | Lachnospiraceae | 100.00% | da112e0e657d41b9597ff901f8097f5d |
| Family | Lachnospiraceae | 100.00% | 8f835503258ae3ec73b1064d98889a5c |
| Family | Lachnospiraceae | 100.00% | 5ddcf54bb29c51a557cddc9fe88d6952 |
| Family | Lachnospiraceae | 100.00% | b06197a862d8e284d524110063452eee |
| Family | Lachnospiraceae | 100.00% | 4aaab71371c16bbc23bf7c809c18b717 |
| Family | Lachnospiraceae | 100.00% | add2012fce88794d55cffb9a53e4bb12 |
| Family | Lachnospiraceae | 100.00% | fd0495a8a3cd3e9c82d9da5b3fe0d984 |
| Family | Lachnospiraceae | 97.30% | f19b97acf59ca76102ea7fe2d3a22e4f |
| Family | Lachnospiraceae | 99.99% | 6bbce516b4c02e0bff0323d81394375e |
| Family | Lachnospiraceae | 100.00% | 4654b89324cd525989348e50bc984e89 |
| Family | Lachnospiraceae | 100.00% | c599d3fe38e74700768e800d8368b516 |
| Family | Lachnospiraceae | 85.03% | ef7b5fa9f134fd8e3e14f6765225534a |
| Family | Lachnospiraceae | 100.00% | f4494eecc1065b6802c65cc3e35a17f3 |
| Family | Lachnospiraceae | 100.00% | 6cfe942485fa57b76fb174256f527dcd |
| Family | Lachnospiraceae | 99.98% | 7442c2d9ce414448398e305bcd71c6a5 |
| Family | Lachnospiraceae | 100.00% | a2684edbc21e1567a8f3f9a9dbd70b34 |
| Family | Lachnospiraceae | 100.00% | cc502cb9315c620608c4ba6bb0c10223 |
| Family | Lachnospiraceae | 99.34% | 945286916ee6472c783004bdf011fce5 |
| Family | Lachnospiraceae | 100.00% | e5f62696981016c899b4774b57596c8d |
| Family | Lachnospiraceae | 100.00% | adf261dcb80d437cc9171ce076989609 |
| Family | Lachnospiraceae | 100.00% | 0cd62bed5cd39db735da6253553763f4 |
| Family | Lachnospiraceae | 100.00% | 495bbe626f17e3c6802d86dedaf4abe3 |
| Family | Lachnospiraceae | 100.00% | e40950d13e6b231345c0f506ad511233 |
| Family | Lachnospiraceae | 100.00% | f9ca9cd2e7337ff0f9205b640d376d4e |
| Family | Lachnospiraceae | 100.00% | 63a2b786bb3b6d8b6b34b2fdefbba163 |
| Family | Lachnospiraceae | 100.00% | c654cccd9788b1cb4072056919c9098c |
| Family | Lachnospiraceae | 100.00% | c361165c378bf9c31a21aeb9e68f476e |
| Family | Lachnospiraceae | 99.94% | ed00eebbee404fb4aac56c6f7e62ce3a |
| Family | Lachnospiraceae | 84.71% | a2acaed68a31965ba37ec1fc9598e6be |
| Family | Lachnospiraceae | 100.00% | 0e29fc29c56670729ab97295a6cf349c |
| Family | Lachnospiraceae | 100.00% | b9729a82b4bf297dacc9513fdbfaf5d1 |
| Family | Lachnospiraceae | 100.00% | 4e0d8a98e733c87bd897add0c437c7a0 |
| Family | Lachnospiraceae | 100.00% | 8d14ff66495090a441d45ffdfc327362 |
| Family | Lachnospiraceae | 100.00% | 76248426ede7dc678ad07ecef54d5dc6 |
| Family | Lachnospiraceae | 100.00% | 5822966fe85323037e1a1050def3297c |
| Family | Lachnospiraceae | 99.97% | c0d2a7c535bebbbd8d407742cdad9b2e |
| Family | Lachnospiraceae | 100.00% | 6ba910ad9d74d0254286df991763cd90 |
| Family | Lachnospiraceae | 98.90% | 03f9887594261b1c1217d37f44e611f6 |
| Family | Lachnospiraceae | 100.00% | 2d0f4fd1dcd7c19d31faa5072faa9d7e |
| Family | Lachnospiraceae | 100.00% | b5485ccfc746aa97a5b8ddeb6c7c5ecb |
| Family | Lachnospiraceae | 99.98% | fc7590587c66f917acf68f343adefc55 |
| Family | Lachnospiraceae | 100.00% | 08a8228c9e07c94f8e61671451d3f335 |
| Family | Lachnospiraceae | 100.00% | 1445178a8073c19d8b6721129d71b8b4 |
| Family | Lachnospiraceae | 99.99% | 9f127d1c3aa6571e95d6ba8f00a16cd2 |
| Family | Lachnospiraceae | 100.00% | 19442c181afe86a88139a80f01eeafa7 |
| Family | Lachnospiraceae | 100.00% | d96ea05161574080101988c4a7b67e75 |
| Family | Lachnospiraceae | 100.00% | 92f8092934d84f7dfe12cdce49a7e146 |
| Family | Lachnospiraceae | 100.00% | e0f2ae2ac02423643c9eccd7a0340dd2 |
| Family | Lachnospiraceae | 99.99% | c30841384a1f68073c5cf246971ba1b0 |
| Family | Lachnospiraceae | 100.00% | 1dc412f8315dce173ad1d7bb4b3be486 |
| Family | Lachnospiraceae | 99.93% | 8c2a17b878314a3bfb681afb69b3e469 |
| Family | Lachnospiraceae | 98.84% | 65d76a4aa57464bcccf55097f972cb6c |
| Family | Lachnospiraceae | 99.98% | fc0fec8980a52f1925e5ce0983189cc1 |
| Family | Lachnospiraceae | 99.99% | 86be49fe0bfedfe67161bf0a028c7176 |
| Family | Lachnospiraceae | 100.00% | 9f510db5c87a4173ad4727f7123144e0 |
| Family | Lachnospiraceae | 88.73% | c83acd27e6daf689928c3523a1963cd5 |
| Family | Lachnospiraceae | 99.99% | 6403e742caee8ae41b6da1b1bb0c3a1b |
| Family | Lachnospiraceae | 96.23% | 217f70b3b5c40246f193639f849fcd54 |
| Family | Lachnospiraceae | 99.99% | c0149423e8b50359538780c574a03d9e |
| Family | Lachnospiraceae | 99.92% | 4d97af288379b80821406e27c5bdad0d |
| Family | Lachnospiraceae | 90.13% | 70a0cfff9f344413924f900947c61a29 |
| Family | Lachnospiraceae | 92.84% | 7a34c64555f12d1efaec46fb083d17f5 |
| Family | Lachnospiraceae | 100.00% | 5a17d4e620c5594ca92f367ad797a3b7 |
| Family | Lachnospiraceae | 96.54% | 0e49d6c785188de50a77b2c99bed8436 |
| Family | Lachnospiraceae | 86.82% | 0fa8c76d8ceabed3f6c476b5b5d75c96 |
| Family | Lachnospiraceae | 100.00% | 0915292f45d7092e3e9a5c1e5a5e2d27 |
| Family | Lachnospiraceae | 99.98% | dc4b11fc6d455a6b01f1b65428b40538 |
| Family | Lachnospiraceae | 100.00% | 1b2d250a25e98fa1f9751581bae31ba9 |
| Family | Lachnospiraceae | 100.00% | 1e603231c02e0e95ab09036b9af8aff2 |
| Family | Lachnospiraceae | 100.00% | 82849cf50a7a278f8f6597c42abacf4c |
| Family | Lachnospiraceae | 99.40% | f5300d8c1a777babd821aaa6cc8a930d |
| Family | Lachnospiraceae | 100.00% | 59f1ad241e8884043b81c287de8719ad |
| Family | Lachnospiraceae | 100.00% | 67f8c8abc23c7cfa5e157acf0a29b00c |
| Family | Lachnospiraceae | 100.00% | ed29c73d1b8affc1627214d8232a5321 |
| Family | Lachnospiraceae | 99.95% | 0a27a19dfe8fa1df920eb0409b4cdae2 |
| Family | Lachnospiraceae | 99.99% | bed25123b974459fc388213d09a7f2c8 |
| Family | Lachnospiraceae | 90.25% | 02c2c677b40685ee6a7a48b9e0aea727 |
| Family | Lachnospiraceae | 99.38% | 74b5be5604c2fff5c4e3d92910e1b72a |
| Family | Lachnospiraceae | 99.98% | 5c699da3eaddceb0d33f10e48f9d0982 |
| Family | Lachnospiraceae | 100.00% | 8bed8ea7e1ec092d9bae7326305f4237 |
| Family | Lachnospiraceae | 99.79% | f5dc4bace0f0aa2473d699245e6eb577 |
| Family | Lachnospiraceae | 86.33% | 5e39aed802bbb316d6fae014375ae085 |
| Family | Lachnospiraceae | 72.15% | 85fa53cae96bf049c480000321860b81 |
| Family | Lachnospiraceae | 72.21% | 54393956f1f88af4cc397c2dcf9d2712 |
| Family | Lachnospiraceae | 87.27% | e9acc267b692851e13ee55c1d264cd49 |
| Family | Lachnospiraceae | 100.00% | c332245f336a09c2836aa66b2177e1d9 |
| Family | Lachnospiraceae | 100.00% | aa2e80f391cf26fe4de3e98ed791a579 |
| Family | Lachnospiraceae | 99.98% | ace0e0aeddbd1f39dd98b56fd56a7fbf |
| Family | Lachnospiraceae | 95.62% | ab4040b7037ee8d4f34a52202e2e13b9 |
| Family | Lachnospiraceae | 99.62% | a8db1a1ef2b90937978f6672b59579c3 |
| Family | Lachnospiraceae | 81.13% | 84fe154fcfee9ff4c78178441d3162ee |
| Family | Lachnospiraceae | 100.00% | 2ef149b8e742d9eed0c2b3c505f0bae2 |
| Family | Lachnospiraceae | 99.74% | 021985c1e017633fd640caed32f9992b |
| Family | Lachnospiraceae | 99.32% | 1b17294e2f9c09563f28c2ed2ae126cc |
| Family | Lachnospiraceae | 75.68% | edb26f6cd709570ae66099d06d8217a4 |
| Family | Lachnospiraceae | 100.00% | 19ad71107944cbd6c60aca30b8e15d3e |
| Family | Lachnospiraceae | 99.99% | 5c28db78072734530165bff703cdd4df |
| Family | Lachnospiraceae | 97.74% | d042e13c0647ec28f23d859d7e9b6ee7 |
| Family | Lachnospiraceae | 99.99% | cd7a3f698476e257938ff16b5779cdc5 |
| Family | Lachnospiraceae | 99.99% | a3663829e34b7b858badf59a7383172f |
| Family | Lachnospiraceae | 100.00% | c20fcf681393a8b73c796ef771ddc2da |
| Family | Lachnospiraceae | 100.00% | e3bd816df980ce9910a72fd801e07ddf |
| Family | Lachnospiraceae | 99.98% | 4e88c58bc48afc23645089993635b1d7 |
| Family | Lachnospiraceae | 99.88% | c562b19047b8192d7312d71d169bdaee |
| Family | Lachnospiraceae | 100.00% | d4a2eef855639a993f5f4cc44388220d |
| Family | Lachnospiraceae | 98.16% | 64b2d281d47324ceda77dde8a5b07bf0 |
| Family | Lachnospiraceae | 99.99% | 498dc16be72bc07a086bc8fbddb96674 |
| Family | Lachnospiraceae | 99.70% | c6a23fcd99eb2c99e8ed8c4ef3f29098 |
| Family | Lachnospiraceae | 99.27% | d946cd08bf0d5433131287caadc5310e |
| Family | Lachnospiraceae | 100.00% | 827db254aec2baa203c4e1d2feb99f45 |
| Family | Lachnospiraceae | 88.64% | 68ea1da8e7ca0658e51705ac4c610c71 |
| Family | Lachnospiraceae | 88.87% | 90d314062991646aeb955a95164fbc25 |
| Family | Lachnospiraceae | 84.89% | 757ac636d22c4806353006556036285c |
| Family | Lachnospiraceae | 98.62% | 2c204f3302a2431caca4059aa4f73a9d |
| Family | Lachnospiraceae | 90.35% | a4ee617723193a42002773ae695eaaac |
| Family | Lachnospiraceae | 100.00% | df81aa0272d556b1663d4869f4a6bff1 |
| Family | Lachnospiraceae | 99.98% | 1eda5acc099f78b320ceecf5a17bc03c |
| Family | Lachnospiraceae | 99.39% | 84c2e4551c9eed806411aa58c0e17d64 |
| Family | Lachnospiraceae | 100.00% | a254da50525e39b4c3b26683e4863bb8 |
| Family | Lachnospiraceae | 100.00% | 1209caef525847120ef0984fc29a6c93 |
| Family | Lachnospiraceae | 100.00% | 6b789f44dd647ebd3a8751ab53c91947 |
| Family | Lachnospiraceae | 99.99% | 1b1a9294ed4f96a5236610a6704d77ac |
| Family | Lachnospiraceae | 97.34% | f3e6b7e632dfa2adc62e4136e1c9e16d |
| Family | Lachnospiraceae | 99.99% | 1a1116fbea47a4106614f25fb4115c7c |
| Family | Lachnospiraceae | 100.00% | ec149130472464ad02b9b37bab772eab |
| Family | Lachnospiraceae | 100.00% | 14177b0af03d9be50180b78e2ef3be79 |
| Family | Lachnospiraceae | 100.00% | d481d96107b46eb7108a2ad6a07a135a |
| Family | Lachnospiraceae | 98.72% | 035793405e750b731bb3808a0b259314 |
| Family | Lachnospiraceae | 99.98% | b9896fbb6a81d424e85507edb3d7950b |
| Family | Lachnospiraceae | 100.00% | 9eb1fefe13cb396c38d640e5ddb3256f |
| Family | Lachnospiraceae | 100.00% | df8fc8203cf5a6004488bcd3cb8803f6 |
| Family | Lachnospiraceae | 100.00% | ff2378c0b9545be630333deea4633dbe |
| Family | Lachnospiraceae | 100.00% | 399806fa4a95909ee2b8195fb205300e |
| Family | Lachnospiraceae | 85.68% | ddbe1ae1303d5fa7435e3f8d814d765f |
| Family | Lachnospiraceae | 100.00% | d31196f69f388677158a80b54683478f |
| Family | Lachnospiraceae | 99.95% | 5c42b98c2c0faae7a42e927d5c80ef2e |
| Family | Lachnospiraceae | 100.00% | 33033d49a4dcf7f644e9ac55e0d3be59 |
| Family | Lachnospiraceae | 99.98% | 72933734c21e9fc62f00e743b93eade3 |
| Family | Lachnospiraceae | 99.99% | 8011fb3c56f1c5111be1ee9138e0c5a7 |
| Family | Lachnospiraceae | 99.99% | 0303ff137ad88a14455f14dccb37ad69 |
| Family | Lachnospiraceae | 99.96% | 6b48172a1c4227719173f6ac5d24bda0 |
| Family | Lachnospiraceae | 99.49% | 22c11031ae049351eceefc29e1584a9b |
| Family | Lachnospiraceae | 99.83% | fa0b2e24054c41ed9ea860b6ac50d091 |
| Family | Lachnospiraceae | 94.19% | b5acd53390144b593a61ebc4ddf793a1 |
| Family | Lachnospiraceae | 100.00% | dc7b294bcef45ab1f2346e37c026114d |
| Family | Lachnospiraceae | 99.99% | c01e97a0efe7b7c7776546335d3fb525 |
| Family | Lachnospiraceae | 100.00% | d11a7682d31639752232da7d0632ffad |
| Family | Lachnospiraceae | 99.86% | 69265a24a28813e8035d94372743b199 |
| Family | Lachnospiraceae | 100.00% | 670e3e541d9e5ff3f8d151611a493f59 |
| Family | Lachnospiraceae | 99.87% | 2622aa6931ca8f180e0114d0c0c39307 |
| Family | Lachnospiraceae | 100.00% | 8439bf1a6d609c4b299c2d1998471293 |
| Family | Lachnospiraceae | 99.59% | f2eebf3464fd573f3385f995059481dc |
| Family | Lachnospiraceae | 99.27% | 10828a5aef242901b559d6f5357c3676 |
| Family | Lachnospiraceae | 98.24% | 3bf3c3196e80c787922c238f3ee30d99 |
| Family | Lachnospiraceae | 100.00% | 19cf3be20ac190dc98f89f98b8c74d30 |
| Family | Lachnospiraceae | 99.98% | e79c339f4619453b19d7c1c395356599 |
| Family | Lachnospiraceae | 99.99% | 8540aac1e14f18f694d6bbd3332584b2 |
| Family | Lachnospiraceae | 99.97% | c7ab1566fd2a3479b7b6223ead30a5a3 |
| Family | Lachnospiraceae | 90.53% | 9644d425ba4cc9090dc69a674bdffc6b |
| Family | Lachnospiraceae | 82.78% | 290f797e956d56060ed361c0e3da4208 |
| Family | Lachnospiraceae | 76.39% | dd1a5f4c871d306d64898f1e71f444fe |
| Family | Lachnospiraceae | 76.81% | 0b07d1a0506629c57be993c25c425288 |
| Family | Lachnospiraceae | 74.51% | 28e90650a26677a63273b5e9d500185e |
| Family | Lachnospiraceae | 96.23% | 947e43189324f6e77beb180fb5fb9b2a |
| Family | Lachnospiraceae | 74.39% | 9d4af38177c2dcc98e365bad37fe4567 |
| Family | Lachnospiraceae | 89.71% | 48dec3c0d581e289158d969cced18c7b |
| Family | Lachnospiraceae | 73.52% | 9a2aa2095825f10a46f2c1041708cd44 |
| Family | Lachnospiraceae | 73.49% | b2d8798fe547762bf192da311974b23d |
| Family | Lachnospiraceae | 81.77% | 6061c37aea30958aa3f60c818bf27b93 |
| Family | Lachnospiraceae | 76.53% | 760fa6c8417968760bcd045ef9b28186 |
| Family | Lachnospiraceae | 77.00% | 35ec9e545f963349522207577090a017 |
| Family | Lachnospiraceae | 75.47% | b357839c70bdf400c9d3171983c2b57b |
| Family | Lachnospiraceae | 73.54% | eb8285a555dedbfdf7d910c705a98d20 |
| Family | Lachnospiraceae | 80.45% | 1b1a35e8a5c1b9c6423f39004691ccb5 |
| Family | Lachnospiraceae | 72.45% | 0d849a9deacc35c0d18e8f50809eec32 |
| Family | Lachnospiraceae | 86.17% | b1ecb03e5175fd7aaf84eec8252881bd |
| Family | Lachnospiraceae | 75.74% | a9deb1855d3f3f00f5f0546512e9d4fa |
| Family | Marinifilaceae | 80.60% | d1ed40eb6f866fca5696ac61dec6a6b5 |
| Family | Marinifilaceae | 82.10% | c6d0a3e966e846caf68ca44cd6bad20a |
| Family | Microbacteriaceae | 91.91% | 857256f83290d5d067c8108e0bf6d805 |
| Family | Microbacteriaceae | 84.26% | 34019710e7fc70869ba6e3f4f699e3a9 |
| Family | Microbacteriaceae | 74.91% | d2d5dbd142b9ccf77a87c7138df226b8 |
| Family | Micrococcaceae | 72.44% | e5e053c79e2d93113e8d236fe83e800b |
| Family | Morganellaceae | 75.03% | 6add7f76dd114e0086af799aeefa1b40 |
| Family | Nocardioidaceae | 86.72% | c75d1f5e82ab504f580fc3058f9dc83b |
| Family | Orbaceae | 99.71% | f5b6d0c1d9b774c9e724f3a2838bad76 |
| Family | Orbaceae | 97.54% | cb739a1695c582e45aedd35ce93a7225 |
| Family | Orbaceae | 98.37% | 5fd8c92d277029c49e76e7cea24e4cc9 |
| Family | Oscillospiraceae | 73.91% | fcda41251c1548683dd34cfc3365af6d |
| Family | Oscillospiraceae | 99.96% | d75986170e2c767a9aed8a2056919241 |
| Family | Oscillospiraceae | 99.98% | ec9d6aa7dd52633c89544512e9a13e10 |
| Family | Oscillospiraceae | 75.20% | 0e80dc0673c6462d72eb61a60e2ace79 |
| Family | Oscillospiraceae | 99.96% | 6ed87ab04d4238ac53eeead1589fba41 |
| Family | Oscillospiraceae | 99.93% | 63a65d7a461df7bde92ab76a5bf9f070 |
| Family | Oscillospiraceae | 99.79% | 039c13ae9d4226dcf16a2978ca71e4ea |
| Family | Oscillospiraceae | 99.99% | e213066abe583a032db8bbfc8e8f9323 |
| Family | Oscillospiraceae | 100.00% | 8aae2f272ce78538139d75010b4d3acc |
| Family | Oscillospiraceae | 99.98% | 36161850701e67a2768cc9f0b73761de |
| Family | Oscillospiraceae | 99.99% | 5f76cf3e066b2cf50e57ee34179c9e0a |
| Family | Oscillospiraceae | 99.99% | 5dcb093bea06fd2468caa058e8de09da |
| Family | Oscillospiraceae | 99.66% | a772795d2682c02cd6a0eb20504cb445 |
| Family | Oscillospiraceae | 99.94% | d2746193a67d255587d4ca1807ecc986 |
| Family | Oscillospiraceae | 99.99% | 4e1ce46f8d3d40b705b6d30790c87276 |
| Family | Oscillospiraceae | 99.99% | 75b1ebccd9168a67c4f3a55ceb098406 |
| Family | Oscillospiraceae | 100.00% | 91395adb900d01726e40693ee9b44862 |
| Family | Oscillospiraceae | 99.99% | eca3445ebf6331f0007e00e29911f26f |
| Family | Oscillospiraceae | 99.18% | 96a0e15c3dc3b13748a4d55f47b502cf |
| Family | Oscillospiraceae | 99.85% | 396d34b9bd0f33094ea0c52bc1560ea1 |
| Family | Oscillospiraceae | 94.91% | 861cd362ba9e74613d03a705f1544540 |
| Family | Oscillospiraceae | 96.71% | da5d6236cb3ed1a78165e379614ee6ff |
| Family | Oscillospiraceae | 100.00% | e5b4fc3fc3bbe6f76c584a2db67acbfb |
| Family | Oscillospiraceae | 99.78% | 347dd69ab48c18642b8e3775643c9b81 |
| Family | Oscillospiraceae | 100.00% | 9005273450b490d77190db86afdc6b05 |
| Family | Oscillospiraceae | 99.71% | fd33cd1b14efe007e299df7556e9f76c |
| Family | Oscillospiraceae | 99.97% | 061e222ad8c72162506ce7fc5d0a015d |
| Family | Oscillospiraceae | 100.00% | af6ec4363e65be3ff9559e67c019aa6b |
| Family | Oscillospiraceae | 99.96% | 5a30e882eae129126bb4d69958106ceb |
| Family | Oscillospiraceae | 99.90% | 9ee4b352287870f482e71877fd0e3873 |
| Family | Oscillospiraceae | 99.82% | 2df63f24022a91df63254d23e3454278 |
| Family | Oscillospiraceae | 100.00% | 860bb38bfbdeed700b4a964793acf875 |
| Family | Oscillospiraceae | 100.00% | bd73b5b4cca8ccdd9ce7fcc29611df4f |
| Family | Oscillospiraceae | 73.81% | 865412358f3a41abfca92e7d8253ca3c |
| Family | Oscillospiraceae | 99.89% | 198b58021ede530e314aad0725cc5172 |
| Family | Oscillospiraceae | 99.91% | 6144ba21e427157678ee663d6bd81453 |
| Family | Oscillospiraceae | 99.78% | b1902fa68f25e61b327908c11bd8f54f |
| Family | Oscillospiraceae | 99.65% | 9edddfa1aeb8cadf8edf38c1539a166a |
| Family | Oscillospiraceae | 100.00% | b657fdc47a75f448331e4dbfeea7b72d |
| Family | Oscillospiraceae | 99.94% | 6b27a1b9e70d1cc53fb58c2cdc56ac55 |
| Family | Oscillospiraceae | 99.95% | af71d0f62fd99ed246333ea1b2148ea8 |
| Family | Oscillospiraceae | 99.74% | 0322af0b87ca767bc35d356407a52607 |
| Family | Oscillospiraceae | 99.94% | 1d3bdd38477e59b14753be779b1125b8 |
| Family | Oscillospiraceae | 100.00% | d456dc40cdcf481fd56897bd9f46ef17 |
| Family | Oscillospiraceae | 99.98% | ef4b822b0465527ebf92c5d7ed9846e4 |
| Family | Oscillospiraceae | 99.77% | 759df79c705f7d5b3da485273ef75ab2 |
| Family | Oscillospiraceae | 99.95% | 18cb43e155d489b91a242b020efc28ac |
| Family | Oscillospiraceae | 100.00% | e3fd0aef47f011d8f2a43fe6becb938b |
| Family | Oscillospiraceae | 100.00% | 0b02b820f433035893b7d393858db94d |
| Family | Oscillospiraceae | 99.79% | 5f2d014a7609c7d4ab7bee0212d0c27a |
| Family | Oscillospiraceae | 98.07% | 5f8e1d651f5cb39dadb12d371cfcccfd |
| Family | Oscillospiraceae | 99.97% | 5bfe2488e57710b527928557f2947bcc |
| Family | Oscillospiraceae | 99.94% | 76e930458de6dc7b33b5b1683d25c59c |
| Family | Oscillospiraceae | 100.00% | 03209ba228aaf2a7eb0d89ebf355c751 |
| Family | Oscillospiraceae | 99.95% | ff122767f8ccd40b1221ce5fda08fb39 |
| Family | Oscillospiraceae | 75.21% | b03cb30894d0b2b6c870216abf6761a7 |
| Family | Oscillospiraceae | 99.73% | 192ab317103368320d548e2a5396d8b4 |
| Family | Oscillospiraceae | 99.98% | 96da54963f90c3e9020420d365186ef1 |
| Family | Oscillospiraceae | 99.57% | e8e3c7aee788481ec332244c1f94029d |
| Family | Oscillospiraceae | 97.00% | 4959570a01a568fbc01fd99e71732cb6 |
| Family | Oscillospiraceae | 70.77% | 490b5d83cf3d06c8ed077e7080da65d8 |
| Family | Oscillospiraceae | 71.07% | fd25b4d3342a0c0ddb93c82f2e447d47 |
| Family | Oscillospiraceae | 92.38% | 8108091a0f1b62cbe5235cf6d8f9de57 |
| Family | Oscillospiraceae | 91.39% | e004927940108ccaa6223bb05bfb1415 |
| Family | Oscillospiraceae | 91.43% | c2b1163b07aaf7d4a9659d2da762af49 |
| Family | Oscillospiraceae | 93.63% | a400010ca99d42fc41ed8083e92d8242 |
| Family | Oscillospiraceae | 92.43% | 2b46db1948966304de875239aac7ddb1 |
| Family | Oxalobacteraceae | 86.71% | 97c89d4e62304d98f20cdee45a1ac512 |
| Family | Oxalobacteraceae | 72.61% | 953089ba2ae7db53d86a16cfdfb85bcc |
| Family | Oxalobacteraceae | 96.22% | 9c175a7648d752fef47fadaa89de2092 |
| Family | Oxalobacteraceae | 79.45% | 91deb1068bf33838fa61a80afc1d6fd5 |
| Family | Oxalobacteraceae | 88.14% | bd028fc8ce2dbd1ed555f741a282a7d0 |
| Family | Oxalobacteraceae | 86.65% | 38ec74ecd31aaaadfc16dcb4448a2261 |
| Family | Oxalobacteraceae | 89.63% | e429c40b83811835fdd6bb2007711709 |
| Family | Oxalobacteraceae | 75.71% | c464820b91605f9ad3c07454a1661f13 |
| Family | Paludibacteraceae | 88.69% | d2fbf63c77ab269dd09dca2b91778425 |
| Family | Pectobacteriaceae | 76.16% | 568da3e8d002eb804b6e400a62be65a5 |
| Family | Pectobacteriaceae | 70.26% | 795878079f768b6c7ae4092269c8e04a |
| Family | Pectobacteriaceae | 71.72% | 7d852996d7438b3ba757854c26ba8ec1 |
| Family | Pectobacteriaceae | 83.44% | 0f92f2c75495efc8e6b4497c3b9567a4 |
| Family | Pectobacteriaceae | 81.08% | aa0c899d77f4f6b431b845fd2135bc44 |
| Family | Peptococcaceae | 99.37% | c6bf48db5f74a782305e536b1c34c2bc |
| Family | Peptococcaceae | 99.42% | b0042687f4aa3c7118bbc859f1280896 |
| Family | Peptococcaceae | 99.63% | 25c54eab41c8df481ae6c8711a8882bf |
| Family | Peptococcaceae | 99.32% | f5003409e905bc9dbe3a2f6a47b1b0a0 |
| Family | Peptococcaceae | 73.12% | 88d1eb11dfd23669a5ac451a7e890e80 |
| Family | Peptococcaceae | 72.50% | 06489532c4f0b43d48b28067a0c4797a |
| Family | Peptococcaceae | 98.34% | 7ff6b91899f4a1138fe6df265a8818e5 |
| Family | Peptococcaceae | 86.58% | f714c699049f6d3ae4bcdda5a3329ccb |
| Family | Peptococcaceae | 84.50% | ba44ddc52d54f208f614bfe8b90fe668 |
| Family | Peptococcaceae | 99.97% | df1ea15de0618cadfd70dc1c034ef3fe |
| Family | Peptococcaceae | 72.40% | bce8a326b936ac019fe40bd7e77101c9 |
| Family | Peptococcaceae | 70.29% | 108223fe5a058ae7771e18289a859c62 |
| Family | Peptococcaceae | 99.98% | da574b2e552db7e864f4c3252e5ef506 |
| Family | Peptococcaceae | 99.98% | 28c202ba008a471cb0b079c40ce5de88 |
| Family | Peptococcaceae | 99.98% | c4135bd6bd4e3ea7cafae6999a5fd62f |
| Family | Peptococcaceae | 99.98% | 1f44f4adac6b13a1e0b7673047751d6a |
| Family | Peptococcaceae | 99.98% | f4e25a89e75a946143ae954354a3a00c |
| Family | Peptococcaceae | 99.87% | bd19b68b832398635e4bf3ddaa641eeb |
| Family | Peptococcaceae | 79.39% | 04079eed6def7386769d4316582f4768 |
| Family | Peptococcaceae | 99.98% | 9241ac195705bad14c8010e7708a1970 |
| Family | Peptostreptococcaceae | 100.00% | 71bcdf60231ce073a75bef627fd22d76 |
| Family | Peptostreptococcaceae | 100.00% | f0a1cb6bedd975fdab9c5231e8c6543f |
| Family | Peptostreptococcaceae | 99.99% | 2aaeaf26982b1c63b638314bdcd2d74d |
| Family | Peptostreptococcaceae | 99.58% | 9e358001b249ae7263d1f7572a359f9e |
| Family | Peptostreptococcaceae | 100.00% | e75fceebdcec36c7f3453e2659af4523 |
| Family | Planococcaceae | 85.42% | 12f99be43441df0439fb04a2f0b156ff |
| Family | Planococcaceae | 85.44% | d95f0c10d1b2bae7c220e09543af75d6 |
| Family | Pseudonocardiaceae | 70.33% | 0767027e344d91ef788d55daebbdaf72 |
| Family | Puniceicoccaceae | 88.78% | bb17b6e45ad92595a8c0fcd887c66024 |
| Family | Puniceicoccaceae | 99.26% | 68ba4def96984f814c9423a7c058e080 |
| Family | Puniceicoccaceae | 100.00% | 102e560a2ef4499fdb6bb8ec34bf5429 |
| Family | Puniceicoccaceae | 97.49% | 446a94dbc3e35ce5c712644c74978043 |
| Family | Puniceicoccaceae | 100.00% | 5e3e9ffd07d972507230ce8974171da8 |
| Family | Puniceicoccaceae | 97.42% | 893fe2b2a9fc60197ad57622c82d5f58 |
| Family | Puniceicoccaceae | 100.00% | a30f99acee8fcd6a814bec19a747be20 |
| Family | Puniceicoccaceae | 76.08% | 694515e291e8eb824bcaab07a680240f |
| Family | Puniceicoccaceae | 100.00% | 5aae07687155dc4d56ac509eeab683c3 |
| Family | Puniceicoccaceae | 100.00% | 25924be0318e1183755adf775910af05 |
| Family | Puniceicoccaceae | 99.91% | 11d2d1b0cba572a7433e96e38eb2d317 |
| Family | Puniceicoccaceae | 99.99% | 8284ecfc88ff5912be7cdd345c657682 |
| Family | Puniceicoccaceae | 99.91% | f4b92815f639ae4bca8fbd0be3a17fc1 |
| Family | Puniceicoccaceae | 93.10% | 875f9ee2d77ec2f6972a0b2109c4c68f |
| Family | Puniceicoccaceae | 100.00% | 5e446fa5c7172fd8e8658ed33db362bd |
| Family | Puniceicoccaceae | 100.00% | 183e73f356f668ee67d20d7b56940ed6 |
| Family | Puniceicoccaceae | 94.34% | 5cacf2d3a34245b082c87ff720490e1a |
| Family | Rhizobiaceae | 99.88% | 993aa3a1c834424bfac0e34b653dc3ea |
| Family | Rikenellaceae | 74.05% | 0f605baca874fabc497419de6e499493 |
| Family | Rikenellaceae | 83.03% | f6f7e37895b95637c9d2a07d92306589 |
| Family | Rikenellaceae | 88.65% | 907ec41034a6c884db4f39642a4b0874 |
| Family | Rikenellaceae | 72.93% | d4fb490a30ad56089ee5e33ab124fb7d |
| Family | Rikenellaceae | 75.31% | 041b7f014518861b870903de969b4d8a |
| Family | Rikenellaceae | 77.50% | aaf127339f21f12a1c2c2799973979c2 |
| Family | Rikenellaceae | 74.38% | 587d4dd35e0b88c4adea7e724c6c0c1e |
| Family | Rikenellaceae | 75.62% | b5be2f7b840662c450d32275e6140fb8 |
| Family | Rikenellaceae | 98.60% | d9177aeb77e6f3cfad334a5676fa03c5 |
| Family | Rikenellaceae | 74.97% | bb7cfd3b88ec2a79dcdaf93462c12448 |
| Family | Rikenellaceae | 74.65% | fb90b4f40912576d4cee7dc1ab141d69 |
| Family | Rikenellaceae | 99.46% | 902f9a738e95977669a68f647a3ec709 |
| Family | Rikenellaceae | 74.46% | c483985acf174fc14898c7ab08a4ae45 |
| Family | Rikenellaceae | 75.27% | 3e0861d7e0b7c3acb92037edcc7365c0 |
| Family | Rikenellaceae | 92.82% | 34b13fc225a42edb13ab1d86ab6d6801 |
| Family | Rikenellaceae | 99.63% | 0c5b9514c75df5c943ab681b20518a97 |
| Family | Rikenellaceae | 73.52% | efc101b3fcfddf5ac263fa0e4db21a4c |
| Family | Rikenellaceae | 70.33% | 0350ac44914d93a1eded2fe380a45306 |
| Family | Rs-E47 termite group | 93.63% | 5137c1e642e7915b1802af820c4e33cb |
| Family | Ruminococcaceae | 79.11% | 04ace6b2c4c6a81993939e4405af4dd7 |
| Family | Ruminococcaceae | 98.03% | 3a4ebfa07a50d6cf3ccde6bfc41e12ba |
| Family | Ruminococcaceae | 99.29% | a07ab1dbe1a9165773d5400f72c34db8 |
| Family | Ruminococcaceae | 99.67% | c12bef97797fef6499bec822aea4f8da |
| Family | Ruminococcaceae | 97.83% | 5a3841a1152d5429a057fccbdf9456f4 |
| Family | Ruminococcaceae | 97.89% | b4d6ba534c38e3d6801deb274c1bda1c |
| Family | Ruminococcaceae | 81.78% | dd8cfe578ebbd47f45b9364eea4a292a |
| Family | Ruminococcaceae | 90.31% | 6cd024ccd4fcc8e63a5d1d1fa5ced992 |
| Family | Ruminococcaceae | 91.29% | 1712aff7ab732908097f3c46c64f6517 |
| Family | Ruminococcaceae | 93.58% | 4710dbc0e951458edf451ade668fc00d |
| Family | Ruminococcaceae | 80.47% | 538291ae19911a4f1e8b9c3b02082c82 |
| Family | Ruminococcaceae | 79.55% | aa42b549c5d118cee9a82451091d9bb4 |
| Family | Ruminococcaceae | 91.86% | 559a9ae316ba7cf4f976bc3658942029 |
| Family | Ruminococcaceae | 94.77% | 40127817008bd75b3c1433118f15df5b |
| Family | Ruminococcaceae | 91.35% | 3844b05c2006bab986d0415cd52aa548 |
| Family | Ruminococcaceae | 89.83% | 1a307a4f2c5df3a4296cc2e095e62e94 |
| Family | Ruminococcaceae | 91.27% | 16992ccfd38f23e59cb6db435843f21f |
| Family | Ruminococcaceae | 82.96% | ae146f30e3c257ff19fee623733eeef0 |
| Family | Ruminococcaceae | 93.26% | e683eacba855bdc71ee9a50831859b42 |
| Family | Ruminococcaceae | 92.46% | 076223f58870d3b5192eafe3c4af9cf3 |
| Family | Ruminococcaceae | 89.73% | 588a1c186b192915139ac891163f900e |
| Family | Ruminococcaceae | 85.15% | 97bc262885fb4b9503889c2721ca8169 |
| Family | Ruminococcaceae | 94.80% | 95059e299c0f71a75e432dedd56d0a0a |
| Family | Ruminococcaceae | 92.54% | f6fd3aa21cf454859e579eb601a98518 |
| Family | Ruminococcaceae | 93.40% | cd1ad0c1d6a32e06f22cbd4a082880bc |
| Family | Ruminococcaceae | 98.22% | 1eac03c2f2208a25cde1fc009c657ecc |
| Family | Ruminococcaceae | 98.24% | abfb6bc86d7a8c82eab6bdffe5a3fef1 |
| Family | Ruminococcaceae | 89.75% | c6764c51ca1f3efc955ae52f6ec432bd |
| Family | Ruminococcaceae | 85.24% | f80383533f306f097c2b82d5b9104132 |
| Family | Ruminococcaceae | 95.16% | b720fea82700df64e1e5fd6a5a69cf29 |
| Family | Ruminococcaceae | 81.39% | 14c17374014f7a6ffab258e16f3abbba |
| Family | Ruminococcaceae | 90.55% | 66334add9efee2a7c55c194b66b32ebf |
| Family | Ruminococcaceae | 91.31% | cd258163ab3830372d7853c6b4099e5e |
| Family | Ruminococcaceae | 92.39% | f47e8cfb0bfd213c4e1dd507bdcf5f5c |
| Family | Ruminococcaceae | 88.40% | 224b72579351370be5af08d682cc5cba |
| Family | Ruminococcaceae | 86.12% | b00b3c7b11e193bbb036b95b93aa4a7e |
| Family | Ruminococcaceae | 99.95% | c8b058816ec88bc7e6db301117ba3405 |
| Family | Ruminococcaceae | 98.22% | ba1cdc0cc5519035bbe17a1c35da6326 |
| Family | Ruminococcaceae | 95.29% | 4f5a94e9b1a5413b28a4286b674391d7 |
| Family | Ruminococcaceae | 93.78% | 3f34d331f44c72c0d13430a9386ba865 |
| Family | Ruminococcaceae | 91.37% | bb67554adf9236be5f5a1531be51771a |
| Family | Ruminococcaceae | 91.13% | a5ab19bf5156af23f38f92402af2ace9 |
| Family | Ruminococcaceae | 94.56% | bcd1398a420a8f75a44f1663e9f80567 |
| Family | Ruminococcaceae | 90.93% | 91197051215bb8be6dfd47a157ff9b1e |
| Family | Ruminococcaceae | 90.21% | b3d9f7b3f5cec84f7a91d62e34561ef2 |
| Family | Ruminococcaceae | 80.40% | 6a876e79195c6b73c5d23c0bf04a4c78 |
| Family | Ruminococcaceae | 90.29% | 3d0bf1cbe58f8806ac3ba627b6045bc2 |
| Family | Ruminococcaceae | 91.67% | 8a98fd4454c5b1633bf38849eae8d5df |
| Family | Ruminococcaceae | 98.31% | efc0fdc0d725eaf235daaf00ab17c30d |
| Family | Ruminococcaceae | 90.28% | ac1f5e9bad49a8cadcb156b7f3c07469 |
| Family | Ruminococcaceae | 98.24% | 6bb68a20be1a41b9c13816becfb41200 |
| Family | Ruminococcaceae | 78.61% | 1785deb8af749d99ca09ebea4c344dc8 |
| Family | Ruminococcaceae | 91.13% | 9afe3264c2d371a505f692b0fffa079a |
| Family | Ruminococcaceae | 89.65% | 00c00d51ea61fde36e0f80207b0f208e |
| Family | Ruminococcaceae | 90.79% | b289f2f0595d7341d54a5a68373c99a5 |
| Family | Ruminococcaceae | 90.25% | 7c083abed1c0b870c6c2e1dc9b2e53c1 |
| Family | Ruminococcaceae | 85.02% | 0158970abccd4f06e907e566dcc91d59 |
| Family | Ruminococcaceae | 70.68% | 652924c24d2b3ade476a7c37778b0cab |
| Family | Ruminococcaceae | 99.25% | 46beb8476672effa6c3bf1f27cd961f3 |
| Family | Ruminococcaceae | 94.73% | 49d0891c3a7170d260d44a6ce8ec58fd |
| Family | Ruminococcaceae | 93.05% | 2ad879db1e330642dc46af14272a79c3 |
| Family | Ruminococcaceae | 92.40% | b1636fb3312c2620c51b4890c4ff3858 |
| Family | Ruminococcaceae | 93.10% | 05bcc7266a67f8f0449eb65b1bc254d4 |
| Family | Ruminococcaceae | 82.70% | b6ba4c0d651cdd92aae469ce727aad48 |
| Family | Ruminococcaceae | 81.41% | 782fad27689b0458f09ff1edfef7ade1 |
| Family | Ruminococcaceae | 93.64% | a1761f3b6fd3b9cd12771a022c42d406 |
| Family | Ruminococcaceae | 92.95% | e9c5a944e4d6f11b48b88e25b528215e |
| Family | Ruminococcaceae | 89.67% | 98e734dea4540ec0653309af0c2081a5 |
| Family | Ruminococcaceae | 99.28% | 36af4b2bc7f5da19efb8451a82c1b8bb |
| Family | Ruminococcaceae | 98.52% | d1c7b59e6ce4eef411ca4da46bc8ca49 |
| Family | Ruminococcaceae | 81.39% | 97c037ed28f622db7fc46985eb91cecf |
| Family | Ruminococcaceae | 92.27% | 077e5dc6b32f59d2b6d6a79beb61680b |
| Family | Ruminococcaceae | 85.82% | 7e82f29fd803b30e62216fa0d76b4fe7 |
| Family | Ruminococcaceae | 99.68% | 039466a05a8373ca09e95382e5272e17 |
| Family | Ruminococcaceae | 89.91% | 39930c75bc858c7cb1a671260072e623 |
| Family | Ruminococcaceae | 80.62% | f1a9a9304d115fae2d05aa1a3736e83e |
| Family | Ruminococcaceae | 87.67% | 8d0995687b6075d3801c861a79adf6b5 |
| Family | Ruminococcaceae | 91.53% | 81389cd2bbeb53f720a1b8562b5fa066 |
| Family | Ruminococcaceae | 90.99% | 0ff2cdb2eec80e73b009b4fcee1a3da7 |
| Family | Ruminococcaceae | 80.11% | 09549d9083972bfb0ca973eab8fa35d5 |
| Family | Ruminococcaceae | 97.91% | d9e2fa300fa5f1339764c5ebdfe8ed47 |
| Family | Ruminococcaceae | 90.09% | 3007030fa6149bbef6323163895ae92d |
| Family | Ruminococcaceae | 95.10% | b874571b91441213f021a3ca37b6b4fa |
| Family | Ruminococcaceae | 94.70% | 7b5761bf5df7a91e66667d84144b71f5 |
| Family | Ruminococcaceae | 81.73% | 48bfc23672122ee02bf2ec3f18a73406 |
| Family | Ruminococcaceae | 87.49% | e580e56abbb99eb4bd3d6fb2d6a1cc97 |
| Family | Ruminococcaceae | 95.34% | e88d1431e4d139c253056150c9231131 |
| Family | Ruminococcaceae | 97.97% | 0ae3df2a83afbc7eb85c1a4f52b77b3d |
| Family | Ruminococcaceae | 92.24% | 990f8d1fbacffddeec198133243b525c |
| Family | Ruminococcaceae | 82.92% | acdf3c8d2ac7dd981f1c845acd77737f |
| Family | Ruminococcaceae | 87.30% | a823ddd12783bc030e27185e0204a3fb |
| Family | Ruminococcaceae | 80.46% | 51ad18217b51ddff513b91ee2be65068 |
| Family | Ruminococcaceae | 99.74% | b251a2bf53e37c7fd1e143236fcc2e97 |
| Family | Ruminococcaceae | 97.69% | 50bfaab5bb53100a763fef628f39b361 |
| Family | Ruminococcaceae | 91.24% | 4a2494e93eb2ac3ae770711f06d72857 |
| Family | Ruminococcaceae | 91.77% | 0bccb2327d502d37ec608baa03eeadd7 |
| Family | Ruminococcaceae | 82.52% | a49f5c4ceef0f81d1d5f97d606ee9511 |
| Family | Ruminococcaceae | 96.91% | 07100ee99163aa1a6c517f6d4ba05b66 |
| Family | Ruminococcaceae | 99.95% | fde53203521b9a45fe8ac796ff8d17fe |
| Family | Ruminococcaceae | 99.96% | f044400c198f3eb584c83914c8c58553 |
| Family | Ruminococcaceae | 83.59% | dbd6632dbaf4fc9baa2253be6a3599b0 |
| Family | Ruminococcaceae | 93.57% | c6cad72921f51ec963eed7ddefde6576 |
| Family | Ruminococcaceae | 80.47% | cf692ed38441c675abf397f48af9948b |
| Family | Ruminococcaceae | 93.36% | bf2cf06b49174ade4e69e709fef87b82 |
| Family | Ruminococcaceae | 90.16% | e3f7a88d01bcd3a3d08e07883f2c2edc |
| Family | Ruminococcaceae | 82.01% | e793dcc2395781812b8c0d04e075b89d |
| Family | Ruminococcaceae | 93.59% | febb03f8657ca8ce771a57553f6cfa29 |
| Family | Ruminococcaceae | 92.82% | ec544725d6e7093c0934b8c4bd96edcd |
| Family | Ruminococcaceae | 91.43% | 590b797f29952c150d547f601cf4cf28 |
| Family | Ruminococcaceae | 77.56% | 172b30a476f7b222b874d7243623d52e |
| Family | Ruminococcaceae | 93.23% | b767baad05dde77ac64ff4d76bf1e2bb |
| Family | Ruminococcaceae | 99.56% | 0c286d525e1c4620dad1b09eb6eef64c |
| Family | Ruminococcaceae | 92.10% | cab28642e8f79499c55d68b642e2880a |
| Family | Ruminococcaceae | 85.56% | b9f7355e346491744bc1002f5b37f797 |
| Family | Ruminococcaceae | 100.00% | f534166ff7fac3bb9fded26e3eb44fe4 |
| Family | Ruminococcaceae | 82.03% | 77ebbfc982ff9ed5412d3956f874dabf |
| Family | Ruminococcaceae | 81.43% | 4e65bd0c22629e8f23ffe09d2da13577 |
| Family | Ruminococcaceae | 94.46% | c08561c7b85334e64ae869a03b68bc72 |
| Family | Ruminococcaceae | 93.15% | 93efd139297f564cc9dfcdd8161a9bda |
| Family | Ruminococcaceae | 90.83% | 13f9aef0760c6866782f31be6a71859c |
| Family | Ruminococcaceae | 90.60% | 27996c4f36a0059c388922982bee931e |
| Family | Ruminococcaceae | 89.33% | e6492246dd7f06a5d99674512c67df95 |
| Family | Ruminococcaceae | 99.96% | d3e35ff02e006f63265853c3a756a000 |
| Family | Ruminococcaceae | 97.16% | d2fce324c3a57aa9e252efd7321fe98e |
| Family | Ruminococcaceae | 95.35% | ab9a0c3c7071ef3e4480f48c674234b2 |
| Family | Ruminococcaceae | 81.10% | d0e07f2f52d91c241f91f39ee34bd28c |
| Family | Ruminococcaceae | 93.17% | 815c22174181c58a0407dc751bc6873d |
| Family | Ruminococcaceae | 82.16% | 5fb4ca64d6ed90511c0ff7ad9dd7dd9d |
| Family | Ruminococcaceae | 98.47% | 2aeb8f9a260c16b3492d2518158be016 |
| Family | Ruminococcaceae | 79.06% | 4765a95f13689435ea40222ef34a9d9e |
| Family | Ruminococcaceae | 93.57% | d362df02b225460dbdc3475636c2ac62 |
| Family | Ruminococcaceae | 97.67% | e0d2751ad33e011e11b13f2073d0552d |
| Family | Ruminococcaceae | 92.52% | 32b7048644657c4da9ec9b4f17cc5d56 |
| Family | Ruminococcaceae | 90.55% | 73bd109c143d36118a2b826b11f38ddc |
| Family | Ruminococcaceae | 90.46% | 3b53a510885d935e592cbe2ca5622a5e |
| Family | Ruminococcaceae | 94.79% | 62b81b201c2848b3aa059dff6bc614f3 |
| Family | Ruminococcaceae | 89.85% | 64ff9335956bab5a02c8e09351d9d3aa |
| Family | Ruminococcaceae | 98.54% | 421e22dfa8d17332ecef58ecfda19930 |
| Family | Ruminococcaceae | 89.80% | 960c356d0cb2023fe8c6cd290a65bff6 |
| Family | Ruminococcaceae | 94.70% | 6336575b6566774390b56d7facc90afd |
| Family | Ruminococcaceae | 91.84% | b39b5667fbf8337c1e6530fc1fd022c3 |
| Family | Ruminococcaceae | 97.85% | 12423ea79dc0ea1dbff2c659fc19cfb9 |
| Family | Ruminococcaceae | 94.24% | 06376fd0000eb803fd7a8a5c6ef56a1a |
| Family | Ruminococcaceae | 84.96% | 4d381a576d9adbf154ff9dc5786fd2dd |
| Family | Ruminococcaceae | 78.30% | 4bdb6aa2b2b232b76087d1a56492830d |
| Family | Ruminococcaceae | 99.93% | b3d12bcf53999e2ba4fa83d38b636497 |
| Family | Ruminococcaceae | 96.80% | 25f022fc618c6bb12de7434237c184bd |
| Family | Ruminococcaceae | 90.54% | 4a9435304e36dda357f1a8fd9809d5cd |
| Family | Ruminococcaceae | 89.02% | 9238b71b4b65030033bc01e08f4eea15 |
| Family | Ruminococcaceae | 93.47% | 6fb46a915e9389bdab7a589016459c62 |
| Family | Ruminococcaceae | 97.47% | 65097a64001f8c05f761a7108b52e7aa |
| Family | Ruminococcaceae | 87.70% | 696e8195401a7ac0d8661736ff9b4520 |
| Family | Ruminococcaceae | 83.83% | 63c67be6302f3cbacb1c3565df7fad30 |
| Family | Ruminococcaceae | 97.47% | 41597ec3dbe1c695917535ec76c8b0aa |
| Family | Ruminococcaceae | 84.63% | 8d2c2816b543144a863a328b57d8a873 |
| Family | Ruminococcaceae | 79.46% | 3b82b8920aa798025a88d0a39d3ff53c |
| Family | Ruminococcaceae | 92.74% | 9ea4d2e45b04d0951f76b94135fa666a |
| Family | Ruminococcaceae | 91.05% | 63e840056d09b1c5a4436056108ef426 |
| Family | Ruminococcaceae | 99.29% | 681d92dd2fb51613bec65249f314b1c9 |
| Family | Ruminococcaceae | 94.74% | 1ba111659f2096f1912f835076693582 |
| Family | Ruminococcaceae | 99.99% | e4e1acbaafeda1307d38184e27822016 |
| Family | Ruminococcaceae | 99.91% | 51f0c328c47bacfd4cece00c9118a644 |
| Family | Ruminococcaceae | 95.81% | 2dc9ee2446bf720f5b231c9e793ec886 |
| Family | Ruminococcaceae | 82.50% | dbe27715eb23f225600a1d3b4c6d8a4d |
| Family | Ruminococcaceae | 94.99% | 725837f0b26217931b1060a48690be98 |
| Family | Ruminococcaceae | 92.50% | 6c3ab903a5e5f41110dfb8afcf72856c |
| Family | Ruminococcaceae | 86.39% | 376494fad67dd14cd649e07d8d7ffdde |
| Family | Ruminococcaceae | 100.00% | a6d0240ed46227c79c827741134ea833 |
| Family | Ruminococcaceae | 81.78% | b61d6b5b39b4669f84652b968d32c738 |
| Family | Ruminococcaceae | 97.48% | d7bf1765c7aa89c4c977a5facb6ec579 |
| Family | Ruminococcaceae | 94.33% | 62a9598d4efc978a8adc18bc041e7ef2 |
| Family | Ruminococcaceae | 90.56% | b59b2651a84b828c081a456cd8fe88d9 |
| Family | Ruminococcaceae | 90.31% | 835e8a716d1497cd57c409ffe465d0ed |
| Family | Ruminococcaceae | 90.81% | 0ba84a90e304894b06d5f36ee578df2e |
| Family | Ruminococcaceae | 81.00% | d33ee7148b6b17e56e1643ebbdf1b1f2 |
| Family | Ruminococcaceae | 88.81% | 416a75aff23b15410daed14ef8186aed |
| Family | Ruminococcaceae | 89.28% | 6da5c5ba55a336097965ca58ee100b30 |
| Family | Ruminococcaceae | 93.98% | e03a8ad6f4269c72c2a90abbf84561da |
| Family | Ruminococcaceae | 96.35% | 6738a10af49fa1146a25b7469180ed2e |
| Family | Ruminococcaceae | 83.73% | 9b8e7a34af3c82d3133c09f1c30705c6 |
| Family | Ruminococcaceae | 93.11% | 733f843b1e582fd620035cacdf6f4010 |
| Family | Ruminococcaceae | 95.57% | 11b4b535d1f04c22fdad4aeae464146a |
| Family | Ruminococcaceae | 92.92% | cc79b8481a3905a6f3e1cb591bd8425f |
| Family | Ruminococcaceae | 82.73% | 828e231690942e8a11c04774d647e8f2 |
| Family | Ruminococcaceae | 74.86% | 79193768f8501cd05cb7043a65f90298 |
| Family | Ruminococcaceae | 75.02% | cae38e097988c5fe69f109770c31c9ef |
| Family | Ruminococcaceae | 80.28% | e72e071ecb9420e26b36b3a4ff4732fa |
| Family | Ruminococcaceae | 86.09% | a3d572425f9d29b0bb9fced16380eff5 |
| Family | Ruminococcaceae | 98.57% | 8d169169f914a373c77cde1a27bb081f |
| Family | Ruminococcaceae | 74.28% | 31a04679aef89af0847533bb117e7d02 |
| Family | Ruminococcaceae | 79.56% | 836f455dd648c451808821d225a803ad |
| Family | Ruminococcaceae | 74.09% | 0e7bde9bb5332cf130d523c0b11e5ca6 |
| Family | Ruminococcaceae | 79.71% | 0ecf528a45b3ddf0f950741b0f6be6ba |
| Family | Ruminococcaceae | 73.40% | 4af0cb275a7025dfa892e83e63944da8 |
| Family | Ruminococcaceae | 79.70% | 2676db3b6af7a1e128750cc86f8aa8c4 |
| Family | Ruminococcaceae | 74.09% | 356a9ff182cc9003488e02e061f71188 |
| Family | Ruminococcaceae | 79.86% | 3dae13be7c7d3667442462a5c8d44b7f |
| Family | Ruminococcaceae | 98.27% | 3c569dc6163d3baa42e262c986ad75dc |
| Family | Ruminococcaceae | 96.93% | 2a67fef7e6ecb2a01f1dea60ef8d5c89 |
| Family | Ruminococcaceae | 71.29% | 49ed04e0469f68035bba112a8b36db04 |
| Family | Ruminococcaceae | 85.68% | 6fc1e7b499bc33b883a59a45e06a3037 |
| Family | Ruminococcaceae | 79.82% | 82b5ef9af32bdc9b23cc750221e258b7 |
| Family | Ruminococcaceae | 98.59% | a6aeb84890f0d7ad87674a8a9610abde |
| Family | Ruminococcaceae | 86.61% | 6a82ae6e8ca0da378a5ab937e97836ae |
| Family | Ruminococcaceae | 85.81% | 3b50035af83f9348bf1a8c6cd6772830 |
| Family | Ruminococcaceae | 73.76% | e2799a43defed9377797650b3feeb081 |
| Family | Ruminococcaceae | 99.93% | b544d1a9206588afa3b7ad1f8b8f42a5 |
| Family | Ruminococcaceae | 98.57% | 2c2565f20475b0c614e8441ab324231f |
| Family | Ruminococcaceae | 98.71% | fd528e6b387e2db8a86a9054d5bf8dce |
| Family | Ruminococcaceae | 97.56% | b9abca38c266cc9c5f974331bb6eb210 |
| Family | Ruminococcaceae | 98.20% | 2783afbb474dc74bff62d98dbcd0cd95 |
| Family | Ruminococcaceae | 86.44% | 57337050b2ca05e3aecd396c51512538 |
| Family | Ruminococcaceae | 97.60% | 4b7ae8cc023f77f37a5f86c630d629e3 |
| Family | Ruminococcaceae | 96.53% | 2f101e7a434cdb8f9b83c5b54b52e7f9 |
| Family | Ruminococcaceae | 94.17% | 778423e44f9364a200eb3ea5e484f31c |
| Family | Ruminococcaceae | 75.88% | cc09e2ddea994919e03175348f5571dc |
| Family | Ruminococcaceae | 82.08% | 71e2cb5c4bca5b1fce89fb50d1480e6e |
| Family | Ruminococcaceae | 71.74% | dfa7d948cd6f612913214c08138b6749 |
| Family | Ruminococcaceae | 75.08% | eb5ffab4ecc63a8d4b9b4dfa2cf651cf |
| Family | Ruminococcaceae | 74.34% | 691eda481f42fd3a903181207f1850ca |
| Family | Ruminococcaceae | 97.57% | 99613dd14da445979a74f7be1d447b80 |
| Family | Ruminococcaceae | 81.64% | d64850ab9022c8ca90ab0d66bb32de77 |
| Family | Ruminococcaceae | 93.55% | a4f0c20763503bff5374f83686230e37 |
| Family | Ruminococcaceae | 99.76% | 37586d63d9473bc3defc52ac69a90c99 |
| Family | Ruminococcaceae | 75.92% | e73d25acd2dc79ff85b8a32449d643fe |
| Family | Ruminococcaceae | 74.54% | 7b239f382bf3f3c9f5169eda52c49b4a |
| Family | Saccharimonadaceae | 79.71% | ef4f4c4a7d2fd48bf52ba1d47fb0ff20 |
| Family | Selenomonadaceae | 95.54% | f79ed8e37a225b0a5c4428f3b7997438 |
| Family | Selenomonadaceae | 90.45% | d3c3585d4f79ccc215a6ee8f86580a93 |
| Family | Selenomonadaceae | 90.37% | dd856e0e7d0036c70f917951e3ba3f94 |
| Family | Selenomonadaceae | 91.06% | fe387fddd487edd2bc154dfe3c7463cd |
| Family | Selenomonadaceae | 94.97% | 718120cebc81fe3fd87d34b98aa93217 |
| Family | Selenomonadaceae | 84.77% | dc1246c2e25161bac71c69f24a68165c |
| Family | Selenomonadaceae | 86.21% | 08bcaf641cb944296223dec411ee929b |
| Family | Selenomonadaceae | 94.00% | 6186caa97d5e1d0954df47034a145d03 |
| Family | Selenomonadaceae | 87.20% | 10a7895fe0c957314ae2049f93018213 |
| Family | Selenomonadaceae | 86.14% | 12cc267bef594c53e0076b8b1757ee36 |
| Family | Selenomonadaceae | 86.21% | 078e7d1a80a77e204ebe9607e9b7996c |
| Family | Selenomonadaceae | 93.55% | 77061ebf27c58b85ab59fc3e55a58d11 |
| Family | Selenomonadaceae | 86.22% | 9e8c38adc9ad9fc990a85b79470fe871 |
| Family | Selenomonadaceae | 94.08% | 5cd21514b2a3f134a3b628b6c99174f1 |
| Family | Selenomonadaceae | 87.67% | 51f21036ca136673f2482399367ec4b6 |
| Family | Selenomonadaceae | 93.97% | 840f8d554d520c259a576ccfdc0725ba |
| Family | Selenomonadaceae | 87.68% | 0479c961cbbea33f9f12b0862e847f87 |
| Family | Selenomonadaceae | 86.21% | 0446cac5b9e9436dff3ed5d78580aacc |
| Family | Selenomonadaceae | 81.30% | 8b2355aa743cd079ce1c4a394a205224 |
| Family | Selenomonadaceae | 93.74% | 7971d171f686a444b6930a47f2099f10 |
| Family | Selenomonadaceae | 85.23% | 6e9f59556c6bdbd711386d0e5eb4b268 |
| Family | Solirubrobacteraceae | 95.55% | 7f62732162f07334dace758a91c6fc59 |
| Family | Tannerellaceae | 99.94% | db785240bf02d3dee8557f62eb0ae5a4 |
| Family | Tannerellaceae | 99.94% | f4295dd1c59da355068e93b17dc97966 |
| Family | Tannerellaceae | 99.94% | 52f79532987a8b54fa96c1f182f9328e |
| Family | Tannerellaceae | 99.95% | 043a04f489f300641946f0cfd303dc42 |
| Family | Tannerellaceae | 99.95% | fcf2a0b4af4ad5f1d4aa44239e8262e2 |
| Family | Tannerellaceae | 99.93% | 7a6dbf351a94d276d93405c605f210a2 |
| Family | Tannerellaceae | 70.42% | 153d8d8c6b1094bd46476e89d1d5d43c |
| Family | Tannerellaceae | 71.62% | 71c9cadeaeafa44efce3662160cd6589 |
| Family | Tannerellaceae | 99.93% | 337277f81335c044da0e4b467b98c508 |
| Family | Tannerellaceae | 71.07% | 07c71c858661c5787f3d4fcc543ce762 |
| Family | Tannerellaceae | 99.98% | 3900bb2186f74954ea90ff184577c9c7 |
| Family | Tannerellaceae | 99.98% | 015d856a596821f474e64e6a19c0bc5e |
| Family | Tannerellaceae | 99.98% | 0e42423989d888d20a6bade1957d5ea2 |
| Family | Tannerellaceae | 72.03% | 077b5deb6a9f2a536ec41f1e8e88a8e6 |
| Family | Tannerellaceae | 71.13% | 25faaa2e10911a34151ebd4da288f727 |
| Family | Tannerellaceae | 76.42% | b5a791774d727766dd7a3966cfad5d38 |
| Family | Weeksellaceae | 92.10% | 90acce5030dc9fb4130b80e0d538347b |
| Family | Weeksellaceae | 79.96% | f5cfa4c853d95aebb39f7ace5b468ff9 |
| Family | Williamwhitmaniaceae | 99.45% | d07eb22098ff036d398b92ebe9d1328c |
| Family | Williamwhitmaniaceae | 97.03% | 6b71f314a76b678b636e55c51e79226b |
| Family | Wohlfahrtiimonadaceae | 100.00% | 31c7ddeae4eff233d0e54e3216651b03 |
| Genus | Anaerorhabdus furcosa group | 74.09% | b89f8eb8b8ff7c9f00c4fb84d4909fa2 |
| Genus | Anaerorhabdus furcosa group | 74.09% | af1019eece6ea39b41c73b41ee792341 |
| Genus | Anaerorhabdus furcosa group | 92.06% | b98987f91cc90cebbd4b92cc7c70fba2 |
| Genus | Anaerorhabdus furcosa group | 70.55% | 1738c5b01366125bf6f699e444f0da3e |
| Genus | Anaerorhabdus furcosa group | 92.17% | ee8af59f2fc928cf2bcde594235d916c |
| Genus | Anaerorhabdus furcosa group | 74.08% | 80efe0f2feadf9262df087337ce44ddb |
| Genus | Anaerorhabdus furcosa group | 74.83% | b6bd7651e6fcbf9208361defae81f7a3 |
| Genus | Clostridium innocuum group | 99.85% | 286870adbe497d431354f5bfa5b7eed1 |
| Genus | Clostridium innocuum group | 99.90% | cd6e49e176be5b626e87dc83748213bc |
| Genus | Clostridium innocuum group | 99.97% | 4f9214d6c5ff0426c3da88d4e9fda917 |
| Genus | Clostridium innocuum group | 99.97% | ddd4f36f498faa4e87fce4ca1a770b10 |
| Genus | Clostridium innocuum group | 99.89% | a6a539cf24e718c8f9da07b32c20f1c6 |
| Genus | Clostridium innocuum group | 99.60% | eea15ec3764debbe68649ab1b0d42fa9 |
| Genus | Clostridium innocuum group | 99.97% | f1487b0ae9a9d228dc65d4adab57c3ca |
| Genus | Clostridium innocuum group | 99.85% | bbeb375ee81cc14a37ad8d12cdaa83ca |
| Genus | Clostridium innocuum group | 99.59% | d9c45f2c7aebad7410b266aca842e750 |
| Genus | Clostridium innocuum group | 99.62% | d9ed0289739ed272d370babd4fb09709 |
| Genus | Clostridium innocuum group | 99.98% | 1423299604d368825e1a804619d7a61f |
| Genus | Clostridium innocuum group | 99.96% | 596d4b062dfb79da588330832d3aa327 |
| Genus | Clostridium innocuum group | 99.98% | 92aa8ebf730545ad2558a9a05ccc9591 |
| Genus | Clostridium innocuum group | 98.64% | 0fcd714ff09b2a1757890cf5bfae7b45 |
| Genus | Clostridium innocuum group | 99.98% | f9319178f2f6c230661371e9a7e2272a |
| Genus | Clostridium innocuum group | 99.84% | dc1dad342bdd25610fff409a155f6ef0 |
| Genus | Clostridium innocuum group | 99.83% | 0dc00e2bcbf7dc17f448aebf62e626b8 |
| Genus | Clostridium innocuum group | 99.85% | 7fc40727c12cdd2a26943934f838ce38 |
| Genus | Clostridium innocuum group | 99.95% | 0314b21f56d44aeda8391198ff2626a7 |
| Genus | Clostridium innocuum group | 99.85% | b15919811d35419a2a463dfa1f71f2f0 |
| Genus | Clostridium innocuum group | 99.83% | c0ca0e94821c5b1637f19c5530746c86 |
| Genus | Clostridium innocuum group | 99.99% | 74ee6d249bda996b831e17404a837e8f |
| Genus | Clostridium innocuum group | 99.86% | 7bfad1b638ca3578e41b888ea9528421 |
| Genus | Clostridium innocuum group | 98.22% | a66aaf29f753966a8421d9bef732bbac |
| Genus | Clostridium innocuum group | 99.94% | 3daab112372284e24b519a06e0fcaa73 |
| Genus | Clostridium innocuum group | 99.96% | a0f540195b3cb9f243f4a7fbc774a267 |
| Genus | Clostridium innocuum group | 99.94% | 4c2ba9d777296db29b4098f2d6d0bd55 |
| Genus | Clostridium innocuum group | 99.45% | b645583326662f7f02445dd3edd86df7 |
| Genus | Clostridium innocuum group | 99.77% | 740b69768ac28946e54cef4597145a55 |
| Genus | Clostridium innocuum group | 99.79% | df12b808c6cfa4186ee38218fb3a32c9 |
| Genus | Clostridium innocuum group | 99.72% | f25f88987452270bb74b0f2a0b6f721d |
| Genus | Clostridium innocuum group | 99.86% | 9e127e94e28e645d51a4294187ce9277 |
| Genus | Clostridium innocuum group | 99.89% | 1dacf97518c6c34f09cbd662e1d5510e |
| Genus | Clostridium innocuum group | 83.68% | 7934b89e97699c3ea90171d60c7baac2 |
| Genus | Clostridium methylpentosum group | 75.59% | 50f11258d7fd75a931fc1f863d81948a |
| Genus | Eubacterium coprostanoligenes group | 99.90% | b3b3aacf5125d1cb1cbc313249b221dd |
| Genus | Eubacterium coprostanoligenes group | 99.70% | 7ae8e1b6e634dadfd82c1cd491c52ad9 |
| Genus | Eubacterium coprostanoligenes group | 98.63% | a31cead35b50dabf7377762dd926ca14 |
| Genus | Eubacterium coprostanoligenes group | 97.25% | 7e07d17d52c141b4ad286e535b646135 |
| Genus | Eubacterium coprostanoligenes group | 94.05% | 936d3869d0021b8de249a4af9ed51770 |
| Genus | Eubacterium coprostanoligenes group | 99.22% | 7b254941215d457f0db41e362df53597 |
| Genus | Eubacterium coprostanoligenes group | 99.89% | 1b48c6305d8da7c1dc4ef94c0a55b5dd |
| Genus | Eubacterium coprostanoligenes group | 99.84% | 230131ab0ff1f9b72f39aea6958abe59 |
| Genus | Eubacterium coprostanoligenes group | 99.86% | d981e8e11f6d8fdf5015a405f98226ad |
| Genus | Eubacterium coprostanoligenes group | 95.76% | 6b07667cb0d5cbb137aecb92c0a1bf26 |
| Genus | Eubacterium coprostanoligenes group | 99.90% | ca1677e96bc1979d9e4cbbc6946d8db4 |
| Genus | Eubacterium coprostanoligenes group | 99.53% | 679b415d130001f27c2ebc6f624531a3 |
| Genus | Eubacterium coprostanoligenes group | 99.62% | 3d8db95e7aaba933f1ec9c8d7f9b7469 |
| Genus | Eubacterium coprostanoligenes group | 99.81% | 813929abe2638680c4085432649378da |
| Genus | Eubacterium coprostanoligenes group | 99.56% | 13dc8f481e91f5a5492aad1be7adcb55 |
| Genus | Eubacterium coprostanoligenes group | 99.90% | db3ed92b3b416e84ab20d3e891d861e8 |
| Genus | Eubacterium coprostanoligenes group | 97.32% | 128d95b1470cf74895e9319272e7fc3f |
| Genus | Eubacterium coprostanoligenes group | 99.71% | 45664454a9ab5acb40bacb3eae2fde9f |
| Genus | Eubacterium coprostanoligenes group | 99.92% | d7970ff60edb2aa4928bfb90ed8f0dba |
| Genus | Eubacterium coprostanoligenes group | 99.75% | 4d16a49008c3a338a3e0a98d1a0a8d6a |
| Genus | Eubacterium coprostanoligenes group | 99.57% | b9e420e04a8508562441ff180619f5c4 |
| Genus | Eubacterium coprostanoligenes group | 99.88% | b2da2d2ffa031bc2c85e1e38458aa44c |
| Genus | Eubacterium coprostanoligenes group | 89.61% | 192e53cadfaffd0b19c982cd19773067 |
| Genus | Eubacterium coprostanoligenes group | 85.89% | 3f0055c7b9efe125dc8c924f35e7d270 |
| Genus | Eubacterium coprostanoligenes group | 72.49% | 7f69755bae72e2520c6143b3973419be |
| Genus | Eubacterium coprostanoligenes group | 99.89% | bf835c9e267ffb9bf48a26ea01e87cda |
| Genus | Eubacterium coprostanoligenes group | 99.58% | 12fd70d7fad9a0d1f6d1e757030aae9a |
| Genus | Eubacterium coprostanoligenes group | 76.97% | 46f26bb7a8b411f153808b670f5785a4 |
| Genus | Eubacterium coprostanoligenes group | 76.17% | 3ee9f92a7e25cd982e5e2292bb03c1a1 |
| Genus | Eubacterium coprostanoligenes group | 73.35% | 5f0e9159696eb9bb3ea6ffb3b5ffd340 |
| Genus | Eubacterium coprostanoligenes group | 98.95% | 41517e320afc1f85f2729b4d4d4d8603 |
| Genus | Eubacterium coprostanoligenes group | 91.70% | 866b58d2d056f6c2b73b52cfe279514b |
| Genus | Eubacterium coprostanoligenes group | 96.19% | 958ac5e5cdd11df571e8a90e9053d064 |
| Genus | Eubacterium coprostanoligenes group | 73.52% | ccd387604f96fd898ab170c2df23b222 |
| Genus | Eubacterium nodatum group | 81.73% | 3cb41c05cbaea86055d0ae71576bd441 |
| Genus | Eubacterium nodatum group | 99.16% | ec320aa09d595eec1038e5ce26ca35c1 |
| Genus | Eubacterium nodatum group | 94.77% | c44c5ddb64acda1397b683aff1928008 |
| Genus | Eubacterium nodatum group | 89.55% | a1560fc1f0ab39ef28b23c01ff81d86a |
| Genus | Eubacterium nodatum group | 93.47% | 2e0aa857e01d81e6e50650a39831a53d |
| Genus | Eubacterium nodatum group | 99.16% | 3039fa3580d091263a31cfa43fb6f61c |
| Genus | Eubacterium nodatum group | 99.16% | 84695519413054786a72a7f18bb094c2 |
| Genus | Eubacterium nodatum group | 99.15% | 7eec5814aea56e69a7825cd20fb78dda |
| Genus | Eubacterium nodatum group | 99.15% | f34a724c44c4cd1a1165e6c922f6837d |
| Genus | Eubacterium nodatum group | 98.11% | 8cfb246efed5612d8ebddf67d4e8baed |
| Genus | Eubacterium nodatum group | 98.81% | b895e425b61bc895b3fec22e4df1c3a6 |
| Genus | Eubacterium nodatum group | 98.99% | b5ccd9d1f2d7eb3fbb2f599897682742 |
| Genus | Eubacterium ventriosum group | 75.09% | 9cc84a71460a709ba64f0af80cdd0907 |
| Genus | Eubacterium ventriosum group | 81.20% | daab01453968767662c24826a3e4b4db |
| Genus | Eubacterium ventriosum group | 90.98% | 0e6e962f8af199027f756ff289fee840 |
| Genus | Eubacterium ventriosum group | 88.64% | ea8d76e6589b06c78bbb6d6712fe0a9f |
| Genus | Abditibacterium | 100.00% | 026e6c3b07eb2e88b5a02cdd52b0c69a |
| Genus | Acetanaerobacterium | 100.00% | 58ad055fefc53d0227d818fa5d31d9ae |
| Genus | Acinetobacter | 99.63% | 6803d90f6304b734928376a782de761c |
| Genus | Acinetobacter | 99.40% | cd03fcee5303fe0f150aeba86ae5309e |
| Genus | Actinomyces | 92.17% | f3311004edf11a12bd325be00a599e40 |
| Genus | Actinomyces | 98.16% | 9c06a2e0f803982be889535c5218d40a |
| Genus | Actinomyces | 96.34% | 64aac432c8cd85f485c764a1fa8cc40a |
| Genus | Actinomyces | 99.90% | e487e0d64364b88046fc13bc64d36b94 |
| Genus | Actinomyces | 99.72% | d3a56945bc754b02d6dd35004f9a0d17 |
| Genus | Actinomyces | 99.82% | 6110ea88242d22c8a0ae9be42c9772d0 |
| Genus | Akkermansia | 100.00% | f0b01249e540cab4e9e6cf40aa40a08a |
| Genus | Akkermansia | 100.00% | c93f2441390feae70ed195f5d43a6f71 |
| Genus | Akkermansia | 100.00% | 4b16b9026b1c98961ccf9f9e59b9776a |
| Genus | Akkermansia | 100.00% | 55937519dd63461953e41f1277503eee |
| Genus | Akkermansia | 100.00% | a1cb13c50fb88a1f48094941ff396cac |
| Genus | Akkermansia | 100.00% | 24fd2c030b07dbf8451bfa778c217133 |
| Genus | Akkermansia | 100.00% | 0da2008a66615d8ca378d981a812969e |
| Genus | Akkermansia | 100.00% | a4425f94b16dd5df2f9bb1ac47cb8ffa |
| Genus | Akkermansia | 100.00% | 91981056ee9dd52d71722c5088545d3f |
| Genus | Akkermansia | 100.00% | c9c2ba98bdb2c0d1a1b91fa8d2057fd4 |
| Genus | Akkermansia | 100.00% | 7216bc8d14efedc1a6b24081256d5468 |
| Genus | Akkermansia | 100.00% | 26cfec76369eb4c67a1edeeca24ba020 |
| Genus | Akkermansia | 97.54% | fa6b8366f7f4dbbd6623cd2d989dda2e |
| Genus | Akkermansia | 99.87% | 716d6b020484051507b1f6bbf4bedd19 |
| Genus | Akkermansia | 97.84% | b30317ad24e309fe0f4c74c6b3df7715 |
| Genus | Akkermansia | 95.26% | e3efa5607e87d6fc060b7a30ef9f9b7a |
| Genus | Akkermansia | 97.74% | cc473a5a3036ba27f85d77d03b613216 |
| Genus | Akkermansia | 95.61% | b59aa25b8500a900902c0b7abf97f021 |
| Genus | Akkermansia | 94.07% | 72e2556f75f63fee73d387e029a775d9 |
| Genus | Akkermansia | 93.25% | 73372f2e2e35426f946ee6f99d32cb5b |
| Genus | Akkermansia | 98.33% | 2f425192676af915fcfa9f166591ecf5 |
| Genus | Akkermansia | 93.30% | 9a10ec8961ae8bb5d5e9b9ab9ab62029 |
| Genus | Akkermansia | 98.39% | c4154429273fb032e12878b999691f89 |
| Genus | Akkermansia | 98.17% | a5a2dbd83e34920ab0b6fa9f08509216 |
| Genus | Alistipes | 100.00% | a63219e33219c1ac48c8c36e96a13f7e |
| Genus | Alistipes | 98.98% | 4d43dcf4cfe2cc51d9e7cdf0de4fa1e3 |
| Genus | Alistipes | 100.00% | 9e4e0a48e5c50dbe32acd73f26b73b64 |
| Genus | Alistipes | 100.00% | f546a77e7fc9cd8164a8406a424ef8b1 |
| Genus | Alistipes | 100.00% | 164d31c221e071410637b4b647c7f683 |
| Genus | Alistipes | 100.00% | f1eebf83544c7eb76e07ad84250dbe00 |
| Genus | Alistipes | 100.00% | 72efd7f2f185e18e8d25fd54829f0192 |
| Genus | Alistipes | 100.00% | 0e14ca93f0fa35d931f5bf43ec417b0e |
| Genus | Alistipes | 100.00% | 86cb9cf633279c79e39e91608b3231d2 |
| Genus | Alistipes | 100.00% | 24e0943a3992fead2626af849bf0d819 |
| Genus | Alistipes | 100.00% | 787ba537c5303d5c7db7cbbde026972c |
| Genus | Alistipes | 96.89% | 9c540fe0c89fcb22ace180fa48b60d5d |
| Genus | Alistipes | 100.00% | d50e48e919297a3f1a0118c5220cf410 |
| Genus | Alistipes | 99.96% | beb21b358f4a13d6a9fa3bd600c199b6 |
| Genus | Alistipes | 85.46% | 1aa3805bff269f2c62f44b9fa59b3f85 |
| Genus | Alistipes | 100.00% | 9890d81465ac9cdc2dba94830f485a4d |
| Genus | Alistipes | 100.00% | c6176c64913839e1582de697c971c8cf |
| Genus | Alistipes | 99.01% | a850dec06756dbbab8e92d43b52d0f88 |
| Genus | Alistipes | 99.03% | 2286fc91de917f7e3851e8a710c5e081 |
| Genus | Alistipes | 100.00% | d39ba831d7ff21a2bdbee7ddec7aff8e |
| Genus | Alistipes | 100.00% | 4cc4f2b9a405c0fecf9963557d5551b5 |
| Genus | Alistipes | 100.00% | ade6f1ebcc7a864fdc9094558ffc48be |
| Genus | Alistipes | 100.00% | fdc42c67f26eedb80ba778d9d0b50cda |
| Genus | Alistipes | 80.14% | f088daa0ac48cbe9bae9678f0fb3af03 |
| Genus | Alistipes | 100.00% | 51e38f07a5624c8c5898a94d1cda0522 |
| Genus | Alistipes | 95.10% | 91a7d56367d4a09c1fb8063f9ce0628b |
| Genus | Alistipes | 82.05% | 1e228bce8c342b4b97654305246415f5 |
| Genus | Alistipes | 74.53% | 8676a5c8e29936d7c613cb5b0a8e7414 |
| Genus | Amnipila | 93.79% | 6ad43b83f3a6625c78e05fd2ff57d327 |
| Genus | Anaerofustis | 99.51% | 944867a40951b7f555f96a1d842997d9 |
| Genus | Anaerofustis | 99.78% | ee77c9031e561f1853f745ddbfceb93e |
| Genus | Anaerofustis | 99.50% | be03d44b89ec50a6febb4db64923c5ce |
| Genus | Anaerofustis | 99.62% | 373e525841dc13ef3080ce4246718cd2 |
| Genus | Anaeroplasma | 93.13% | 7689fc6ce7dda355af2b3c524a9419d3 |
| Genus | Anaerostignum | 71.35% | 6ad9256cd998e49e62344c0239cb7bdf |
| Genus | Anaerostignum | 72.35% | 32b0bda1041f2eeb5efb083d58377ed0 |
| Genus | Anaerostignum | 71.05% | acbc2fa6f0df7b68bff28b39da0597ef |
| Genus | Anaerostignum | 71.50% | e26e09909071ecbc8cfe2a640495fe87 |
| Genus | Anaerostignum | 84.23% | ac6aae6f7d719f1020f1e324919ca79c |
| Genus | Anaerostignum | 89.58% | 83e21e6b9c9f411ae6018ea4bc82b00b |
| Genus | Arcobacter | 97.41% | 7a6651e6322d12961d0c22174cf03c5c |
| Genus | Bacillus | 97.13% | 232e1c63d801b0b6ecd3b9040998e66f |
| Genus | Bacteroides | 100.00% | c47a62ad84c85bb0effe321cdc6e57c2 |
| Genus | Bacteroides | 100.00% | 5b1b81e7d6cd8b3c13bf39bf2e94279b |
| Genus | Bacteroides | 100.00% | d908aaa995df1997879911ce1667ae37 |
| Genus | Bacteroides | 100.00% | a559319bfc804ec7695536d65a4c09b3 |
| Genus | Bacteroides | 100.00% | bf3ee3064b9e60bed9bf0d4cad6d59ec |
| Genus | Bacteroides | 100.00% | b036b77c48201a9f81c31c3aec26eeac |
| Genus | Bacteroides | 100.00% | f5604f9295f4fec4335470c70b48fd72 |
| Genus | Bacteroides | 100.00% | cf1c5aec9639ef1ddc8a8cddbdc7542b |
| Genus | Bacteroides | 100.00% | 2c2934afd158ed46f3020ccd73846a1a |
| Genus | Bacteroides | 100.00% | 411b992d89b1db59d97500bcc7827439 |
| Genus | Bacteroides | 100.00% | faa08827afe046a303182702b1b83759 |
| Genus | Bacteroides | 100.00% | 12823ee18edbc98357bc3e6a478b7801 |
| Genus | Bacteroides | 100.00% | 82c138affaacabf03be87c0bd6f52e1f |
| Genus | Bacteroides | 100.00% | 37997871ad8ba06686c021bb016254ae |
| Genus | Bacteroides | 100.00% | c19ea40adcd3aded858aea8ff41539f4 |
| Genus | Bacteroides | 100.00% | e9173847ae692a8c6735b7169198f417 |
| Genus | Bacteroides | 100.00% | 279ccc533996dfb0ac50b66c84454a01 |
| Genus | Bacteroides | 100.00% | ea6ca2d618a05270621341ccdbdf3298 |
| Genus | Bacteroides | 100.00% | 58c4a60f2baba11b201601ee74bbdcc4 |
| Genus | Bacteroides | 100.00% | 6690ca7b6a68d52c3f79577a087c1668 |
| Genus | Bacteroides | 100.00% | 1bb06ae80892843b47d98d9f14f411e2 |
| Genus | Bacteroides | 100.00% | 9a8374f1592ac2e450ac353e6d59ce5e |
| Genus | Bacteroides | 100.00% | d9e4605883bc7d14af1802fe630a97d7 |
| Genus | Bacteroides | 100.00% | 684a5180db2eef1749317eedf57a2fea |
| Genus | Bacteroides | 100.00% | d619436f8155d72a2ab215ac9c9150b7 |
| Genus | Bacteroides | 100.00% | 3897328da78c3f0185f79666eec8c2eb |
| Genus | Bacteroides | 100.00% | d96ee4ebcd458f092546c5b97755bb3b |
| Genus | Bacteroides | 100.00% | 7bfdc33804b8df9b39bee81a11d8ec17 |
| Genus | Bacteroides | 100.00% | e3e2bfab14d50802805c6fb2c2eb707b |
| Genus | Bacteroides | 100.00% | 20438aa1a92c0c92a3d37a77f08ffa34 |
| Genus | Bacteroides | 100.00% | 4e90253b236bc593925d4ffa4544adfd |
| Genus | Bacteroides | 100.00% | 96909c9d648a0f20dbc84c8fa81294a4 |
| Genus | Bacteroides | 100.00% | 919286e08b79b56410ee6de0dfcbd96a |
| Genus | Bacteroides | 99.97% | bc577efaacb59c655fb7bfad873fd22f |
| Genus | Bacteroides | 100.00% | fe8e73f6e0b49d5884361e6440ebdcdb |
| Genus | Bacteroides | 100.00% | 43e83175034660e53b012ce4c50f186b |
| Genus | Bacteroides | 100.00% | f89983ae436edeee649b737ecbbc90c7 |
| Genus | Bacteroides | 100.00% | b31166e62c3861d54ffef6a53adb75df |
| Genus | Bacteroides | 99.98% | 57f075657048f3d8094cc678a947db3e |
| Genus | Bacteroides | 100.00% | 9ab886bdb0c375acc0ed6c49d04bdd22 |
| Genus | Bacteroides | 100.00% | ea3bd58327e36b23a6d6e36e83314729 |
| Genus | Bacteroides | 100.00% | d72fbe07f2d4660b78556ebac22b8a0a |
| Genus | Bacteroides | 100.00% | d338e5f36d68161a9d88a80a621ab428 |
| Genus | Bacteroides | 100.00% | 38a2d9edebd2559abbb1c49ea0f52fc3 |
| Genus | Bacteroides | 100.00% | 3f58df2e90a44e94140e432dc21f1311 |
| Genus | Bacteroides | 100.00% | 56625c8f596484db63c60482372b3b3f |
| Genus | Bacteroides | 100.00% | 69def49dca710fc8b519c3652515d442 |
| Genus | Bacteroides | 100.00% | 4ebf6186262623cae9a365b27de94620 |
| Genus | Bacteroides | 100.00% | eda905e5483c24a1bc3b4157d5284225 |
| Genus | Bacteroides | 100.00% | 36a0b57a3762dac3eef335ca6151ea9d |
| Genus | Bacteroides | 100.00% | b4dfeddd1ade11d01af992285510d9b1 |
| Genus | Bacteroides | 99.97% | 461075275f9506dcd93d06065f85fdb5 |
| Genus | Bacteroides | 100.00% | ec3b597dba7012cbeefddaf6e6d18255 |
| Genus | Bacteroides | 100.00% | 8282cb34067421096baf09e2bf1e62d4 |
| Genus | Bacteroides | 100.00% | b7e29fd903d471f97a06e20fc7009f27 |
| Genus | Bacteroides | 100.00% | 8fd7f37d6176b8cf3bbba6ab096303c3 |
| Genus | Bacteroides | 100.00% | c0af867ef09305f44e5d304c614f2842 |
| Genus | Bacteroides | 100.00% | 3a6340331a7ebc550e810be7c92496be |
| Genus | Bacteroides | 99.99% | 2dd49490d6a24598e2341106ada671c2 |
| Genus | Bacteroides | 99.99% | a856e428685b7428e9cf7d710308cd73 |
| Genus | Bacteroides | 100.00% | b830eba4c0a88e6944fae3cafc416e19 |
| Genus | Bacteroides | 100.00% | 5f4bea4560a70f87b750a41b0e01f801 |
| Genus | Bacteroides | 99.99% | 2ffa1c5b88b3482afb37e0232b028b2b |
| Genus | Bacteroides | 100.00% | 9c8210c60d33be851d2c8e1ba816bbbb |
| Genus | Bacteroides | 100.00% | 4b56928f712d53a2aba98b01cd8c759e |
| Genus | Bacteroides | 98.97% | 8706390b6dcf0c8e129e553617ef53ab |
| Genus | Bacteroides | 99.99% | 9d4ed069db3d208074891eb90f45c170 |
| Genus | Bacteroides | 100.00% | 8c572474eeb90a08c18ba1959073df0d |
| Genus | Bacteroides | 99.81% | 80a8faf36a9191be7fd47f6602425add |
| Genus | Bacteroides | 100.00% | 8f9d66813b4350f57e93005187160d04 |
| Genus | Bacteroides | 100.00% | e904b862eb696252d4389c46093a00f3 |
| Genus | Bacteroides | 100.00% | 585832f1024166b0b8f0d334dfffb801 |
| Genus | Bacteroides | 100.00% | ff8393466786bd2ce117623ac44fee08 |
| Genus | Bacteroides | 100.00% | cdf5ed0814277216af92c96265f3621a |
| Genus | Bacteroides | 100.00% | 3853fb5c5b9d02be2f0d69fc1a20f38b |
| Genus | Bacteroides | 100.00% | a82e91b9e5f47a536b44efe156871a82 |
| Genus | Bacteroides | 100.00% | 3cd76566159bf4695d403b0a830a46b9 |
| Genus | Bacteroides | 99.97% | 43f443ba3039aaf174db2dfc95f682f5 |
| Genus | Bacteroides | 100.00% | 6c3350f4589d5de998b1b7bae21b8bab |
| Genus | Bacteroides | 100.00% | b1febca22c197e05ae1e870d5f313782 |
| Genus | Bacteroides | 99.93% | 0e6976602303f02016f7f7f16e5f1cae |
| Genus | Bacteroides | 99.95% | f3f93ea62f8734e5a6349075fd7c09eb |
| Genus | Bacteroides | 100.00% | 675e7ff959aa8cdc440772f48778103e |
| Genus | Bacteroides | 100.00% | a095ad1f8a168d65e5b35ed1d31a6c73 |
| Genus | Bacteroides | 100.00% | 02076327a40b41e980001d1d16ed53c7 |
| Genus | Bacteroides | 100.00% | fccc16c6e02cb7557a5fa7f5ae08aaef |
| Genus | Bacteroides | 100.00% | c41290aa09743e6c4f369ae7f1cfee19 |
| Genus | Bacteroides | 99.54% | 24cacd61061ba46fce1b0d30c3e992b7 |
| Genus | Bacteroides | 100.00% | cdf13cca19d9f8175b066b8846ae6eda |
| Genus | Bacteroides | 100.00% | 5ece169faf98bb575fb9c2bd7f68f9f2 |
| Genus | Bacteroides | 100.00% | 44798acdfd3eeaec6f5b5ea07f3ffc9d |
| Genus | Bacteroides | 99.99% | 2121d128034bd807d1b8ad495826dde5 |
| Genus | Bacteroides | 99.94% | 54c3e2e285f6ffcd7b11e5ec55c4fb61 |
| Genus | Bacteroides | 100.00% | e6045f5940a5695cd58748acb3ce75e9 |
| Genus | Bacteroides | 100.00% | 7c844bdfc254fd6857ed426a5ba3a7ac |
| Genus | Bacteroides | 100.00% | e161952d19aaf9d5ff71fb113dc5bbc9 |
| Genus | Bacteroides | 100.00% | ce8138ced09a7c70967d9630b2de8d59 |
| Genus | Bacteroides | 100.00% | 939dd6607ccb5d0eb77dc84460a84164 |
| Genus | Bacteroides | 100.00% | c56b0d2b0dd60ecb3ff8ff2b859020f2 |
| Genus | Bacteroides | 100.00% | f84e5b62037dab48e069ed61cceb3169 |
| Genus | Bacteroides | 99.97% | 1223886741e61d84d295110da2a18404 |
| Genus | Bacteroides | 100.00% | e00ee61282546b993e5f3e3d866c478c |
| Genus | Bacteroides | 100.00% | fafbcdd62ea5705270bc1bb83fa4a61e |
| Genus | Bacteroides | 99.94% | 588961db4c7865611514b1659e040923 |
| Genus | Bacteroides | 100.00% | cc3c2ebf934cb552ffe1ddb429c8d08a |
| Genus | Bacteroides | 100.00% | ba77d51f11c355c4fb8b7cf95f3e768f |
| Genus | Bacteroides | 100.00% | f1b5a2b720bc9762137b57b6c6d4bbe2 |
| Genus | Bacteroides | 99.97% | 82f1428e7e6a9f942b821a86b5ac92af |
| Genus | Bacteroides | 99.93% | 8f32bffe5c16c89f13c415006835ab4e |
| Genus | Bacteroides | 100.00% | 4f33181765b680a12f041d209752b660 |
| Genus | Bacteroides | 100.00% | 8812cf218a66d658bb00703a1c8e10ad |
| Genus | Bacteroides | 100.00% | 0d6e129365c35ac38f4d7be1a668b90b |
| Genus | Bacteroides | 100.00% | 0f44ee3bf0792a30422f2e595bccd92e |
| Genus | Bacteroides | 100.00% | 4e001a4319e19c0722a91f11ab40fecb |
| Genus | Bacteroides | 100.00% | 5692209c211e6f974191b3a058d6f284 |
| Genus | Bacteroides | 100.00% | d4d6be9c03f370514d560efee9692947 |
| Genus | Bacteroides | 100.00% | 72b7a9ed4061030ba1fb133f8df97c26 |
| Genus | Bacteroides | 100.00% | c12be00eb103128bf03005bdaae69899 |
| Genus | Bacteroides | 100.00% | 206d9a43041d40d91362b0d3c31a9b46 |
| Genus | Bacteroides | 100.00% | b31e3f67df2df0a1f2dbefed3b488099 |
| Genus | Bacteroides | 100.00% | e8a2b5094fb878b6a91620ddfb0ab54d |
| Genus | Bacteroides | 100.00% | d01cbacb8ac55a5d8c71d44aba441e83 |
| Genus | Bacteroides | 99.93% | b76c6f2b214aa86fa8107f858ac26950 |
| Genus | Bacteroides | 100.00% | 12243f7f8742269de74e39d9afbcabd0 |
| Genus | Bacteroides | 100.00% | fedaff40ff72a0aa6f17c8e5e3041dbb |
| Genus | Bacteroides | 100.00% | bb92d377601cd7692be81ac3f2ab9ce7 |
| Genus | Bacteroides | 100.00% | bd52c4ea1ff9e8af436dc7a5e3280428 |
| Genus | Bacteroides | 100.00% | 117f11c3b236e9b11a0270d044a2152d |
| Genus | Bacteroides | 100.00% | e01a4122cb5a9bd289520111fe25ec46 |
| Genus | Bacteroides | 100.00% | 6f495048eccaa0fd5082a2a9c07202a2 |
| Genus | Bacteroides | 100.00% | ed6bfd86ff7a83e674b70a4954e6fd05 |
| Genus | Bacteroides | 100.00% | c6e9f4835f720e38bcf7cd61ed0a456d |
| Genus | Bacteroides | 98.25% | 76656e9a18450ca6fada38204eadd913 |
| Genus | Bacteroides | 100.00% | 6557b3813fef635167da79caed22f567 |
| Genus | Bacteroides | 100.00% | 96d8d96ae2ada91f15887ec3189c1d15 |
| Genus | Bacteroides | 100.00% | 81fda12478944c2253a6995d36eaf251 |
| Genus | Bacteroides | 100.00% | e7986366daa50ec4f795a761052f0dd0 |
| Genus | Bacteroides | 100.00% | d69947145c27a597601c6b1dbbdca6bb |
| Genus | Bacteroides | 100.00% | 3c65b394869ee44c3e3ad94b0afe620d |
| Genus | Bacteroides | 80.89% | 719d708ae8957f786cdba777923c8d37 |
| Genus | Bacteroides | 100.00% | 169758219d5b1595ce1c67b6c3758632 |
| Genus | Bacteroides | 100.00% | cb5e0f843b64714ae3550283676acc32 |
| Genus | Bacteroides | 99.99% | 204cf25d24527db47ac4807e493198bc |
| Genus | Bacteroides | 100.00% | 86572f8b8657f61f7bbcd7352bf038dd |
| Genus | Bacteroides | 100.00% | 5a50a5c57d4caeec9687559fb52ec7d6 |
| Genus | Bacteroides | 100.00% | 3e420b1304310b22c524208bfdad9a43 |
| Genus | Bacteroides | 100.00% | 593452d53bacaeb7eeb9d9218977fc8b |
| Genus | Bacteroides | 99.99% | 2d750b7dfd78d0a42aace8fd7ba9d373 |
| Genus | Bacteroides | 100.00% | 053ce283d61bdf4f705ee35c6be85e83 |
| Genus | Bacteroides | 98.98% | fc04ccd6129bbf9ffc8a5234125afc88 |
| Genus | Bacteroides | 100.00% | 9ed170f341578083c70075cab60c9b21 |
| Genus | Bacteroides | 100.00% | 0c55ab25259106b367f196ee179fd7ee |
| Genus | Bacteroides | 100.00% | 15f7baf2918365f0165dcc137c956795 |
| Genus | Bacteroides | 100.00% | ba21722ad83e8c9dea3b7b0d8be5ae09 |
| Genus | Bacteroides | 77.00% | ca9c4c76997d3e188865ecf82486cac3 |
| Genus | Bacteroides | 99.99% | ad1d6b25879876f48c1e363772caf5ac |
| Genus | Bacteroides | 99.97% | bd8486fd1d4e949cdaae1112f9505af4 |
| Genus | Bacteroides | 100.00% | c7847ceb0684d869647fb8eb101a0a98 |
| Genus | Bacteroides | 99.96% | cd13b63ef34872d71f982bbe9d4ddf51 |
| Genus | Bacteroides | 90.06% | 38cac64a6cf0701434f02f711498076f |
| Genus | Bacteroides | 100.00% | 54d082da1d750e5713a901b43257e93a |
| Genus | Bacteroides | 100.00% | 67f64c648fc304193ba42e21d8836a50 |
| Genus | Bacteroides | 100.00% | b16cf2028efbaacb78c603a5c17a61e0 |
| Genus | Bacteroides | 100.00% | 5b80ce2e91b22d3a398f126e3879213d |
| Genus | Bacteroides | 100.00% | 620da8c8d0bdf605b7777141bd157f43 |
| Genus | Bacteroides | 99.97% | 3061eec0272b3f40b1fc980ff5910c47 |
| Genus | Bacteroides | 100.00% | ccdd23925bada127508271c2a66b63df |
| Genus | Bacteroides | 100.00% | 9ee448052e479408b354b181ba8ccf1d |
| Genus | Bacteroides | 100.00% | eda49cf194e5d5e04c0b0c7ffa84185a |
| Genus | Bacteroides | 100.00% | 15cb34557914be391bf32b4a49784ea9 |
| Genus | Bacteroides | 100.00% | 6239ffbeabeca1e3f5104baab51afdf6 |
| Genus | Bacteroides | 100.00% | a44e66f0e4455c11924a9657855fb033 |
| Genus | Bacteroides | 99.97% | 7f57b2d55ae29e55c88c7d0fef0b68cf |
| Genus | Bacteroides | 100.00% | 114a27fc1bd54ac6eee0b0e588ade19f |
| Genus | Bacteroides | 100.00% | c301d30617c42abbbe38b0bd5540dd5d |
| Genus | Bacteroides | 100.00% | 4e26b667c987ef46f6de816131c208d4 |
| Genus | Bacteroides | 100.00% | 8419b6f4fe3a03ee766bebb730c06a65 |
| Genus | Bacteroides | 100.00% | 75e7be933c1423f8df93033ec7dade5b |
| Genus | Bacteroides | 100.00% | b0e73f93d20c824bdc6219d372d3db37 |
| Genus | Bacteroides | 100.00% | 439b9aabfdc08df1be23576e80155b4f |
| Genus | Bacteroides | 100.00% | 5fea5ac4bbe61f5b3f52fe658793f503 |
| Genus | Bacteroides | 100.00% | 38a2212a647b2b4800f421237fd41af2 |
| Genus | Bacteroides | 100.00% | 13e20aa893794af8dfe937117349d269 |
| Genus | Bacteroides | 99.99% | 2ef1a0277b3e23c18b45b6b0ba2728d0 |
| Genus | Bacteroides | 99.90% | c525be6ff9c4d567f426619165191d9a |
| Genus | Bacteroides | 100.00% | 00187a18656dfdcbce0ba4de3d3ddb8c |
| Genus | Bacteroides | 99.86% | 4048bc32a1200391bb255025861d2769 |
| Genus | Bacteroides | 100.00% | ecb5ae6bab5876cc679b2ad305e0434f |
| Genus | Bacteroides | 100.00% | 61f669ae91e6cf63fad048c20984686e |
| Genus | Bacteroides | 100.00% | d7d8bace096a1db0cd367e86e0f86c16 |
| Genus | Bacteroides | 100.00% | 24ab93901d8393369e0631306748a903 |
| Genus | Bacteroides | 100.00% | 625b8fc102794cad867bb6945948a8e1 |
| Genus | Bacteroides | 99.86% | c8464eff6fb4069bdf83d5ff9b081df5 |
| Genus | Bacteroides | 100.00% | 53780bbc6b056bc60e552b94393f5e7e |
| Genus | Bacteroides | 100.00% | a493dee6485d2cc13bdbeb1c8841ff63 |
| Genus | Bacteroides | 99.99% | c91cd299eaf20b09321321d5c9eb0448 |
| Genus | Bacteroides | 100.00% | 70f7adeaaa198ac821ad78b7e0f67971 |
| Genus | Bacteroides | 100.00% | 1208c9909212a49b1c0623f09b98577c |
| Genus | Bacteroides | 99.97% | ec7e1f23df00e66bb8871e3a4c1096ea |
| Genus | Bacteroides | 100.00% | 420d556d7b944bebb103e6b4fa0360da |
| Genus | Bacteroides | 99.96% | d1a54ed62a7635e448928c127ea6897f |
| Genus | Bacteroides | 99.97% | 89718135696fe91b4ba3ca0175986040 |
| Genus | Bacteroides | 99.30% | e12bdc1413048cb23a74cc7cde5fb152 |
| Genus | Bacteroides | 99.96% | 0f0bb706a774d8e6150efef18f0a5557 |
| Genus | Bacteroides | 100.00% | 0c575e2a1ebf783417ac7cfc1045aed2 |
| Genus | Bacteroides | 96.38% | bbad486e9c8101bdca4e46f9b4bac66b |
| Genus | Bacteroides | 100.00% | 99304a40a1957247368a90adbcf753c3 |
| Genus | Bacteroides | 100.00% | 1814d5dc0824fd55bc6e192b347a1989 |
| Genus | Bacteroides | 99.94% | 1b8a2f73d052f0cba2157a550b621957 |
| Genus | Bacteroides | 100.00% | b866cb571186f8caa7781222e17caea6 |
| Genus | Bacteroides | 100.00% | 63e8d6b0af8d73a5bc3c98bcb00ce49d |
| Genus | Bacteroides | 99.63% | f049e2221626a413cd7e83b9fddbe9db |
| Genus | Bacteroides | 100.00% | a4aaa5810930976811c886c86199e1a4 |
| Genus | Bacteroides | 100.00% | ea12ef0af7b48bf4f8c129781d7c5f77 |
| Genus | Bacteroides | 100.00% | 392b753839ce1c020e351c412a25db9f |
| Genus | Bacteroides | 100.00% | 3f3262475e148b00223a8358c07da094 |
| Genus | Bacteroides | 100.00% | c79625d93fab010b39c0cb4812376310 |
| Genus | Bacteroides | 100.00% | 37abeec69f5802dfff7053507450a1c0 |
| Genus | Bacteroides | 100.00% | 3324626e71f52739224012f16feaade7 |
| Genus | Bacteroides | 100.00% | 5c07760cce36718cb7eb78d40ae90ed2 |
| Genus | Bacteroides | 99.96% | 0f4cc2403d33777bc3fdae2583f38cca |
| Genus | Bacteroides | 79.27% | 417d747d91f843cdc661bb4ad55b9e3e |
| Genus | Bacteroides | 100.00% | 832fe418b2f0a551f900ad7a4d7ae95a |
| Genus | Bacteroides | 100.00% | e3f1ca37374fd7e5ad6fa16e5e577bcf |
| Genus | Bacteroides | 77.03% | 7ce0235fd0c58e3eba86ed88c42c6254 |
| Genus | Bacteroides | 100.00% | 436ec56a15bbbf345c28bf555272e4f6 |
| Genus | Bacteroides | 99.95% | f9a739b190516a754216474a59c95a8a |
| Genus | Bacteroides | 83.43% | 569609637ded876a2e5a5dc5c1004a02 |
| Genus | Bacteroides | 99.98% | 06d50168a4f6e91ddf77667e30c8958f |
| Genus | Bacteroides | 100.00% | 2df92fb62b8f22ca9d1565fbdb11ebc1 |
| Genus | Bacteroides | 100.00% | 4136bb14b22200e0711ea85062d3d334 |
| Genus | Bacteroides | 99.79% | 1722f49619b023712bbcba5552de21d5 |
| Genus | Bacteroides | 100.00% | dfd66fd96eba83ddd01dad003af5e257 |
| Genus | Bacteroides | 100.00% | 8e3aac7cf52daf89fba77f9be53f44d9 |
| Genus | Bacteroides | 100.00% | 97c1d3aecd91547b2308ebd856c079a9 |
| Genus | Bacteroides | 99.83% | 4307208eeb96c635e78e582c61fee371 |
| Genus | Bacteroides | 98.97% | 0e74b035d0468f025e3fd9f3f439409d |
| Genus | Bacteroides | 100.00% | 7cf344ab7dc70efbdc12fb3dd49ca94a |
| Genus | Bacteroides | 99.96% | d6388cd7121a6424ef8bdc7226d1d104 |
| Genus | Bacteroides | 98.58% | a11e5e23117b1b06a9a35e76ac44da13 |
| Genus | Bacteroides | 99.95% | 34bdc4e684f0e93f3e08cdcf4d2caf79 |
| Genus | Bacteroides | 100.00% | 6f6037d0da07c66087d2b9574d5b362c |
| Genus | Bacteroides | 99.41% | 3a3da06cfccbf720e1be5d29bb93d47c |
| Genus | Bacteroides | 86.54% | a6f967c046d71bc2df3e69ebb7b735f6 |
| Genus | Bacteroides | 100.00% | f88f8fb0cb9a6d5a8c3abcfe7a8b3a2a |
| Genus | Bacteroides | 99.95% | 23688e4e44eb61242e6daaa6a4e1e40d |
| Genus | Bacteroides | 100.00% | 8ffd5c84ebb1f2273937b33891ab28ff |
| Genus | Bacteroides | 100.00% | 256b1bae3385dc274d22c03bd8873f37 |
| Genus | Bacteroides | 99.99% | ad2dfc85b420b0cbb92073d1afe2633e |
| Genus | Bacteroides | 100.00% | 216f030902ccef79fdae735478b1b8d6 |
| Genus | Bacteroides | 99.94% | ee1f220c6d4aa66fa115f0cb1d15ba2c |
| Genus | Bacteroides | 99.94% | 887b79f4d81b45ec458db591267f3b06 |
| Genus | Bacteroides | 100.00% | e0899825a44bfed0291064be298c4657 |
| Genus | Bacteroides | 100.00% | bc620d611e7f4f6ed138a5a1fff06c80 |
| Genus | Bacteroides | 100.00% | e383a95b58dd95f05d319a68768395dd |
| Genus | Bacteroides | 100.00% | 1da7feed95b44713174a289f3a8765f4 |
| Genus | Bacteroides | 99.24% | 8d8ff07f7bcb8075c6033d0e4e23dd49 |
| Genus | Bacteroides | 100.00% | d633ed7874450b62f5cd6c23940e06a3 |
| Genus | Bacteroides | 100.00% | 9cd93bab2e5d56b463d91d823b49b202 |
| Genus | Bacteroides | 100.00% | bb69e37ec9b605f89355f4bd1caa601f |
| Genus | Bacteroides | 100.00% | 6b9e463d35418199492b3b1c38e1336d |
| Genus | Bacteroides | 100.00% | f61f4bf54d2fe499dd0913218e91de9a |
| Genus | Bacteroides | 100.00% | 73e1b188e5a64143648fd0582e7af60b |
| Genus | Bacteroides | 100.00% | aa101e160635387711ddb9c445585d13 |
| Genus | Bacteroides | 100.00% | ac3d777a77b89e2fdb400bf80c25a92e |
| Genus | Bacteroides | 96.05% | 69db344bfb479ce3b574f1cf3e6838f8 |
| Genus | Bacteroides | 100.00% | 952c7bec39af8bc1d58a1591e4a43bf8 |
| Genus | Bacteroides | 100.00% | 4a5b978c0492d6cbd0db725949c66546 |
| Genus | Bacteroides | 100.00% | 707d256dc10d44475e4d829823a75554 |
| Genus | Bacteroides | 100.00% | 982a1deba26eb99164393933a55f359a |
| Genus | Bacteroides | 100.00% | e6ff95c58d33e8ff5e39299bca7e6af2 |
| Genus | Bacteroides | 100.00% | 6828f5fd5ffe3d9818bcdb1b3d324e00 |
| Genus | Bifidobacterium | 81.57% | 2d0d1e70213514a71686b975749fce6a |
| Genus | Bifidobacterium | 79.52% | 1a653de44e0f0f30139bf5e48aa41cfa |
| Genus | Bilophila | 99.58% | ac92cf3c3964343ecb3384d6e99a0690 |
| Genus | Blautia | 71.92% | 232bc672bcc43d93dcfa3adeb6d0ecd5 |
| Genus | Blautia | 74.40% | 98b441433fb5f567b78c0d8024484341 |
| Genus | Blautia | 70.84% | 9247c524adaa76651992c06442bf1381 |
| Genus | Blautia | 75.12% | c489832e825fd05bd35f57aed77e0aae |
| Genus | Blautia | 73.29% | 0cdb40460fbf2f28a0e7299a444da049 |
| Genus | Blautia | 72.91% | 836d88ea82ffa44ca3c54f9094e071cf |
| Genus | Blautia | 72.06% | deef2db068fc1b17837b6f9315d5eeca |
| Genus | Blautia | 72.53% | 1ef3bac4b2b06e6e8b263c959706bc23 |
| Genus | Blautia | 74.18% | 18bf58bd3ce2ad9c36443ea3a6252c96 |
| Genus | Blautia | 71.31% | 5bc832061f5561065e9d84ff9c6c4d95 |
| Genus | Blautia | 77.03% | 06b55d1d90deb3d726ce24bbba04a10c |
| Genus | Breznakia | 73.27% | fc6d9b4bc3346ff4b422ce7096f700f8 |
| Genus | Butyricicoccus | 81.14% | 4b8bee1dbd81699a753985e965c1a937 |
| Genus | Butyricicoccus | 98.35% | 0a8e06355489cd9532ebf8bf3167b044 |
| Genus | Butyricicoccus | 81.12% | d7f1aa0c3fe48f8edfd22ddf8aa5cbff |
| Genus | Butyricicoccus | 99.14% | 40b8c48e74fec1b3e123b1dd27ff5337 |
| Genus | Butyricicoccus | 81.12% | d6f742866bc4efbf50bf6fa5c306abb4 |
| Genus | Butyricicoccus | 99.05% | 1526ff74623db0182b7b76cabecfa4d4 |
| Genus | Butyricicoccus | 99.13% | 68562659d4c7b186e40b50ecf64ed05b |
| Genus | Butyricicoccus | 83.71% | 80d7a3a26857563559751ec8bf26e4c0 |
| Genus | Butyricicoccus | 88.98% | 0eb32ca46280cef19e9c9b349b9dde52 |
| Genus | Butyricimonas | 88.91% | a35354a41c3c9ebfc530b1283225cac0 |
| Genus | Butyricimonas | 86.68% | 26d5e61d7c807cbf823363dd6ac6fa61 |
| Genus | Butyricimonas | 86.37% | 52412d0fabcf9293de76765dd3720e38 |
| Genus | Butyricimonas | 80.12% | 596cf6ba1178599eda672a58370c0f44 |
| Genus | Butyricimonas | 88.91% | f72cccef87f8c9c9e15c0a2052a1c9f1 |
| Genus | Butyricimonas | 88.91% | 6d2714a6b5a3d3cf49d959c0ca81b73e |
| Genus | Butyricimonas | 76.42% | 5cb048df6f8b896ed9752c257352d2c4 |
| Genus | Butyricimonas | 85.50% | 7b74ebfa12314512a502ab6637d744f1 |
| Genus | Butyricimonas | 97.09% | cdf794e6d306a3a5090c3bd6d93f2ae9 |
| Genus | Butyricimonas | 88.92% | 11d0b0228ddbf1f8179d0506babfaed2 |
| Genus | Butyricimonas | 96.38% | 6a66b9c888a8aacd94db2429a5cce3b8 |
| Genus | Butyricimonas | 88.92% | 1920e888b9e85e80f2d28403e516147d |
| Genus | Butyricimonas | 90.75% | 625fc940759cb8506d6f9f8467ba9aae |
| Genus | Butyricimonas | 86.32% | c3682f72431157d9b94ad9795ac5794f |
| Genus | Butyricimonas | 91.53% | fa0ad1ccbeddd0ec672f2c82b9fced41 |
| Genus | Butyricimonas | 91.50% | 9af0f6477ff91b879e0c3ae0a2fa43a4 |
| Genus | Butyricimonas | 96.18% | 3b8d3792d4217b6081611f5be1cfc6a3 |
| Genus | Butyricimonas | 76.34% | ba2c2993175d642fa0476b76754d69ff |
| Genus | Butyricimonas | 94.97% | 81f952c8c31a45c2df0a6944cd696890 |
| Genus | Butyricimonas | 85.51% | 39f8ba4fa0d627cfe9ef4efdb3014529 |
| Genus | Butyricimonas | 76.20% | ec4f91a9bcc1d1733950a3740f7f8651 |
| Genus | Butyricimonas | 86.01% | cdd6553b28c367454850a22a82a39050 |
| Genus | Butyricimonas | 94.52% | 47ce7f5940b832003f0f7fab8b15160b |
| Genus | Butyricimonas | 85.51% | b6a488018a7a2b01db14326ddcf6176c |
| Genus | Butyricimonas | 91.45% | c605eafa7d72523efce8cf001802eec8 |
| Genus | Butyricimonas | 78.67% | 4587431d049d03e8141294c2ad28f166 |
| Genus | Candidatus Rhabdochlamydia | 100.00% | 8466c97df0f1fa0cbf4ef763c1050b4d |
| Genus | Candidatus Soleaferrea | 93.49% | 1a9fd16a3326b81188afc68338249963 |
| Genus | Candidatus Soleaferrea | 83.68% | 9657caea4bdfb72510b0f59551c0ae9c |
| Genus | Candidatus Soleaferrea | 85.50% | 2068a9da9844e08c2162f62ef15f36e4 |
| Genus | Candidatus Soleaferrea | 72.25% | 86d3dbfaf3c87722376579d50c87c007 |
| Genus | Candidatus Soleaferrea | 70.51% | 78219c39a192b07a47210b98c5a8f8b4 |
| Genus | Candidatus Soleaferrea | 100.00% | 7ff5c8921d2092579c6e00308c67ae7b |
| Genus | Candidatus Soleaferrea | 72.26% | 6715daccf1e0fd43ec823989a7b4e988 |
| Genus | Candidatus Soleaferrea | 100.00% | acae36cb021b62c578f29bbd075940b1 |
| Genus | Candidatus Soleaferrea | 99.98% | c820b2c25723cb61d94c7438924a7fc4 |
| Genus | Candidatus Soleaferrea | 100.00% | e3d0a536d4c1b811bb0c55ce9617e4c2 |
| Genus | Candidatus Tammella | 90.13% | b9e05ecf37bd1004ee70253b757bd33b |
| Genus | Carnobacterium | 76.51% | 3730b7e53e62287fa85e754071ce032c |
| Genus | Carnobacterium | 76.08% | e3c82c0fbbb314e7654489d0ad6efd61 |
| Genus | Carnobacterium | 73.18% | 1291872a929aee681c77ebb59a76b2da |
| Genus | Carnobacterium | 76.05% | 3effd86e90ecd266dd8eda0102c68513 |
| Genus | Catonella | 99.99% | 1987893f82b7ae8dc12ad3dcdd430e6a |
| Genus | Cerasicoccus | 93.78% | 45666569cff450893858b564e15092b0 |
| Genus | Cerasicoccus | 97.52% | 15d3ab3766ce591f70c0905b969a0a9c |
| Genus | Cetobacterium | 74.63% | 474f2b72240da64666ac8cded67e81b5 |
| Genus | Cetobacterium | 73.27% | b4e2ffe472703c97855d111c66cc5c67 |
| Genus | Christensenella | 98.72% | 7650b49fce67889b2c46bb79f3aab173 |
| Genus | Christensenellaceae R-7 group | 99.57% | ad4205724232522e9a2d83b4439d9ad0 |
| Genus | Christensenellaceae R-7 group | 99.58% | 0c46a734ff0372c3b42e986d8d4fbd7c |
| Genus | Christensenellaceae R-7 group | 72.74% | 923c3bc5cbaf21e9c7a0c1aaff204b63 |
| Genus | Christensenellaceae R-7 group | 77.77% | b5c0ee58ac6d2b32b394df06d9ce1e9c |
| Genus | Christensenellaceae R-7 group | 99.78% | 76c34ce0806c7107891597aac4194fb2 |
| Genus | Christensenellaceae R-7 group | 99.88% | a3f654b93846b57cfec8bd4e0223591b |
| Genus | Christensenellaceae R-7 group | 97.66% | 78e0cb78330427148cdc185b5bac013a |
| Genus | Christensenellaceae R-7 group | 99.53% | 217a44d94c70387ee55527f2d9908193 |
| Genus | Christensenellaceae R-7 group | 80.52% | 91212de5a3ebeef860e015e44e42bff4 |
| Genus | Christensenellaceae R-7 group | 99.36% | d04ff089680f044643240ca17a67f9e3 |
| Genus | Christensenellaceae R-7 group | 85.70% | fae075d576bebe06290f1d9f46eff94c |
| Genus | Christensenellaceae R-7 group | 72.00% | 61f75e0e13b90935ebab1adf951ad69b |
| Genus | Christensenellaceae R-7 group | 97.69% | c9d7c1e77855aef123c47595269826fd |
| Genus | Christensenellaceae R-7 group | 99.54% | e31057fca83e3e9be1cc45466cfe6098 |
| Genus | Christensenellaceae R-7 group | 99.93% | 5c43675886a82a462de2cce85b69b543 |
| Genus | Christensenellaceae R-7 group | 99.90% | e777efaead7d4e47abd472a35f2b54a7 |
| Genus | Christensenellaceae R-7 group | 76.30% | be6027f79cb46f124431eba5d7178ad7 |
| Genus | Christensenellaceae R-7 group | 97.40% | cb62442fe238ab3db23991ba35c7ed8d |
| Genus | Christensenellaceae R-7 group | 99.97% | afb59f8a4133e56badb1879218ae7e38 |
| Genus | Christensenellaceae R-7 group | 99.98% | 32642fd503e91d04a68b1c39465b5039 |
| Genus | Christensenellaceae R-7 group | 78.74% | 15a8b2937f33cd3e3a8aa49b00564eb5 |
| Genus | Christensenellaceae R-7 group | 97.79% | 518b42d0470da9799e62512d303ceb99 |
| Genus | Christensenellaceae R-7 group | 98.82% | b13d96408cd73b23278192edebcc2f4f |
| Genus | Christensenellaceae R-7 group | 98.15% | 4a0207883cbb601384dd2894a1868eef |
| Genus | Christensenellaceae R-7 group | 76.86% | 5eff65e61237106e51d11afed54ebf18 |
| Genus | Christensenellaceae R-7 group | 70.67% | 84b4d359a85e80e8881af117d32bf4c6 |
| Genus | Christensenellaceae R-7 group | 92.35% | 1eb3b6ed0ec4bd604f920066ebac33ce |
| Genus | Christensenellaceae R-7 group | 87.10% | 1fce6036bef94945386db423f4fce694 |
| Genus | Christensenellaceae R-7 group | 75.19% | 1fb97cc1c05dc0378e7e062a6fd177fa |
| Genus | Christensenellaceae R-7 group | 90.97% | 26bf355c075900edc32815262da8ecbd |
| Genus | Christensenellaceae R-7 group | 89.28% | 32c9c9e00bb51f32785158d3dd179cfb |
| Genus | Christensenellaceae R-7 group | 76.33% | 5905f269180d14f2a79d35d3bc0d2aa2 |
| Genus | Christensenellaceae R-7 group | 80.47% | 9b62bc6aca3a3c4c208014b4744d40d0 |
| Genus | Christensenellaceae R-7 group | 85.98% | 2f31644e55d9b6ae508171c4cca68eb8 |
| Genus | Christensenellaceae R-7 group | 99.69% | 1b7503013e22b1781c3bbcfcfa7d60c8 |
| Genus | Christensenellaceae R-7 group | 98.69% | 82212f75814142720d30d520a48c18ca |
| Genus | Christensenellaceae R-7 group | 99.00% | 90391923088d8310263dabdf85bca923 |
| Genus | Christensenellaceae R-7 group | 99.59% | 4645ffae5c74f336b87c2cacdfbab3e4 |
| Genus | Christensenellaceae R-7 group | 99.76% | 7d3271fd8d6a27d982f3e15c2ac36132 |
| Genus | Christensenellaceae R-7 group | 76.72% | f66f994739212422900788a74ffec5e5 |
| Genus | Christensenellaceae R-7 group | 94.68% | 2014f2d26ea5179a3e0df05b2a21adc1 |
| Genus | Christensenellaceae R-7 group | 98.85% | 0e7028874fee24e8727f0632c265d1e7 |
| Genus | Christensenellaceae R-7 group | 95.49% | 4862a8e41af6c04a73d87af78492e424 |
| Genus | Christensenellaceae R-7 group | 83.45% | 0564cdc1392a177f0879614c994ce639 |
| Genus | Christensenellaceae R-7 group | 79.03% | 991f3998020443955b5b5b552a97d553 |
| Genus | Christensenellaceae R-7 group | 76.48% | f24e997aef4bd2a3b8d4b1ef0550c5e4 |
| Genus | Christensenellaceae R-7 group | 79.23% | 4bbf7dff55efb691be5cb72f62270e9d |
| Genus | Christensenellaceae R-7 group | 94.90% | 5c0d5fea87cb3d3c1be3abda633451c4 |
| Genus | Christensenellaceae R-7 group | 94.97% | 5ba85102bb647cbf7de396310c476d6f |
| Genus | Christensenellaceae R-7 group | 87.43% | 3c47874969892dcd8c3bc684de9220e0 |
| Genus | Christensenellaceae R-7 group | 78.03% | d446cffdba231a5158a41483c7e832dc |
| Genus | Christensenellaceae R-7 group | 86.18% | 1bb70f34c3df0b1aa1d00d32876a4903 |
| Genus | Christensenellaceae R-7 group | 73.77% | 70df034e9c4816b21a19b2911524a4bf |
| Genus | Christensenellaceae R-7 group | 86.03% | bfe6328099f637e30e10a2e94e1d27c5 |
| Genus | Christensenellaceae R-7 group | 98.86% | 5ae995060e103967be79db3caa55916e |
| Genus | Christensenellaceae R-7 group | 96.94% | 334151858f4fea66d037bf6e907c588d |
| Genus | Christensenellaceae R-7 group | 83.16% | 258d231e66dda3b8fafa6eede5e1745e |
| Genus | Christensenellaceae R-7 group | 86.15% | 0953579b88a90a991f15cbccd276e84d |
| Genus | Christensenellaceae R-7 group | 86.78% | be87a62fd5502e80cdb6e006748198b4 |
| Genus | Chthoniobacter | 99.11% | d471b147f8a4c5de14b348ad16c92068 |
| Genus | Cloacibacillus | 100.00% | 6e5c05c753d9f9e724f52b6a6182434f |
| Genus | Cloacibacillus | 100.00% | 92954ebe9cc030f75bd3cc71bf8e47f7 |
| Genus | Cloacibacillus | 100.00% | 93acdffa9530fff9fc32c02d6cf81b97 |
| Genus | Cloacibacillus | 88.82% | 3843ab49aaa85d2a1612ba8eda61dd3c |
| Genus | Colidextribacter | 76.66% | e89f74a3fa22ac4eb90c12e2161d95bb |
| Genus | Colidextribacter | 72.46% | a775a111c2e5a070f75fd50261eb0962 |
| Genus | Colidextribacter | 80.88% | 443e6a7a3fe96349081a71d7c7893510 |
| Genus | Colidextribacter | 76.27% | bc5c1eb653a93c2628e25a9e92eeb781 |
| Genus | Colidextribacter | 72.60% | 1e9a96ab928969b40d2afec2db68ae54 |
| Genus | Commensalibacter | 99.95% | 4a62aecc299ef17fe31d21750a22104c |
| Genus | Commensalibacter | 99.98% | da0b43ef3804e18c4f2e069aa1a844aa |
| Genus | Coprobacter | 70.74% | 56baa8616a1a0f1fbf50ab2ee214882b |
| Genus | Cutibacterium | 96.78% | 83071799eac1cebba91918529165f752 |
| Genus | Cutibacterium | 96.78% | a12f0929f365e3ffe445890919158fab |
| Genus | Desulfarculales | 100.00% | 04093441fd7e65a010479ae6208de40d |
| Genus | Desulfarculales | 100.00% | 57a631ec68f9eb567aac99d6f0194533 |
| Genus | Desulfovibrio | 80.41% | 6eb2006a2ba06eddaddf4a80938707f6 |
| Genus | Desulfovibrio | 98.86% | 6a87e62915b7482753e926b2063a6272 |
| Genus | Desulfovibrio | 100.00% | 9920069e6b1eb8457c6020ee8beea132 |
| Genus | Desulfovibrio | 99.59% | 31377b43031dc7bb65748efbb858bceb |
| Genus | Desulfovibrio | 97.18% | 3ea9ab8ca78c2a1bf51f00e6864022a5 |
| Genus | Desulfovibrio | 100.00% | 4b788a2ebae59aeb170c9d8a4ce5c831 |
| Genus | Desulfovibrio | 99.17% | 83f5c6423f3733372a8e6e8aa0079cf4 |
| Genus | Desulfovibrio | 99.75% | a4a0d584415c6b2b264471bb6b0110d0 |
| Genus | Desulfovibrio | 100.00% | 1f51ae64a1e209e8dde29cc47dc52a89 |
| Genus | Desulfovibrio | 100.00% | 02f06df284a59c63b7a6ff4fb3507c64 |
| Genus | Desulfovibrio | 99.76% | 36ca23130d6cde5cb7ae751de4242216 |
| Genus | Desulfovibrio | 99.63% | d00f7d2ab822413c2c4ce53f5aa1372a |
| Genus | Desulfovibrio | 98.43% | 7d297d15538f4cd6988c1f35c6f7156a |
| Genus | Desulfovibrio | 98.08% | 5d927493bcb2298b5ffa0b0e4a1dbffe |
| Genus | Desulfovibrio | 98.22% | 66d2423002f7362e6b293411e8b06c8f |
| Genus | Desulfovibrio | 97.56% | f4342a7afff4410e14a114e196e63b40 |
| Genus | Desulfovibrio | 98.15% | b2811815a91b67ef53ead90e53f737af |
| Genus | Desulfovibrio | 97.80% | ddba7e4ed8084b47cadf2336e887a7d2 |
| Genus | Desulfovibrio | 96.62% | 5a5dbb006dd74b57e86b039b7c14dbff |
| Genus | Desulfovibrio | 99.73% | 2c4a6c4efc1cd7391f7001cf75b61a5a |
| Genus | Desulfovibrio | 96.34% | 5e530993ec341efaf79683b2a0ce4c08 |
| Genus | Desulfovibrio | 99.54% | 296f18a104f16d8c52cb3db2a084e2b5 |
| Genus | Desulfovibrio | 99.10% | 589b01b0aa911713845df8f21851afde |
| Genus | Desulfovibrio | 98.19% | 4a89a0ce4e81eb4dd64996fb2353237c |
| Genus | Desulfovibrio | 99.76% | c226651b8f78a118c899851a9354229f |
| Genus | Desulfovibrio | 96.24% | 3911499d5317c7be3f26a57fee155efb |
| Genus | Desulfovibrio | 98.91% | 023543601f57656df1c9fc80ca7253b9 |
| Genus | Desulfovibrio | 97.81% | 7bf27bceb4841351c3a3c60ea0236df4 |
| Genus | Desulfovibrio | 89.23% | 70dc54fe7eef63ee4927e83a2a20ca48 |
| Genus | Desulfovibrio | 100.00% | 2d81c5c8cebcfe4fed2ebbc2a8689c67 |
| Genus | Desulfovibrio | 100.00% | 337a384255894a25cbf60c7960c8f8ff |
| Genus | Desulfovibrio | 81.45% | 749d533fa57efbf605a9f592c2a6f89b |
| Genus | Desulfovibrio | 81.83% | 021e0751592821bd05a40598b5673a97 |
| Genus | Desulfovibrio | 73.54% | 36dc582be153e5fa30660b7b116de9ee |
| Genus | Desulfovibrio | 84.57% | 9fed57105ab00e51b9a567a1783664ca |
| Genus | Desulfovibrio | 87.86% | 67436e374a33df82273c06bc9da77d04 |
| Genus | Desulfovibrio | 98.56% | 5f28da0c011b4afbeedf8f506751a1a9 |
| Genus | Desulfovibrio | 72.29% | fd9fcd2b6d090a3ca08e4020e3d30f89 |
| Genus | Desulfovibrio | 88.84% | 525a28aaae2bafee8c3a62f651d94d2d |
| Genus | Desulfovibrio | 82.95% | e4bfd1cb1c136cfbb6f295047da68a04 |
| Genus | Desulfovibrio | 99.95% | f42a913672a0a45191286447bd28850b |
| Genus | Desulfovibrio | 99.94% | 85b58bfc5d294355bedc936158ae1cd3 |
| Genus | Desulfovibrio | 99.40% | e58a1cdda58c82bd1a2f07c0d679e460 |
| Genus | Dickeya | 99.91% | 3a7640643c69919a0354113b8f60aa8f |
| Genus | Dickeya | 99.89% | 4c0b08ea9e38a57fa39285bbe8fbf8f4 |
| Genus | Dickeya | 99.67% | 8ef50fa0ba0ee6d9118bc0561cd66637 |
| Genus | Dickeya | 99.69% | b3a87e6cf232e38eeb762a979d54a847 |
| Genus | Dickeya | 99.69% | 8dca78e51cc3512ee6ac37226c7ffaa9 |
| Genus | Dickeya | 99.80% | 902af8e753f3d1e173877ec5dbfb8b9f |
| Genus | Dickeya | 99.76% | 2af3ad172274a5518fa2aa9752f59208 |
| Genus | Dickeya | 99.94% | e213aa6eab20e28d5c8b83be217ddab4 |
| Genus | Dickeya | 99.93% | 540ea6e7e293f7a98b70db095a6fd69e |
| Genus | Dickeya | 99.92% | 24ff919b7db9a07690aa8b14c461039f |
| Genus | Dickeya | 99.93% | b7ebd78530f2b7daf9a7c1c5bb2e7601 |
| Genus | Dickeya | 99.92% | 07a4ad46bfa93021c7daba56558f8c6b |
| Genus | Dickeya | 99.92% | 6d272c4001912a0282b5f9edfd42074f |
| Genus | Dickeya | 99.74% | 072fbccd5eb39e08323fd1d1abbe17d7 |
| Genus | Dickeya | 99.98% | a51c97f796f563ecac8c48ec4e92f022 |
| Genus | Dickeya | 99.94% | d339e115feaf2b837f4ba0a3e9a1e32a |
| Genus | Dickeya | 99.94% | e0691e9c3dc5e50dc2624a80e46c90b4 |
| Genus | Dickeya | 99.93% | 0b8ced36aa6914edddcb97c3a108cb03 |
| Genus | Dickeya | 99.96% | 6acfebdaf3aa6eefe16b69b72b807ba5 |
| Genus | Dickeya | 99.92% | c4d287b969fc960fd42fadc52d67f6cb |
| Genus | Dickeya | 99.95% | b84ceea89632ece157e46812afdefd4c |
| Genus | Dickeya | 99.95% | 976ea920b2ad406f27592d5210c4c970 |
| Genus | Dickeya | 99.95% | 7000ff2d13f4a56e318ba029c7ee9789 |
| Genus | Dickeya | 99.80% | 90cac311949869433916d61d4d003739 |
| Genus | Dickeya | 99.94% | 672c150aa22d4d3b76dff609dca104bd |
| Genus | Dickeya | 99.83% | 38e8f215780329a34577b0279b31a306 |
| Genus | Dickeya | 93.05% | e897b63e724576b15e8fae9997375ac6 |
| Genus | Dickeya | 99.94% | 849828ca52af8e3a943e35618761a481 |
| Genus | Dickeya | 99.94% | dc7fecc9ef5d3abed87e9887960c9399 |
| Genus | Dickeya | 99.93% | 0acf7cb98f59b6b135497561444f785e |
| Genus | Dickeya | 90.56% | 5ef8b9687029310889bcf4d9c9dca287 |
| Genus | Dickeya | 100.00% | 1b9ecbe3d31ba57488c8eb1f9c5372ec |
| Genus | Dickeya | 97.37% | 156b73af5133ce1ea970cdb65d9c4ef0 |
| Genus | Dickeya | 99.91% | 4b84eae0d5988e97c832daf2383340b0 |
| Genus | Dickeya | 96.84% | 52b57562beacab3bb982462a1dfcaa9c |
| Genus | Dickeya | 99.93% | e2a0eaae836b0e22fb81b118e576bf39 |
| Genus | Dickeya | 99.99% | 3e4b54ec0093c86d06c143159a7a1559 |
| Genus | Dickeya | 99.95% | 79b2bdeb2ae5e05f2be49ab8c637f8df |
| Genus | Dickeya | 99.99% | 6034e0c558045783c45ba339e9b6c4a6 |
| Genus | Dickeya | 99.91% | 793d6e9f0ee26c241c65f6b87720b746 |
| Genus | Dickeya | 96.26% | b07240161241df091dd76c975a8a5ed8 |
| Genus | Dickeya | 95.41% | dca39e0f09bde098ea2713b7a86fc4e4 |
| Genus | Dickeya | 99.96% | 924b34d2f4d7a1ca3e0e861d5475235c |
| Genus | Dickeya | 99.89% | 78705043055525a129d853ebc7674c95 |
| Genus | Dickeya | 99.96% | ffd10dd4c9f6a114e31cc03905830be8 |
| Genus | Dickeya | 93.89% | 1b9d4f5b2938c4b9f812d42afa12f3ac |
| Genus | Dickeya | 99.96% | 617e1f76108b93e11d09e323538e613a |
| Genus | Dickeya | 99.89% | 2ee9567ab662674ba6243f2b6f1578a3 |
| Genus | Dickeya | 95.46% | 1d4315deaad49240950cbc41921b737b |
| Genus | Dickeya | 99.83% | 16d7312342ca175b070f7284418e78b3 |
| Genus | Dickeya | 93.77% | 94e7a0f104df79080c8a7b80a697ba4e |
| Genus | Dickeya | 99.80% | 2ca154f311fe230a4e1527034822e5c2 |
| Genus | Dickeya | 99.96% | f43ec04baa051795046464e216b1bf74 |
| Genus | Dickeya | 100.00% | 447dc264e5bebba720b6cea743cf46a5 |
| Genus | Dickeya | 99.96% | 10988c77e6e7f07f76ddfaa87e657ba8 |
| Genus | Dickeya | 99.97% | 0dfcdfecb328c04b19564ceb8f0d9df2 |
| Genus | Dickeya | 96.92% | 5eb551f74c669f49a650f351d6204e88 |
| Genus | Dickeya | 99.94% | 1c1eb97e6007e8386a16acdb112f3e5d |
| Genus | Dickeya | 99.89% | f45a8742358a24633bd8425adf298b25 |
| Genus | Dickeya | 99.61% | 36a3fc8f2d7223cd00b9de1ede4e4c90 |
| Genus | Dickeya | 99.92% | 155f4bb0d1bb8621d12a03ac5a0679ae |
| Genus | Dickeya | 97.38% | ea6df2ec8cf6ffc36f6893487c492de9 |
| Genus | Dickeya | 93.12% | 9f05bc4e4842d4044330b4f0a31c9ad4 |
| Genus | Dickeya | 99.90% | 7949d9d0ffdd360fd39dd85ff0649c27 |
| Genus | Dickeya | 97.38% | 8c7daa31f83011d21e741ed42524dfc0 |
| Genus | Dickeya | 99.88% | 36af27164cc01d035f0558a8f8674ce6 |
| Genus | Dickeya | 99.86% | e6c8f32e88bee38f61599963d06b44ca |
| Genus | Dickeya | 90.64% | cc08d446c2fb01c710091534c991db23 |
| Genus | Dickeya | 99.94% | 3fa2b110e52998cd88a3bb7eb982a040 |
| Genus | Dickeya | 99.63% | 1396de44a72e198155747870b4e238c9 |
| Genus | Dickeya | 99.83% | 56935a7841b116267cbe2b6eec30150a |
| Genus | Dickeya | 100.00% | 032bc6eb3503eaae6ed3b6a7c68e5a54 |
| Genus | Dickeya | 99.84% | 098810585c2ded655da98384ef154f1e |
| Genus | Dickeya | 96.88% | 1fcd83f168af6be5b53af14ae1109076 |
| Genus | Dickeya | 99.96% | 2d2d5ecb8de0f5852e2e33fda91df71a |
| Genus | Dickeya | 99.84% | 42c6b0f6b6d0c7ca7aadd03d6af79bae |
| Genus | Dickeya | 96.35% | 87b6549fdc0d1539a9c0925137df2227 |
| Genus | Dickeya | 99.71% | 64a0760659d2ad6297afc3bd1e659366 |
| Genus | Dickeya | 99.86% | 85e809c2b3bd2a45c5747c33857dfe37 |
| Genus | Dickeya | 92.52% | 3874883434e5faf7ea5fbfd5b953ceed |
| Genus | Dickeya | 99.92% | 72d381932d02b5e37720b499bcd0d742 |
| Genus | Dickeya | 99.93% | d1f84eb0d8a7d2b72d5c026cf1956b00 |
| Genus | Dickeya | 95.68% | aaae3b8a244183500532a64530f9981d |
| Genus | Dickeya | 96.85% | 64411fcc37b72c0444103abc596100b5 |
| Genus | Dickeya | 100.00% | fcff3c9f469ca6cf20ff3e399debc9b9 |
| Genus | Dickeya | 99.98% | 2b1871b0de13e643ffe4f928f7376d0d |
| Genus | Dickeya | 99.92% | b991a2f590ef221e1059afb50b6ae7b4 |
| Genus | Dickeya | 99.89% | ef97e1bb021a1268752ffd912e6ce6e8 |
| Genus | Dickeya | 99.95% | d35df9ee2e375dfe2b25d103ece32358 |
| Genus | Dickeya | 99.95% | 6d5416620167c66c7f5ed15c5ce1535b |
| Genus | Dickeya | 99.86% | 569cf27ef63ce36628bca0f20625ff7a |
| Genus | Dickeya | 99.86% | e3d9346a7846702e3707e8b9d057dcda |
| Genus | Dickeya | 99.99% | ce5c571563bd53339ce23f29b748376e |
| Genus | Dickeya | 100.00% | 83b3df022e52e1e448051f9f903f21a6 |
| Genus | Dickeya | 100.00% | 7574d78535cf01439c091f66c4ebab2b |
| Genus | Dickeya | 91.17% | 63eb165dafac73fd4750e39226a393f4 |
| Genus | Dickeya | 99.84% | 40cc64a6642ba651baad96de90994c38 |
| Genus | Dickeya | 99.81% | f4f3e5874908703de67448156d781f25 |
| Genus | Dickeya | 99.84% | b721b8363bbfb074d39e8e5b595d0e90 |
| Genus | Dickeya | 100.00% | 9aca997e9fcd322d151f2f6f52e46af9 |
| Genus | Dickeya | 96.20% | f38bc63ea39900af42f87eedcdc3cddf |
| Genus | Dickeya | 99.95% | 7139a4d2a01f55a10d3986e039e919b4 |
| Genus | Dickeya | 99.83% | ff83e03aca808cad1c2315758e57b76e |
| Genus | Dickeya | 99.92% | 1e2d1fd258b6a821b6d106b63c5830ef |
| Genus | Dickeya | 100.00% | 58345435d271c27090c083db42367c91 |
| Genus | Dickeya | 100.00% | b4ca12cb117b2e79341b36795c42c261 |
| Genus | Dickeya | 91.52% | cfbe0555f1e2683ae9da7b245bad9a5a |
| Genus | Dickeya | 99.86% | 4cfae0eff8bf8f672219bdf8fbb9c2cd |
| Genus | Dickeya | 100.00% | 30f5964b068160bfea8b0091d2c1aa48 |
| Genus | Dickeya | 99.93% | ea46b24e2e50c39065fb5db5477223e3 |
| Genus | Dickeya | 100.00% | 683818678037bd5f55525345de58d50d |
| Genus | Dickeya | 100.00% | 973db50cc6bae768b9729c638179acf9 |
| Genus | Dickeya | 99.88% | 962053e27528e76cc12c7c7b48ed4965 |
| Genus | Dickeya | 99.94% | 69841f3ec3a98dab0cc99e4f5b777831 |
| Genus | Dickeya | 100.00% | 3f4fae9db70d0e4700f4dc8c16af2ed9 |
| Genus | Dickeya | 99.89% | 8a9632e7bf3ae47c0d6ef34dd0e9c8c0 |
| Genus | Dickeya | 88.93% | 6526a09c1c6b113358e0092e90189a20 |
| Genus | Dickeya | 99.76% | 81efc955d049fc6a18a9d86f56cbf81c |
| Genus | Dickeya | 99.97% | 84a64591b15ad94dad070d434dc28bb3 |
| Genus | Dickeya | 100.00% | 8de8202d6678868cb03a62a3835db52b |
| Genus | Dickeya | 99.88% | 700cfc2e20a09c696aeb13a616653981 |
| Genus | Dickeya | 99.98% | 05bc518f862b368e0598c665fa879e39 |
| Genus | Dickeya | 100.00% | 90626095244ebfb6d15998d5755f77a9 |
| Genus | Dickeya | 100.00% | d5cd67581916f0676c1c2fed998244ed |
| Genus | Dickeya | 99.76% | 0480dae302cf11bc872b5feb50fb8097 |
| Genus | Dickeya | 99.71% | 27653eb2244f4f716763152d198ddd98 |
| Genus | Dickeya | 99.80% | 4d54bef9473a5095e32e576faa46f612 |
| Genus | Dickeya | 99.95% | a546c6906318f1d98d5e61680be2f81c |
| Genus | Dickeya | 99.82% | 264df53f8b4cd0f7fbd2732cdf6c2e09 |
| Genus | Dickeya | 100.00% | a6b8f9b03bbf6529f3b2f0b9710f03e9 |
| Genus | Dickeya | 91.09% | e07f9163006c8eda0f772339f21aabca |
| Genus | Dickeya | 99.96% | 010ac3e3f978b29aee0e9c50c6d95597 |
| Genus | Dickeya | 99.88% | 6ea46cc046eda53821bcdc512f5e8d64 |
| Genus | Dickeya | 100.00% | 75f50cdb33093fff88a44080b218229f |
| Genus | Dickeya | 99.93% | af821b6a057bd5047d35c11858796239 |
| Genus | Dickeya | 99.95% | 0d5781bc42894f47a697894411ee19cf |
| Genus | Dickeya | 99.99% | dd9b991e076fbac7d45028bd5b139587 |
| Genus | Dickeya | 99.97% | 7248aac63dbda6604b93789c87fa772d |
| Genus | Dickeya | 91.59% | a55a52e092abe231d2a454d3f68b08e3 |
| Genus | Dickeya | 99.86% | e8f923090adebcff3f1b7a005f9f56c8 |
| Genus | Dickeya | 100.00% | 84a4427b264f63d5153df1393c21e638 |
| Genus | Dickeya | 99.99% | b640394f63a3b6d1ff92d019b972f77c |
| Genus | Dickeya | 99.73% | 4312a89d144b8777f3616935dbcaa766 |
| Genus | Dickeya | 99.97% | 4830322883c335ebeb9adca32864a498 |
| Genus | Dickeya | 99.88% | 98c81f8b8c226017e0c76317f0e4e46a |
| Genus | Dickeya | 89.91% | 568f29400b76d7da2406fbd4746cf95b |
| Genus | Dickeya | 99.86% | 4345e750853f9d7a212889ff098db517 |
| Genus | Dickeya | 99.99% | 8d41f3efdaf29cdb350313cde9607646 |
| Genus | Dickeya | 99.83% | 1f33a75e67eb657b6cfea1686c3ad3af |
| Genus | Dickeya | 77.57% | 9854ef6e91b0b38aa7fd8395a9372e8a |
| Genus | Dickeya | 93.07% | 259b271db91d661339f79e338fb8ac9d |
| Genus | Dickeya | 99.88% | 82c3048ecafab2861fe38142cef63e3e |
| Genus | Dickeya | 99.86% | 276b0cc1826eb72be435e184f72530f1 |
| Genus | Dickeya | 100.00% | eaea2297cd0442ed8c155e387845dca8 |
| Genus | Dickeya | 99.80% | 6038fe9e6e14631aa70bc9663f068a44 |
| Genus | Dickeya | 99.86% | 31af0a23d7230d271348bf655a4d9596 |
| Genus | Dickeya | 99.86% | cc1b81daa98d031184c897595c68521c |
| Genus | Dickeya | 99.93% | f1e56380fde22100c2b976387304b2e2 |
| Genus | Dickeya | 99.86% | 3c2c6ea574199b03b50cbb7a8f694903 |
| Genus | Dickeya | 92.57% | b2e8a8a434a5ba2895d5cca193fa318d |
| Genus | Dickeya | 99.99% | 55c4111658d88a30d869fb4e551bff04 |
| Genus | Dickeya | 99.96% | 0235216d1f0728d660504b9e6f88940a |
| Genus | Dickeya | 100.00% | 5e2c89bac856b02011f1fa7d8401628a |
| Genus | Dickeya | 99.86% | 6413315a612f6bc29af5c77f24c11350 |
| Genus | Dickeya | 96.86% | 0ae8f19ec9995c66bbef24d657335acf |
| Genus | Dickeya | 99.98% | 7befcdecf464618441187548dbc2aeba |
| Genus | Dickeya | 99.89% | a396dbdeabd4b90c6a29b5a537df2438 |
| Genus | Dickeya | 95.63% | 1b6a5d55065df13c5379edb6368ab5d3 |
| Genus | Dickeya | 99.97% | 11d4b6c7c3b6074d5ee62bb7423fbe30 |
| Genus | Dickeya | 99.93% | 8b296b01525e06ad16f1ed4c1cc5dddc |
| Genus | Dickeya | 99.94% | 32982489e1f4ff57a4f9c0db426e946f |
| Genus | Dickeya | 99.95% | ed5bae3ab681735040d342c480e622c0 |
| Genus | Dickeya | 99.80% | 0796b16f0ca2182e04719cd1ed60d579 |
| Genus | Dickeya | 99.83% | 9bf52a0b60ed34121a6565fe01bc1fd3 |
| Genus | Dickeya | 100.00% | da733a427356420c32cec2d277ac97eb |
| Genus | Dickeya | 99.99% | c2a97075a02260eaa2e0f48adece293d |
| Genus | Dickeya | 99.89% | fd4c82021884ca691de0ed5e8e95cffa |
| Genus | Dickeya | 99.99% | dd096d392a3892b827b449e0f806ee2d |
| Genus | Dickeya | 99.69% | 1b199b8d10f9c8b9551cea856a8bb8da |
| Genus | Dickeya | 99.42% | 382ec016777d73078799a374d58d344b |
| Genus | Dickeya | 99.82% | b1d010de8720511816e252f9c1e7e6cf |
| Genus | Dickeya | 99.94% | a5aaec04d2ac8ed9adce40d9ee3c6c05 |
| Genus | Dickeya | 99.75% | c09dbbde80fa953a95ff989d9a98ac6d |
| Genus | Dickeya | 100.00% | 7784eb8ffca09d3716f92a9869815c9b |
| Genus | Dickeya | 91.52% | 209e1ba443a7b624d6de8fd18ce79c73 |
| Genus | Dickeya | 100.00% | ded9d5d222eb4a596873abf79909d291 |
| Genus | Dickeya | 99.99% | 2d9dc4d80b70ee243d3fc5d2fecfa2d1 |
| Genus | Dickeya | 99.90% | 9606b27d8f97b548c8f4144ae2c54492 |
| Genus | Dickeya | 99.87% | 237682bd9cceeb8a969ff65b2ce8fb08 |
| Genus | Dickeya | 99.71% | 33a4d14cc54fe8be7261842a350e6b9b |
| Genus | Dickeya | 99.91% | ae90720304466d4de94f1574acd74ba6 |
| Genus | Dickeya | 99.96% | 9de5884251cbb69e3135abc917056451 |
| Genus | Dickeya | 100.00% | 89444eadbb44510ef0139eced6552751 |
| Genus | Dickeya | 99.86% | abf277faa32905dc89a8fef5e25f78b1 |
| Genus | Dickeya | 99.57% | 24baefc710f8963337d87b34cb3dfb3d |
| Genus | Dickeya | 98.49% | 04e30317d3a234c8d48b39da48114bd1 |
| Genus | Dickeya | 93.85% | f9fc94a9c592e64e20c6302472135cd2 |
| Genus | Dickeya | 99.94% | 2cc842de0d30a55f540fb34fe8648c4c |
| Genus | Dickeya | 99.94% | c8b4f4423ba229a27be2325149028937 |
| Genus | Dickeya | 100.00% | de84cceef8ae60882177f5978b18d6c0 |
| Genus | Dickeya | 100.00% | fc07cb5f43f8d813bfaf490890ab83fe |
| Genus | Dickeya | 99.99% | 470629befe202dc8dddf885bb0f5da79 |
| Genus | Dickeya | 99.97% | af233d64daefc9443945b4e49295be02 |
| Genus | Dickeya | 99.45% | 023ae9cbc88a0e728cfa4061685e2ccd |
| Genus | Dickeya | 99.99% | 77149bbc124ca803c50695c33fcc0d9a |
| Genus | Dickeya | 99.89% | 7b3c8aa21d79bc5fc60c24a6102fd53b |
| Genus | Dickeya | 99.92% | 199a1c781b51253a799ef800df6bb05b |
| Genus | Dickeya | 95.40% | 0dab017a3f48ebff196b7a3d562731ec |
| Genus | Dickeya | 99.30% | 2079428a226bc8d9074d9b1477398a04 |
| Genus | Dickeya | 99.87% | 3ba597274b6680e855fdae4de0797fb8 |
| Genus | Dickeya | 99.94% | 10db7f9b4a34ef838c1d477fa6da0530 |
| Genus | Dickeya | 99.93% | 746d41569b5ddca1a99bad93cb25a504 |
| Genus | Dickeya | 99.83% | 4c0f2ebb2227ea299925778971615e62 |
| Genus | Dickeya | 99.51% | 8b39951f8f05a340e1f715ef89d17e72 |
| Genus | Dickeya | 100.00% | d0bcaca8b2849c855f6a996b52851576 |
| Genus | Dickeya | 99.69% | 15cf4e52725e133d292e6e2d6e9d407b |
| Genus | Dickeya | 99.84% | ecc68f272b32c7d8a80b4f61e3b42a09 |
| Genus | Dickeya | 100.00% | ddaf0cefb1cc2f1c9e379bd47739e101 |
| Genus | Dickeya | 99.93% | daef70140af4ecefe8680813f17aebbc |
| Genus | Dickeya | 99.99% | 72a2756f99e19e242dc11addd3dbc000 |
| Genus | Dickeya | 99.96% | 9439c31d0d90e171dc5878b2aa754438 |
| Genus | Dickeya | 100.00% | 1dd27485fc9953f81e7f6828c4e071b5 |
| Genus | Dickeya | 92.76% | f318e94b4f0383c788f2c1f8ddcaa078 |
| Genus | Dickeya | 99.60% | 46bfddfd50ca1247768519c97200f4e9 |
| Genus | Dickeya | 99.95% | fae80101d0a0974ffffc869aa121cfeb |
| Genus | Dickeya | 99.69% | aabe2cfa08af08f125637ff3bf5f0a5b |
| Genus | Dickeya | 99.86% | a4e9628bdab1ebf58a03596b26583bed |
| Genus | Dickeya | 99.97% | 2de14dc50df5302a988b35831cefb424 |
| Genus | Dickeya | 99.99% | f428b1f1e564445823e193f90949d091 |
| Genus | Dickeya | 99.88% | b606ffc6b19bbb68ec3a391d313579c0 |
| Genus | Dickeya | 100.00% | feed0eb40cd59e4fc425657e20d93bac |
| Genus | Dickeya | 99.89% | c2f40f2c753f32b2a10e172c8f2a5a2a |
| Genus | Dickeya | 99.98% | 0e9dc5026261fa9aa3cf59ad6a713c0b |
| Genus | Dickeya | 99.98% | 3f99086c9768c207b7242c06abffa5fe |
| Genus | Dickeya | 99.88% | 3bf3e16ef4e7339a8f6863ce726bc21d |
| Genus | Dickeya | 99.99% | c9505be38494543f88478319d29243b7 |
| Genus | Dickeya | 100.00% | c938866a453c08afd3cfe7bd805b41b8 |
| Genus | Dickeya | 100.00% | 07575f3fa5c5e2e628341151797414c2 |
| Genus | Dickeya | 99.97% | f04963984e66f77855dba475fbbad919 |
| Genus | Dickeya | 99.80% | 90141bf33e7977c5d4b7a1a8f770a276 |
| Genus | Dickeya | 99.96% | 68384833be06d70a7ef4217cd5f0fe56 |
| Genus | Dickeya | 99.94% | 66dd0c63f58ee622d53eeee9b4a0bb98 |
| Genus | Dickeya | 100.00% | b54aabcd13d19bc916918ea521bd87f4 |
| Genus | Dickeya | 99.99% | 5a7d154faa3e6769c022a5b7e5c2797b |
| Genus | Dickeya | 99.86% | 69eb1a04d579147ae0a8c008eddc012a |
| Genus | Dickeya | 100.00% | e1a0e2f2a4e1c66c50b134bf81ba6154 |
| Genus | Dickeya | 100.00% | 47e3dbacd06951ecffea525e09a9d24c |
| Genus | Dickeya | 99.89% | 9efd82873b3506bcd26b3900fad7b964 |
| Genus | Dickeya | 99.98% | 52369876543255b9232796f36f9212ef |
| Genus | Dickeya | 99.98% | eea642e34a15f951da20fa7ca10395fa |
| Genus | Dickeya | 100.00% | 09ea2072e342917d9f5084ff9e7e3250 |
| Genus | Dickeya | 99.99% | 1d6851e24fd844f969d2c962dd15bc16 |
| Genus | Dickeya | 100.00% | 2f3f4435b8d470236bdd727345b91c48 |
| Genus | Dickeya | 99.95% | 134bf467c9e90259b4d13be7a3dae2cf |
| Genus | Dickeya | 99.59% | dc4db623e31d5fbbe87b391744f7caa0 |
| Genus | Dickeya | 99.92% | 21636c6884feb754898b4c3c8f34bbca |
| Genus | Dickeya | 100.00% | df0e9175441d755670030c3809ed3379 |
| Genus | Dickeya | 89.38% | 7036250762ad4313c8f1585c60658e3f |
| Genus | Dickeya | 100.00% | fdb04febd66e3365d78f1c8d23ea58f9 |
| Genus | Dickeya | 99.97% | ac53c16fc9662cf5bd6b80a5b35d9938 |
| Genus | Dickeya | 99.68% | 8f394a2dc00fabba6eae50ccf5cbb748 |
| Genus | Dickeya | 99.74% | 6792b85ec8945b2383b2e0c27b9e6491 |
| Genus | Dickeya | 99.90% | a1d353ce8e996ea833966eddd0cf78d9 |
| Genus | Dickeya | 91.77% | 724a4ad558296fe5a1f8f3f0aa75860d |
| Genus | Dickeya | 99.97% | bdc42c26a8fffde52bc6004e9f37c45d |
| Genus | Dickeya | 99.97% | b752498fa83ec8646e1b5faed3091a75 |
| Genus | Dickeya | 91.38% | 1d510f3d4d5aaa1b71d221a8293e38f2 |
| Genus | Dickeya | 99.67% | 76fadc4bab527192586ac184248ba6f7 |
| Genus | Dickeya | 92.79% | b20e0ff1cef152ec82b5f2b518ff2cfb |
| Genus | Dickeya | 99.92% | d624b488b65e773c27a08ca5746015f8 |
| Genus | Dickeya | 99.96% | 39eab7b1f51bc1065ca967074bec786c |
| Genus | Dickeya | 99.53% | f2cf24d8e521109c1269d0a1b6b13e7c |
| Genus | Dickeya | 100.00% | dfb500a032be21d347e07894a2d0687e |
| Genus | Dickeya | 100.00% | 45291f042ecb44d1f8056e9255396c4a |
| Genus | Dickeya | 99.99% | e0c5bd5361bee1fc600cd22d1e27b29a |
| Genus | Dickeya | 99.79% | 3bd0829718ac6ff36bed5f98363784be |
| Genus | Dickeya | 99.99% | 4e4820a987ca8a557382b850e9b9e2d5 |
| Genus | Dickeya | 99.83% | ba2d3279ac274614d17b584adf9838b0 |
| Genus | Dickeya | 99.97% | 998fbd56ec83062ad49e722828ce2e40 |
| Genus | Dickeya | 94.01% | 458f579302892a3575140012de826e16 |
| Genus | Dickeya | 99.99% | e911dcacb58597354dd3725cda4b715b |
| Genus | Dickeya | 99.96% | 0418bc98c5d70ad35c00502b279d81e3 |
| Genus | Dickeya | 99.96% | 9b5678f40bde93dbb6bf2fd159e0bbac |
| Genus | Dickeya | 99.88% | c59647cba1097b1b4c828074877a039b |
| Genus | Dickeya | 99.87% | 667c233d4187e44ccf7d45f47b4861f4 |
| Genus | Dickeya | 99.93% | 871b01f8fd3a9081bc4b6022554b2e29 |
| Genus | Dickeya | 100.00% | 9ea22f67947030ad81c7388617f59053 |
| Genus | Dickeya | 99.93% | 7c793cb9f42483def53da4d167357335 |
| Genus | Dickeya | 99.65% | 626aa878dd91df0f7ec411c2e333d6ec |
| Genus | Dickeya | 100.00% | 5d18863659c7ef00444cf46b46a6f38a |
| Genus | Dickeya | 99.94% | 2d0fdf1199cef99875e5d1f3542d281b |
| Genus | Dickeya | 99.92% | ae48028a694a90748f305659e45deef4 |
| Genus | Dickeya | 100.00% | bfc758b6589c5ffa17060971f3a515a5 |
| Genus | Dickeya | 100.00% | 78496ab817df8309c26fa3646b60df0a |
| Genus | Dickeya | 99.90% | 59a5c6d7086ce9fe4450d0ab79ed013f |
| Genus | Dickeya | 99.99% | 2ba058920cb4f161d5ad10156dd11ce9 |
| Genus | Dickeya | 95.28% | 0d5c303dd83a75239bdb60ccd0506ad9 |
| Genus | Dickeya | 99.97% | 33163e3c5ed5f221cabf280d201228df |
| Genus | Dickeya | 99.99% | 50d1029244a2eaad40eb92bd243ee49e |
| Genus | Dickeya | 99.73% | 13a72427cf393af373ab95e2f17c052c |
| Genus | Dickeya | 99.92% | dc24224b8accb798b4ae6e1101c27fbd |
| Genus | Dickeya | 100.00% | 276906d0d213104bc15ff3cb8b062166 |
| Genus | Dickeya | 100.00% | 8496c83171b1c398bc9583110895bc18 |
| Genus | Dickeya | 99.96% | 20929a9dfa39c7e4202f5cd0f3376272 |
| Genus | Dickeya | 98.21% | d41fe4c12372a55e5dc005f162ee7d8f |
| Genus | Dickeya | 100.00% | f23eb73f3efba4f5b0402f503bba47d3 |
| Genus | Dickeya | 99.84% | fda0cc6896025e217d889df54331b910 |
| Genus | Dickeya | 99.83% | 4aecf871508a755069d11ee02fabcfb5 |
| Genus | Dickeya | 93.93% | 0795f5e1951ce4984885b3ce6d84193f |
| Genus | Dickeya | 94.20% | 0b14fab2938c319db498bb4a456c0acf |
| Genus | Dickeya | 100.00% | 46104596f1dad9bf825586e6c32408f1 |
| Genus | Dickeya | 100.00% | 8b01237e13a01d4e811f0d1890820319 |
| Genus | Dickeya | 99.99% | 133d5a17eea8cd57e3e5123035dbfd00 |
| Genus | Dickeya | 99.95% | 70995886e03861a4542ad0067927a633 |
| Genus | Dickeya | 99.95% | 9ee01dac5bec77a439a57f758a088d04 |
| Genus | Dickeya | 99.95% | bbaaffa634b12a70f84ea209b1e3e942 |
| Genus | Dickeya | 99.88% | e8bc259d44256e7c97f48d11c11ae223 |
| Genus | Dickeya | 99.88% | 0b0d11ab3f046feae38f99f92c19e501 |
| Genus | Dickeya | 100.00% | 49c5f9b41be2ffba4ca7389d03cb69d9 |
| Genus | Dickeya | 86.28% | 5a1db2665dd0372af483aa8cdb0c1641 |
| Genus | Dickeya | 99.83% | 45f7f9d1b169664a9d6cc33c4d997fcc |
| Genus | Dickeya | 99.76% | fd624c4164a7df58eeac465ef416a31c |
| Genus | Dickeya | 99.17% | f7bbc998592423dd9904210b6774b0d4 |
| Genus | Dickeya | 99.99% | 11c8fda7a470114e63766bf53a1607f6 |
| Genus | Dickeya | 99.98% | 4cff67c31454e4738ded23a6d9107479 |
| Genus | Dickeya | 99.79% | 80d7fb640674089d4cf2f655b3847f5e |
| Genus | Dickeya | 96.89% | 326d42d41783ae2afcb6993b28beefe8 |
| Genus | Dickeya | 94.47% | 320d0f77a01988bfe97ad7841ecb26f0 |
| Genus | Dickeya | 95.23% | 54a32d4599affdac8e94abb535f3eab8 |
| Genus | Dickeya | 100.00% | 74275d6fd084ab938fc2119c9aa1a935 |
| Genus | Dickeya | 99.85% | 8ac4fb8a7163303986dd99b653d3b6ff |
| Genus | Dickeya | 99.99% | 21dad2aba53890ab10eb538e17c3debb |
| Genus | Dickeya | 99.99% | 1238d897cdfe51e68223b784ee570a11 |
| Genus | Dickeya | 99.83% | 09ee6a765e0d1d096a9aadd058e6d96f |
| Genus | Dickeya | 84.02% | 8e110ba2969adc571e781499a2e13484 |
| Genus | Dickeya | 99.90% | 904c8e7d099e2a2c33307783f7a21834 |
| Genus | Dickeya | 99.66% | 9e5842bff5b139322ac3c39c3a3f0033 |
| Genus | Dickeya | 100.00% | 1eefbc6ed25515764048e8f46cfd8723 |
| Genus | Dickeya | 100.00% | d49f2e81623590f4111bee2c078dbd17 |
| Genus | Dickeya | 99.93% | 69a22c26a662f23cac1eb1d87fda3d61 |
| Genus | Dickeya | 99.93% | 47ddcace42c246ca563d312192c0dedd |
| Genus | Dickeya | 99.94% | 9603527e7e02ec7d498ccb6ad07b6fdc |
| Genus | Dickeya | 100.00% | 6faf108e85c9564cccfcc5803f3049e8 |
| Genus | Dickeya | 99.85% | 9cc4309a648d5aea5980af9d6b65f941 |
| Genus | Dickeya | 99.98% | fbc8e8558d901289a19a07603a11c58f |
| Genus | Dickeya | 100.00% | f009374421b948e62d13f1342576d4ca |
| Genus | Dickeya | 99.91% | 5fcdbdea69656f955e010045ad3ce90f |
| Genus | Dickeya | 99.91% | 1c9de497867ebe44c9e1132717a6cd5d |
| Genus | Dickeya | 99.90% | a4f0df737f071b3fada46a1f5bdde58d |
| Genus | Dickeya | 99.89% | 276f1936fb215640d12f73cea11ca345 |
| Genus | Dickeya | 100.00% | 8b6c2f73690933b4a90be19510147de0 |
| Genus | Dickeya | 99.99% | cc9a59b481e92664a23a2f930ad337ff |
| Genus | Dickeya | 99.99% | 20392d0f464ebbfc5c31d21eb7a538b3 |
| Genus | Dickeya | 99.99% | b9ad5207ee43ecafec327406539a14ef |
| Genus | Dickeya | 99.85% | 5bb11d5e1311ca4b68b3fe262acbd569 |
| Genus | Dickeya | 100.00% | d7a30256b592f48a3d84df1211ce8d95 |
| Genus | Dickeya | 100.00% | f710c8f7b6a6547ae9c4c9e8e1be4aa7 |
| Genus | Dickeya | 99.00% | 8524041cbc8b572fa8b2553b9ada5cc8 |
| Genus | Dickeya | 99.91% | ce90113c6221a68d35bcda9f35099ee4 |
| Genus | Dickeya | 94.87% | 7258afa4b11a9253445c87869c339924 |
| Genus | Dickeya | 100.00% | 75c2759f77d945d6d88c0853753afaa9 |
| Genus | Dickeya | 100.00% | 6e8e3a8d5b5c76e13f7482c2bacf4fd6 |
| Genus | Dickeya | 99.97% | acf8e12b875d0d47f210573ba230ad08 |
| Genus | Dickeya | 99.96% | 85eca169d1a2bfe8f4b69d7cd6c4d686 |
| Genus | Dickeya | 99.74% | b1a1f21fb9defabb39fa7d632faf12d8 |
| Genus | Dickeya | 95.17% | 1c6e6ccd0ee01cb4bbac630d30beb1bb |
| Genus | Dickeya | 99.93% | b64f26b9d54a1582551383257dfeb30b |
| Genus | Dickeya | 99.99% | 87c7ab8447ca6122ed90dfe363fbd255 |
| Genus | Dickeya | 94.05% | df65036baf3f35b65779c069689095a9 |
| Genus | Dickeya | 99.76% | 7b25169d0d4366b9034d18ad72663304 |
| Genus | Dickeya | 99.98% | ce449a98938395466a9f4d1232cb75cc |
| Genus | Dickeya | 99.96% | 2728809c11ad450c99e2f642d0de0dd9 |
| Genus | Dickeya | 99.98% | b41be59e5676f5a0d794d5a8f996e853 |
| Genus | Dickeya | 100.00% | 5a224518418621969aec9dab5430ef32 |
| Genus | Dickeya | 99.86% | b06f3c85deb96476d31177aec48fb114 |
| Genus | Dickeya | 99.85% | 67d845b3b679272fd4220b802f9d3b9a |
| Genus | Dickeya | 99.95% | 9bd0394f7c05955167f6b2a91793412d |
| Genus | Dickeya | 100.00% | f22b3de8dca7e4203651549abb740448 |
| Genus | Dickeya | 99.95% | 56806ad6dbfecd242db100918efa1ac9 |
| Genus | Dickeya | 99.92% | ed73742ef4cd4097d1db54728043c643 |
| Genus | Dickeya | 99.98% | ce779abeacd02fe5b6039762b517a31f |
| Genus | Dickeya | 99.99% | 04dc35adecfe871394bdaacd8c3e909f |
| Genus | Dickeya | 86.88% | 2f06880c9529baf89fdab86818479112 |
| Genus | Dickeya | 90.43% | 0a1c5bb55c9ab8cbb2b7193b9db9029b |
| Genus | Dickeya | 99.96% | 6ba7766f4a50908cb4ec89f526588574 |
| Genus | Dickeya | 99.80% | 6d09577ecc2f4e41b0054b38cb9d5f33 |
| Genus | Dickeya | 100.00% | 73e1ab55d8d94cca2583bb04dadad4a3 |
| Genus | Dickeya | 99.97% | 48fc8e7dbc0238376fc74b6685e19a2d |
| Genus | Dickeya | 99.80% | f9fd18559b6f60fdf2ce05ab32c83df7 |
| Genus | Dickeya | 99.98% | 11a5caab7f7836db561b0558063586d8 |
| Genus | Dickeya | 99.64% | fd5609facc09d3664792867bfdf7063a |
| Genus | Dickeya | 99.98% | b8e8bdeefba696eed09d49b95a560a51 |
| Genus | Dickeya | 99.98% | 5133106498d43302c78b31b487b5db0d |
| Genus | Dickeya | 99.58% | 83a389144deeaf5d0f60a6acc3fbc018 |
| Genus | Dickeya | 99.98% | 6f52372697024a6aea6be12780dbb23a |
| Genus | Dickeya | 99.94% | 4e7d257ec42028d268c0ea8a9d56455c |
| Genus | Dickeya | 99.94% | dd4b5b98544088812a25aefd65a1dc74 |
| Genus | Dickeya | 99.94% | 94f942330bf1f194f37657379089d3d4 |
| Genus | Dickeya | 100.00% | e4455f87131d2699d1ee3e64569539fa |
| Genus | Dickeya | 71.53% | 1fe49399e0d3ebc8d8e2a4aa4ab9baeb |
| Genus | Dickeya | 99.98% | 97223ac01daf2638e9da13baf4cc40d8 |
| Genus | Dickeya | 99.89% | e60637994f291a3555c2822811ba407c |
| Genus | Dickeya | 99.86% | c0fa5f2eb28e631b5c87b8032512c7b6 |
| Genus | Dickeya | 99.86% | 4ed67016f5129fa3b4bc2993d7820600 |
| Genus | Dickeya | 99.99% | 9ff387fd431bc92fb2d0d8afe252f461 |
| Genus | Dickeya | 99.99% | 6c11d260b2fff65307f1f4cab1a7b81f |
| Genus | Dickeya | 100.00% | 96ed031da651525c7ca1a9f4ac54417c |
| Genus | Dickeya | 88.89% | a79c5ff94961c9039e09970bc88f39c3 |
| Genus | Dickeya | 99.97% | d881baa5fa26bcdc78fb76319c92af9c |
| Genus | Dickeya | 93.96% | f48f824f9be01763f1eccaa7f6d9b09a |
| Genus | Dickeya | 99.90% | ff0971bad13ed2d4ed655def7493ef51 |
| Genus | Dickeya | 99.92% | b417372320e91886cd07f0ad3dabf158 |
| Genus | Dickeya | 94.37% | f331c0ed0ef015a0e85be2486cdce640 |
| Genus | Dickeya | 99.99% | 03e0d2b2d6b20f3442ababfa131c946c |
| Genus | Dickeya | 99.99% | 129ac040fffbb78e5926e4428264186b |
| Genus | Dickeya | 100.00% | 5c68f36988032dcacaafc4c4d16ed548 |
| Genus | Dickeya | 99.98% | 0f64f757067e8d06d3ef72cfeda6a9a0 |
| Genus | Dickeya | 99.99% | e68a84c40b79afbd9eff8e9d88376f48 |
| Genus | Dickeya | 99.97% | 36790c26248614d8eb7fa2fd543be846 |
| Genus | Dickeya | 99.99% | 2334bdaaa0c351f4e445efb3a227f425 |
| Genus | Dickeya | 99.98% | d6a33e35ec01515b2828b0411184ebf2 |
| Genus | Dickeya | 88.58% | c6014063f89a7888440cf9dac7642ff2 |
| Genus | Dickeya | 99.99% | e03f677e7275f69a85b02e48b3bf427c |
| Genus | Dickeya | 99.04% | ec95ff66a62507de6be69d7e110bab88 |
| Genus | Dickeya | 98.69% | b7be1fb60786787063928da24832c4fa |
| Genus | Dickeya | 76.31% | dba7d522990bbc56c7cab72b28e28b4a |
| Genus | Dickeya | 99.94% | b7ea2a851415b124b110100ad1cd261d |
| Genus | Dickeya | 86.40% | 9a1a3192412a81cb1db70a36e175cdcb |
| Genus | Dickeya | 99.99% | bf1ff413852314e263ae8ed2eb67e369 |
| Genus | Dielma | 100.00% | dac205799b0ad5641d6a2b7b7dcb571b |
| Genus | Dielma | 100.00% | 271cea33907e220a6a58881956c6d6c1 |
| Genus | Dielma | 99.99% | 69000761b5693464fbba1b02d73e494e |
| Genus | Dielma | 100.00% | 7a8267f1a4abf921a9f5adc37dec9639 |
| Genus | Dielma | 91.56% | 7b96c2e3b5449f4ffe423714f466a0f0 |
| Genus | Dielma | 90.09% | 36462718e964179421955fc4f38c6305 |
| Genus | Dielma | 100.00% | 5cab4ae9b29d44b0da514a960c172f68 |
| Genus | Dielma | 92.88% | 9bffbc2c0520aba127ec848c60e9a6f0 |
| Genus | Dielma | 89.60% | fe3468e69d036be5057be13428487e77 |
| Genus | Dielma | 97.45% | f69298c21a8f20e6151867bfe59994c9 |
| Genus | Dielma | 97.26% | 1b471006dd75b950c25995a09547015f |
| Genus | Dielma | 96.94% | 5771856859c02f99e59c9cfa6295a18e |
| Genus | Dysgonomonas | 88.11% | 01d040b35b56353a0cfeb8d31f7d4c3d |
| Genus | Dysgonomonas | 74.17% | 90426eeec2c113dab8f7ca52b85094f1 |
| Genus | Dysgonomonas | 98.64% | 9e15adf70bd7d7f4beaca089e92483fa |
| Genus | Dysgonomonas | 89.09% | 1ed6c49e2f49f10e18aea60d5ef1f8d7 |
| Genus | Dysgonomonas | 99.70% | 6334efee75bb6c3f80941c845dc345b8 |
| Genus | Dysgonomonas | 88.18% | c3a431dec91a03fd6a9d231b98c91f8b |
| Genus | Dysgonomonas | 88.94% | bd6c35c03f182789ab2cd8c7866b6576 |
| Genus | Dysgonomonas | 89.08% | 1c22aad3cd9a664d9029066333bcb8fa |
| Genus | Dysgonomonas | 73.76% | c732e5b81559ab1126aa20640196a0dc |
| Genus | Dysgonomonas | 98.80% | ed8ad0010da17a17749d7c0494bd1882 |
| Genus | Dysgonomonas | 98.94% | d1e5292ef0e778185c087554e7d3be2e |
| Genus | Dysgonomonas | 99.06% | ae60f993f9c02c576084b93ccf38e12a |
| Genus | Dysgonomonas | 98.93% | fc26ba8472fabe9a989150d35db7e9a5 |
| Genus | Dysgonomonas | 98.94% | d76410bc47fbda84d02aae9ff0d493de |
| Genus | Dysgonomonas | 98.93% | add2321d9a47b9d7d58970b08d4a194f |
| Genus | Dysgonomonas | 86.28% | f65c92c3343b0a4a0fa6a40e93bcf6e2 |
| Genus | Eggerthella | 100.00% | 270f742af29205a40f9fa99a103bac11 |
| Genus | Eggerthella | 99.99% | d1a37e5595526024d01cd9ee6b7e38d9 |
| Genus | Eggerthella | 100.00% | d4713216182eae3201ade7a0c26cf0cf |
| Genus | Eggerthella | 100.00% | 323ca59b3f61b22727701cc4beb14749 |
| Genus | Eggerthella | 100.00% | 66a2e1f4865e9c5d9af909e8f44e9b71 |
| Genus | Eggerthella | 99.99% | 39a32dd012d38a7318dd9c663a7dbeb8 |
| Genus | Eggerthella | 100.00% | db765ca367b5761f44b0e725b7bad039 |
| Genus | Eggerthella | 100.00% | ff5a8625d29644d041e9ec7ef0153b42 |
| Genus | Eggerthella | 100.00% | 41e6bd2a550f40ddae725eb889d91642 |
| Genus | Eisenbergiella | 75.88% | 281077649c21d6b07e3898cdb459217d |
| Genus | Eisenbergiella | 78.72% | 32476a4f601373e914b8e00a4f2751d1 |
| Genus | Eisenbergiella | 76.58% | 2907d941d05a5b758b217376b234a5b7 |
| Genus | Eisenbergiella | 77.28% | 8302dfe7f464e77172b4addceefa2a40 |
| Genus | Elusimicrobium | 99.41% | 0d1da22df72f6ce55d6a733806058988 |
| Genus | Elusimicrobium | 99.31% | 7237baff3db9d2af88d1fad2e462a521 |
| Genus | Elusimicrobium | 99.81% | 683a24a5dd8206aff0d29d0ea70620e5 |
| Genus | Enterococcus | 99.09% | 7c8a1b0934ddf54f3121684e875d8fd2 |
| Genus | Enterococcus | 99.13% | 75e59028ca7533680c1f073ee9b9cf57 |
| Genus | Enterococcus | 96.62% | 4359556e54cc7b9ae941236be156cb43 |
| Genus | Enterococcus | 96.80% | 2b67ff6106da718a83ad328dd8f7e491 |
| Genus | Enterococcus | 99.09% | c2a62564a350333e75b73119031302cf |
| Genus | Enterococcus | 99.13% | e52e7157d0ae7c989a5d9426873c2afb |
| Genus | Enterococcus | 99.06% | 0b2d7172a171a7c000dcdbae96305003 |
| Genus | Enterococcus | 99.06% | 960cb26dfbe0bfaf52b264ce2290ae40 |
| Genus | Enterococcus | 96.86% | 89c6934f4eaf82d43f716c972573d912 |
| Genus | Enterococcus | 99.07% | df3f5b705e223385e1395abd6a1f59dc |
| Genus | Enterococcus | 96.79% | ec7d870f02470bfa603011a1e6c67260 |
| Genus | Enterococcus | 99.07% | 79e7e0ca50b20754a8bcf69ff04ba661 |
| Genus | Enterococcus | 99.11% | d2bd3ba3b6b51b48528a8ca2e6ffcfd4 |
| Genus | Enterococcus | 99.11% | d1531fc83c02949ff5fd3baddd22dd13 |
| Genus | Enterococcus | 97.33% | df5e4139febec3bce7caab8f3d8a9be4 |
| Genus | Enterococcus | 99.07% | a6c53625e46331b563a04c0a2c33993f |
| Genus | Enterococcus | 99.09% | 33920b57316022a972bbd3fff241c836 |
| Genus | Enterococcus | 96.78% | 7f9e12d7fbdda6051574834551c17f09 |
| Genus | Enterococcus | 99.09% | e8d456e28c9ae8188684883a5af7f9e4 |
| Genus | Enterococcus | 87.28% | b086edaf222eaeb31f68f543b76395d6 |
| Genus | Enterococcus | 97.00% | 52659c27c94ce14e3484e4cece3374eb |
| Genus | Enterococcus | 99.07% | 291fa011e950a7c398944404f435f8b1 |
| Genus | Enterococcus | 99.11% | b029ff91be16989f66f31bf4eedd67f3 |
| Genus | Enterococcus | 99.16% | 4647d0a778a8d323ad338cfda98211da |
| Genus | Enterococcus | 97.73% | bdb496d6063fa348d960b67389e55062 |
| Genus | Enterococcus | 99.13% | 29bae2ab57763a19d5840e51ea0795b4 |
| Genus | Enterococcus | 99.14% | 5d620f55290235d8a3fea41df232964a |
| Genus | Enterococcus | 99.04% | c75e0a357fe6395f80cdbefc5e6c0c79 |
| Genus | Enterococcus | 87.34% | 976343b999840f03f946723da5e82d15 |
| Genus | Enterococcus | 99.23% | ec69829ba88a5973a9db7503cfa6c54d |
| Genus | Enterococcus | 98.92% | 579ce5bbc434e996a9f1b622ee0ebd68 |
| Genus | Enterococcus | 97.74% | bc212c6325cd6569df359050a4cb7c07 |
| Genus | Enterococcus | 99.09% | e4510b58d8c1b97d96c73d7ae612c8dd |
| Genus | Enterococcus | 99.11% | de7130830f9f9a188443963cf6604ead |
| Genus | Enterococcus | 96.79% | ad261f9450069e7ebe3ed8edf41924e5 |
| Genus | Enterococcus | 95.52% | c8a2f4c4d7fa2f647e9fe0d66aede8de |
| Genus | Enterococcus | 99.09% | 0fe3ff39f8eda6357d66afcae06e42eb |
| Genus | Enterococcus | 99.13% | a9c86ccc131ff1df14fe9fd3e5b1c7fe |
| Genus | Enterococcus | 96.62% | c466eeb914a7bdd04dbcd27c19574797 |
| Genus | Enterococcus | 99.07% | 656eb7b0ac9129ecece890617aed0397 |
| Genus | Enterococcus | 99.11% | acb6a9833479897bac9ada60e9c4fe04 |
| Genus | Enterococcus | 88.14% | 5e4eda748e7f4aab3940a690541399b0 |
| Genus | Enterococcus | 91.89% | 6e809c89f0c16314827427e4f87590d1 |
| Genus | Enterococcus | 99.06% | c34d17778a8ffc7fa4617d7409a873b8 |
| Genus | Enterococcus | 98.84% | 39570ab3034f7e47a315fba716ea3809 |
| Genus | Enterococcus | 96.27% | 5eb8467559c8cc8b27ae3e0d2d38a275 |
| Genus | Enterococcus | 98.83% | 9c4c64f955f9a6f023f539bd6446e05e |
| Genus | Enterococcus | 98.90% | af9da952db45a390fe8f72bf9d9d9ff4 |
| Genus | Enterococcus | 98.81% | 6f5f274326e7c13529309a46fb8d0d1a |
| Genus | Enterococcus | 87.62% | 6be8fd56cfdd3bd2825ec4a77cf3ab66 |
| Genus | Enterococcus | 99.11% | 6905cf572bfd93b9534b563b6b850f07 |
| Genus | Enterococcus | 98.81% | be1e533cd88bad06b5c88eefa848e091 |
| Genus | Enterococcus | 98.84% | 92db07a25f9315f30d28f2907fb3885f |
| Genus | Enterococcus | 99.11% | 079efab49f94e0017be6efbd377b6a7c |
| Genus | Enterococcus | 99.16% | 3989863bc472f9732a18539d4785a5cb |
| Genus | Enterococcus | 74.25% | 08ffa7545959e64b03dc916fc32b4ca0 |
| Genus | Enterococcus | 96.69% | 42023f34e75cab5352e5a0b87e81853f |
| Genus | Enterococcus | 99.10% | dd4cd486961640212614a0f05d06158e |
| Genus | Enterococcus | 98.81% | f4c7728a39ead64501414a4953ec4481 |
| Genus | Enterococcus | 99.09% | b42923e9f961e93eed498dde8ffad07e |
| Genus | Enterococcus | 93.91% | 65f305c3cb6ab9f84c45d5e5422919d4 |
| Genus | Enterococcus | 96.51% | f1ddb4120ebe0f352d3971ab737fac2c |
| Genus | Enterococcus | 96.65% | 61725b727b394479ae15b20764f88b49 |
| Genus | Enterococcus | 99.09% | f65b836d1d5976d3e4f844266fa77ffb |
| Genus | Enterococcus | 99.15% | b34919286645cc5fdf6cbc2be5062c17 |
| Genus | Enterococcus | 99.13% | b6efde00b1bd1aa74a7fedf4281aa5ac |
| Genus | Enterococcus | 98.82% | 4bf72a4ecec6d875ee3a96b247aaadd9 |
| Genus | Enterococcus | 99.17% | 87f3f5e223d2ee6f567e465849f5b952 |
| Genus | Enterococcus | 99.11% | 204bf640526264774c513bcaf4e88069 |
| Genus | Enterococcus | 76.98% | 760335fde95b6d9ea15f5488d35d9345 |
| Genus | Enterococcus | 99.10% | 3fa0db4c9271ebfeb3c2807a466b4884 |
| Genus | Enterococcus | 96.96% | 03c0c58bc2b9ef4480c1e03ac1b2530d |
| Genus | Enterococcus | 96.79% | 58cacb216c13b4e67cab9369cc0d2f2e |
| Genus | Enterococcus | 98.86% | 2fff80249913a00bcd0155acef364ba1 |
| Genus | Enterococcus | 99.13% | 277b16e597ad122c62f92e7d71400157 |
| Genus | Enterococcus | 97.83% | 8f381490147a7f6fa97ca3e77d703805 |
| Genus | Enterococcus | 99.18% | 03b2303b2db0357b70716a9bc690fee5 |
| Genus | Enterococcus | 98.81% | 3d43025fef1548eb1c21ac5a95c7870b |
| Genus | Enterococcus | 86.26% | e46be93d65c1802696c7204d5e9d49cc |
| Genus | Enterococcus | 99.07% | 5bda78bbc77879bc60274fe1fb736bc0 |
| Genus | Enterococcus | 99.15% | 77dc552f3f9bbdf199420d7b04321789 |
| Genus | Enterococcus | 99.13% | f32649c96a329cf7c779690e32b0a81a |
| Genus | Enterococcus | 96.90% | df115ebe23278ab3d342fbebc1da546b |
| Genus | Enterococcus | 89.90% | 8db7d833f733e7834575627fe37a2c6a |
| Genus | Enterococcus | 72.85% | 4cf8357ef6bc88ce299eee1ff3852c4b |
| Genus | Enterococcus | 90.06% | a3d7a1c4a50e4fc74eef2e2c9136c4ce |
| Genus | Enterococcus | 91.11% | f6f0fd0151d7306077b9c7c66a17db42 |
| Genus | Enterococcus | 96.61% | c86ec17462b8666c8d6768b3c895e250 |
| Genus | Enterococcus | 87.75% | baa05dcedd7cb10772724f1ed6c913c6 |
| Genus | Enterococcus | 86.38% | 7a1f7bdbac8fdf6824f1acb553a6e1ea |
| Genus | Enterococcus | 99.15% | d44bd20727de9f50cd492963f59c435a |
| Genus | Enterococcus | 98.48% | ebe9b5898b46efba36ef3f5efdac77fc |
| Genus | Enterococcus | 98.23% | 8b93161b93de510f74ad8d382f631f13 |
| Genus | Enterococcus | 99.32% | 59e6965eff97f116c1a7373da85a7957 |
| Genus | Enterococcus | 97.23% | 99b5e1020ef7c63bb0a3fd1f0a639a05 |
| Genus | Enterococcus | 97.08% | 0fc67ee6684317be2806bcf56b06ab17 |
| Genus | Enterococcus | 94.69% | 817b68e27e2d9bcac93eeaa702d05887 |
| Genus | Enterorhabdus | 87.59% | a76a155ab862fdc1ccbff5cabeba543f |
| Genus | Ereboglobus | 80.50% | f8b2687877b6006eca387e7a1eaec49c |
| Genus | Erysipelatoclostridium | 99.68% | dea1056dff1df961bfc132e1b928bcc2 |
| Genus | Erysipelatoclostridium | 100.00% | 062b6887f0c9c480bd90b4ee1e0027d6 |
| Genus | Erysipelatoclostridium | 100.00% | 5ee0bee029241f152f6fa92308b58f77 |
| Genus | Erysipelotrichaceae | 92.38% | 6d077e00b201df5a124accd74713ff1d |
| Genus | Erysipelotrichaceae | 95.62% | 691013a1c06cf084a6ae3f8d18892734 |
| Genus | Escherichia-Shigella | 74.56% | 8f47d2c8c24224c448b404368f4803c8 |
| Genus | Escherichia-Shigella | 96.19% | 64a44e3a497a7eb7c84797409671a696 |
| Genus | Escherichia-Shigella | 97.47% | c66b2b6df5c8a02ddacfd754ce0b3480 |
| Genus | Escherichia-Shigella | 98.43% | b571b0daf8c7ce7a814d611ab1b6ffd1 |
| Genus | Faecalitalea | 93.40% | 5c01dcfb700747770d5ee06bbf33214d |
| Genus | Faecalitalea | 91.75% | 74fe1c04077b93d4c9376ff12f71b60d |
| Genus | Faecalitalea | 99.69% | 5954ed9331f0e4fefb17ea5d6879d91d |
| Genus | Faecalitalea | 99.69% | 36c7c6586bf8d12b78b0d5b30d84bb80 |
| Genus | Faecalitalea | 99.67% | 1ffc8d8662911849cb5458ab72e755b2 |
| Genus | Fusobacterium | 100.00% | d8d18fcd2755ef7ee6732a4cf80518ab |
| Genus | Fusobacterium | 100.00% | df8e350203d920bf14c7fb8f7868d3b8 |
| Genus | Fusobacterium | 100.00% | 59f796ba5059e44ece88d013f030f75a |
| Genus | Fusobacterium | 100.00% | b506a4db4c74ef38ac2783d8c1fc82e9 |
| Genus | Fusobacterium | 100.00% | 6ad1495ac766c6110d057f0bcdf376af |
| Genus | Fusobacterium | 100.00% | 146c2453ac01db908862f43ad831fe34 |
| Genus | Fusobacterium | 100.00% | 6ff8b063f90481e227583efb95bf1dfc |
| Genus | Fusobacterium | 84.97% | a1d8bd5262883ec7fb31499f1f0a5b46 |
| Genus | Fusobacterium | 100.00% | 090ec1dc41903365e901a09edfca74d4 |
| Genus | Fusobacterium | 100.00% | fb8617f87747129ecf050987ce81c863 |
| Genus | Fusobacterium | 100.00% | cec9d2eb84736df6e872f8888866ee42 |
| Genus | Fusobacterium | 100.00% | 27af247dae9c3acee576271c47199e48 |
| Genus | Fusobacterium | 100.00% | 1a67dd1c2fff47a32a945a742d81ce21 |
| Genus | Fusobacterium | 100.00% | 8c24e58d791df357136cbf3e5f138d48 |
| Genus | Fusobacterium | 97.62% | ff46cdb45e5026e3487c47fce031b609 |
| Genus | Fusobacterium | 94.98% | 2ba3c9cb306771d73a332c84e5dc88c9 |
| Genus | Fusobacterium | 87.08% | 32dfd2849501fa0c44aa8743c965981b |
| Genus | Fusobacterium | 99.71% | cbc01c1538d5b91e95309f9866b10ded |
| Genus | Fusobacterium | 99.60% | 248ce46047b43f677e8be9fc5a9001f3 |
| Genus | Fusobacterium | 100.00% | c262098520676f761738906277f56425 |
| Genus | Fusobacterium | 100.00% | 69a102bb358f7ff7f13c3895206e97c0 |
| Genus | Fusobacterium | 88.83% | cf0fa1342194fc1e7864d63cc6ceacda |
| Genus | Fusobacterium | 100.00% | 51d52ae37c367ba656a76ef82923d0f8 |
| Genus | Fusobacterium | 100.00% | 0b68e4c3bc80e14477fefd24cd62654a |
| Genus | Fusobacterium | 100.00% | 0a2834acf4abccb58a5d47c863ab6725 |
| Genus | Gastranaerophilales | 100.00% | 45a2e549024608885814a2f7e9ec750b |
| Genus | Gastranaerophilales | 100.00% | 5d1aab392606694e2fd866e79896b2c8 |
| Genus | Gastranaerophilales | 100.00% | c2570a49796e1a54141ba2fb0d7366ed |
| Genus | Gastranaerophilales | 99.97% | 89ae03fcfbb7dd268c2ea72c2e1512ae |
| Genus | Gilliamella | 83.38% | 91e317cca1a089fe44cf3cb4c321502f |
| Genus | Gilliamella | 80.22% | 913c6765900616e2147e97f8b2fe90af |
| Genus | Gilliamella | 77.08% | 623d0d7bc1878089d1798dfed51bd5ea |
| Genus | Gilliamella | 75.96% | 18983c738f849c6e6fce2cc08a22e433 |
| Genus | Gilliamella | 70.07% | e63e6691911d637d07bbbf7819c5f60c |
| Genus | Gordonibacter | 96.63% | 387b23cab9d9774fffbcafd8ff7e4674 |
| Genus | Gordonibacter | 78.64% | 79e4e4e827f6977c63d45931b426661b |
| Genus | Gordonibacter | 98.45% | 07c8250d400da0f2089036e7fad8d45c |
| Genus | Gordonibacter | 97.59% | 702a56476a3bffe5e5b3bf4f3571e329 |
| Genus | Gordonibacter | 99.99% | 8c55bbc1bcf0535a28f1182a7be069d6 |
| Genus | Gordonibacter | 98.67% | de5cf3b4712ebe42c957974f3a61bc90 |
| Genus | Gordonibacter | 97.57% | ea8676a7aa195d75f8c0d39f8c83cc60 |
| Genus | Gordonibacter | 97.59% | dd4b2fcb9db3353d4d0e21e9f2202794 |
| Genus | Gordonibacter | 96.15% | 7fc814a73e8b8c87ede5213be25a0755 |
| Genus | Gordonibacter | 98.03% | d92b0e88c87f9cb81b403ec370588d6e |
| Genus | Halomonas | 80.14% | e4dd77e2dc6224b1181b2a436de0a1d0 |
| Genus | Halomonas | 80.05% | 2aec735001d67636017d54cce4560b99 |
| Genus | Halomonas | 81.28% | 4b4c087cb5c3d3f59c149d7bb833332b |
| Genus | Halomonas | 79.80% | a250df30c276ff240e7208ea3649062e |
| Genus | Halomonas | 80.14% | f2885a4af47dbf047bddeba62cefcaa4 |
| Genus | Holdemanella | 81.41% | f01f0d4364cdc8ff3b24ad232594c355 |
| Genus | Holdemania | 100.00% | ad73556540e59c84d634bf64d7ad85f6 |
| Genus | Holdemania | 99.98% | fb28fbf8506b88a5c9b157734cf91178 |
| Genus | Holdemania | 100.00% | 8a88e4c68fc83c930206fc0492252d7f |
| Genus | Holdemania | 99.98% | a0b35755b89330e12ef266b9ae280fae |
| Genus | Holdemania | 100.00% | abcafb349dc55716ff4e34ac6d2fd40f |
| Genus | Holdemania | 99.98% | db3e180e3cdb2c273f05b404bfbf7347 |
| Genus | Hungatella | 74.75% | 6b1a990ebb6c54c8988ea4402a17e7bd |
| Genus | Hungatella | 72.25% | b8a5f3ba1a95e2d4aeed3820bcae37d7 |
| Genus | Hungatella | 74.78% | a4f879a04e6746929b8e115201de074b |
| Genus | Hungatella | 75.86% | a3e6a19fd94ade812252b045b561f606 |
| Genus | Hungatella | 71.58% | 284dc1451fe045e29178e26440c8afd7 |
| Genus | Hungatella | 74.56% | 756d7f3281d4775a6f030cda635432e0 |
| Genus | Hungatella | 72.82% | 1f7efcf6074e0ca0761c3436e4c1c6a9 |
| Genus | Hungatella | 75.40% | 475308f26a11a51d69dea07f48043e77 |
| Genus | Hungatella | 75.74% | 21be30b196456721a9ee909b559a22de |
| Genus | Hungatella | 73.58% | d314d9e474663519bb7b424a5053e10c |
| Genus | Hungatella | 73.52% | 6d4d1664730ca1e14b40e1fba004ea9c |
| Genus | Hungatella | 75.96% | ac5ee6a4866e1d5557fbad25fb5a4f2b |
| Genus | Hungatella | 71.46% | 4ea83274aaf7d6d7523cacffdc1b9aa8 |
| Genus | Hungatella | 73.95% | ed6a5219224d8de551d2f4520ffc87f7 |
| Genus | Hymenobacter | 100.00% | 4cd94cc4f735931bbfeb82edce6fb30a |
| Genus | Hymenobacter | 99.86% | d54206c8976b159f9dc5a49539d96d90 |
| Genus | Hymenobacter | 100.00% | b512598d30cf9b62f022e754c8a3b815 |
| Genus | Intestinimonas | 84.18% | 146f7bdcf22eab72485d9e15efb5956a |
| Genus | Intestinimonas | 80.89% | 8632a2887c7e9803f8e476bf800c3bb4 |
| Genus | Intestinimonas | 76.63% | 3f063da96e84b6e64e5db363921340fc |
| Genus | Intestinimonas | 73.01% | 7265a04da8af15c3948665b691ee499e |
| Genus | Intestinimonas | 81.24% | 9a10abeeb76da3d232e3fdb9aea9ff12 |
| Genus | Intestinimonas | 74.17% | 2216743061c65a5346f012cd62cb6619 |
| Genus | Intestinimonas | 71.65% | 9ef20b1795c00263943de8830ae754b5 |
| Genus | Intestinimonas | 83.07% | b48bbbcbb392e2a66c7e15a1d141882c |
| Genus | Intestinimonas | 73.19% | a04cc49289f8cb267fae107fb946fc46 |
| Genus | Intestinimonas | 74.09% | 66a4e10f321d454a2b7487321dee3d2d |
| Genus | Intestinimonas | 97.87% | d52e4ba72b7d78f09c2ea54aa01a18a5 |
| Genus | Intestinimonas | 84.19% | fb75ef649035477afcfc8b1f3922063d |
| Genus | Intestinimonas | 98.02% | 3d227d86c6a50e7200e9e75f3d0dce6c |
| Genus | Intestinimonas | 98.05% | 1663186fbdf048f24b4e22e8ec0090c2 |
| Genus | Intestinimonas | 78.22% | 101c447a44ded319b7d8bb87f11175e2 |
| Genus | Intestinimonas | 82.26% | 6ab1eff1ff8c613c6fd53bed7f2ea555 |
| Genus | Intestinimonas | 97.03% | 8106dc55de89b4b04dd8470fb43095a9 |
| Genus | Intestinimonas | 78.22% | fe4090aee084f2d4d6ea278f9bf6468a |
| Genus | Intestinimonas | 78.74% | f60a2516e6fe9693a3da49e053860074 |
| Genus | Intestinimonas | 86.57% | 7430a73d125b95bdeda550afc563e968 |
| Genus | Intestinimonas | 76.76% | ad3be8d1d1516eb6c4f088699cef1236 |
| Genus | Intestinimonas | 86.51% | bb23fcbc7b0ae27466463ddc8e415fef |
| Genus | Intestinimonas | 71.60% | 9093cd87e1d14fd988fd79efc5f28de1 |
| Genus | Intestinimonas | 82.15% | 8b1ed532905350bd990052a645480a68 |
| Genus | Intestinimonas | 97.90% | 167256b0a2e0d4e8b79023a5b0ef1e36 |
| Genus | Intestinimonas | 88.47% | 2ee7dfaacf322624075869fbd8802d63 |
| Genus | Intestinimonas | 88.54% | e41f61d26f8254132bec76931076d099 |
| Genus | Intestinimonas | 79.55% | 5bf036560e71bf81ba198347b6f8bdb8 |
| Genus | Intestinimonas | 76.76% | 29af929dfab1d60c110c35a423937d73 |
| Genus | Intestinimonas | 79.52% | 88cd9e13fd321aa9987e2c48f8a83862 |
| Genus | Intestinimonas | 83.47% | 514dd02d5b116caea3f6d41046ce637d |
| Genus | Intestinimonas | 81.56% | 689badbfca64cd1b8ecf39c698e93d8b |
| Genus | Lachnoclostridium | 72.40% | e9c50967af576781f28aacef6fd61735 |
| Genus | Lachnoclostridium | 70.93% | 947425927396c5586ae488b2e86a2dbe |
| Genus | Lachnoclostridium | 90.86% | 59f6a5a934d1427c907bbc47ab343b22 |
| Genus | Lachnoclostridium | 73.79% | 8806e32545fcee27dd7aacbae15aeeca |
| Genus | Lachnoclostridium | 80.54% | 0d6d937dae20bf79ca26819898843a22 |
| Genus | Lachnoclostridium | 77.59% | a0384cb9ec0aaf3ad55b8e9d466998c7 |
| Genus | Lachnoclostridium | 86.47% | b64c076b74d20f536b92e78550bc1fbf |
| Genus | Lachnoclostridium | 85.61% | 3f1447763220aaa5315f1b617ea9a3e8 |
| Genus | Lachnoclostridium | 82.35% | b85890d4c886fc3321b3cfa1c8949034 |
| Genus | Lachnoclostridium | 81.42% | 447c5971613bb227cf9afd63f3212de2 |
| Genus | Lachnoclostridium | 82.51% | 57ccb790ca8af4011409118d7e586017 |
| Genus | Lachnoclostridium | 75.36% | 5b14b5fa31e63638d74dee980315a9dd |
| Genus | Lachnoclostridium | 82.18% | 453e78de0e70f43ed6abd8ec1e758641 |
| Genus | Lachnoclostridium | 71.89% | b5d66885fe52862bd9651e92db13f5f8 |
| Genus | Lachnoclostridium | 99.95% | c7c665cd05f9e180ceff714ddeef1a27 |
| Genus | Lachnoclostridium | 98.56% | 3d6dc2ec88eba43f09ca695882fad940 |
| Genus | Lachnoclostridium | 99.95% | f3a466138d947c7807f8f53d38e7d890 |
| Genus | Lachnoclostridium | 99.12% | a51e88f58352fc6ee3550e7a45d8cc14 |
| Genus | Lachnoclostridium | 98.59% | 156a2a0757a74b2b799b2fb75b7b63c7 |
| Genus | Lachnoclostridium | 99.19% | 7dfb3b0d2d03bca2ad4837120ae91367 |
| Genus | Lachnoclostridium | 80.14% | 5659dab5cdda711bc42e482980670300 |
| Genus | Lachnoclostridium | 98.84% | 818e0ab9ac66a82fe76a46e146feabc1 |
| Genus | Lachnoclostridium | 95.46% | ff2aa4e822ffe5668ba7f6d419b21a61 |
| Genus | Lachnoclostridium | 95.96% | 5d71fa0692ac385e9447e23f2d3ea20c |
| Genus | Lachnoclostridium | 77.46% | 5c8bedb29713735dbb44f21248bfc2a4 |
| Genus | Lachnoclostridium | 97.84% | ccb5a5aea3b5547528e9f19bef74ceeb |
| Genus | Lachnoclostridium | 96.00% | 158313e1bf75e19b9b0b31a4d0feb9e2 |
| Genus | Lachnoclostridium | 99.60% | da5da6862406125847295df87ea9e468 |
| Genus | Lachnoclostridium | 97.28% | fab6e8d565414de4d0cee346ef3708f4 |
| Genus | Lachnoclostridium | 99.96% | 72f84099af86cfb612666c4f0a901158 |
| Genus | Lachnoclostridium | 99.61% | 5276cf6ff2d46c2fdfee7b7114b814c7 |
| Genus | Lachnoclostridium | 99.22% | d2d6d4b4993439ae47114c57cc964e89 |
| Genus | Lachnoclostridium | 70.85% | 6bf44c6ff00681a9c205a27323c91500 |
| Genus | Lachnoclostridium | 75.28% | 945731bc6a1997422aa6135371fa5b77 |
| Genus | Lachnospiraceae NK4A136 group | 76.62% | 324ed7d808330f6b19bb96d704947646 |
| Genus | Lachnospiraceae NK4A136 group | 75.82% | 1b938afa5df8365de020e4f3d27a1837 |
| Genus | Lachnospiraceae NK4A136 group | 76.77% | 33a90cc4c0666a0e64ce7c4bf9b47294 |
| Genus | Lachnospiraceae NK4A136 group | 75.34% | a0989809b9ad90f74e595faa4b3db993 |
| Genus | Lachnospiraceae NK4A136 group | 75.96% | 070d72329ba66638ee5b3de188fcbb3f |
| Genus | Lachnospiraceae NK4A136 group | 71.87% | 586dafff596f549dd0823ad707ccde52 |
| Genus | Lachnospiraceae NK4A136 group | 71.61% | 758b2250f53fe0f51379cc174e9c648e |
| Genus | Lachnospiraceae NK4A136 group | 74.79% | b7201e0e36ea9d63acd12d31a4881aec |
| Genus | Lachnospiraceae NK4A136 group | 78.61% | 7fff0e33e0e3ece334d6dc17d73f557f |
| Genus | Lachnospiraceae NK4A136 group | 80.18% | eb64bd62e40552c35432befbf612e9b5 |
| Genus | Lachnospiraceae UCG-010 | 79.85% | cb09803ed5883121845f2271a30016ef |
| Genus | Lachnospiraceae UCG-010 | 96.69% | 982f376c8ccf45db3671d3ccedcd4b43 |
| Genus | Lachnospiraceae UCG-010 | 85.30% | d8128eb2b7148ca699c9a94700fd7404 |
| Genus | Lachnospiraceae UCG-010 | 89.83% | f4c31add499fa38108d38d78b0f39017 |
| Genus | Lachnospiraceae UCG-010 | 79.76% | 75a7f2543a52d75c90084265052ca22e |
| Genus | Lachnospiraceae UCG-010 | 85.45% | 15fc6202bb60b87d8860e1178361c1b5 |
| Genus | Lachnospiraceae UCG-010 | 86.12% | e4f624deb5662bdb72436f5d9cf7fd91 |
| Genus | Lachnospiraceae UCG-010 | 70.85% | 35823581e7e3f1776175bdbd4f3f0cb1 |
| Genus | Lachnospiraceae UCG-010 | 88.69% | 8dd0635a6511dc62e3e7cf6eaf9b5762 |
| Genus | Lachnospiraceae UCG-010 | 70.64% | 92e01351b5160522a4c645de38908c37 |
| Genus | Lactobacillus | 80.18% | bee32bc2ea717520c1490bcb5abcdab9 |
| Genus | Lactococcus | 85.45% | 3ba5dd87c7b8ffb920492b81bb5a3603 |
| Genus | Lactococcus | 99.94% | c8b934f354c7405e583d194309d6f0ea |
| Genus | Lactococcus | 99.23% | 1158b1f462515340eeec68e39f8115b4 |
| Genus | Lactococcus | 99.90% | daf4d0332d91482ca233632a8f3141df |
| Genus | Lactococcus | 96.41% | 93e0658b13eae287bf30e0512a8540bf |
| Genus | Lactococcus | 71.28% | 7fcd0bf61adaa45ce368aa9ea8f652dd |
| Genus | Lactococcus | 70.29% | b5ff540bf8df43a699cc6687d8457805 |
| Genus | Lactococcus | 71.28% | f93ec952102fe019c635e139a7d8cbfc |
| Genus | Lactococcus | 71.98% | 7208b8c81a3ca8b71295286b7b29afe9 |
| Genus | Lactococcus | 71.28% | e88ac761afab85981e883f27cbd40f4a |
| Genus | Lactococcus | 70.03% | cecc8344eb4431147e98654996520bba |
| Genus | Lactococcus | 76.82% | d471fd836d683fe3f15482e47a26d227 |
| Genus | Lactococcus | 78.93% | 7e7e9a18e813aef2517acef6fe00e685 |
| Genus | Lactococcus | 78.99% | 9a0f8082f9d8d043b33bbeb72948bcc8 |
| Genus | Lactococcus | 72.65% | 60021e11bac9b8e898467ee3216b85df |
| Genus | Legionella | 99.65% | 44646deda6194036f2864e620d1dbf15 |
| Genus | Leifsonia | 77.43% | 167746c490389a06f02cb2219e29f188 |
| Genus | Leifsonia | 85.55% | 5c2779a203ab0ffb9a5a87fd8e3fd7c1 |
| Genus | Leifsonia | 76.76% | 18138f98122247583038ce20bca21bdb |
| Genus | Leuconostoc | 99.95% | 0c6b51d808b79298a8bb03a236639495 |
| Genus | Lonsdalea | 72.77% | 7810315c0dd80460e154b65ab3c3d858 |
| Genus | Massilia | 99.93% | 0e469208a3622a5443b0a3b657803804 |
| Genus | Massilia | 99.93% | 09c71146672281a5e664ad66583a9c52 |
| Genus | Massilia | 99.93% | fa06ae22246fe219c85e270c60c71c07 |
| Genus | Massilia | 99.94% | 7e1b8c2eb24ee6f3c8893122a4dcb3f7 |
| Genus | Massilia | 99.93% | a4a3ed83618f124550c1f0b569ebec6d |
| Genus | Methylobacterium-Methylorubrum | 99.99% | 14a7bf8c7f97b442f5e1d17c13008cd5 |
| Genus | Methylobacterium-Methylorubrum | 99.99% | 9591e5d7df6bf4bcfd339190d4b96b9f |
| Genus | Methylobacterium-Methylorubrum | 99.99% | c72098f6cd7546b44f8ae9dba64f27f2 |
| Genus | Methylobacterium-Methylorubrum | 99.98% | 137f90af9f988d4c445bbb486c233455 |
| Genus | Monoglobus | 71.46% | e5031a89a1a22850d4505f407dbf0ca1 |
| Genus | Monoglobus | 72.63% | 8dca526b6e9308a4de9acea590ad0628 |
| Genus | Monoglobus | 99.30% | 3be8dfdfbd017a057ced0030f5861d4e |
| Genus | Monoglobus | 99.36% | abc0852a5b470d4639f3ea4ccbab3d00 |
| Genus | Monoglobus | 84.67% | 5b065aaf648e882efd7ead3eb20022e2 |
| Genus | Monoglobus | 82.54% | 4e7222584a86b28fafcd3c17b36229a6 |
| Genus | Monoglobus | 94.06% | 1c6b4dbca4514b82af1892710eb561b5 |
| Genus | Monoglobus | 99.21% | d673a4a7a82f707e5ef444f33829f2a6 |
| Genus | Monoglobus | 98.95% | be2685beccb62612732446eb1322b080 |
| Genus | Monoglobus | 92.21% | e53d7203e723ac3468a676bbaf2a0b57 |
| Genus | Monoglobus | 91.84% | d9c8ef60417823ddc6343a96c5b10abf |
| Genus | Monoglobus | 99.20% | 229e9aab0ee0d9a53d65281e07b1eed6 |
| Genus | Monoglobus | 72.98% | 9b2322eddef58fb96e85f1b8af7510ce |
| Genus | Mucispirillum | 96.64% | 35153571791383c3c0c3658eb1804bd6 |
| Genus | Muribaculaceae | 99.38% | 283980eacc9ae51a522224c0b1d76031 |
| Genus | Nakamurella | 70.75% | b85ecc56453da9b8d122e6699ef6a0ce |
| Genus | Negativibacillus | 71.31% | a575946e2bf53b35e00b13b3732fd245 |
| Genus | Neisseria | 98.68% | 679a30407d5813a4740b1f65bc0c6391 |
| Genus | Nocardioides | 80.61% | 003fc277cc93e6bd05759531b3ba6ad8 |
| Genus | Odoribacter | 100.00% | 76636dfa7a43fa53ddcbabf0f79340a8 |
| Genus | Odoribacter | 100.00% | 287de2bcfa4e445fdf1152f41f19bb92 |
| Genus | Odoribacter | 99.78% | 8f124a948398de71a7b274cfcec880c1 |
| Genus | Odoribacter | 100.00% | 3cff474eeae35c9a35ee48ffc70340d1 |
| Genus | Odoribacter | 100.00% | 3b14478967a94d45a68bdb2ef557835e |
| Genus | Odoribacter | 100.00% | 820b6a35f7dc00bad5d99a19338723fd |
| Genus | Odoribacter | 100.00% | e33d9506dfadbc136462910b61cf9432 |
| Genus | Odoribacter | 85.89% | 8db4f8701d64c8bdf20660b05912f2ba |
| Genus | Odoribacter | 81.07% | 4b772f2b0954519c635e6530a08d83a5 |
| Genus | Orbus | 98.91% | 20351471529a4ae2f933230ac7b10346 |
| Genus | Oscillibacter | 99.19% | c3ddf2039d0c1b6e5f73c0f93b81710b |
| Genus | Oscillibacter | 99.23% | 46317f0c08a6ae074239f55d9c467463 |
| Genus | Oscillibacter | 99.23% | 10f440412ff33f737218278f5a9fa26b |
| Genus | Oscillibacter | 99.19% | 6ca1267c2de40c96d1655f847068885a |
| Genus | Oscillibacter | 82.14% | 3ab363bcd1a034a5b8693d84b943565c |
| Genus | Oscillibacter | 99.15% | a0ff5d60dc5e768a0c086e55a5f22484 |
| Genus | Oscillibacter | 99.26% | 0749d9c7b29cfd2ef41052d36335839d |
| Genus | Oscillibacter | 99.25% | a260549478d66b408d8b974fff28da2c |
| Genus | Oscillibacter | 99.89% | 67a812096b8f34b2053e1db8b4f968c6 |
| Genus | Oscillospirales | 98.71% | 86348871465132d1c444fb9f1ffd454b |
| Genus | Oscillospirales | 94.57% | 87f00cd95386c7ad3506187a31c6e779 |
| Genus | Oscillospirales | 98.97% | a72d569aac1672aea78ace6c6f187da0 |
| Genus | Oscillospirales | 98.73% | a1b85a1990b53f9444c63afc99fb955d |
| Genus | Oxalobacter | 90.04% | e6e836328428088dbdf24a33d72ba753 |
| Genus | Oxalobacter | 83.46% | cdefb90ca577353b1c3d912a5438b4cc |
| Genus | Oxalobacter | 81.13% | f707d93045a8783239b20142967c4ac7 |
| Genus | Oxalobacter | 84.57% | df047184ef38e7411370db6806193775 |
| Genus | Paenibacillus | 99.07% | 3796263ffe5aa5bdc6bb17ec3ecfb27e |
| Genus | Paenibacillus | 99.85% | 3065620d117d8df5abac2557b394d826 |
| Genus | Paenibacillus | 97.11% | 1893ab21d32b7f21efee6209525053dd |
| Genus | Paenibacillus | 99.06% | 7afc3451fefed616d257e92b74f4e13f |
| Genus | Paenibacillus | 99.54% | 9b00dda314520c693367c46f79bc6b39 |
| Genus | Paenibacillus | 99.82% | 6fdc417981a2824119d10d2dcc6fc9e3 |
| Genus | Paenibacillus | 99.81% | c60496df94b431e952d2bea2948edb30 |
| Genus | Paenibacillus | 99.70% | 22537d8dff1dc94ceeebcbb6369e2b41 |
| Genus | Paenibacillus | 92.71% | 4c302d4a25831536a80a4ee4f1999933 |
| Genus | Paenibacillus | 92.72% | 9a594495c41f65e12b833b07b89bcdec |
| Genus | Paenibacillus | 99.85% | b5120a9f17ac78c4ff5cf8f85e19784d |
| Genus | Paenibacillus | 99.81% | b6be611d5553f4dd089607c3f074dc19 |
| Genus | Paenibacillus | 99.78% | cd36d529e76f11807bf897610467bac7 |
| Genus | Paenibacillus | 99.60% | 8adda1248b9c4e5fe85161d9b8c9f7f8 |
| Genus | Paenibacillus | 99.57% | 4aefd91abdae86f29fe1da3e100847e0 |
| Genus | Paenibacillus | 98.56% | e1ea1ef49204fc50c3db3cf0dd027e43 |
| Genus | Paenibacillus | 99.77% | cac7f69b44d4a41bc35bc74049044363 |
| Genus | Paludicola | 85.12% | 279e3e427bb56d83342aae531e69f795 |
| Genus | Paludicola | 78.78% | 8082af77a5288bdb297b9ffceec99801 |
| Genus | Paludicola | 90.63% | 4fa90ba73f31d6abaffead01f8eb6ebe |
| Genus | Paludicola | 94.53% | a36edccf5954c9e715f68e4c1cd09ebb |
| Genus | Paludicola | 97.75% | 7afa9a5edabf3db6ce5cf29f012b4a95 |
| Genus | Paludicola | 97.10% | 8721bfeb7a9f78a5081d54bd123a6fdb |
| Genus | Paludicola | 90.60% | d96efcaff24427d0342d554cb2b204b2 |
| Genus | Paludicola | 71.09% | 6f764a44f54c3e2b72ee93dc7152a4f2 |
| Genus | Paludicola | 94.59% | bb9a0cfa98e1bcdcb99940cbaaa32fbe |
| Genus | Paludicola | 89.25% | 9f8d5345d16b34d6e3ca62784276f22f |
| Genus | Paludicola | 72.34% | d53da898361ed1caa6ad64c4db37f715 |
| Genus | Paludicola | 93.72% | 6ebecefa156a0454d20303b0ed2bda49 |
| Genus | Paludicola | 73.33% | 89a03c42c168c70ea3663295cd841d06 |
| Genus | Paludicola | 96.89% | 034c2915fa930f7382f6024836479bdd |
| Genus | Paludicola | 92.33% | c761095b11b2d52d816caa874b08fb81 |
| Genus | Paludicola | 84.40% | 54d81ab5231d9aa1e173c081a63d4dec |
| Genus | Paludicola | 91.34% | fedd7921eaf735c4141dcb99ce1e37ce |
| Genus | Parabacteroides | 99.97% | 54b24c7c3abf66b5e9deac873aebfea4 |
| Genus | Parabacteroides | 99.95% | b04ee14988547959a9debfdeb201050c |
| Genus | Parabacteroides | 95.45% | 8ae3df64e4136837489689f80045379b |
| Genus | Parabacteroides | 95.47% | db2374faa3d739369bd38dcc52bd45d2 |
| Genus | Parabacteroides | 99.97% | 6abb6a1205168e95530bb2540296ff0d |
| Genus | Parabacteroides | 95.45% | a265fc80678e1386ac463ee340f3e539 |
| Genus | Parabacteroides | 99.97% | 1d0b43c054c8014aca3ab9b9163471a3 |
| Genus | Parabacteroides | 100.00% | 0d469dd29d5a0df6e4649d6dcc34f70d |
| Genus | Parabacteroides | 95.16% | a80a4e440c518a7f583b822f1b84cbdc |
| Genus | Parabacteroides | 99.41% | 841e9cb88f43de64579572ddf7f31124 |
| Genus | Parabacteroides | 95.41% | 4145bfbeb890e8c905aff3d996b144bf |
| Genus | Parabacteroides | 99.97% | e3ae3283cfc5778a1b9dc26181fde625 |
| Genus | Parabacteroides | 100.00% | 423f3e3a7c0749419bcbe12b44847bda |
| Genus | Parabacteroides | 99.97% | a207e131e7bab1ef424dbb05171fa6fe |
| Genus | Parabacteroides | 99.96% | af8d7e2a240153205981b5a20b5c1e92 |
| Genus | Parabacteroides | 100.00% | 7b01af5fddffa43278a2c8a0e5029722 |
| Genus | Parabacteroides | 100.00% | 5a705844478988637f63f32bd51c388e |
| Genus | Parabacteroides | 100.00% | c8f539a484b16f3132f0430115180876 |
| Genus | Parabacteroides | 100.00% | 2592869b69cc8d589e57f72e560cc1df |
| Genus | Parabacteroides | 100.00% | 99ffdbf9bc57213b4bcf6ff4b48303a8 |
| Genus | Parabacteroides | 99.99% | 9afbf42703372caecad14dd39d3b6edc |
| Genus | Parabacteroides | 99.96% | 07fc08d936a2ac9de5cc04702bff2186 |
| Genus | Parabacteroides | 100.00% | c319afdbc84dc3bca70499a838db222e |
| Genus | Parabacteroides | 100.00% | ebb527d1a6142073df8a4249a4011cac |
| Genus | Parabacteroides | 100.00% | 2e726268d1ec2c12d90fdbf9c963d5c3 |
| Genus | Parabacteroides | 94.86% | 91973edca2e416261bb132a93a340e89 |
| Genus | Parabacteroides | 100.00% | 2c79a518ecfdfa1b35a8b018e5d23b40 |
| Genus | Parabacteroides | 99.97% | f6381c34aa7067cfe68264859faf4f82 |
| Genus | Parabacteroides | 100.00% | dacaae160cef83726a20e5397764e70a |
| Genus | Parabacteroides | 100.00% | 7472ecd6b3d9206e9059fa4dbb39636e |
| Genus | Parabacteroides | 98.70% | 1933823deff2ef24b14d512e1e22f222 |
| Genus | Parabacteroides | 100.00% | e3f8a06cce1f7c519fbe466093b29be7 |
| Genus | Parabacteroides | 100.00% | 340ebe7212fad9bfd0b325ec369e6eaa |
| Genus | Parabacteroides | 100.00% | 3e1a3152a7b758d9b9db7a2057d5e416 |
| Genus | Parabacteroides | 100.00% | d2aab5d00eec9781bfdbf22c5b9e279d |
| Genus | Parabacteroides | 83.90% | 25e277bc6a2a736917a2d6f360daf431 |
| Genus | Parabacteroides | 100.00% | 4db22afe7db2fbab9769aa05b28387d9 |
| Genus | Parabacteroides | 100.00% | 7bddf613ce93ead373d59613f24a3168 |
| Genus | Parabacteroides | 98.70% | d14f8f07579b827a2436d498964da06e |
| Genus | Parabacteroides | 98.31% | 7552b98d8c7d49cd5a03e3864f70df24 |
| Genus | Parabacteroides | 98.41% | 536478e018cd64338a4de3ca408a8674 |
| Genus | Parabacteroides | 100.00% | e3d9b5853db5bfc1e5ada0dbffff6535 |
| Genus | Parabacteroides | 99.96% | 888dbc703ecb7985577ffc72b367b763 |
| Genus | Parabacteroides | 88.10% | affebaf491f3fdba832a49b2e833fe60 |
| Genus | Parabacteroides | 99.99% | c5049c40c70b9243967417070677cccd |
| Genus | Parabacteroides | 100.00% | 4655042734c42547303ce3ceb2e74907 |
| Genus | Parabacteroides | 98.58% | 1696cd60b75ee3d412a8f620b6dcfbd2 |
| Genus | Parabacteroides | 100.00% | dcd9add32c89779f8e1c0e918955cf6e |
| Genus | Parabacteroides | 100.00% | b4bd9134a4c53e917b4a757197086ee7 |
| Genus | Parabacteroides | 70.75% | dbe11b588ab70a1be050b60d3ae9e6da |
| Genus | Parabacteroides | 99.69% | d47e9db4a21f15bbae7cd5d95d802c5b |
| Genus | Parabacteroides | 100.00% | 7e366034e1e8f928abe5e3f60fd0b62d |
| Genus | Parabacteroides | 99.99% | 72393f2e15fbe623f3e6d7f407650c8e |
| Genus | Parabacteroides | 100.00% | 1472f4fb55e4a98824ba1a532ae0a73e |
| Genus | Parabacteroides | 100.00% | 31350024146bcb19673f85ddb10bfc40 |
| Genus | Parabacteroides | 100.00% | 6a067aeec69a802b2d8740a39b5cbf9a |
| Genus | Parabacteroides | 100.00% | 5de6e949c0d911b6b768c461bc7e837b |
| Genus | Parabacteroides | 100.00% | 661ea170c0cb3962fe3989556d7cc2e6 |
| Genus | Parabacteroides | 99.66% | 005de3c61400371a5fe173f310fa0972 |
| Genus | Parabacteroides | 100.00% | 79d4a843a19010414d159d24a0ccd808 |
| Genus | Parabacteroides | 98.74% | abd352584dfdef469a805ac145c5c8ba |
| Genus | Parabacteroides | 100.00% | e8706bbe205f5c83c9b854f4434a7eac |
| Genus | Parabacteroides | 83.22% | 1b91e21c903835f328061c75ff536961 |
| Genus | Parabacteroides | 100.00% | e401965f780c6d67c366444fedd7cfaf |
| Genus | Parabacteroides | 81.64% | 578f6618889e44c92b2bb2015357be59 |
| Genus | Parabacteroides | 100.00% | 8814eb1fd1a6134d1c0ef99532bb554c |
| Genus | Parabacteroides | 100.00% | da46565280f2d5fbf293e941ad46a53c |
| Genus | Parabacteroides | 100.00% | 7c7cff025779a43c0b085674bf748df2 |
| Genus | Parabacteroides | 100.00% | a130e942479e51f7015bd2cf20b7e3a3 |
| Genus | Parabacteroides | 100.00% | cf492de99a90a330e5298bf96abec0ef |
| Genus | Parabacteroides | 99.97% | 2814c57a0afb3b5cf8dbf586735e67d4 |
| Genus | Parabacteroides | 100.00% | 3c9309a235e9769dc1ea1fff5385ce19 |
| Genus | Parabacteroides | 100.00% | 7d149f6dac2b44a4cfd699b531389142 |
| Genus | Parabacteroides | 100.00% | 697840e58f06e58ed9d0aee708bed21e |
| Genus | Parabacteroides | 99.52% | 022d1823d2d2a3e47fe8ffa2c738784f |
| Genus | Parabacteroides | 99.96% | 5cae9f5de9eda832d306a3981533296a |
| Genus | Parabacteroides | 97.95% | f760482120941f4c90c03030446193f1 |
| Genus | Parabacteroides | 99.95% | aba9b8a9e23a99225496f6f5d7304475 |
| Genus | Parabacteroides | 100.00% | 0ca8a4d09b198d14a9bc664cc615b6e0 |
| Genus | Parabacteroides | 100.00% | ada4ca386994deff3fc851fbb1e8c5a4 |
| Genus | Parabacteroides | 99.99% | e88c8b5486289848a39687e5761ced61 |
| Genus | Parabacteroides | 99.74% | 6e34e2809991d54ecce10df4b75aad36 |
| Genus | Parabacteroides | 94.50% | 882d1c5fce5a4be09d16dc6433ceaf0a |
| Genus | Parabacteroides | 100.00% | 09110ae7a6b8d525c1d9836c5100c942 |
| Genus | Parabacteroides | 89.16% | c15c19f6065568e5d937bff36c79c0af |
| Genus | Parabacteroides | 100.00% | d20071535acad95962370ccbcd7288be |
| Genus | Parabacteroides | 95.90% | 8ef10cdecfd3d3e1fe84d8dd974d6724 |
| Genus | Parabacteroides | 100.00% | 6c7dd7e212cd0c5fd2b2c85386d160e7 |
| Genus | Parabacteroides | 83.26% | 3e53fe11ee6ff4cdd08cef779ccd3b51 |
| Genus | Parabacteroides | 99.69% | d1daf6113b6714404a25ddbe94e52d4e |
| Genus | Parabacteroides | 99.49% | 3045a617567eb63b553fa63be4a7cd2c |
| Genus | Parabacteroides | 100.00% | 9c39f12cf81a34b0f788fada2e359b67 |
| Genus | Parabacteroides | 99.97% | f165baa223edef85fd9bcf00b0c1ea44 |
| Genus | Parabacteroides | 99.96% | 01aed209d1cbd34738f3212339dbef69 |
| Genus | Parabacteroides | 99.58% | 6a5e3db2be611927ad8b945a0da988ee |
| Genus | Parabacteroides | 97.31% | 61b584bd2e125639eec30080c1526fdd |
| Genus | Parabacteroides | 99.96% | 61d66d59dee95f26923f0d41a822c7dd |
| Genus | Parabacteroides | 99.95% | 9e14276d44de6fb60098b3d966c29f29 |
| Genus | Parabacteroides | 99.96% | 91d2ca8364c8e5e45f5dc8dd6b8b5408 |
| Genus | Parabacteroides | 100.00% | 94d1931759c0cfbef949675e3f401e13 |
| Genus | Parabacteroides | 99.96% | 772dd2d032cd4e9c4bd1ba819cb4c5d6 |
| Genus | Parabacteroides | 100.00% | 1b73e023716111fee92379ec60e4b7e1 |
| Genus | Parabacteroides | 99.96% | 579ec2a82382470ee04c969405dd2327 |
| Genus | Parabacteroides | 99.99% | cc1810dc5166291e4a4f11cdf727f7b2 |
| Genus | Parabacteroides | 76.18% | 05ff07fe879155216842e583227d2c79 |
| Genus | Parabacteroides | 100.00% | 07e30523017952f4121bf4ec73b29174 |
| Genus | Parabacteroides | 81.00% | 6fb5c01a4ab5efc2756da4941d381c88 |
| Genus | Parabacteroides | 99.97% | e11d079d7f6c536af3916fb823783db4 |
| Genus | Parabacteroides | 97.93% | 9b9ea9aaa1a389807b2cbe68f74354c6 |
| Genus | Parabacteroides | 100.00% | 51aa1a07bf9e56488827356417b93a26 |
| Genus | Parabacteroides | 100.00% | 7e7594d3ca354c713ff2ef0fab5a9d4f |
| Genus | Parabacteroides | 99.96% | 1c21b6b8e6c3c9ec02632eb5ea9a62b9 |
| Genus | Parabacteroides | 75.36% | 4f2bf979e2747cd8032d2505902b823c |
| Genus | Parabacteroides | 100.00% | 170f1480ff0ed06b3c15299ba8657b87 |
| Genus | Parabacteroides | 100.00% | 37c7c37a05515efce2a601e63e2e658f |
| Genus | Parabacteroides | 99.99% | 636dcd6dc155304457fad9a0558cb1ac |
| Genus | Parabacteroides | 71.03% | ceaaf66708b8224a0b3efabd782963ed |
| Genus | Parabacteroides | 99.99% | 147e4932edfc49ecf3d334d6d5bc6846 |
| Genus | Parabacteroides | 99.65% | 4cb237a15d5ecac89715450df114f0f7 |
| Genus | Parabacteroides | 80.22% | 051d9d88278b9a29e249129521a32ded |
| Genus | Parabacteroides | 99.26% | 3b168cd28ef1311088bd7c28ce4afa93 |
| Genus | Parabacteroides | 92.33% | 2fd9464d5c436f042a9db087d0551678 |
| Genus | Parabacteroides | 98.28% | be5666665be0800e74cf06d8a241e299 |
| Genus | Parabacteroides | 74.49% | baaf67578a7eb6e6e72223a0a8fd90d7 |
| Genus | Parabacteroides | 100.00% | daf958cc3193f0b241cd8bf73f96bb93 |
| Genus | Parabacteroides | 88.59% | 4da183ce6ae51780ed5657be1acf3c92 |
| Genus | Parabacteroides | 96.08% | a2663400893f33b01090f897a3c086da |
| Genus | Parabacteroides | 99.99% | 8f14ea1110007fb3c73d79ec2a7e659f |
| Genus | Parabacteroides | 88.26% | 356e463939c7c0be0cadab33c287342e |
| Genus | Parabacteroides | 76.17% | 449daea38168e823fd8439d1a6c89a2c |
| Genus | Parabacteroides | 95.95% | d9bf2af7eb1fec0b286cb0773d80c89e |
| Genus | Parabacteroides | 100.00% | ecb6d23bad064e0d03e5b3b7fb762e9e |
| Genus | Parabacteroides | 78.91% | c040e5718d9076b51c4d59e4caa7d43b |
| Genus | Parabacteroides | 99.88% | 944f780a1c3ab58ae35f3ff0f7d9e4bd |
| Genus | Parabacteroides | 74.99% | 7b7f98ce11f34be33ff3412b0e56759f |
| Genus | Parabacteroides | 96.68% | d8a4634e3316007fae013bb332f4e52e |
| Genus | Parabacteroides | 98.34% | 4ccbf6e4610316f75d0bd080f8425793 |
| Genus | Parabacteroides | 98.15% | 87f4dfb61e1a4681d640ee8c3d7cbc36 |
| Genus | Parabacteroides | 87.40% | 9a6e860048d30198788101388d791265 |
| Genus | Parabacteroides | 83.90% | 96512a37cff6967f6798e2aa8a707043 |
| Genus | Parabacteroides | 100.00% | b3771043d58a0840e73fe0962c402350 |
| Genus | Parabacteroides | 99.97% | 41cd85e5ffd1db5b57b43d8b282695c7 |
| Genus | Parabacteroides | 99.87% | ad1112e4211483d56bab35d826498c9e |
| Genus | Parabacteroides | 73.04% | 62220ba4b2a34ab50aa42fb7a2fdac04 |
| Genus | Parabacteroides | 81.84% | a406af3b4eab701d2006efa45774c6b4 |
| Genus | Parabacteroides | 81.19% | 4e3665136d8ad5a86bd8e9abae15b99f |
| Genus | Parabacteroides | 99.94% | ded4af936ebedbd9f67ad18097f8eab2 |
| Genus | Peptococcus | 99.46% | 93160866ff928570c434ccdf00a60256 |
| Genus | Peptococcus | 99.48% | 057cc40c76073a90093da5d9ea9c0b54 |
| Genus | Peptococcus | 99.33% | 9ad7a09e30019bbc040f080b2610e29e |
| Genus | Peptococcus | 99.30% | a1087a00380f6515aa798ea1350b6736 |
| Genus | Peptococcus | 99.26% | 49cea3ac3913f47abc357dfb7161310a |
| Genus | Peptococcus | 99.24% | f53f32adf9533f45bccd110ac1169549 |
| Genus | Phascolarctobacterium | 99.92% | 3909b7ec2f528094c77c339e19d6b510 |
| Genus | Phascolarctobacterium | 70.23% | de0aa4b422eb99ae5532aa3877c81158 |
| Genus | Phascolarctobacterium | 70.31% | 2ba774088668311d4234948945a3438c |
| Genus | Phocea | 91.16% | aa44cd59a9b60b5ab394727804b956b2 |
| Genus | Phocea | 82.12% | 0fb2e00fa9b1976ea42e2f7bfb055c8b |
| Genus | Phocea | 79.08% | 919f44eb235afb0aacb0261d06978fe4 |
| Genus | Phocea | 79.09% | accd98aa18b08eb3162afff5c7cf28cb |
| Genus | Raoultibacter | 99.99% | daa2d58e3dcadc9eb45585f2970e7d53 |
| Genus | Raoultibacter | 99.99% | 39b6d16ec89d4670675eaeae4b99dcfc |
| Genus | Raoultibacter | 99.97% | 50b4a1ab19d9d50e47beccb124c4555b |
| Genus | Raoultibacter | 99.98% | 1b90e870dc1d4f362229789eefd2c086 |
| Genus | Raoultibacter | 99.99% | abec05a7d0212657654d7c6116b046bf |
| Genus | Raoultibacter | 99.75% | 0e1daa3d733805cfcd421cf814aee044 |
| Genus | Raoultibacter | 100.00% | b4f026c0cc97705ac5cadf01a3174867 |
| Genus | Rickettsia | 94.83% | 793a24c17d44d36dae0dab0102656853 |
| Genus | Rikenellaceae RC9 gut group | 86.45% | 0233c9c09061f047f6f22525577aebe4 |
| Genus | Rikenellaceae RC9 gut group | 71.60% | 61c5e8b3e2bb477c103bc609eb8dc63a |
| Genus | Rikenellaceae RC9 gut group | 77.14% | 3a3f53ec2eb9c454edd4ad91f8024430 |
| Genus | Rikenellaceae RC9 gut group | 71.28% | 09970674e4824032a8978e7c2b61e910 |
| Genus | Robinsoniella | 78.25% | 7e1972dd07e32b5e0dcd7b3eb986a895 |
| Genus | Robinsoniella | 82.12% | 98d5eefac71b851008c6f755e1c1abdd |
| Genus | Robinsoniella | 81.91% | 16ebfc21ffe6af1b2195236627f7e60a |
| Genus | Robinsoniella | 79.93% | f82d2a6858132af71ed2eb44b7133336 |
| Genus | Romboutsia | 95.41% | b06f7f3a792c533a73539f806734e6a6 |
| Genus | Ruminococcus | 72.93% | efd38ea258750b2775ffadf8b926ff3c |
| Genus | Ruminococcus | 74.75% | dc37ebedb46059db5dd45bca51da5ac8 |
| Genus | Ruminococcus | 100.00% | 31f5f8c014edcc4b9b6bdbf79b84ad8b |
| Genus | Ruminococcus | 71.99% | ee0d5b0168011b0a8b6c043683b77ee8 |
| Genus | Ruminococcus | 71.10% | f99e8cb26cc8da6e2c24d8c34c801d8f |
| Genus | Ruminococcus | 71.32% | 81dd2d16406f5b53fb0e3ff3f0c413e6 |
| Genus | Ruminococcus | 76.85% | 53af40cc2d06fa03b948be76afa31e24 |
| Genus | Sanguibacteroides | 100.00% | 6b0de8e24d831f16fcdae8d2dcd002d0 |
| Genus | Sanguibacteroides | 100.00% | 62a42c7b89fd6976c4c0c9f5d8fd9bf0 |
| Genus | Sanguibacteroides | 100.00% | 96c913230830f8f11059864420e4140f |
| Genus | Sarcina | 94.65% | dfb984414f8dee2933f835abee59b372 |
| Genus | Sarcina | 93.16% | a10aa46d0653234b65c8ac27afa53549 |
| Genus | Sarcina | 93.13% | 6796b3c7f244db32942ff59684e59bf3 |
| Genus | Sarcina | 93.26% | 50f699522bbd0a9eaed47ff53e4efe5d |
| Genus | Sellimonas | 84.02% | 0c1e24c2df64508e287431b96dd1a1c0 |
| Genus | Sellimonas | 89.81% | f2a3dca4a95da37c2206ecc88a823de4 |
| Genus | Sellimonas | 97.56% | 3da1866985e92ebc5c747a6aaf385b6c |
| Genus | Sellimonas | 93.37% | 314b85e4461166e324d648a035c445d0 |
| Genus | Sellimonas | 74.03% | 11413cd1b6ac74f5bd53c18ec5db9072 |
| Genus | Sellimonas | 97.84% | b4c291c3f44b1b71966fbd3ef34013b2 |
| Genus | Sellimonas | 78.18% | 5146cc750d2663f2d21d3635060746a8 |
| Genus | Sellimonas | 85.70% | 38d6440ac36065b2e4203b0c1d8d0964 |
| Genus | Sellimonas | 89.74% | 7edc7d1d4d3152537b122a0f683c7657 |
| Genus | Sellimonas | 75.01% | a34ab995831ed2bf283ec20eb393242b |
| Genus | Sellimonas | 92.38% | 7621863fef8e9b9d0ddca4497f79c743 |
| Genus | Sellimonas | 98.67% | 1e80c3feab2f6cc71fabe89543d4b9b0 |
| Genus | Sellimonas | 82.63% | 2fc2ebb28ad9a2b793e59705ecf3757e |
| Genus | Sellimonas | 71.60% | d0c2914ab81654a0d99cd501e0feb648 |
| Genus | Sellimonas | 84.25% | 5d88459143cdebe73c7338a62adbbf07 |
| Genus | Sellimonas | 98.29% | cf608c6d092efa34a738b1d927205dd3 |
| Genus | Sellimonas | 85.30% | 19f41b741d6389e9767c226142bb99f0 |
| Genus | Sellimonas | 95.19% | 50a8ce9e9ef4494c8652fe59ff1dd0b4 |
| Genus | Sellimonas | 81.91% | 3bb81fd4d920189a43626ffa23c466ff |
| Genus | Sellimonas | 87.15% | c19ed03b73de2638164f7728ab123412 |
| Genus | Sellimonas | 98.92% | 7e2bf0cfe0763954ae45385ac0bdba0d |
| Genus | Sellimonas | 98.21% | ad51ca9454abddb49b3984dd170bdebd |
| Genus | Sellimonas | 70.05% | cbda6f33cdfdc1d2a7eef67baf40aafb |
| Genus | Sellimonas | 92.62% | 4178675a2dfb3197d2200458d5da3f44 |
| Genus | Sellimonas | 99.71% | b0b63e3350f5de6c9978d23376eb1aed |
| Genus | Sellimonas | 77.22% | d396ccd3fd936e4c80470b6e3a36dd65 |
| Genus | Sellimonas | 83.65% | 293e42f468eb0cf7f8ab0d0b3f7b2023 |
| Genus | Sellimonas | 96.00% | 8970aae6cd1de4b9a3ec6b195087f9d7 |
| Genus | Sellimonas | 97.42% | 2c3e94825125fb573465439d088b1262 |
| Genus | Sellimonas | 88.47% | fdbb24d8828021ff5b54c18d1e92269e |
| Genus | Sellimonas | 95.80% | d50e8ea1dc372e01e5a98e27cc5f6572 |
| Genus | Sellimonas | 99.61% | 36451f6d478a7329bcd0875a90c82727 |
| Genus | Sellimonas | 83.10% | 5fbe61b94fcb1296ec1f2c4efe1529d8 |
| Genus | Sellimonas | 97.47% | 73af614331911b01fc9fc51d95c82efa |
| Genus | Sellimonas | 85.17% | bd01711e0baee0234390bb533ae1ee86 |
| Genus | Sellimonas | 82.31% | 5bcae697d72ac4152f17a3aeee4278a7 |
| Genus | Sellimonas | 86.95% | 53e6fdd4aab4db59c4398f56f10cc0d7 |
| Genus | Sellimonas | 83.06% | 9c5e551d4d6236f04489f386f68904a5 |
| Genus | Sellimonas | 71.34% | d31405167ba6c9f31b6b98a65eaad6ef |
| Genus | Sellimonas | 75.64% | 7cf9be07c185bc49453002ea16db8d2c |
| Genus | Sellimonas | 81.47% | 46a092c66e7e3a0771a9810b19f776f4 |
| Genus | Sellimonas | 91.71% | 6bd4aaf5088838890d6a54ee63552e2d |
| Genus | Sellimonas | 98.47% | 89bb363923643ab4917bcce98d588fc6 |
| Genus | Sellimonas | 83.68% | 33c76afcea286a07d23cf836e282dab4 |
| Genus | Sellimonas | 97.53% | e60815e0e6b7c9bd55fe768b5d5eac91 |
| Genus | Sellimonas | 77.59% | e28193073342ec8db6279f28cc23b61e |
| Genus | Sellimonas | 72.38% | 62addcf07ea22f1d6455e1f0c667788b |
| Genus | Sphingomonas | 98.92% | 1a463602cf085b7969884367fd56b26e |
| Genus | Sphingomonas | 99.59% | b5446492508d2e60459080cc3c3adfe8 |
| Genus | Sphingomonas | 99.04% | 0255074715f620f2113c73f317e42957 |
| Genus | Sphingomonas | 99.32% | 64ea6310091f5a1312563e8ce7c2a3f9 |
| Genus | Sphingomonas | 99.51% | 491c6c4b5dad515a08083fdbd332d412 |
| Genus | Spirosoma | 90.22% | bd7f6ea0f34b5269ab33d0079d008f24 |
| Genus | Sporomusa | 100.00% | 087c8d867b1a12250069c8b4f1f75d31 |
| Genus | Sporomusa | 100.00% | 13f47a4517e1ed88c589e9afdb0289ab |
| Genus | Streptomyces | 72.63% | 9c883dcf57fc98c72e6f8305da006ad1 |
| Genus | Subdoligranulum | 78.89% | 71c33462a52269562b142ec609c4692a |
| Genus | Subdoligranulum | 71.10% | 74dced69bc211357ffa53dd440a109d0 |
| Genus | Subdoligranulum | 73.81% | eebefa815f7d62e3db702eb7a1ce2b9f |
| Genus | Subdoligranulum | 72.08% | 8e1488049bde7720bd1c1964027bd0a5 |
| Genus | Subdoligranulum | 70.32% | 8b4706456d5e7542f6848342890093d8 |
| Genus | Subdoligranulum | 88.45% | def435c9a63b6bb5afb6b2c685202498 |
| Genus | Subdoligranulum | 95.79% | 84af3dc88629934855acdf94a2d99176 |
| Genus | Subdoligranulum | 95.83% | 499409937ec447217a8673f3dafdda9c |
| Genus | Subdoligranulum | 95.29% | 0e744667412f478bc4aabbc03923a009 |
| Genus | Subdoligranulum | 77.10% | db8178aaaa6197e1203511f87987e773 |
| Genus | Subdoligranulum | 89.09% | 10204c78f3d233950ed30d42b8f1783d |
| Genus | Subdoligranulum | 83.42% | 5a95f2b964d04a7180c1488bd60cae68 |
| Genus | Subdoligranulum | 77.00% | 7781a26c86f230d973773902300a941d |
| Genus | Subdoligranulum | 87.89% | 820de46ea8dd0d8f90361d97fa425ab1 |
| Genus | Subdoligranulum | 95.56% | 1c827d9c38635c97b1e8dadc7fd9af5f |
| Genus | Subdoligranulum | 84.39% | 4b7135e84962695d015b95e9760dd999 |
| Genus | Subdoligranulum | 88.45% | 70cfc06cd9a003e864b18daa2415d54e |
| Genus | Subdoligranulum | 70.23% | 59e5f47110fdd8b8845939361de39152 |
| Genus | Subdoligranulum | 85.41% | 746b53b0fc5457e650c71778ea116f01 |
| Genus | Subdoligranulum | 95.33% | 71776047858a90d1af847043bf83ad52 |
| Genus | Subdoligranulum | 88.65% | 1ede7bbe9ca15e4c7d74a5efdee006fb |
| Genus | Subdoligranulum | 89.69% | 1ec610652c3702b7742da78c7f8e503d |
| Genus | Succinispira | 99.62% | 4fee1899e594fe9fb9ba8610f0026044 |
| Genus | Succinispira | 80.56% | 6a03371957f20aa8d029d372d988e580 |
| Genus | Succinispira | 81.32% | 5d46b01d3935838d4dafa474f18ff839 |
| Genus | Sutterella | 74.88% | ee33b66d55e246cb5cb3c6d2defaf8e6 |
| Genus | Sutterella | 80.08% | 3ffac7659ab1b8452191509788a12804 |
| Genus | Tepidibacter | 93.44% | 3c5bca461b02d54c6f3233ffef70b3bb |
| Genus | Tepidibacter | 94.99% | dd812ed8688d792d38221e04ec5b9b14 |
| Genus | Tepidibacter | 99.85% | 733d67109946de5c465efbab99736cc4 |
| Genus | Tepidibacter | 99.86% | 880ad42302bb70e98c985fd71a314360 |
| Genus | Tepidibacter | 99.89% | a6ee2b7b11fa2b20b0dff02a865ea3ac |
| Genus | Tepidibacter | 99.65% | 0ebefb3b6aa6b2d917e3919a7542fedc |
| Genus | Tepidibacter | 96.92% | 4469af743a8f11ed415144fdfafcb4d0 |
| Genus | Tepidibacter | 99.51% | c015ab12ef0234aeb1d42088103af3ec |
| Genus | Tepidibacter | 99.60% | 76d34be263d456939aa61f392fb6ff39 |
| Genus | Tepidibacter | 99.76% | d711bb0ac4b5b48da5d83160cfec6b24 |
| Genus | Tepidibacter | 80.62% | c8e82cb5f3ad978ba00a2c93551192e8 |
| Genus | Tepidibacter | 99.56% | a1153e5c967475e9264774b19ffafaa9 |
| Genus | Tepidibacter | 90.36% | 64dd3b9f3807417a8c7412f3c7a6a233 |
| Genus | Tepidibacter | 73.69% | cadbf061e9941a5534b091117eec6e8e |
| Genus | Tepidibacter | 96.39% | 84896bbd214f8cc5e64ccf2d281c02e6 |
| Genus | Tepidibacter | 93.42% | 9676e41c5689f1c8d629cebf8a5a624d |
| Genus | Tepidibacter | 99.77% | 48d049baf32d36a67cf21cf6867d6f12 |
| Genus | Tepidibacter | 99.68% | a1fd63f5b2348aa1267bec56ee86583b |
| Genus | Tepidibacter | 83.29% | ae9532b30cd28fd011bb0227accacb51 |
| Genus | Tepidibacter | 74.78% | 2af80d855e51434aa836ef2eca206892 |
| Genus | Tepidibacter | 87.17% | 936002476ccef3bdb645e2eaddf71831 |
| Genus | Tepidibacter | 96.69% | 4778e1231bf7524f1fc45f9d4eb343fc |
| Genus | Tepidibacter | 95.71% | 7d5dfd9d559602cfe8c2468456a7a9bb |
| Genus | Tepidibacter | 98.16% | b682d9993ea3a55cac4c82c439ddcf37 |
| Genus | Tepidibacter | 80.77% | a36833477bbb6c5822dc452347014a80 |
| Genus | Tepidibacter | 80.98% | b5f99c1cef01a59a07c2973222abed1f |
| Genus | Tepidibacter | 99.27% | aab3044e984a8afd982e2e0ee9e5036d |
| Genus | Tepidibacter | 78.45% | 1033914d3f72eb5d88ac80f566bea2fa |
| Genus | Tepidibacter | 76.78% | eb27f3ff43f2c54233e2372dd6a3033d |
| Genus | Tepidibacter | 98.74% | 5d64426760b0f5f771f58fd45ef0d446 |
| Genus | Tepidibacter | 99.29% | cf8ccf9324db338242bb8be172067cf8 |
| Genus | Tepidibacter | 96.86% | 2e9d4473a5ce9ebf1967c8be03f4184e |
| Genus | Tepidibacter | 99.04% | 1327e9f7487d45786301ecd5b27bf00e |
| Genus | Tepidibacter | 99.17% | 17a540927825c1b7ec06afd73829ecc7 |
| Genus | Tepidibacter | 74.92% | 6b922e005825f86c9dd6424f82bfe536 |
| Genus | Tepidibacter | 99.83% | f3f88ebfa91ae92ef2acf2e715efe18d |
| Genus | Tepidibacter | 99.83% | 5a3da2cdbc82de0871ddb4e56d97242c |
| Genus | Tepidibacter | 70.67% | 0e529f23a1d5a62e4159cbec2f5761dd |
| Genus | Tepidibacter | 74.69% | 5ba6b8f2ffc6b1e494255dfc955a7139 |
| Genus | Tepidibacter | 91.85% | 00f0cd999cbddef8e6b97cd528ffc03d |
| Genus | Tepidibacter | 83.32% | 4c032c795d43e28feef50f6df2936626 |
| Genus | Tepidibacter | 81.40% | 83a46cb6aa7ec5960be0c963a0154a30 |
| Genus | Tepidibacter | 99.61% | f0f52db34c076c3c2c9aebb2fa375c79 |
| Genus | Tepidibacter | 86.35% | 5d94eb4666be42911441f9b48954a9e4 |
| Genus | Tepidibacter | 95.89% | 4aab950ba1981b3285aec5fdde3398ac |
| Genus | Tepidibacter | 93.43% | 6fb0127de5fe1c52c5e2e259f2b080e1 |
| Genus | Tepidibacter | 84.18% | d14a0529c5b1f0ced0d6ecc1f0d7f382 |
| Genus | Tepidibacter | 96.07% | c72144bd5b150aa64f718138f57efb56 |
| Genus | Tepidibacter | 95.63% | dd3ac655b4a8188751e523fa3bbc89d0 |
| Genus | Tepidibacter | 83.79% | 9f5ddd8f8f67f472d7f2a12c285bc2a2 |
| Genus | Tepidibacter | 100.00% | 767efddf3c8cfdd46c2d56a3df46519e |
| Genus | Tepidibacter | 73.01% | 21894002b3c14d77694a38cf2867f56e |
| Genus | Tepidibacter | 90.16% | 52326542b22fbeabd0dbdedddae81fae |
| Genus | Tepidibacter | 96.48% | 98aa857f8d4b71b8430670aeb04d5a5d |
| Genus | Tepidibacter | 94.48% | 5bc2b17163936e64d74df03b2d1fa89b |
| Genus | Tepidibacter | 94.14% | 435b33b1d5dadd64a9dff21f6745fece |
| Genus | Tepidibacter | 96.96% | 11696b9a1ef0021c7f01738a61a8f598 |
| Genus | Tepidibacter | 99.06% | 845c7b3176ef9e2b5f3c28ee6e916201 |
| Genus | Tepidibacter | 99.68% | 0e565047781f1c5260950b11590acd84 |
| Genus | Terrisporobacter | 96.06% | f45348f0d6f0883229e4018dcd1833a2 |
| Genus | Terrisporobacter | 76.35% | 11c44f60a3c2aa7a050412e9febdfdc5 |
| Genus | Turicibacter | 70.42% | babf2602c0f690daa8ed5b508599ec26 |
| Genus | Turicibacter | 85.78% | dafab221a783cb71aef26728895cef29 |
| Genus | Turicibacter | 85.74% | 2d89dd8eb25ef9a1790ef72b0ada3f0d |
| Genus | Turicibacter | 90.17% | 34e6fd19e5f9bedca53a7583d4048071 |
| Genus | Turicibacter | 95.86% | f995349e094b5af7fac800d07263ee61 |
| Genus | Tyzzerella | 96.52% | 3a341f94ad611cc95846c5dfbac07f48 |
| Genus | Tyzzerella | 97.03% | 45b9a779552178f9299bfdb949c3af76 |
| Genus | Tyzzerella | 97.25% | 9abd42e8353a9cf968c6edea22c45e4e |
| Genus | Tyzzerella | 83.83% | b460ffc9ab060e3c78ba11b6cfb1d138 |
| Genus | Ureaplasma | 99.66% | df805300ebd1fa9ceb0041704d199e55 |
| Genus | Ureaplasma | 99.70% | aed2776416c16a6054dce8112549ca49 |
| Genus | Ureaplasma | 99.60% | 7bd813092e9c5fec15863e4552bf198b |
| Genus | Ureaplasma | 99.87% | 10146ce45a66ad6cb4896c5d41eb304f |
| Genus | Ureaplasma | 99.87% | a68cb87e06cf799f39bbecaece4fe33f |
| Genus | Ureaplasma | 99.32% | 8042e1c6475234395b76e21aed70a608 |
| Genus | Ureaplasma | 99.64% | c354bc65ebb86b247e482e5e035109ba |
| Genus | vadinHA49 | 99.97% | 2571a9b7aebbefe2ac17756f80305729 |
| Genus | Vagococcus | 96.05% | 50a3aa3d6ed5d8c20a9b93ccd65776c9 |
| Genus | Vagococcus | 95.12% | c3d3c5d5a85479be34b4315f5fff9b28 |
| Genus | Vagococcus | 95.95% | 9baa431afbfae866a12bfff7129119ba |
| Genus | Vagococcus | 94.46% | b35f8b826f651b726101dc8c89054518 |
| Genus | Vagococcus | 74.05% | bee4038dce14784c6e5bdf2fc36da531 |
| Genus | Vagococcus | 95.71% | bc59adb01782b5a38450a16a3415e605 |
| Genus | Vagococcus | 96.10% | 8193041a0c7a6f56099b2392f48face0 |
| Genus | Vagococcus | 97.99% | ae729b5d6923a6df405f49dc3029fe56 |
| Genus | Vibrio | 90.69% | 2aa46f67e67aa998b1fb39f2a1cca899 |
| Genus | Victivallaceae | 99.18% | 6cbe1eb1b423f9c1a2cb5b5efd489cdb |
| Genus | Victivallaceae | 99.11% | fac6f4ddb3e7de2f2d662667a7447c16 |
| Genus | Victivallaceae | 99.47% | 021e2428a3a66d553b180d081f8e3502 |
| Genus | Wolbachia | 99.44% | ca03fc34f38b7d4e9d4de7008163c3e2 |
| Genus | Wolbachia | 99.94% | 34546bfcbaae3795e0d2d52e8b8f53ab |
| Genus | Wolbachia | 86.26% | f064e893d32e8653c8b279f641a2238a |
| Genus | Wolbachia | 86.34% | 7cbfa2e7371636b727fca23549558e96 |
| Genus | Wolbachia | 86.26% | 8eee018b82364c42933c33f6abd3b1a9 |
| Species | *Clostridium aldenense* | 95.74% | 3cf04971060de4efe2dfff9c83a10299 |
| Species | *Clostridium aldenense* | 88.78% | dec4828bf7f1f34ad62c01c9cb5bbb4b |
| Species | *Clostridium aldenense* | 88.92% | b11d4b00f280807091e9e17dc174eb4c |
| Species | *Clostridium citroniae* | 81.55% | 5bf10b3d5b03e4283f8126ac533f7511 |
| Species | *Clostridium colinum* | 96.85% | daa8006e086b4f787a0a6f2777969850 |
| Species | *Clostridium colinum* | 96.83% | a31232f45de98fcd91027a7e8592330d |
| Species | *Clostridium colinum* | 96.90% | 8352fc6de0a051149f85948a39e8fae3 |
| Species | *Clostridium colinum* | 77.34% | 235597ff6267481a1d468def854bbfb7 |
| Species | *Clostridium indolis* | 99.83% | 4d2665164e680490c7e757327bb06218 |
| Species | *Clostridium indolis* | 99.83% | 7f3e2cb4928a909ca9e5258ec4fcfa41 |
| Species | *Clostridium indolis* | 97.82% | 8d1f84dde4d96849ea71881adca0dfeb |
| Species | *Clostridium indolis* | 99.83% | 312965339980f0753ce6d8ce1df329b3 |
| Species | *Clostridium indolis* | 99.78% | 07b4ca9d0d3407ccdc2200fe8aa793a1 |
| Species | *Clostridium indolis* | 99.83% | 58b79557c6538d1a0674c50d82f9f8e8 |
| Species | *Clostridium indolis* | 99.78% | 2479780009086d850a0e82ef6ddfb1d1 |
| Species | *Clostridium indolis* | 99.83% | 8e5c67192ebf520a84d97dd17a4676ca |
| Species | *Clostridium indolis* | 99.78% | 6d9e58c475ac414585e1615190bd1cba |
| Species | *Clostridium indolis* | 99.83% | 9d8d6f9d91d3491c7ed9aa76836ac298 |
| Species | *Clostridium indolis* | 99.83% | 0f062497f32c968535e6df5fe4520ac8 |
| Species | *Clostridium indolis* | 99.78% | 14a3b4983685322311a2bacd3b4c3f6e |
| Species | *Clostridium indolis* | 97.22% | 1ab2c2e06c7fc9a09d8171a9a4ab772f |
| Species | *Clostridium innocuum* | 72.49% | 8f5cb34bb9d8328893b2f551c9bfb1a2 |
| Species | *Clostridium innocuum* | 72.40% | 3d5ba9320a759791c81150c1f1b7a229 |
| Species | *Clostridium innocuum* | 72.92% | 1049dc937610b5e5868e9a48e3ef4d23 |
| Species | *Clostridium innocuum* | 71.70% | 10df93e3bef90ec6e479669d74f82970 |
| Species | *Clostridium innocuum* | 77.73% | 60cb9d9044d39a1cc09a9b924b97cf69 |
| Species | *Clostridium leptum* | 81.77% | 1bce05e2acfab9bdfbd2567ab6f1b15b |
| Species | *Clostridium leptum* | 97.37% | d633c18f307e89932c48aff0a1dea5e4 |
| Species | *Clostridium leptum* | 75.60% | f9642eae6f3b30ae5c54fa980a64466b |
| Species | *Clostridium leptum* | 84.74% | 578442aa0bbf40be4e88cc228523cae5 |
| Species | *Clostridium leptum* | 71.51% | a5b7cf7a830893041cefc22cb101a588 |
| Species | *Clostridium methylpentosum* | 87.00% | 7a06461ed0ce4b91a15709bc78f78de3 |
| Species | *Clostridium methylpentosum* | 99.20% | 3d46f2b6df9546253ccef01e8277a763 |
| Species | *Clostridium asparagiforme* | 97.75% | bbbc2d7fb730c182368012a483c4a731 |
| Species | *Clostridium asparagiforme* | 97.34% | 714a4a9a08305ee685f3754cd62f8230 |
| Species | *Clostridium asparagiforme* | 96.62% | b80da528e018b1773d336fcd60f615fa |
| Species | *Pseudomonas geniculata* | 99.56% | 596077a90eff70d548fcc3aa6c3e71a8 |
| Species | *Pseudomonas geniculata* | 100.00% | 05be3e6db58eff0c9db6de2852f00fcc |
| Species | *Pseudomonas geniculata* | 97.08% | 20034f00bd542cbd4b1527fc62275a9f |
| Species | *Pseudomonas geniculata* | 99.12% | 8c92176a13ca8b42e9405302ab90e02f |
| Species | *Pseudomonas geniculata* | 97.72% | f2bb70825515d585f6d637f28e850ab5 |
| Species | *Pseudomonas geniculata* | 88.66% | 88c9434d7a1443636eaaafb5d75e7865 |
| Species | *Pseudomonas geniculata* | 99.99% | 204446f5d2ad2707313c65145b2bddbc |
| Species | *Pseudomonas geniculata* | 92.98% | b4bda873fbed539ab862c1dcaa0c1661 |
| Species | *Pseudomonas geniculata* | 75.42% | 51af03d48f87c112c6280b6ce2233ada |
| Species | *Pseudomonas geniculata* | 90.97% | de25e5239b01d3a5e1dbb4c0b37dc55f |
| Species | *Pseudomonas geniculata* | 100.00% | eaf6ca98a88a0433e0da714aec6d27c8 |
| Species | *Pseudomonas geniculata* | 90.36% | dc878a2482957b6a7f935ba52907eb85 |
| Species | *Absiella dolichum* | 88.87% | 334f83d88c363e626e64e34e58c610b4 |
| Species | *Absiella dolichum* | 89.89% | b3bdc9defbce3a515efd309242d76b8f |
| Species | *Absiella dolichum* | 88.34% | 62768b8f3839012c5eb972b43105ca05 |
| Species | *Absiella dolichum* | 88.31% | 438db8954a125aea1a98b94a97517db5 |
| Species | *Absiella dolichum* | 88.39% | a133b1e849e5e0198348055ee6975a32 |
| Species | *Absiella dolichum* | 72.88% | c5d9c2519138ab07cce805c4704af5de |
| Species | *Absiella dolichum* | 72.83% | 3c65831ab6ead98d979e0517437ccf52 |
| Species | *Absiella dolichum* | 93.87% | 410bc885fe0d602e186e3ef259fd8947 |
| Species | *Absiella dolichum* | 96.52% | 2d7caaab46d2020203061e42c8fe7bc9 |
| Species | *Absiella dolichum* | 91.17% | 7dd1e298a0ce1d972ede37ce561ffebd |
| Species | *Absiella dolichum* | 86.55% | 5f8bcffcea86227702cf8eeb19d89932 |
| Species | *Absiella dolichum* | 89.87% | cfce815ce888e36e5afa8f42c1230c80 |
| Species | *Absiella dolichum* | 89.85% | c38ab6623c25bbe22a2a06c81be4f7b6 |
| Species | *Absiella dolichum* | 88.33% | 3615451297e8e7e897404ff4d031215c |
| Species | *Absiella dolichum* | 88.41% | 8d7d0ff3e7a288dd0b8e6177d4cb5a5c |
| Species | *Absiella dolichum* | 96.85% | 900e8b21b042942378b1be5772206382 |
| Species | *Absiella dolichum* | 88.21% | d5c40fd23a72c319474ea58b15ed516b |
| Species | *Absiella dolichum* | 96.85% | bbbedaf628a6f0540709c1d61f685549 |
| Species | *Absiella dolichum* | 87.17% | 3961ab9ac94dc700ba43c2b7803eec4f |
| Species | *Absiella dolichum* | 93.05% | 50aef9420c6ed8696ff900a09ba8e504 |
| Species | *Absiella dolichum* | 87.88% | 664d3901b7a9ad8349df638b61a744f4 |
| Species | *Absiella dolichum* | 84.62% | 818965aa9b81e5e079bb385ccfd12e77 |
| Species | *Absiella dolichum* | 72.71% | 9288449350e96b53f3eef0b5d314aab5 |
| Species | *Absiella dolichum* | 87.98% | 0c7615bd8905c4b43c097e9ae158aa88 |
| Species | *Acinetobacter sp.* | 99.58% | fa5b55fea1c6c192b78197286f8d27f6 |
| Species | *Acinetobacter sp.* | 99.69% | 2ffdabdde8efd5301eee2fff88bc2e23 |
| Species | *Acinetobacter sp.* | 99.14% | b9a8eee7cbf157178d8824adaadbcb4e |
| Species | *Akkermansia glycaniphila* | 100.00% | 5cd45029801a731610f6320529f9c1c2 |
| Species | *Akkermansia glycaniphila* | 100.00% | 3002d311484f42d7a09e73d45cf6e00e |
| Species | *Akkermansia muciniphila* | 77.05% | 8839c7bf3be14411f1b2b9d60609085c |
| Species | *Akkermansia sp.* | 85.74% | 783944a4addb168cb174acf50068b0d5 |
| Species | *Akkermansia sp.* | 97.30% | f7daa5d8610fd96b0f5e183077342d7a |
| Species | *Akkermansia sp.* | 97.42% | eb45f39d5aed9a81b538a790956ef0e0 |
| Species | *Akkermansia sp.* | 99.57% | 56884b5689141e39d6c1f069f048692b |
| Species | *Akkermansia sp.* | 75.29% | b901d0448f3f44ae4bb5b80e7bdad815 |
| Species | *Akkermansia sp.* | 70.91% | 65f087ca2f21fac3099853ef5985bf3e |
| Species | *Akkermansia sp.* | 95.50% | c5a5c57bc20636abcd54718e9543fd01 |
| Species | *Akkermansia sp.* | 77.48% | 6b6da5a88d3bc3b77902c18c84a454ac |
| Species | *Akkermansia sp.* | 80.72% | f98aa42bc04a790c23fb5c68f38cbd22 |
| Species | *Akkermansia sp.* | 82.32% | 978e8b5c5097493e9c621f43144b60b4 |
| Species | *Akkermansia sp.* | 97.22% | 1e4adf059b658f8b515f7ad8c8c0838f |
| Species | *Akkermansia sp.* | 77.28% | dd1ec67515416ae8564b4a49a44cce0b |
| Species | *Akkermansia sp.* | 91.60% | 56e4ce805435596784fce7159b79b37e |
| Species | *Alistipes finegoldii* | 96.75% | 6bb1c2ab8dcc5a4ea0e69abe121caffa |
| Species | *Alistipes finegoldii* | 99.94% | 06ea02949b85e1c9f11ca5232dfe9893 |
| Species | *Alistipes finegoldii* | 99.94% | ea39fe2e6999675ab3239bfc7ca7f725 |
| Species | *Alistipes finegoldii* | 85.68% | 374b279f2ab2b542c3cbcfeef94c2afa |
| Species | *Alistipes finegoldii* | 96.78% | 7992c919ce19673978e8c2f01cd10055 |
| Species | *Alistipes finegoldii* | 79.43% | 17efe5b615651e14ec8dc87b79890a52 |
| Species | *Alistipes finegoldii* | 99.12% | c6b82f920e0a4d7a6faf28963843da9a |
| Species | *Alistipes finegoldii* | 99.45% | 9d0d7206f7644ea6e5dc39ae0af17c8d |
| Species | *Alistipes finegoldii* | 97.56% | 5182b27f60d2ec5b9ddb020743a9c652 |
| Species | *Alistipes finegoldii* | 97.63% | 400ba0354b6ca7e20d5023619664ee52 |
| Species | *Alistipes finegoldii* | 86.04% | c992bcf879846f4c491eceafffbee0a2 |
| Species | *Alistipes finegoldii* | 98.84% | 56038c720fd769a05efecda0ad4c424e |
| Species | *Alistipes finegoldii* | 95.33% | a5cc1779d7683909b3f83ac63718f4ff |
| Species | *Alistipes finegoldii* | 99.47% | 9ba06f15e941c5665ab9cd5f2c92d4aa |
| Species | *Alistipes finegoldii* | 71.01% | 67c6252a0a5164637f46bd207f4a1466 |
| Species | *Alistipes finegoldii* | 90.32% | 927d3bfc4922f6c773d4185d0d5397f6 |
| Species | *Alistipes finegoldii* | 98.85% | d04f318b899f6d63debdaf71ba31b307 |
| Species | *Alistipes finegoldii* | 86.10% | a6b2e26db3086b2c2fe568ca4c48c96f |
| Species | *Alistipes finegoldii* | 71.90% | 405c7d2e44d5eafa508f78ebae5e9b2f |
| Species | *Alistipes finegoldii* | 99.84% | b437e58f3177fd29d2bd96f54d193ac0 |
| Species | *Alistipes finegoldii* | 99.11% | 1c696c6b3ec7408235919b5247585dfb |
| Species | *Alistipes finegoldii* | 98.65% | 4b1c3724e479de94907ce0fd70bfd2f6 |
| Species | *Alistipes finegoldii* | 99.83% | 255c0e7cfa96ad36a88a1f72fd41968b |
| Species | *Alistipes finegoldii* | 97.44% | b1bd9b0c8a0baace7a64f4e0c44f4675 |
| Species | *Alistipes finegoldii* | 85.82% | 55ef35ae56e61a4bcabeaf4dba6e60aa |
| Species | *Alistipes finegoldii* | 71.90% | 02a5026a7ccd0185e3e5310c8397da53 |
| Species | *Alistipes finegoldii* | 77.07% | c2665e39800bc089247e81d7edb3e990 |
| Species | *Alistipes finegoldii* | 97.14% | f02514a27eef1b400024c9004233aefe |
| Species | *Alistipes finegoldii* | 97.07% | 6d35da43d94e60637ec4262807a124cc |
| Species | *Alistipes finegoldii* | 98.80% | b7be1ba57b89b6fc60a8f17d6a11a350 |
| Species | *Alistipes finegoldii* | 71.07% | 242ced3d18a8c861c31b96bad69cfa85 |
| Species | *Alistipes finegoldii* | 72.29% | c9e86467d5eee655bdffa91d9c3210d7 |
| Species | *Alistipes finegoldii* | 91.85% | 325564fd97bdce78e9e2d01d3056fb90 |
| Species | *Alistipes finegoldii* | 99.94% | 1b065d0086ca688dd4bc872ed4cb1352 |
| Species | *Alistipes finegoldii* | 91.61% | afd3ded2c9634e3a57b992374d1c0121 |
| Species | *Alistipes finegoldii* | 92.61% | 558a3cabcb8e427954e5cede1d9bcade |
| Species | *Alistipes finegoldii* | 93.59% | befe72e66d4993576ead4e34fb5a3645 |
| Species | *Alistipes finegoldii* | 92.85% | 536c0d1115712dd663184d82900f981a |
| Species | *Alistipes finegoldii* | 97.31% | da49ef70f917a7c7004d16cb3884e558 |
| Species | *Alistipes finegoldii* | 78.16% | 7ae9f08666ecb0e6a605e2165f1b7818 |
| Species | *Alistipes finegoldii* | 97.49% | 4d5a904c5f56cffa99a786a47a7c5fef |
| Species | *Alistipes finegoldii* | 86.46% | 7d6c0193b02d27293d30dba340e4519c |
| Species | *Alistipes finegoldii* | 99.45% | 86335ecc286f86220d36aa50abd8f46f |
| Species | *Alistipes finegoldii* | 95.66% | 9a3360fdcc9484f9de7e39ad8b5840c8 |
| Species | *Alistipes finegoldii* | 94.75% | 812838b745c92a163d6753bb9b273da6 |
| Species | *Alistipes finegoldii* | 94.25% | a30f8d62062172049ad029bbc5452ca0 |
| Species | *Alistipes finegoldii* | 97.54% | 9e39f80c98bfeef99084946a90c53cc3 |
| Species | *Alistipes finegoldii* | 97.43% | f7436b5f367d29908ff753a181bcd59b |
| Species | *Alistipes finegoldii* | 97.59% | 1cd345efe023fb93677ca5220b2f0d24 |
| Species | *Alistipes finegoldii* | 90.45% | f1bbb2bc9579f889895b8696c332b6e4 |
| Species | *Alistipes finegoldii* | 99.36% | f2ce2833f3cd222ea5edcb81ef0e59bb |
| Species | *Alistipes finegoldii* | 98.19% | f76e5984ae04f228b239c0664ea58467 |
| Species | *Alistipes finegoldii* | 91.98% | c65f103c0a36c60061e1d71e6b8bc780 |
| Species | *Alistipes finegoldii* | 90.11% | c9cd18c1643c7fb8e95cd4fc93b60772 |
| Species | *Alistipes finegoldii* | 99.94% | 32dec36e2e698fcbd66c79d95b81b3f6 |
| Species | *Alistipes finegoldii* | 96.99% | e14285b42de3f84e310ef0df6380bf96 |
| Species | *Alistipes finegoldii* | 74.97% | f7cab6db463a01f865a7ed83ed37d2ca |
| Species | *Alistipes finegoldii* | 78.80% | e1bd46c4bfe307ccfe291291a203df96 |
| Species | *Alistipes finegoldii* | 99.41% | 2bee7495be2cdff2f0424de0bdc9266b |
| Species | *Alistipes finegoldii* | 87.59% | 845dbb6e1c9993c39be842b42288edab |
| Species | *Alistipes finegoldii* | 85.90% | 2398adcdf1fb299cf36749edaad40b2d |
| Species | *Alistipes finegoldii* | 94.13% | a973f6babe6a3daef6118a7ddca4efd4 |
| Species | *Alistipes finegoldii* | 70.22% | 843ed81b56e6794c22848f4d86af84dc |
| Species | *Alistipes finegoldii* | 74.47% | 223bca4b10b6bbe98677c3962395c108 |
| Species | *Alistipes finegoldii* | 82.40% | 24297711eee7a928bb4b2df25d722191 |
| Species | *Alistipes finegoldii* | 84.63% | ca182035f9e4e20fbd58b7d2dca633fa |
| Species | *Alistipes finegoldii* | 97.09% | 24111e0e286cb5d728ed7a61273b38ea |
| Species | *Alistipes finegoldii* | 72.84% | f3f1c8f7eb3c5db7ba190c14317e5f07 |
| Species | *Alistipes finegoldii* | 78.49% | 3284b4d5bb6212d668ca39d70e7e216b |
| Species | *Alistipes finegoldii* | 99.16% | 02d7d4df9de189933fda3a5d14f577cb |
| Species | *Alistipes finegoldii* | 88.65% | 69f16893dee12e46f09224a434a53159 |
| Species | *Alistipes finegoldii* | 95.28% | ef9e4617739aaa95349f792e0e310079 |
| Species | *Alistipes finegoldii* | 87.95% | 2c83514c4c88ed045b8132aaf86af7e3 |
| Species | *Alistipes finegoldii* | 85.99% | d9f2cd87cc6c52c8c5a721285ee4a8ba |
| Species | *Alistipes finegoldii* | 95.13% | 8a1c20a5083b54e5d9b3c37a2188e5f5 |
| Species | *Alistipes finegoldii* | 75.13% | 43c1c1625110b6aa321ca992f8af95ff |
| Species | *Alistipes finegoldii* | 97.46% | fc04fcb8b6e1edc206e0fecdce3f5a24 |
| Species | *Alistipes finegoldii* | 88.12% | c9edbdfbf1eaa192882cbbc50dbb3a67 |
| Species | *Alistipes finegoldii* | 94.25% | 2dab1c760c93a00622c9bddf7458039a |
| Species | *Alistipes finegoldii* | 86.84% | 3ee371bb54dccfcad90092593c9bdd13 |
| Species | *Alistipes finegoldii* | 93.00% | 8c935d9718b2911d173655e430634409 |
| Species | *Alistipes finegoldii* | 91.90% | 46ac4a7851d8f7c679269f1f5641507f |
| Species | *Alistipes finegoldii* | 91.99% | 9c6366d54bc72a375824aaff14d68c07 |
| Species | *Alistipes finegoldii* | 85.20% | 612dbf3100dcd12fc9f38eb26e6b6cab |
| Species | *Alistipes finegoldii* | 73.62% | b261440befd6f5f395a2678745403f08 |
| Species | *Alistipes finegoldii* | 84.48% | 5c50977cdcfc0571253a3b35bc0eba35 |
| Species | *Alistipes finegoldii* | 88.72% | 90ebdfd94429a3481b1e82d73349c51c |
| Species | *Alistipes finegoldii* | 76.51% | ae050290f3cfd54e69829f2951537bcb |
| Species | *Alistipes finegoldii* | 98.86% | 7e4af60f4971816168e4bf4b42524d56 |
| Species | *Alistipes finegoldii* | 70.60% | 5f2a9dd721fa796a0b6b3fe473feec9a |
| Species | *Alistipes finegoldii* | 99.44% | 299eed6a9802cf9568b1f785c7b56fe5 |
| Species | *Alistipes finegoldii* | 99.50% | dc15e82a2d1efae6cbfe803f3782f843 |
| Species | *Alistipes finegoldii* | 87.88% | 3cfa4a70c1668178b4882374e63cffe2 |
| Species | *Alistipes finegoldii* | 99.54% | 67ce3e1ba472644531efe3a181fa335c |
| Species | *Alistipes finegoldii* | 96.39% | c8455dd952f0901d88f3294f7a51a41d |
| Species | *Alistipes indistinctus* | 76.53% | a45256a7cf4bf8208c06adcfd7462c37 |
| Species | *Alistipes indistinctus* | 98.36% | 2d4fd699e7e3954b1bd2c1e71594ba53 |
| Species | *Alistipes indistinctus* | 99.99% | a325c8bc110b05b06aec7c8b45437b05 |
| Species | *Alistipes indistinctus* | 99.99% | 280d6b5a2b0a62b4ca054dabe0dd26d6 |
| Species | *Alistipes indistinctus* | 99.99% | 4b1c9185a45d51a6cff4f1bb45dca8f3 |
| Species | *Alistipes indistinctus* | 99.48% | c6dc66837b241d5811f59d428439f532 |
| Species | *Alistipes indistinctus* | 99.90% | 55cea8bc686cc45b152e06c02f876579 |
| Species | *Alistipes indistinctus* | 99.99% | 18932dc1a1873a11be83d83816d18be2 |
| Species | *Alistipes indistinctus* | 98.91% | 51df1a648654f0b0c92e8564bd141760 |
| Species | *Alistipes indistinctus* | 99.99% | 4edcfdf700056826e3bbe9a10c603e63 |
| Species | *Alistipes indistinctus* | 99.29% | 9f1e2fc43e19add30990f4a5ac2d5b7d |
| Species | *Alistipes indistinctus* | 99.92% | a349de4839eb5c15d7d4c4293973bcc0 |
| Species | *Alistipes indistinctus* | 99.99% | 521197c7e3b5f48d16e59354b3431256 |
| Species | *Alistipes indistinctus* | 99.99% | f70272a36c9974ac0ef4f1b7c115689b |
| Species | *Alistipes indistinctus* | 99.41% | a64996a368056a2b918ec625af507b03 |
| Species | *Alistipes indistinctus* | 97.32% | 24e3e3fb50c31f0d6b6183f8b6da6b7b |
| Species | *Alistipes indistinctus* | 99.99% | 2a079d6d6ada6521f4bf55823f37ffa2 |
| Species | *Alistipes indistinctus* | 99.99% | b0b6f7565122f66aed0ea11ac0459470 |
| Species | *Alistipes indistinctus* | 99.97% | 73c23734e7ba1711cbe57a3cab11a0b3 |
| Species | *Alistipes indistinctus* | 99.85% | 8948807c5e67d40a5c1ab88dfbbbfb3d |
| Species | *Alistipes indistinctus* | 72.42% | f192d8420d7f3ce942c2081118c616f4 |
| Species | *Alistipes indistinctus* | 99.99% | e14e1ce6f03ea20d0367e4e801062760 |
| Species | *Alistipes indistinctus* | 89.05% | 609c251b0a8842e04aeb1036c05b52f4 |
| Species | *Alistipes inops* | 100.00% | 29ef0334b09b1af14e3fd6fde2169145 |
| Species | *Alistipes inops* | 71.04% | 5a7cd160c44d53bace231df1319da24f |
| Species | *Alistipes inops* | 100.00% | 20348cc1e85ff03de426d711beac0df1 |
| Species | *Alistipes inops* | 100.00% | ee6b53c988f4d2c5baeedac7fe0fa7fc |
| Species | *Alistipes inops* | 76.66% | 49ef66bc177d0765c8e108543f21cd44 |
| Species | *Alistipes inops* | 99.97% | ab0998c2d237a06c4d4978c589a7a5a1 |
| Species | *Alistipes inops* | 99.17% | 5b7d2230400547b121c8cda42502356e |
| Species | *Alistipes inops* | 100.00% | 4b9bb2be1417d3b1ae92bf08739c879d |
| Species | *Alistipes obesi* | 99.86% | b75ff32bfd668ec899938bc60b878262 |
| Species | *Alistipes putredinis* | 95.13% | bb7994122da70b9e174dcd756fec42d6 |
| Species | *Allofrancisella frigidaquae* | 91.46% | b1bc6e6711a8f38f6b7c467ab29eba9c |
| Species | *Anaerofustis stercorihominis* | 80.56% | 22e058095f6e1295e02b4697e6be6922 |
| Species | *Anaerofustis stercorihominis* | 80.56% | 4d38ec959cb57db603328de483f06003 |
| Species | *Anaerorhabdus furcosa* | 99.95% | 1ff408d338276f23049cc6d3b32a7b66 |
| Species | *Anaerorhabdus furcosa* | 99.94% | 239c723fb82f2e5df6038a88206711f8 |
| Species | *Anaerospora hongkongensis* | 99.86% | 77ba755a37a1ea9a0c4b8cbb7f3b648a |
| Species | *Anaerospora hongkongensis* | 99.88% | ed80c5f65cf04a0e18ed0d5f97800ddc |
| Species | *Anaerospora hongkongensis* | 99.87% | 186878c42d2b0f085246299509596d5e |
| Species | *Anaerospora hongkongensis* | 99.88% | 91a553fae8d49992e2c04c5fac34fb80 |
| Species | *Anaerosporobacter mobilis* | 98.92% | c7c2719cbaa81b9338d7dda122ea0c32 |
| Species | *Anaerosporobacter mobilis* | 98.88% | 553a1c0eb0d270f94f4776b001bf71d0 |
| Species | *Anaerosporobacter mobilis* | 80.18% | 0b7a6bff4dc467d17ce46b2926246601 |
| Species | *Anaerostipes rhamnosivorans* | 89.82% | 98659a69be6790a0e203e476eed355dd |
| Species | *Anaerotignum lactatifermentans* | 77.23% | 924de452ba3a781083567d1eaa01e7b3 |
| Species | *Anaerotignum lactatifermentans* | 75.91% | f4fec63b82e950b97907a8af0e0eea95 |
| Species | *Anaerotignum lactatifermentans* | 75.91% | 8cd2ebabb4a4eb51a69db8bfb817de04 |
| Species | *Anaerotignum lactatifermentans* | 77.28% | bfa8a94e3a99b6845fd13704f8bd8f6f |
| Species | *Anaerotignum lactatifermentans* | 79.32% | 6c63ae0a8b9f7afdeca7fc0e2516faeb |
| Species | *Anaerotignum lactatifermentans* | 83.10% | 6ba28cf67399f1d147e070b1280339dd |
| Species | *Anaerotignum lactatifermentans* | 83.10% | 4fa2d8ede2eceebe6f7a859829fba1b6 |
| Species | *Anaerotignum neopropionicum* | 87.98% | 7be4783bd8e8d5baa0feeb7a18be41c8 |
| Species | *Anaerotignum neopropionicum* | 98.02% | 4183325bc2f9ddf867cbfe17ef159a41 |
| Species | *Anaerotignum neopropionicum* | 97.69% | 2f1471c6c7faefa0d57ea2e22b105224 |
| Species | *Anaerotignum neopropionicum* | 97.64% | 9dccaf74c64950946af943f8c6843fdc |
| Species | *Anaerotignum neopropionicum* | 86.73% | 0e9030ef41807b9e12dd6111d21e983c |
| Species | *Anaerotignum neopropionicum* | 87.94% | 9c991996f46716ec82cd291ef8dd65c9 |
| Species | *Anaerotignum neopropionicum* | 97.70% | 9a394643bba8f8bd89478b229d6b8b67 |
| Species | *Anaerotignum neopropionicum* | 97.64% | b921571f086789cd46a8579ed91ca7c6 |
| Species | *Anaerotignum neopropionicum* | 80.02% | 60cf8a0b9a0a177b1c563e91ae537886 |
| Species | *Anaerotignum neopropionicum* | 97.60% | c5313cdbebcda84cf3afbf1766864404 |
| Species | *Anaerotignum neopropionicum* | 88.15% | e3263fd694c595b8146a8814d9a0acf0 |
| Species | *Anaerotignum neopropionicum* | 97.84% | ee71cccdfa9e82d37fbea56fb636f753 |
| Species | *Anaerotruncus sp.* | 71.46% | 36bf7ab705225c190efb77052e469861 |
| Species | *Arthrobacter luteolus* | 98.50% | 138040a40b03a0aa26a0add55a2b4dc7 |
| Species | *Asaia lannensis* | 99.72% | e5372ac01ad36ccaab833fbf7bea7507 |
| Species | *Azospirillum sp.* | 99.97% | 512c2325054f3bb368273de820bfb52e |
| Species | *Azospirillum sp.* | 91.64% | 42343d2b51d340bcdc8d4bb981e3c26f |
| Species | *Azospirillum sp.* | 99.95% | ac271ff68bc5303b095240d47f232f33 |
| Species | *Bacillus andreesenii* | 72.87% | 96bad1ba1a63b7b54f78cc48d287f0f9 |
| Species | *Bacillus andreesenii* | 72.81% | 4a9b713dcc265c7c048c660a327bd2f6 |
| Species | *Bacillus andreesenii* | 92.88% | 83ffbf1bbb25889e17ec9b45a13b29ba |
| Species | *Bacillus endoradicis* | 99.87% | 3b6d186d130fa8becd97d722ccdc92e9 |
| Species | *Bacillus endoradicis* | 99.88% | ce4143907c49c9e5a17dd70c3f402bf3 |
| Species | *Bacillus endoradicis* | 75.03% | d5c2fd10cfc1b7ad25df2189fbc11510 |
| Species | *Bacillus endoradicis* | 99.93% | 7437b6fd491152c9e830799484ffa3b3 |
| Species | *Bacillus endoradicis* | 74.49% | 151c686ccbaa3a5f3723ee4600b97a5c |
| Species | *Bacillus endoradicis* | 99.55% | 99fa33aae9ff440119aebc28193ba632 |
| Species | *Bacillus endoradicis* | 71.11% | f17163853b5e720aee1e8355cd1aacc1 |
| Species | *Bacillus endoradicis* | 99.57% | 2b7aa7e65bd1da54a7a06dff30d3bb52 |
| Species | *Bacillus endoradicis* | 99.93% | d0140b2a7f27431569101701af2dca5b |
| Species | *Bacillus endoradicis* | 74.48% | 8f34d660cd343c55c2f960f80ba2022e |
| Species | *Bacillus endoradicis* | 99.83% | 6ccdf816922db1319651cc69aa025b96 |
| Species | *Bacteroidales bacterium* | 99.54% | ed53216120c55b7f5200a1a2d24f69e7 |
| Species | *Bacteroidales bacterium* | 75.45% | 59d2b5acd52c73fc4f6064779b41f588 |
| Species | *Bacteroides acidifaciens* | 81.55% | e821036bc30d579cac9065333d2f601c |
| Species | *Bacteroides acidifaciens* | 81.59% | ab6fbea079385cca432d72958cb1d145 |
| Species | *Bacteroides acidifaciens* | 81.57% | c9a7f72e762cf0d5d66b1e104c176370 |
| Species | *Bacteroides acidifaciens* | 85.01% | a62beb8c113721b0362097c476ddf049 |
| Species | *Bacteroides acidifaciens* | 79.60% | 47eb7faba539cdf4133028a7a46e4163 |
| Species | *Bacteroides acidifaciens* | 79.59% | b066a02055bf67cfee19b3c236683f44 |
| Species | *Bacteroides acidifaciens* | 79.41% | 0be37113362af2a31328c19b03c875ee |
| Species | *Bacteroides acidifaciens* | 80.86% | 7f2d419c9443cd14017f13e61ccc453c |
| Species | *Bacteroides acidifaciens* | 79.44% | 8424856ec3fd24b0d6ae604715fb24b3 |
| Species | *Bacteroides acidifaciens* | 79.54% | 47c650ba368d2360f3675b64f84eb3f9 |
| Species | *Bacteroides acidifaciens* | 79.42% | ca83708323cf359f56bb98db67981885 |
| Species | *Bacteroides acidifaciens* | 78.26% | 6109037cbcf646c45e183cc0e3aa80c3 |
| Species | *Bacteroides acidifaciens* | 79.78% | 0783280deec26b728d207e0fad31e85d |
| Species | *Bacteroides acidifaciens* | 80.16% | e89c63cea290f146232eefd144803990 |
| Species | *Bacteroides acidifaciens* | 71.86% | dcb9633a6e6bb38a42f84c2fe1d9ecfa |
| Species | *Bacteroides acidifaciens* | 80.96% | 066f5dbbefa6a404904ea414a1c3cce8 |
| Species | *Bacteroides acidifaciens* | 84.96% | a4a5ab2c114abb89ca64a401f815d05f |
| Species | *Bacteroides acidifaciens* | 74.26% | 70682ca012f8c432a6d84a3dee1e6ac3 |
| Species | *Bacteroides acidifaciens* | 80.89% | f443549732033a0662336f5ff8836208 |
| Species | *Bacteroides acidifaciens* | 82.27% | 73640a4d41a9ad0b127005e8c3284d7e |
| Species | *Bacteroides acidifaciens* | 79.51% | cb96fa244e48896a7e829b403164f3d1 |
| Species | *Bacteroides acidifaciens* | 89.70% | 0c2fa392a20f959d60b94ac5a071c667 |
| Species | *Bacteroides acidifaciens* | 82.12% | 20f6c47e46c9cbc9ad63cc295eec65f2 |
| Species | *Bacteroides acidifaciens* | 75.42% | 3b07e4d665285987c1ae853b926de1f7 |
| Species | *Bacteroides acidifaciens* | 78.79% | 4f9924be2db854489ae707283c5bd8ec |
| Species | *Bacteroides acidifaciens* | 82.04% | a16789c891a1e5178759236f70684dc2 |
| Species | *Bacteroides acidifaciens* | 78.00% | d39133aa42adc2ed55e1c74d8400ed1b |
| Species | *Bacteroides acidifaciens* | 89.40% | e3a02299a0d4140a5b1e6109f99bdcb5 |
| Species | *Bacteroides acidifaciens* | 89.39% | 3d8a9a6af67ff64b73e9f80c3995ae09 |
| Species | *Bacteroides acidifaciens* | 89.38% | d2e1bf18d1e26146bcd86d76c2bb72ac |
| Species | *Bacteroides acidifaciens* | 89.06% | 76582387e0a384801169a82d0bc673fb |
| Species | *Bacteroides caccae* | 92.91% | a71e6b41eb5f0a97265cd641368247e5 |
| Species | *Bacteroides caccae* | 92.64% | a7c8f01e771df2f51e58e004767e43ec |
| Species | *Bacteroides cellulosilyticus* | 85.60% | ef2f7facc065cabd752e073af035f4a9 |
| Species | *Bacteroides cellulosilyticus* | 99.82% | d0bcdb55d335747e48e3d16fd2ac38e8 |
| Species | *Bacteroides cellulosilyticus* | 99.82% | 3af6a3988d36400e51df6e683a94f460 |
| Species | *Bacteroides cellulosilyticus* | 99.82% | a44694ebef199f2cf69d394ef804d170 |
| Species | *Bacteroides cellulosilyticus* | 79.25% | 7b2e2e3a0ac9e893a28c1cb82c8f6c0d |
| Species | *Bacteroides cellulosilyticus* | 81.40% | 2ce04dfc0703d16412d978f83ecb5931 |
| Species | *Bacteroides cellulosilyticus* | 74.63% | 1ec6cd4a60b7b0b1554e92ec14d081c9 |
| Species | *Bacteroides cellulosilyticus* | 72.31% | 670863d105bfaa338949001636071d0d |
| Species | *Bacteroides cellulosilyticus* | 89.58% | 23b33ab1b0f740c8cbe248f936e84f46 |
| Species | *Bacteroides cellulosilyticus* | 74.45% | ac9e6031042899835d57081969cf2caf |
| Species | *Bacteroides cellulosilyticus* | 74.35% | 5e5d127989f3779f4cfd36f83908cf10 |
| Species | *Bacteroides cellulosilyticus* | 76.67% | cd875e5e989d2d18e59dff2a2010b20a |
| Species | *Bacteroides cellulosilyticus* | 80.45% | 78895cc1b4f3667a0ca64b9c93accdc9 |
| Species | *Bacteroides cellulosilyticus* | 85.27% | 81d8b09deb95c7606e07bae2ab2118c0 |
| Species | *Bacteroides cellulosilyticus* | 74.35% | 14a092832db23e03c6bd23a841180f81 |
| Species | *Bacteroides cellulosilyticus* | 82.72% | f4c5be52d47243d787bcaa17362ce3bc |
| Species | *Bacteroides cellulosilyticus* | 79.41% | 9879e134f97f0a04d96edbaf38491238 |
| Species | *Bacteroides cellulosilyticus* | 87.10% | 9f7a0b06427eb4cd5f708c6da96f3b25 |
| Species | *Bacteroides cellulosilyticus* | 73.17% | ac39363084c98cf16bdce5b150cdaf5e |
| Species | *Bacteroides cellulosilyticus* | 83.86% | bcd9058fa4ba892321e7fe07b2c4b280 |
| Species | *Bacteroides cellulosilyticus* | 75.05% | 6108701fb19c7f4578c86a5867961863 |
| Species | *Bacteroides cellulosilyticus* | 99.82% | b03158d7107d5bdde5b89b8f86fe70b6 |
| Species | *Bacteroides cellulosilyticus* | 76.70% | dbcaaaffe7bbd9dedac8c3fcce5d488b |
| Species | *Bacteroides cellulosilyticus* | 77.85% | 28d897dedd4e20cc855022e83515b940 |
| Species | *Bacteroides cellulosilyticus* | 79.10% | c6ca5324979ef35b95ca78d856142f42 |
| Species | *Bacteroides cellulosilyticus* | 70.07% | 623edf30f06537cf765e605d75dea820 |
| Species | *Bacteroides cellulosilyticus* | 99.87% | e0e3cc55b12e9735fe1e00de1a0dd8de |
| Species | *Bacteroides cellulosilyticus* | 81.19% | 75ea1595413b2871e15e266866e3dce8 |
| Species | *Bacteroides cellulosilyticus* | 81.58% | d35e0edc489f12761382af6b5e1b48ea |
| Species | *Bacteroides cellulosilyticus* | 81.49% | 668dd0926cd0fefd86f52e40f928a40e |
| Species | *Bacteroides coprocola* | 76.71% | a8764a38a417d03ada86148f88015b48 |
| Species | *Bacteroides eggerthii* | 89.82% | 2405932b3bbcff706e5f477389f53027 |
| Species | *Bacteroides fragilis* | 90.62% | fe5b418503c5f787239f990d99de3f6b |
| Species | *Bacteroides fragilis* | 90.11% | b31fc9c51794bc3d84c8b54551c804d7 |
| Species | *Bacteroides fragilis* | 71.78% | a13aceb493549f5e9bc092d146fd8bd9 |
| Species | *Bacteroides fragilis* | 71.79% | 903e63c7bf9ff0f0b81f7b62f4e17679 |
| Species | *Bacteroides fragilis* | 71.71% | 4422d24bc0e1babfa1be7daadfefbf36 |
| Species | *Bacteroides massiliensis* | 98.51% | 28986cfe4a9fd0a7285e4db1b8cf4c73 |
| Species | *Bacteroides massiliensis* | 99.96% | 54009b961176e2118ebf482b307a8412 |
| Species | *Bacteroides massiliensis* | 99.80% | 5e14e422d8aa644776894cb28f254fd3 |
| Species | *Bacteroides massiliensis* | 99.80% | 253832148f87bc1f7d507df708b35a75 |
| Species | *Bacteroides massiliensis* | 99.80% | eef2b791a5c6ca1fb51d5eb4d3660fe7 |
| Species | *Bacteroides massiliensis* | 99.96% | a26f52cc4061e55e87e6c691f27309b1 |
| Species | *Bacteroides massiliensis* | 99.96% | 6e19336a4b3d113042d74b32dbbc3f36 |
| Species | *Bacteroides massiliensis* | 99.92% | 1efdaae73a7d361fe1effc66fa19c48b |
| Species | *Bacteroides massiliensis* | 99.99% | ee2bf429bd630236c461e79f6fa8e36c |
| Species | *Bacteroides massiliensis* | 99.81% | 42cc249c8783e87ef7df5a811d01c02d |
| Species | *Bacteroides massiliensis* | 99.90% | 0b3da86f5c86ff8eda088aa477855093 |
| Species | *Bacteroides massiliensis* | 80.19% | 872d16a6b0b89ce420e2c0a49d98458b |
| Species | *Bacteroides massiliensis* | 91.78% | ed1df13db44b2be1568e6d8688df6c81 |
| Species | *Bacteroides massiliensis* | 99.96% | 35690e6b3f221cf653cf94b632169de3 |
| Species | *Bacteroides massiliensis* | 99.96% | 580cca834e5587e60effdf08246f629c |
| Species | *Bacteroides massiliensis* | 99.83% | c6dddbcd0c4a25ceffd29833e9a17a5e |
| Species | *Bacteroides massiliensis* | 99.81% | 00325e85bc9a92c58f48929216d1f558 |
| Species | *Bacteroides massiliensis* | 99.91% | ddd9c7ab039c19ae57de4042b3aa783a |
| Species | *Bacteroides massiliensis* | 99.99% | 2037cd0914c69446c1ca69d5cf0a4451 |
| Species | *Bacteroides massiliensis* | 99.81% | d98d337ab7b51158758fa7aa4f659ddb |
| Species | *Bacteroides massiliensis* | 98.71% | 5c715936ff68865e2a4dbc6156340be3 |
| Species | *Bacteroides massiliensis* | 99.59% | fd4dbcb5446f40c7a4d079d915f268f0 |
| Species | *Bacteroides massiliensis* | 99.99% | e93e5b09719834b90eb177a679df62ac |
| Species | *Bacteroides massiliensis* | 90.34% | ca6c586b49df77ea6d780f4d3d7bfb46 |
| Species | *Bacteroides massiliensis* | 99.58% | b53916a20dddeb06a29e0eaaace8818b |
| Species | *Bacteroides massiliensis* | 71.12% | a6bcdf15bb1796f69964d95acce441d4 |
| Species | *Bacteroides massiliensis* | 99.81% | f7928c46a15c41869a153c717600df78 |
| Species | *Bacteroides massiliensis* | 89.29% | a1a35834df6b654c654e11ec1a1a1302 |
| Species | *Bacteroides massiliensis* | 99.96% | f348df1f5abd194a5e3a312bea26b9f6 |
| Species | *Bacteroides massiliensis* | 99.81% | 3b0dd4476a22976154d62eed8d4d6440 |
| Species | *Bacteroides massiliensis* | 99.81% | 99435f9eb257e4863e070404f2e7799d |
| Species | *Bacteroides massiliensis* | 99.83% | 85408902135000620a5afbb523d660bc |
| Species | *Bacteroides massiliensis* | 96.83% | bcec39ae8bd544d7c5b3fd48eb68540c |
| Species | *Bacteroides massiliensis* | 99.81% | c398f217125c943d5ffc15b504679f01 |
| Species | *Bacteroides massiliensis* | 99.96% | fb6e5ec7c18621911838866f792dbf78 |
| Species | *Bacteroides massiliensis* | 99.82% | 3bcb368ec837169248a2d771bea31c36 |
| Species | *Bacteroides massiliensis* | 99.99% | 65d6c96f1bbc0980f2f60b96fa65b292 |
| Species | *Bacteroides massiliensis* | 99.81% | 57ef56843d7cbad323c574a368752f6f |
| Species | *Bacteroides massiliensis* | 99.81% | bde970950e8d084c97ad2413c94a3587 |
| Species | *Bacteroides massiliensis* | 90.44% | 1b89cc10c66d961e5f502ffb3bc59501 |
| Species | *Bacteroides massiliensis* | 99.80% | 72bb775e32dc1dc7da4c94d256a25b0c |
| Species | *Bacteroides massiliensis* | 99.81% | 4234637c2a6b20563c7d87f5d5ebe28f |
| Species | *Bacteroides massiliensis* | 99.96% | 99904625505aa80db725b75879aaecde |
| Species | *Bacteroides massiliensis* | 99.93% | 6ac1ee3c5230f85aea68bb1898291648 |
| Species | *Bacteroides massiliensis* | 99.86% | 81ca64cf69bfbe24b463567005a64e58 |
| Species | *Bacteroides massiliensis* | 95.29% | f2ffd164d2e22cbfe64994f6bae4c03f |
| Species | *Bacteroides massiliensis* | 98.41% | 628102f67e1034c7f19a794778dd50d1 |
| Species | *Bacteroides massiliensis* | 98.58% | 6ae403f1c5284a3ddaaaaec475f9e136 |
| Species | *Bacteroides massiliensis* | 99.96% | b50b324ad79f87f8c92a2a16aa37602e |
| Species | *Bacteroides massiliensis* | 92.60% | dd473b13cfb1b178efb5eb33f27ee54a |
| Species | *Bacteroides massiliensis* | 90.49% | 14da174fb9a93bdbae3b32086a9d6678 |
| Species | *Bacteroides massiliensis* | 91.67% | 12613cf411a1d83e95e58e98ebb394a3 |
| Species | *Bacteroides massiliensis* | 89.61% | 5aaa89296763c7bb37a92881e27197e7 |
| Species | *Bacteroides massiliensis* | 98.77% | 69ce092838736517570d6eee1eaad6ce |
| Species | *Bacteroides massiliensis* | 99.99% | 907b7f387d8c59176c9f256fa9a3fd8a |
| Species | *Bacteroides massiliensis* | 99.99% | 7bc00cf11ea2be9ce540e76f67ac22d9 |
| Species | *Bacteroides massiliensis* | 99.96% | c63f824ef0841f1ec86b46d057d7dde3 |
| Species | *Bacteroides massiliensis* | 99.96% | 95f660524735ed996ab3a9645bf85998 |
| Species | *Bacteroides oleiciplenus* | 77.87% | 26abae31f1c0177764091e8b57825cd0 |
| Species | *Bacteroides oleiciplenus* | 78.64% | 8c8b91546bc862c05001cab18d8f1a35 |
| Species | *Bacteroides oleiciplenus* | 77.93% | 6d4cd1124e95ac8e56422a6bd35d0422 |
| Species | *Bacteroides stercoris* | 97.25% | ed3b01776cc98c5b6282cde2dd1f0569 |
| Species | *Bacteroides stercoris* | 97.25% | 90fa58c0af0074799cc3f65b104da5c5 |
| Species | *Bacteroides stercoris* | 97.26% | 97f570c2c0491e8acb048dfe3f946132 |
| Species | *Bacteroides thetaiotaomicron* | 86.75% | ed86b6756153aa6454e7914af7c84982 |
| Species | *Bacteroides thetaiotaomicron* | 86.77% | cc7ab79329a59043e3aed5db8202bdb1 |
| Species | *Bacteroides thetaiotaomicron* | 88.72% | 9a0ee36ac000592e1a5e4e50ca32e234 |
| Species | *Bacteroides thetaiotaomicron* | 84.06% | e7a2c1b4f99a8a91222620256a958887 |
| Species | *Bacteroides thetaiotaomicron* | 83.52% | d560110898b3e982732df0074a1ff0f9 |
| Species | *Bacteroides thetaiotaomicron* | 86.50% | ffc9d6260c1c4b34c8e7fa65163da80b |
| Species | *Bacteroides thetaiotaomicron* | 82.37% | 5d9d42b148ee37c17e1b239ada0a42ab |
| Species | *Bacteroides thetaiotaomicron* | 82.09% | 263fb9ce52fd28dbe12c29fc49b53e80 |
| Species | *Bacteroides thetaiotaomicron* | 86.73% | 2a9bd9afbf9b70fc005112cc27eaa011 |
| Species | *Bacteroides thetaiotaomicron* | 86.73% | d139e6a3368a2b5b255dbfe4122ff1ed |
| Species | *Bacteroides thetaiotaomicron* | 86.80% | c84e42828cb2a0e32333bfe2c4cb8afc |
| Species | *Bacteroides thetaiotaomicron* | 88.55% | f7d8bfac03d692e813cf5c006eac0e79 |
| Species | *Bacteroides thetaiotaomicron* | 88.56% | 87a126d956ea44447f4661c38b1ffdbc |
| Species | *Bacteroides thetaiotaomicron* | 88.46% | 9a0b1e6746e34bf86aedd13b0de691ee |
| Species | *Bacteroides thetaiotaomicron* | 88.58% | 723d774bd5206215063ab601af30a709 |
| Species | *Bacteroides thetaiotaomicron* | 88.48% | 166f2e9193e39601e91b2ba229e5fa9e |
| Species | *Bacteroides thetaiotaomicron* | 88.59% | d833ee1775a1cff4caa4bd6494b384f7 |
| Species | *Bacteroides thetaiotaomicron* | 88.57% | 6a05e9049864228647b1f097334dcad4 |
| Species | *Bacteroides thetaiotaomicron* | 88.47% | 08552559d56a4495170467d8e6fe6304 |
| Species | *Bacteroides uniformis* | 99.74% | 6043f577a701f7a5bce15ad550dafd8b |
| Species | *Bacteroides uniformis* | 97.89% | 013b9c1d7c56343ab55191e3fbd1ae13 |
| Species | *Bacteroides vulgatus* | 77.75% | f5d642a427b449de21a21302802704d6 |
| Species | *Bacteroides vulgatus* | 83.69% | ff460c274e6a11c986e6bafe7b741eb8 |
| Species | *Bacteroides vulgatus* | 75.57% | fdf77063760b1dffea9a136fbc5e75f4 |
| Species | *Bacteroides vulgatus* | 76.30% | c15b37130a01b6dde0e6351324e00613 |
| Species | *Bactoderma rosea* | 99.62% | 6d49a47d066725c1c12e4f527f4953ea |
| Species | *Bilophila wadsworthia* | 92.43% | 9f621a6d7269a769c43961b6b3ffd8ed |
| Species | *Bilophila wadsworthia* | 92.43% | ed3cdfd8d2a594f9f2122b553672a1df |
| Species | *Bilophila wadsworthia* | 92.43% | b0368cd69648f05687c6d4f8909b6f4d |
| Species | *Bilophila wadsworthia* | 90.44% | eabab2163d62874275667c0a0e9fbbfb |
| Species | *Bilophila wadsworthia* | 93.81% | 1099d575a06cb0334d64aa81dab82e1e |
| Species | *Bilophila wadsworthia* | 91.87% | ff9a07aecd623c622de31b4aa6728f25 |
| Species | *Bilophila wadsworthia* | 87.14% | 8383bb9fc6ff5258f725ccd2df66894c |
| Species | *Bilophila wadsworthia* | 93.55% | 2d5354deadd6ab25a1614db948be19be |
| Species | *Bilophila wadsworthia* | 90.72% | 4518058adda62637a99659e4c0a1041d |
| Species | *Bilophila wadsworthia* | 97.28% | e6d5edbab917c51e0dd18fa64c3599ae |
| Species | *Bilophila wadsworthia* | 91.69% | 1471668b5f1787bfaa4d8e8d91f29947 |
| Species | *Bilophila wadsworthia* | 96.23% | 622267df3e1380fdfe09677dd88ed0ea |
| Species | *Bilophila wadsworthia* | 96.11% | e0e60bb50663d60e434731ee284e09a7 |
| Species | *Blattella germanica* | 92.78% | 9e136cb88e1e7c769a250bf3a31cf54a |
| Species | *Blattella germanica* | 95.00% | e4a2f4cdbadb95f1b696959ab58c5688 |
| Species | *Blattella germanica* | 88.46% | 79da0b042043a702e93ceef9c02f7b4a |
| Species | *Blattella germanica* | 95.67% | bfd00f20771ebcb3ab2adc8d3daf521d |
| Species | *Blattella germanica* | 94.71% | 3ae773a413e1a98a0d947f9b943a5e3f |
| Species | *Blattella germanica* | 98.88% | 93008cb27ae56a897acf5b06734436d1 |
| Species | *Blattella germanica* | 94.30% | 928eed810f0d257d66cccf282857d44c |
| Species | *Blattella germanica* | 94.67% | 50188999c149124c73ec107b8f3cf708 |
| Species | *Blattella germanica* | 95.35% | 4d16f47e25b10514179fc48b7b98a6f7 |
| Species | *Blautia stercoris* | 88.76% | 593fc148eb16b7f635c69c5142a66bee |
| Species | *Blautia stercoris* | 88.52% | ea239a9c7a095389e9013bde18c11a0f |
| Species | *Blautia stercoris* | 88.68% | 8f4f691a0c7569fea33cab5e95128944 |
| Species | *Blautia stercoris* | 83.60% | 5fb6352a1b30f580b8d92a214697961e |
| Species | *Blautia stercoris* | 84.50% | 9d51138c6f15fd9ea8513d2b201b7306 |
| Species | *Blautia stercoris* | 88.73% | 31a021370f4baac6855687aeed711a8a |
| Species | *Brachyspira sp.* | 95.81% | 765157377bcc90d8155a3460bf4fd595 |
| Species | *Brachyspira sp.* | 96.45% | 56341489588cb4ed0175406821b3a0ab |
| Species | *Brachyspira sp.* | 96.46% | 7f5f9c4ccd89526573e44c7c183beae0 |
| Species | *Brachyspira sp.* | 95.65% | cd28e7dbc349b09cd292d917452a2081 |
| Species | *Brachyspira sp.* | 95.67% | 6e59b3d7bf97e0618021c8f36c9c5a88 |
| Species | *Brachyspira sp.* | 95.84% | 0ca9aa87ad1a6a2a749c27c119c9e5f6 |
| Species | *Brachyspira sp.* | 95.42% | 7259e65dcaabef2e6be261d7366b7209 |
| Species | *Brachyspira sp.* | 95.76% | 4762eae6ddeed7ce14d5e4894b00915c |
| Species | *Brachyspira sp.* | 95.65% | 7d1809bdf92525512ef12973b462b02a |
| Species | *Brachyspira sp.* | 96.34% | c3180d99077104d202cf4dd7fe22ff62 |
| Species | *Brachyspira sp.* | 96.46% | 0d56c94f4fb3c18218e4d527e4b009fa |
| Species | *Brachyspira sp.* | 95.63% | 3ffdb24dfd2eee2a065e25746a41884d |
| Species | *Brevibacterium permense* | 99.88% | 680433146a775ecf50a7181244f102c4 |
| Species | *Brevinema andersonii* | 100.00% | 028125a5ac4171cc52c7041b731fc923 |
| Species | *Brevinema andersonii* | 100.00% | fb89d1ee67a0fe79638add694bc978bc |
| Species | *Brevinema andersonii* | 100.00% | d3695dba62872e9cb46d606b2f64705e |
| Species | *Brevinema andersonii* | 100.00% | 3567e0635723d80876c625d4e4cb8582 |
| Species | *Brevinema andersonii* | 100.00% | cd063e317282ade29189f92afcb9812f |
| Species | *Brevinema andersonii* | 100.00% | e35067e0cd3d46e6cdd559af1f97152c |
| Species | *Brevinema andersonii* | 98.68% | b3d8d27684396e589160da187729bb37 |
| Species | *Brevinema andersonii* | 99.96% | db04ca9e3da6ce3d27bafbf4ee512a87 |
| Species | *Brevinema andersonii* | 100.00% | 99308a2891c70833f27cfd8cb0f06481 |
| Species | *Brevinema andersonii* | 100.00% | 509cfa06af67b0b220397485c73b3c1e |
| Species | *Brevinema andersonii* | 99.24% | a2e01813fa0a2f0bd4fb88a534fbb6c6 |
| Species | *Breznakia pachnodae* | 100.00% | 3a49e4a47360518e34bf3d06014437a8 |
| Species | *Breznakia pachnodae* | 100.00% | 11807357fb24e03c46bc9f7fa641f549 |
| Species | *Breznakia pachnodae* | 75.26% | 58e0ea359b54bd9722b07e1b392fc05b |
| Species | *Butyricicoccus pullicaecorum* | 93.28% | b6a199fc4940accebafdf70547a3bb83 |
| Species | *Butyricimonas paravirosa* | 91.39% | c8028f6f8135bebbeb366c9ea8bc31da |
| Species | *Butyricimonas paravirosa* | 78.93% | eeed28ec1bad5662026fa52320e246e0 |
| Species | *Butyricimonas paravirosa* | 77.91% | 27e42cf8fd7e5d86fd611b6d5c5ca568 |
| Species | *Butyricimonas paravirosa* | 91.48% | 6df04b3fa30d95a18ab134b0549f2ffc |
| Species | *Butyricimonas paravirosa* | 91.35% | a4f2a287187e7798c5d09924dedc3b46 |
| Species | *Butyricimonas synergistica* | 98.33% | 62d52470596024894b71430d29a16c49 |
| Species | *Butyricimonas synergistica* | 94.52% | b10fae8a1de5b33ee08d11e6fa6df50d |
| Species | *Butyricimonas synergistica* | 94.48% | 3c18a14e81d8a91a75439d7431f95d38 |
| Species | *Butyricimonas synergistica* | 87.95% | 8eec50607cebca0b95bb411bd09bc090 |
| Species | *Butyricimonas synergistica* | 98.32% | 9ce53d97ecb2cd6a102e6c336e86bf8b |
| Species | *Butyricimonas synergistica* | 92.99% | ee6b34f80536043330ba55b508d7aa1f |
| Species | *Butyricimonas synergistica* | 93.96% | b6986caf244d0b6f75300e9f2302f3ff |
| Species | *Butyricimonas synergistica* | 90.49% | f681d6e4f242af685c7063721cc6001a |
| Species | *Butyricimonas synergistica* | 77.26% | af7435e847ef8f8df1b306cc8a958094 |
| Species | *Butyricimonas synergistica* | 73.66% | 323c829f79cf3792cb4d1471ba28f92b |
| Species | *Butyricimonas synergistica* | 97.67% | 12adf9cf75e99230d71e710c96dc3a5d |
| Species | *Butyricimonas synergistica* | 98.33% | 40f1513f2f1c4647e819c88c5fea5368 |
| Species | *Butyricimonas synergistica* | 76.45% | c4188f057ea911a4b157321a42a76095 |
| Species | *Butyricimonas synergistica* | 94.93% | e1ed640d16a3cf178effa92231416f49 |
| Species | *Butyricimonas synergistica* | 90.39% | 54f0ab0189b9d1bb40115c3db1338902 |
| Species | *Butyricimonas synergistica* | 97.52% | c5927afb7a1d8ece46eebf772f29c498 |
| Species | *Butyricimonas synergistica* | 90.44% | dd857d0633bb7154882d54bf2f694702 |
| Species | *Butyricimonas synergistica* | 97.67% | ec65b0a09fdaae204417aef62da8c324 |
| Species | *Butyricimonas synergistica* | 98.33% | 448807270922c0c20fa2cbe564204003 |
| Species | *Butyricimonas synergistica* | 89.68% | 26612bba87485b9124602c1f3e26211c |
| Species | *Butyricimonas synergistica* | 73.46% | a23e82679a2275e56715fb163630dbc3 |
| Species | *Butyricimonas synergistica* | 90.04% | e1f1be94acb80bb4733471164acfa8dd |
| Species | *Butyricimonas synergistica* | 77.06% | 6fb7c140cdf08078aa7d93dfb8f60f2a |
| Species | *Butyricimonas synergistica* | 90.42% | 8b2c52916e730e2da9e4f6c234a7d7dc |
| Species | *Butyricimonas synergistica* | 93.95% | 02e4459afd7537685d9849a0ec46fe01 |
| Species | *Butyricimonas synergistica* | 93.94% | 4552c407118e17ec2bd47738bb919f9c |
| Species | *Butyricimonas synergistica* | 93.92% | cd37e6a5e9d5109d3564dc1a79f54d05 |
| Species | *Butyricimonas synergistica* | 88.80% | 0a37020e381f2026b1ad3f3d328c67ee |
| Species | *Butyricimonas synergistica* | 98.43% | df317e0246a6ec59ce4880c6baccf2e1 |
| Species | *Butyricimonas synergistica* | 90.69% | 1c6a6af652d602095488b26aa2d6af0f |
| Species | *Butyricimonas synergistica* | 97.39% | 4b5d340685821e929baa34cd58f52ada |
| Species | *Butyricimonas synergistica* | 98.33% | 74d72e466db1886fdcf1e7ca5048e8ad |
| Species | *Butyricimonas synergistica* | 94.47% | 38af27968bda60895558d3d942a2c6fc |
| Species | *Butyricimonas synergistica* | 98.01% | 81c6507717fb721b7d6749f7040eeb70 |
| Species | *Butyricimonas synergistica* | 84.90% | 53807f0c2d6e9d593b252af499eaebd3 |
| Species | *Butyricimonas synergistica* | 86.73% | 62e4f18a3503c275f42657c98956d5e2 |
| Species | *Butyricimonas synergistica* | 76.25% | 42efcc66bcf5f5f43d5eedd0a14b337f |
| Species | *Butyricimonas synergistica* | 88.79% | 1ca42dd9d0bd2e2e72a6fb283ed04ca1 |
| Species | *Butyricimonas synergistica* | 99.94% | d688a9cb240bf492a4809a8d50319586 |
| Species | *Butyricimonas synergistica* | 99.94% | a4502f9f5f6a38b454128239ee1e39bb |
| Species | *Butyricimonas synergistica* | 86.73% | b8f617859995f520f3874500f8ae4284 |
| Species | *Butyricimonas synergistica* | 89.36% | fb9bbd5cbfcd8c28c02244ea875bf844 |
| Species | *Butyricimonas synergistica* | 89.71% | 5ee6f4b2807d7d2538e10d49a6003497 |
| Species | *Butyricimonas synergistica* | 79.89% | 8c60aace91f2aa9e463672877f957fd8 |
| Species | *Butyricimonas synergistica* | 91.80% | d2615463c48de3f6b11b1c8d3d334287 |
| Species | *Butyricimonas synergistica* | 96.75% | 3b1d871ceb6d93336c1262d801f33a5c |
| Species | *Butyricimonas synergistica* | 88.22% | d691529ce16f16839d678d83575593c4 |
| Species | *Butyricimonas synergistica* | 87.57% | 37bc2809337b80f563702b814aad17a3 |
| Species | *Butyricimonas virosa* | 72.21% | 0adc9e9c8e6308aead2be913d2f6700b |
| Species | *Butyricimonas virosa* | 71.65% | 4ee412271006484a30c45904d2ee829b |
| Species | *Butyricimonas virosa* | 71.71% | 9129bbece4103f1eb32405476adf7fe8 |
| Species | *Candidatus Gastranaerophilales* | 98.72% | 808d38144eb238a61f2d662cec2db269 |
| Species | *Candidatus Gastranaerophilales* | 97.72% | 1ffef80edd559484a96b714a4c9aca6f |
| Species | *Candidatus Metachlamydia* | 99.80% | 98bd80a7a5cbc77c975bb329b9a9a878 |
| Species | *Candidatus Soleaferrea* | 99.59% | 9f9a1992609f49637f246bc25933a5d0 |
| Species | *Candidatus Stoquefichus* | 96.31% | f3c09792fc32e2a09c0533611f77cce5 |
| Species | *Candidatus Stoquefichus* | 85.29% | a74848957ba1c2ca982323886b7f02e9 |
| Species | *Carnobacterium sp.* | 99.93% | 64fb298eeaed7c003db2343449a11d0d |
| Species | *Carnobacterium sp.* | 75.50% | 5fe6b92dfa32f530a7fcd56a912ca0c2 |
| Species | *Carnobacterium sp.* | 80.75% | 87711015677b7dd6327ba3da9c388355 |
| Species | *Carnobacterium sp.* | 82.73% | f2939d2698196df8183f4f108080d763 |
| Species | *Carnobacterium sp.* | 81.22% | 5071cf38d570065bfa1d651f6b0aa2b1 |
| Species | *Carnobacterium sp.* | 83.57% | 9b9bb52bc7bae3ff458fcc5c5396cf7a |
| Species | *Carnobacterium sp.* | 71.52% | 0d7d66c6d7509536b4f5360ded178a75 |
| Species | *Castanea mollissima* | 100.00% | 90de2d49d5f5a6f4005b262bab093f25 |
| Species | *Catabacter hongkongensis* | 85.28% | 491e8238984ec4c7cfdf48afdd564e5a |
| Species | *Catabacter hongkongensis* | 86.14% | 54d1bb57ea98403a5b6168a790a09c5a |
| Species | *Catabacter hongkongensis* | 87.65% | 9b4d6219bbe42550b9f9064d37a525d5 |
| Species | *Catabacter hongkongensis* | 85.37% | a8bb06b2b4783dd4b0baf810b0aa8a18 |
| Species | *Catabacter hongkongensis* | 92.38% | e7fd65388ccb57794e51a624b21fdbfc |
| Species | *Cellulomonas cellasea* | 79.76% | 97f99887bf6d5a84de7a9180cd4c2e78 |
| Species | *Cellulosilyticum ruminicola* | 92.76% | 1f5a4161afe96eab483d5f02b736188f |
| Species | *Cetobacterium somerae* | 96.37% | c7a5d266a87052e6dac2d94c35fa2f2a |
| Species | *Cetobacterium somerae* | 96.37% | 5e00d23383a4be2c7d6cad8f01735e24 |
| Species | *Cetobacterium somerae* | 96.31% | f27680e31bd525d7a4597990f22c80cd |
| Species | *Cetobacterium somerae* | 96.37% | 726e59db2ced0c0c3160aa840f46a32f |
| Species | *Cetobacterium somerae* | 96.37% | cf20d85460aa880e73b0caabd6a0191e |
| Species | *Cetobacterium somerae* | 95.97% | 93b8ca3cfbd8f2534a894f9d81bd949a |
| Species | *Cetobacterium somerae* | 96.37% | 40f0586c7d573dc800949f53ed98745a |
| Species | *Cetobacterium somerae* | 95.78% | 82e4b5871b304f7ff2aec3a204b46f08 |
| Species | *Cetobacterium somerae* | 75.21% | c6a94d473c3a3c340798ef02fe55a009 |
| Species | *Cetobacterium somerae* | 96.50% | a5c900c0d35cb382155e3cf4600b4818 |
| Species | *Chlamydia muridarum* | 82.86% | b47281bf7295b6f8827f2c484b8667bc |
| Species | *Chlorella sp.* | 99.99% | 3c6689b4ed7b8af623b7144902990b36 |
| Species | *Christensenella sp.* | 96.36% | f5c18a9ae63737c3c079d0e510cb315b |
| Species | *Christensenella sp.* | 99.32% | 5d96313f6e92939eedad2b4fdab2a9fd |
| Species | *Christensenella sp.* | 99.30% | 816a903d9bec78bdc4d0952f85e7346e |
| Species | *Christensenella sp.* | 99.40% | 1cc397ecf83b6bf9be96f9a53276baa8 |
| Species | *Clostridiaceae bacterium* | 99.79% | 18c4ebb9ab16466cc644a47dd7a10699 |
| Species | *Clostridiaceae bacterium* | 99.79% | f49757f3c345984ce59a4f3ffb2c7d8b |
| Species | *Clostridiaceae bacterium* | 99.79% | 425a5f94394245c7dec69bae88f96c4a |
| Species | *Clostridiaceae bacterium* | 99.80% | 01b3929378544236a64184a8fd7bd41f |
| Species | *Clostridiaceae bacterium* | 99.80% | 56d05e86a8b5764aa4e01ddc1a544cd6 |
| Species | *Clostridiaceae bacterium* | 99.81% | 8ef8645aea9f6caeaeac41167e5730a6 |
| Species | *Clostridiaceae bacterium* | 99.81% | 4ec23508443a2a284c0bc19184688b8e |
| Species | *Clostridiaceae bacterium* | 99.81% | bd2c18388039400c8f1aaa0514a0b698 |
| Species | *Clostridiaceae bacterium* | 99.81% | ae30142237e4195ab215d3c876c108a8 |
| Species | *Clostridiaceae bacterium* | 84.26% | cef221b281f7fc996be3cae4752bbcdd |
| Species | *Clostridiaceae bacterium* | 84.47% | 5aac152e6a3db588784a37db6f6e5989 |
| Species | *Clostridiaceae bacterium* | 73.71% | e7c1313c4ea605a3577f35fa31aa29a8 |
| Species | *Clostridiaceae bacterium* | 96.29% | adb96de00911d65a0aae6beddea60438 |
| Species | *Clostridiaceae bacterium* | 81.23% | 90c193fe96701ec16e8829faa8f9c173 |
| Species | *Clostridiaceae bacterium* | 95.70% | ab343a0f25c06a125ac817db9ae04638 |
| Species | *Clostridiaceae bacterium* | 99.78% | 486195b5caa821eaaf1663254822509c |
| Species | *Clostridiaceae bacterium* | 99.45% | 1beb34c0ef1c5729bef908f20fb19681 |
| Species | *Clostridiaceae bacterium* | 99.09% | 0ef62fcf857c4f039178b17054f7e12f |
| Species | *Clostridiaceae bacterium* | 99.79% | cd282761c0f13bb4456b20779a9fe07c |
| Species | *Clostridiaceae bacterium* | 99.79% | ccd1a0bbce89a45903a4e12f683693c4 |
| Species | *Clostridioides difficile* | 99.98% | 191fb9b810f1bfe274626fcd46033e95 |
| Species | *Clostridioides difficile* | 99.97% | 75bfd1bc9d8a951096cb54aa5a89f182 |
| Species | *Clostridioides difficile* | 99.98% | 17309c08d5300e4cb88a5e2b74b8a302 |
| Species | *Clostridioides difficile* | 99.98% | 351a3724979e012283576b40efc38a22 |
| Species | *Clostridioides difficile* | 99.97% | 67334550622e218205a2460e084ffb6d |
| Species | *Clostridioides difficile* | 99.97% | 9c8eefdde75f147eaa1f8b9f2b00333c |
| Species | *Clostridioides difficile* | 99.93% | c17b0caec8b2dd477cafbc4fea22ae94 |
| Species | *Clostridioides difficile* | 99.96% | eb95266f0da830eaf3d84e051772d64a |
| Species | *Clostridioides difficile* | 99.97% | 83305d90f1b24b113008373aea69e90e |
| Species | *Clostridioides difficile* | 99.34% | e0ae5f033feb3a4dc15a636de272cbd8 |
| Species | *Clostridioides difficile* | 99.36% | f2e767e4cafb1b73564ee8b88a9e8bcd |
| Species | *Clostridium argentinense* | 93.55% | 1694fbaa3139a4b572838c6f64accd57 |
| Species | *Clostridium baratii* | 96.62% | ad4774b72d741e6dde6839f14020f5fa |
| Species | *Clostridium baratii* | 91.43% | 44dfb612d459214c564bbd5aa666ca7a |
| Species | *Clostridium gasigenes* | 85.57% | bf6c21d955bcab2ae3ab23698b76ed04 |
| Species | *Clostridium paraputrificum* | 99.80% | f92aa4e3fdcbd638c207be7c44f8c186 |
| Species | *Clostridium paraputrificum* | 88.12% | fbdb06120dfc03ea36b48a86b4e139c7 |
| Species | *Clostridium paraputrificum* | 89.47% | d79fffd630364149263248c1ea46dede |
| Species | *Clostridium paraputrificum* | 86.72% | 3a36637ee072ce1a460507d17014a2f2 |
| Species | *Clostridium paraputrificum* | 99.82% | 283f7186f61d25b59e7e188156cd89bb |
| Species | *Clostridium paraputrificum* | 99.85% | 35344da3a76f3cbe1911d6e04be4be41 |
| Species | *Clostridium paraputrificum* | 99.67% | 1c8932a26af6ca5329e23944fed56a58 |
| Species | *Clostridium paraputrificum* | 94.94% | c9b4d8e79313f3e60cd59b98bf07b607 |
| Species | *Clostridium sartagoforme* | 74.37% | 5bf02fed08db12b912ed0825d8d8ee3c |
| Species | *Clostridium sartagoforme* | 74.36% | 97ccd0310cb05fc18fd45cde9e3aaac1 |
| Species | *Clostridium sartagoforme* | 74.39% | d8f1c331b9d594724d4f96cfe5e4d675 |
| Species | *Clostridium sartagoforme* | 74.47% | dafdc1cbfccca3e97ecf88324cc6239b |
| Species | *Clostridium septicum* | 99.16% | 646c827ab5a7fc656dac273cc7ddd677 |
| Species | *Clostridium septicum* | 94.22% | 20b6ae7152b0447a0dd4c52f6c624b41 |
| Species | *Clostridium sp.* | 98.75% | f2be06fe9f5009b9b0de9ad4a177ad0e |
| Species | *Clostridium sp.* | 99.24% | 217d9ab8ea35d3a58f1fe203b2dfb76f |
| Species | *Clostridium sporogenes* | 99.97% | b44f3c52eba25ea4b23d8ea5a9dd3609 |
| Species | *Clostridium tarantellae* | 70.54% | 15cdaaf1072200a0e64794655c0d047e |
| Species | *Collinsella aerofaciens* | 92.02% | 36fe92a14e3125660e240eda85df570d |
| Species | *Commensalibacter sp.* | 94.43% | 0a04b0bf86dad60a2f48f4ddf153cbe2 |
| Species | *Coprobacter secundus* | 91.95% | 400e9867e0c310dfcfec2c022ff7f8c8 |
| Species | *Coprococcus comes* | 98.38% | b23efd97a13eab6eb4354c41ed1ca980 |
| Species | *Coprococcus comes* | 99.42% | 78d4ef4dee6555d9669b29d8fbcd3cab |
| Species | *Coprococcus comes* | 98.35% | c09904a544b1f6490114a43860929bbf |
| Species | *Coprococcus comes* | 99.30% | 8529e618e8125bc04fc90a4787ca376d |
| Species | *Coprococcus comes* | 99.32% | f82f305444f43fbe312a229a4c11f7d0 |
| Species | *Coptotermes gestroi* | 87.62% | 92d8d3a1cd080b9c0424fba481163c73 |
| Species | *Coptotermes gestroi* | 89.99% | 6e9a451998b04d6108fca72bd743f83e |
| Species | *Coptotermes gestroi* | 82.61% | 7e31d2fb30ca079608fd95762ababf65 |
| Species | *Coptotermes gestroi* | 88.70% | 6710f8a022b69b3658d2df6a05ebd05b |
| Species | *Coptotermes gestroi* | 84.26% | aa8bdff4a012bf0961cf88f4c7de2645 |
| Species | *Coptotermes gestroi* | 81.43% | 6172a2d2f8be01e3bf43a517afbee6a6 |
| Species | *Craurococcus sp.* | 99.59% | 54c9e1a64ca8fe5e3205d3ebcaa9e36d |
| Species | *Cronobacter sp.* | 97.65% | f289c3774400d1ed57aa7a5fc032e35b |
| Species | *Daucus carota* | 99.89% | 3aa0058dde5f254d5fd6f9d78d8dca58 |
| Species | *Daucus carota* | 99.99% | 11b2bb2ab1d06df29a95bffe89ef0641 |
| Species | *Daucus carota* | 99.99% | a7a612209b5e93757d0d398b391291a9 |
| Species | *Deinococcus planocerae* | 99.27% | 38aff6662d3e5e9bc7c27a6990a618e3 |
| Species | *Deinococcus planocerae* | 99.20% | 771d2a2cbcd100edc3e34e5c86bca395 |
| Species | *Deinococcus planocerae* | 99.22% | c2e84fe53eb68a518bd4464943deef4f |
| Species | *Deinococcus planocerae* | 93.93% | 4874fcda72a8ea3c12495c03b59dac17 |
| Species | *Deinococcus planocerae* | 99.13% | ed504b219ba49be8bb861d9d2f8d6a6e |
| Species | *Desulfovibrio cuneatus* | 78.58% | f60aa88956221490740b5c42b7e9b5e8 |
| Species | *Desulfovibrio sp.* | 92.44% | 7140c2c5a0aeb0f498758876b6013115 |
| Species | *Dielma fastidiosa* | 90.06% | a0bab0f85bbe0a83a0ca58be2144810d |
| Species | *Dielma fastidiosa* | 90.12% | b313977d9fa339fe02bd8154a63af146 |
| Species | *Diplorickettsia massiliensis* | 99.28% | e2921f281b9e22533e9e7fabaa8a633a |
| Species | *Diplorickettsia massiliensis* | 99.37% | dd8de825caab29db7c6af23aa700f664 |
| Species | *Dysgonomonas alginatilytica* | 83.67% | a5622c101b0cf6dade2e7f94ff290794 |
| Species | *Dysgonomonas alginatilytica* | 85.54% | 9ecbc32dcbb0c3d2f79e626c3c3c11f1 |
| Species | *Dysgonomonas alginatilytica* | 99.15% | a554242d96622bcbc263ae77ab2dba16 |
| Species | *Dysgonomonas alginatilytica* | 99.82% | c54ddceea163c2029a6c4bf846795485 |
| Species | *Dysgonomonas alginatilytica* | 86.68% | 109d8a6690f1655458b7f956bb39aed8 |
| Species | *Dysgonomonas alginatilytica* | 99.91% | 2f082c81fd6c843c6e181c6c1e9cc748 |
| Species | *Dysgonomonas alginatilytica* | 93.95% | ac4befb65bda3b8beda37bb4db919f81 |
| Species | *Dysgonomonas alginatilytica* | 99.06% | 15bdedc7e75f54bfb3207f16c2a22578 |
| Species | *Dysgonomonas alginatilytica* | 99.82% | 1c44646af5b6feb4bcbf3d96e277708b |
| Species | *Dysgonomonas alginatilytica* | 99.95% | c3fc80bcd0300b01d95fd9ac7ca78a10 |
| Species | *Dysgonomonas alginatilytica* | 73.66% | 259724c51267302a8772e8666c9ba9d1 |
| Species | *Dysgonomonas macrotermitis* | 82.12% | 8d7216dd67f5ec8ef5d01a3fcdaeb42c |
| Species | *Dysgonomonas macrotermitis* | 81.84% | f18a7e0cb692458a50b577fbc19d2a97 |
| Species | *Dysgonomonas macrotermitis* | 70.81% | 6207364583a8df25230b39edeb789b12 |
| Species | *Dysgonomonas mossii* | 86.89% | 157e3986ff9f2e82ca5bf9d3d1df42e2 |
| Species | *Dysgonomonas termitidis* | 88.32% | 2945da0ececdb46013d424d1da420ff8 |
| Species | *Eggerthella sinensis* | 90.42% | ad2ef24967ae18ddeb6f547b80089c5f |
| Species | *Eggerthella sinensis* | 90.15% | c0a2adca8248504931808268373c85e7 |
| Species | *Eggerthella sinensis* | 89.88% | eb517df3326c6380eb561948dc52dcd8 |
| Species | *Eggerthella sinensis* | 89.96% | ceb8ef82b8c5d50fcb539588c0b4aeb1 |
| Species | *Eggerthella sinensis* | 91.02% | 5d638d67c0fae8a233d15cc83fa8c89c |
| Species | *Eggerthella sinensis* | 90.24% | 9877ba16ae9b4a609ac3e67fa894c688 |
| Species | *Eggerthella sinensis* | 90.77% | 48d1c623391e3d28f20f777af874acc7 |
| Species | *Eggerthella sinensis* | 90.51% | 76fff711067dfa361d1039e129b58edc |
| Species | *Eggerthella sinensis* | 90.42% | a2ae4fda15e1ed4dcfdd2bc07969aa41 |
| Species | *Eggerthella sinensis* | 83.87% | a7813883a80e53805b2605bad0af95d3 |
| Species | *Eggerthella sinensis* | 88.81% | 0b52d61079f2eb201be9e809a69c31e5 |
| Species | *Eggerthella sinensis* | 88.51% | 8a773be60af8c63491615c65ffb80e7e |
| Species | *Enterococcus durans* | 80.07% | fc988c71f30957939dd4d2a54a7d589b |
| Species | *Enterococcus durans* | 77.85% | bdd3b9e40add57bc2666cee57a12abb2 |
| Species | *Enterococcus durans* | 75.81% | 73aa8e92a00adc6c3a2e0cf327e36903 |
| Species | *Enterococcus flavescens* | 71.30% | 639e42bd767844461f16e2b45a7edc80 |
| Species | *Enterococcus flavescens* | 71.28% | 7d0dc903f3733ea789a3bf4f096b4ba0 |
| Species | *Enterococcus flavescens* | 71.30% | 956bdaba3f7a317220e481dfd6427ce9 |
| Species | *Enterococcus flavescens* | 71.26% | cf9520407b512f7176c5c915beb57cc4 |
| Species | *Enterococcus flavescens* | 70.44% | 25759ac53904e4cf2a2a01d79066b5d4 |
| Species | *Enterococcus flavescens* | 96.23% | bb89c071ef90eda8c22c637ee6188d6c |
| Species | *Enterococcus flavescens* | 96.24% | f761c5c1418ac7dfad775a906b053de3 |
| Species | *Enterococcus flavescens* | 71.69% | 7129aa96be7fd020fb3618f539b9652c |
| Species | *Enterococcus flavescens* | 96.28% | 63e493d6f78e2757ad889f18bd8862a8 |
| Species | *Enterococcus flavescens* | 96.26% | 229a164df9920047fc370ad71b53f892 |
| Species | *Enterococcus flavescens* | 71.61% | de658bed4810d2bf6fd8e584ba25a775 |
| Species | *Enterococcus flavescens* | 73.28% | 3363c35d4e375908b235ae2bf17faaa3 |
| Species | *Enterococcus flavescens* | 71.00% | dcd1db3d56ea584b8e362528e9e7da61 |
| Species | *Enterococcus flavescens* | 96.28% | 08a9f6dd30cf976193917ba1c372c69d |
| Species | *Enterococcus flavescens* | 96.24% | c35084b2ef74f14e59b44146b8402123 |
| Species | *Enterococcus flavescens* | 96.24% | a2e46f566ff73361079ba719d5954051 |
| Species | *Enterococcus flavescens* | 71.32% | 1047eedcf413bacda11fee505cdd2b12 |
| Species | *Enterococcus flavescens* | 96.16% | 94aa4cfcadb8cce57e6640e9c3d0be9e |
| Species | *Enterococcus flavescens* | 71.22% | 3157b3131e434adefcd6753acacae5ac |
| Species | *Enterococcus flavescens* | 70.07% | 074914261c05fe9ce2789b48167bd41d |
| Species | *Enterococcus flavescens* | 70.31% | ad67ba7c8cd471c3adc1ed837e4cd999 |
| Species | *Enterococcus flavescens* | 71.22% | c00dce1d2edaef471f843224ebffd54c |
| Species | *Enterococcus flavescens* | 73.30% | f06ac570edbbedbf76898aca131758e9 |
| Species | *Enterococcus flavescens* | 70.96% | 79d2631f2ad92737101d68a5a6845077 |
| Species | *Enterococcus flavescens* | 71.00% | e1e1f467562c6f072f9b190564621d8f |
| Species | *Enterococcus flavescens* | 70.75% | 660df5670dc7a076c0a1b5d14220e4ac |
| Species | *Enterococcus flavescens* | 71.65% | 68f185bb04eaa0634aa741c6078a4545 |
| Species | *Enterococcus flavescens* | 71.00% | 20f88b684f1e286f049d634f3d27b48d |
| Species | *Enterococcus flavescens* | 72.54% | 05934e659ccfe4c1515b92ca9214cbcd |
| Species | *Enterococcus flavescens* | 95.81% | f2e3db20132f73a6054b78a8b3b975be |
| Species | *Enterococcus flavescens* | 96.28% | 6d7b9b56de7bb5d052f8e4bd603bc83e |
| Species | *Enterococcus flavescens* | 71.33% | b7d9356f1c2055200224bc3549ea7d89 |
| Species | *Enterococcus flavescens* | 71.01% | fd0d0300587831a7a45f934377ade984 |
| Species | *Enterococcus flavescens* | 73.32% | 8a5f716815ea032cd5028ef3f7d33440 |
| Species | *Enterococcus flavescens* | 96.68% | 9367aa8f2f237b70ba8a14874f4d78b9 |
| Species | *Enterococcus flavescens* | 96.50% | db443d6aaec44f3b79b97db29bb3880f |
| Species | *Enterococcus flavescens* | 95.70% | 29d3ea62e3e7fd849f8aec71ae5e9cfc |
| Species | *Enterococcus flavescens* | 71.31% | b537990a033a712ea401b6685687e250 |
| Species | *Enterococcus flavescens* | 71.31% | 57d4d44095d42583f4d1bbc662f69ac2 |
| Species | *Enterococcus flavescens* | 70.21% | 6c4cba47f11593735e6cf2cdd99087a4 |
| Species | *Enterococcus flavescens* | 70.98% | 580446c59e77c758be8bc4f501b922df |
| Species | *Enterococcus flavescens* | 72.48% | 74c2a9635d02a6237d73618ea211f19a |
| Species | *Enterococcus flavescens* | 74.02% | 1512d0878dfb9f949a8a106f19a0057a |
| Species | *Enterococcus flavescens* | 96.90% | 212f4bdbe5e0f373706cec3c57dde51b |
| Species | *Enterococcus flavescens* | 70.87% | 5ff39d72e7032c9be3ce40cac2f1b2ff |
| Species | *Erysipelatoclostridium ramosum* | 92.76% | 3098f9a8e182cdd4fd0544cf2262e37c |
| Species | *Erysipelatoclostridium ramosum* | 90.11% | 5203bec7245324e7a13918f9709d806b |
| Species | *Erysipelatoclostridium ramosum* | 90.27% | 618c45cdfe119e9caa860fb38a773eb1 |
| Species | *Erysipelatoclostridium ramosum* | 91.06% | 7e39a3a5804e6883ee532d13b9897167 |
| Species | *Erysipelatoclostridium ramosum* | 92.76% | 922e0dc08e366058ffae66c961387fef |
| Species | *Erysipelatoclostridium ramosum* | 99.49% | f16b872d319394421a6d4078f013704d |
| Species | *Erysipelatoclostridium ramosum* | 92.76% | fbedc3bb7d4234c584b3269e49921db1 |
| Species | *Erysipelatoclostridium ramosum* | 90.23% | b9e1df59b5b8c467b97635e14accc34f |
| Species | *Erysipelatoclostridium ramosum* | 91.05% | b8cd8066e32848174203d7455b09f272 |
| Species | *Erysipelatoclostridium ramosum* | 92.76% | b27ecac6169e402ee16aa64a044f3cc5 |
| Species | *Erysipelatoclostridium ramosum* | 79.38% | 609c895e034fdf87f0ec3e5ee3b5940d |
| Species | *Erysipelatoclostridium ramosum* | 92.76% | 625d1bbce8e0aa846b1a4614a09a9d59 |
| Species | *Erysipelatoclostridium ramosum* | 88.43% | 62297720e59e3c9d995b9e072750c36b |
| Species | *Erysipelatoclostridium ramosum* | 79.48% | 68838897a48020274651cd159f3e502a |
| Species | *Erysipelatoclostridium ramosum* | 92.76% | 3540dcdef6bd4a1831d896088038df4d |
| Species | *Erysipelatoclostridium ramosum* | 92.75% | f0232052c8e4b4ccf86cd53393243586 |
| Species | *Erysipelatoclostridium ramosum* | 79.47% | 0ae951c5bb9edca4c0e384ae91059ed9 |
| Species | *Erysipelatoclostridium ramosum* | 92.72% | 930ec23632c4ab72d62cb5311f317f3d |
| Species | *Erysipelatoclostridium ramosum* | 91.01% | 17f862f27bf2ab2fd608df9c86799a0b |
| Species | *Erysipelatoclostridium ramosum* | 88.93% | 8dafb28ba92b03a30a3f30453c7abfe7 |
| Species | *Erysipelatoclostridium ramosum* | 79.44% | 5703f39aa1ff4f12a1e452f472e99b58 |
| Species | *Erysipelatoclostridium ramosum* | 73.51% | 82753d9413ae60bcd7e21bff38ad92ca |
| Species | *Erysipelatoclostridium ramosum* | 93.90% | caf3a891960c0f7e7c7fa3770d9d81e1 |
| Species | *Erysipelatoclostridium ramosum* | 74.66% | 27f4e1e5ddb2181e8e1c4fb1294fec37 |
| Species | *Erysipelatoclostridium ramosum* | 79.69% | 1f5fea9fd0fb7e97e618e3a395bb619f |
| Species | *Erysipelatoclostridium ramosum* | 92.76% | f2866a47c70fd9a7175bdded7b9d3f29 |
| Species | *Erysipelatoclostridium ramosum* | 79.44% | 52153aa10acefc70c27f8c675f8ad486 |
| Species | *Erysipelatoclostridium ramosum* | 88.43% | 95b9083b607413f4a1324ece1c174c7c |
| Species | *Erysipelatoclostridium ramosum* | 91.72% | b9bc6be40f15ec1b7e95fb0ff529ee32 |
| Species | *Erysipelatoclostridium ramosum* | 90.19% | 8a1d53b7223388cfa611d2298b8e6fff |
| Species | *Erysipelatoclostridium ramosum* | 81.42% | c35a54608f92a7c07072dee6353d0193 |
| Species | *Erysipelatoclostridium ramosum* | 79.71% | d7256285def51e95317af143781e0a4b |
| Species | *Erysipelatoclostridium ramosum* | 90.16% | e2c0c972d0b5a6f0f7686a0c305cc169 |
| Species | *Erysipelatoclostridium ramosum* | 90.04% | 979d45faf816182d9b03a8ef219da1af |
| Species | *Erysipelatoclostridium ramosum* | 90.15% | 939da1b52e670d3c16d0ccdbe8a74628 |
| Species | *Erysipelatoclostridium ramosum* | 81.58% | 02301b40f2ac15f42f3e849c61be0925 |
| Species | *Erysipelatoclostridium ramosum* | 79.61% | feda1ebdbab5b9acc6a11bb7ccba368a |
| Species | *Erysipelatoclostridium ramosum* | 92.27% | 358160d4b4c21395fd1a01d4ca25828a |
| Species | *Erysipelatoclostridium ramosum* | 90.09% | 275e14577cd43957df62f39cb7a3d6c8 |
| Species | *Erysipelatoclostridium ramosum* | 90.15% | 1efb42ee5f0dc0e537aa73c241cb6225 |
| Species | *Erysipelatoclostridium ramosum* | 76.92% | 94526dd2f3e029315ad17a3235faca28 |
| Species | *Erysipelatoclostridium ramosum* | 93.96% | d8e882541c52aa7992bb5766217850e4 |
| Species | *Erysipelatoclostridium ramosum* | 82.25% | 4eca3903a3b75ee78c1e1594c7d65c8f |
| Species | *Erysipelatoclostridium ramosum* | 91.03% | 426db1f48d030cf3a90538ff554c299d |
| Species | *Erysipelatoclostridium ramosum* | 90.18% | b766c2a1ac0d80810992eadb5aa925fe |
| Species | *Erysipelatoclostridium ramosum* | 92.78% | f86f8b5acf29f6ca152413cd0b90e9bf |
| Species | *Erysipelatoclostridium ramosum* | 91.35% | b3df24d518ad2b91582971c285566f7d |
| Species | *Erysipelatoclostridium ramosum* | 96.95% | f9b74a630a77c5149856a5ae28ff0091 |
| Species | *Erysipelatoclostridium ramosum* | 96.34% | d09079a0cccc31ce19fb5ed41d08caa6 |
| Species | *Erysipelatoclostridium ramosum* | 95.63% | 88c2ea61b569edc4551bbbdaa2680628 |
| Species | *Erysipelatoclostridium ramosum* | 94.81% | b3be052be5116d949908e0f0a4758c8b |
| Species | *Erysipelatoclostridium ramosum* | 93.45% | a45a4f64cab7bd4b7d0127e42ff38487 |
| Species | *Erysipelotrichaceae bacterium* | 99.83% | c355bb94ecd02482fbd4f2163fb89466 |
| Species | *Erysipelotrichaceae bacterium* | 97.46% | 1b27ddb823ce871e7f1992033ba0cbca |
| Species | *Erysipelotrichaceae bacterium* | 99.55% | 0ff1fb53901f21145063eba26b00ff03 |
| Species | *Erysipelotrichaceae bacterium* | 89.00% | ca9143e71f33cefb44c0dc138f58bdea |
| Species | *Erysipelotrichaceae bacterium* | 95.43% | 9fc00ac6164f826bcb6a3a79da2466b1 |
| Species | *Erysipelotrichaceae bacterium* | 95.80% | 05f009f67ff5f435f6c8574e9c989590 |
| Species | *Erysipelotrichaceae bacterium* | 99.75% | 4343d2a6db172f55a8122c70e15879e4 |
| Species | *Erysipelotrichaceae bacterium* | 99.81% | 70a4180a03a616e593fe90289b8b93a0 |
| Species | *Erysipelotrichaceae bacterium* | 99.56% | 67d4f1d97b22b4db0d49e0ac6d8d1da5 |
| Species | *Erysipelotrichaceae bacterium* | 95.49% | ef3e71a4cd40d01121c81e562790c421 |
| Species | *Erysipelotrichaceae bacterium* | 94.52% | 7610f1bcd2388a76b3f4bf272406dc96 |
| Species | *Erysipelotrichaceae bacterium* | 98.56% | 62f069be9e75c20b8c815a7fa80323ee |
| Species | *Erysipelotrichaceae bacterium* | 96.98% | a339b8a47fc8f6572fa70fff635718e3 |
| Species | *Erysipelotrichaceae bacterium* | 97.93% | 25a411bba0f93fdfc411e80fa697faec |
| Species | *Erysipelotrichaceae bacterium* | 97.46% | 8d37cbee274569b15b5b29588d4d5327 |
| Species | *Erysipelotrichaceae bacterium* | 82.70% | 7152ed2cf417a262ca78bbae4236554d |
| Species | *Erysipelotrichaceae bacterium* | 99.83% | 85a552f3a3cc74674ef145a45bd3e12a |
| Species | *Erysipelotrichaceae bacterium* | 94.28% | ae789bae1c4152a25b7c4e2ab5ad31b6 |
| Species | *Erysipelotrichaceae bacterium* | 88.46% | ab1193598594c2eea380f2acbdef3140 |
| Species | *Erysipelotrichaceae bacterium* | 87.19% | 059b0848b100ef5e92c8765da1bcf8f7 |
| Species | *Erysipelotrichaceae bacterium* | 80.00% | 3d26638214cdde13bdc1d1fbfe547a86 |
| Species | *Erysipelotrichaceae bacterium* | 91.50% | fcf871a3494d0e3382147ac365f8a82c |
| Species | *Eubacterium callanderi* | 80.66% | 9ca02a963344915250836307dae27948 |
| Species | *Eubacterium callanderi* | 80.66% | 4ea6e725cc6ab4e79781a58062eac0dd |
| Species | *Eubacterium callanderi* | 79.14% | 24798abc0d5446824c77968b03c156ce |
| Species | *Eubacterium callanderi* | 75.39% | 790c9e4a176a07751d7f4ea60cc8a3f8 |
| Species | *Eubacterium callanderi* | 79.14% | ca4082fcda43023e17c86f42187a3941 |
| Species | *Eubacterium callanderi* | 77.61% | 57076d9d8fb2962b22827dee2d9ed3e6 |
| Species | *Eubacterium callanderi* | 80.28% | d6e8eae0eed202b6fa878fd585319267 |
| Species | *Eubacterium callanderi* | 80.07% | 98af733eb56bdac143e4c35f99a65947 |
| Species | *Eubacterium callanderi* | 97.14% | 433ea36c987a95881d190e43eadf0bd9 |
| Species | *Eubacterium callanderi* | 96.73% | 7184b38d244bf8d177b47f82c3d7b563 |
| Species | *Eubacterium callanderi* | 83.87% | 5bf5b0eadb52f70386c7ca5cfd42a2d0 |
| Species | *Fusobacterium equinum* | 89.67% | 419524a11ad2b9f2cd215994070f718f |
| Species | *Fusobacterium varium* | 92.12% | ac11dfec5cca5fafb394a542d2182a2c |
| Species | *Fusobacterium varium* | 92.32% | c1a629f3db2ce8e98ff7383741b15e7d |
| Species | *Fusobacterium varium* | 92.08% | 000a5bf0a2338b2c2c0a37773729db5f |
| Species | *Fusobacterium varium* | 92.32% | f76982fa5f6bcd4bde7370d31ffa80ce |
| Species | *Fusobacterium varium* | 92.95% | 2048f3d03b85e5aa9dfeb486351d4128 |
| Species | *Fusobacterium varium* | 92.32% | 55456b61e313ba0da52c3b5c5748e608 |
| Species | *Fusobacterium varium* | 91.97% | 2031e88f1c34dc6245fcf9d8df46ca62 |
| Species | *Fusobacterium varium* | 91.63% | 8bd0c083ddd4dd2757e6b8ab2c801c7f |
| Species | *Gabonibacter massiliensis* | 75.55% | fc86f693c8680838441170519ce37a45 |
| Species | *Gabonibacter massiliensis* | 75.39% | 142db24a76825d32e8d13b76ca5fe443 |
| Species | *Gabonibacter massiliensis* | 75.13% | f77668eb42490db033b36b53cc6fd31e |
| Species | *Gabonibacter massiliensis* | 75.19% | 5a30fba29b97fa84239f7d006beef747 |
| Species | *Gabonibacter massiliensis* | 75.61% | d576208009a6dd4e4e9ee98c1948a224 |
| Species | *Gabonibacter massiliensis* | 75.61% | bb9da0cc5e464242bc9710531d63b927 |
| Species | *Gabonibacter massiliensis* | 75.25% | ea161321618bb1d20972e7c715a22fac |
| Species | *Gabonibacter massiliensis* | 75.61% | 61d2728616ee189ac5dd30c0ac752bd2 |
| Species | *Gabonibacter massiliensis* | 75.39% | 289e0ec1451e22ddfca0eb87d20224eb |
| Species | *Gabonibacter massiliensis* | 75.45% | a4315a7cbd80873e5a8c0e413e7c6e29 |
| Species | *Gabonibacter massiliensis* | 75.39% | 89cf3020c474b667859fc4b6687fdf51 |
| Species | *Gordonibacter faecihominis* | 95.53% | 2eda10724555d2dd52bcddaeb73789f5 |
| Species | *Gordonibacter faecihominis* | 95.53% | 5930995fb30954e4390babd920256461 |
| Species | *Gordonibacter faecihominis* | 95.55% | 6932ded2f776ab3f29959cfec7f7124c |
| Species | *Gryllus firmus* | 96.72% | 782ef141862611a4c1142d7e07780ae3 |
| Species | *Gryllus firmus* | 96.77% | 483ab1e6dceef710bd7ba6370f6191ff |
| Species | *Gryllus firmus* | 99.67% | eb80eb2512e6fda4973d0454a65e9a9e |
| Species | *Gryllus firmus* | 99.63% | 760e823b2dd7742177d85f3c5f776c24 |
| Species | *Gryllus firmus* | 99.73% | 732da470f44bc2f038bbcaa5ed3637d2 |
| Species | *Gryllus firmus* | 99.45% | 3c53fec8e4da30fdc2a1e94235e249a7 |
| Species | *Gryllus firmus* | 99.77% | c6ca7aa813281894202125b73d1683f3 |
| Species | *Gryllus firmus* | 99.95% | bc7b2e0081722d92eec9f87d2d5dc93f |
| Species | *Gryllus firmus* | 99.96% | aa933aee8463cfc4f138a245894eabbc |
| Species | *Gryllus firmus* | 99.95% | e62d19c3c5870b45f81fe23496a66b1b |
| Species | *Gryllus firmus* | 99.95% | 0d1b06d7f18b5717abd6961155d64d04 |
| Species | *Gryllus firmus* | 81.37% | 4f0a02eb021997b926bb5aa38c3d23ba |
| Species | *Gryllus firmus* | 98.51% | 65dfbfe214cddccfffe863212d28c489 |
| Species | *Gryllus firmus* | 99.65% | e6de2f34e0271b7522569c4e37f08c5f |
| Species | *Gryllus firmus* | 99.74% | 5f393534512d6d45053ad4ffba304126 |
| Species | *Gryllus firmus* | 99.98% | e3edc1c8760fc9bd99fa70835347e09c |
| Species | *Gryllus firmus* | 99.55% | 0999203ab21bb7a1ee2bf37a3ea0216f |
| Species | *Gryllus firmus* | 99.51% | db73bb86140eebdcb34227e3d02f8862 |
| Species | *Gryllus firmus* | 84.55% | 7db72089b75cbc3e973345dff35ca42c |
| Species | *Heliconius numata* | 99.50% | 7f61aa69ee04f2735186b382b9904257 |
| Species | *Holdemanella biformis* | 99.90% | 602ed1a26b43dfe64a7c2370e5c0f787 |
| Species | *Holdemanella biformis* | 99.88% | dec941dfc4287631d0b51ed1bd0cd979 |
| Species | *Holdemania massiliensis* | 97.74% | 0280ee8e9ec1ba53d2f68be03e55aafa |
| Species | *Holdemania massiliensis* | 72.99% | ed5385e1b01fcffe9ab85df13ae2b5cd |
| Species | *Holdemania massiliensis* | 96.03% | 0077699bfaa196e8f5bf57cfb776fc3b |
| Species | *Holdemania massiliensis* | 98.22% | f54e5d20a136edbbe58ed143af34799f |
| Species | *Holdemania massiliensis* | 97.90% | 2e9663799bf82dfa84a607a129f4667c |
| Species | *Holdemania massiliensis* | 96.05% | 4571fabd7f8ff9f9e4edb561a9a7df78 |
| Species | *Holdemania massiliensis* | 76.95% | 43587200429b3640567c41c0a877811c |
| Species | *Holdemania massiliensis* | 96.04% | 0b58e5d85f9b7723bde5e9284fb8e0e7 |
| Species | *Holdemania massiliensis* | 97.04% | 072cfe3bd703b0e0343ac9ee1e66980f |
| Species | *Holdemania massiliensis* | 99.53% | e94bedb398b08d866e149d701367c56c |
| Species | *Holdemania massiliensis* | 78.73% | 0ce9f393538639dc6f1c35bdb3e7ecbb |
| Species | *Holdemania massiliensis* | 75.57% | 69ff15a96763d6f798c9a6247011a52c |
| Species | *Holdemania massiliensis* | 76.92% | 6973ff7a9ae1e3074d3ec262d4ec7195 |
| Species | *Holdemania massiliensis* | 96.35% | 8d6215fe56ddac1778d96b1875d8f491 |
| Species | *Hungatella effluvii* | 71.78% | f4490f4502517bd8fc144d13b33054c2 |
| Species | *Hungatella effluvii* | 72.72% | 52a9984820de3e9abb7ba92dfc11a184 |
| Species | *Hydra vulgaris* | 99.96% | 41e257115e3d48dc78719d7ad79695da |
| Species | *Hydrogenoanaerobacterium saccharovorans* | 90.35% | 5d764bae41e4ebae749426159b2a62a7 |
| Species | *Hymenobacter rivuli* | 98.63% | ff4c09fda173641a6697227e279bb258 |
| Species | *Hymenobacter rivuli* | 88.90% | bc8695fa8f0b601033e1d437442c4e05 |
| Species | *Intestinimonas butyriciproducens* | 84.84% | a95b93615daac46cf946a573e361645f |
| Species | *Intestinimonas butyriciproducens* | 72.37% | 48dddebc8fab6e63c06a6b0b4144f296 |
| Species | *Intestinimonas butyriciproducens* | 73.33% | 01685b34e36f0aaa99e03f3ef5650f29 |
| Species | *Intestinimonas butyriciproducens* | 86.03% | 1c8d35efb8c5498c12647454dd39c455 |
| Species | *Intestinimonas butyriciproducens* | 73.43% | 4c83d368dbc073e59d5b49012a1b868a |
| Species | *Intestinimonas butyriciproducens* | 74.66% | 0a06eb5f13960c9a3946b6fa50b056f5 |
| Species | *Intestinimonas butyriciproducens* | 73.17% | b26344b0992b434d88836db70248fe68 |
| Species | *Intestinimonas massiliensis* | 73.30% | 9a3d7bb7ab7e3587a0e37fad259b076f |
| Species | *Intestinimonas massiliensis* | 72.45% | c6c59256cc99ceaca6c56cd7ffb406a5 |
| Species | *Intestinimonas massiliensis* | 72.18% | 34a68c727e19c56182c5dfe1df3d2ad2 |
| Species | *Intestinimonas massiliensis* | 70.89% | ffdf54177fe108602bf2122db1f74e8e |
| Species | *Intestinimonas sp.* | 79.36% | a9cf657300669521577973c0ad34a379 |
| Species | *Intestinimonas sp.* | 83.90% | 9fbd9c894cb74e5d481bd2ddbd41b523 |
| Species | *Intestinimonas sp.* | 82.36% | be54b4878e0399b718ff5364d9a587d0 |
| Species | *Intestinimonas sp.* | 84.87% | 72c640f4af1ef7f82915d8206b598b7d |
| Species | *Lachnoclostridium sp.* | 84.07% | ea8cf8e55badb17b4e09b5bd8a58f430 |
| Species | *Lachnoclostridium sp.* | 83.36% | 83cc0146f4278c0467e417f0faf6fd88 |
| Species | *Lachnoclostridium sp.* | 90.36% | 45697465a1c004dfc0fd6e22a7ec0546 |
| Species | *Lachnospiraceae bacterium* | 74.85% | 2398efeed86ee546ce0a3d9025eeb4ef |
| Species | *Lachnospiraceae bacterium* | 95.72% | 74710ababf180b63a2562f1731785352 |
| Species | *Lachnospiraceae bacterium* | 100.00% | 5c9a8799436e4ff0c7329c405f0f6127 |
| Species | *Lachnospiraceae bacterium* | 95.70% | 5a66ecd6da42731329510bfaf7312c6f |
| Species | *Lachnospiraceae bacterium* | 97.37% | 9b1414221b3b4a7b0748c5ae133e2603 |
| Species | *Lachnospiraceae bacterium* | 96.33% | 953db7e2a9a423a7088673eb0d90e8b9 |
| Species | *Lachnospiraceae bacterium* | 99.93% | 62c3b06f5ca3835a418c154c667969d6 |
| Species | *Lachnospiraceae bacterium* | 90.10% | 88585c61070724a2a14f2c71e7a4251d |
| Species | *Lachnospiraceae bacterium* | 96.10% | 4a94c986a239608b96b5bcdfbee7a447 |
| Species | *Lachnospiraceae bacterium* | 96.78% | f0ca902992809c0373219675b36d467d |
| Species | *Lachnospiraceae bacterium* | 97.93% | edf46924b8148a8ecff2bc3a408ea9c4 |
| Species | *Lachnospiraceae bacterium* | 96.89% | 0380fd1a772ad76cb58a7c8cb4d87031 |
| Species | *Lachnospiraceae bacterium* | 99.93% | 81be6691e61546391720f36b5938e7b6 |
| Species | *Lachnospiraceae bacterium* | 99.19% | 3f221177f2a71a7f580d13e839b0f2d1 |
| Species | *Lactobacillus apodemi* | 92.47% | f64c5afccfd20a847b1ea6ef19c5ebb3 |
| Species | *Lactobacillus floricola* | 94.06% | 0d649083179c94085c89c4ade21c7c3f |
| Species | *Lactobacillus ozensis* | 91.75% | fa9df1a3834345c0b8ae839c0d38c4eb |
| Species | *Lactobacillus panis* | 84.93% | d250ad9a6057dbc6dae13d462c976e58 |
| Species | *Lactobacillus rossiae* | 83.68% | 0c0ec4fa4177e8f8c723f0de85c37a78 |
| Species | *Lactococcus formosensis* | 96.85% | dfa7ecdb2909d69e6d34782d7f74afa1 |
| Species | *Lactococcus formosensis* | 96.84% | 3eaef149edc4ab1f568e60ee26246c5a |
| Species | *Lactococcus formosensis* | 96.85% | f5e2bfad09b7c5d794df0aa3eeb3d743 |
| Species | *Lactococcus formosensis* | 96.53% | dd9b4e2bfc8ca41a7be24717946b8385 |
| Species | *Lactococcus formosensis* | 91.44% | 359666d5ebd94bc7994afec6c86aa1d9 |
| Species | *Lactococcus formosensis* | 98.36% | 7b2b3f9b43265f7dc2192dcf6d988839 |
| Species | *Lactococcus formosensis* | 89.62% | b77642db32da204b90dd2eeefd47120a |
| Species | *Lawsonia intracellularis* | 100.00% | 5d7021798df8236389f9e0b9d1e2311e |
| Species | *Leucobacter populi* | 98.61% | fd2ba65e214077697aa0dc37c9a83ad1 |
| Species | *Longibaculum muris* | 90.28% | 38b0c7a0461f513500af2700b9420e4b |
| Species | *Longibaculum muris* | 93.46% | 3b686a69b177232615e6712c967a2fec |
| Species | *Longibaculum muris* | 90.98% | ba73cb80d06046d161fdc677a9612809 |
| Species | *Longicatena caecimuris* | 81.17% | da7e3a453064eea150ee0f69ab4710b2 |
| Species | *Luteimicrobium xylanilyticum* | 81.04% | c43af1562a50a4fd84f876aad6bc9d2e |
| Species | *Luteimicrobium xylanilyticum* | 80.09% | 15890ee80a728103a3c6bc03fd6b654a |
| Species | *Methylobacterium adhaesivum* | 99.26% | 08f5ed0ff5d2c34c031a1feef93fbffc |
| Species | *Methylobacterium aerolatum* | 99.32% | 3645da011da25d809be39d4f0d028544 |
| Species | *Methylobacterium cerastii* | 81.05% | 8af9b75618653e55bdfc98110826c8d5 |
| Species | *Methylobacterium goesingense* | 95.94% | eafc58857f1d762ec68c5cfc2fb47b73 |
| Species | *Methylobacterium jeotgali* | 95.41% | 9d6778fcdbcf5ab374af178af67deb53 |
| Species | *Methylobacterium sp.* | 75.08% | 2e6c7a93b39d750782f3a8be42bde2be |
| Species | *Monocercomonoides sp.* | 91.64% | 726c4bddfba5054bde10cf484546188b |
| Species | *Monocercomonoides sp.* | 91.52% | 3c22ec93c15480788cc36633c388ae3d |
| Species | *Monocercomonoides sp.* | 91.52% | 86a912ac9fa8a0813423176266d06173 |
| Species | *Monocercomonoides sp.* | 90.08% | b9a6432252e40c90b170a601ce49b75d |
| Species | *Monocercomonoides sp.* | 90.08% | a2cb85d6be8eead9009d4c2a1cc2e0c3 |
| Species | *Monocercomonoides sp.* | 91.64% | 5f35f89da664c727dcdd0684a6783430 |
| Species | *Monoglobus pectinilyticus* | 78.42% | 193493fc91775252c297f5924e69e610 |
| Species | *Monoglobus pectinilyticus* | 78.72% | df349efe3287aa52926af4b69b50ec37 |
| Species | *Monoglobus pectinilyticus* | 82.97% | 8ff090944e197cda2670369a4114bb14 |
| Species | *Monoglobus pectinilyticus* | 82.75% | 19afa0393f6b9a071ad9a6762d9bb32b |
| Species | *Monoglobus pectinilyticus* | 82.81% | 8aedbc8c67eb9f0327fdfd40dcf5a17d |
| Species | *Monoglobus pectinilyticus* | 72.76% | 9358b20385e6085483aba7f37478544f |
| Species | *Monoglobus pectinilyticus* | 76.73% | 2f270d56892564ab9ffd7876e97ec088 |
| Species | *Monoglobus pectinilyticus* | 73.98% | 86eb328bf5e4ab4aee056ef84b3f26a8 |
| Species | *Monoglobus pectinilyticus* | 82.24% | 96c13091e1602463fd20a221172f60a7 |
| Species | *Mycoplasma iguanae* | 87.37% | 3d32523b2a9adfeb255c807fcdde7f1c |
| Species | *Mycoplasma penetrans* | 86.85% | 7c618e5f6eb0cf8c7df9e573495d08b8 |
| Species | *Mycoplasma penetrans* | 84.72% | 68a7442e7448105c76875c46e1ea25e8 |
| Species | *Nocardioides halotolerans* | 98.19% | ec16328d0c77168fb2f1b0eb5956e9ed |
| Species | *Nocardioides halotolerans* | 98.10% | c93207ac2d13a179d9c8fb0f796144ef |
| Species | *Nocardioides kandeliae* | 97.74% | 499e9a3d94fbeb7ea3cdf7c3be4bf69f |
| Species | *Odoribacter laneus* | 99.29% | a0b5c077dc8d97b9fe62199c2c6b13ba |
| Species | *Odoribacter laneus* | 99.29% | 2c4ded08e07bbc875004d86f777d7669 |
| Species | *Odoribacter laneus* | 99.08% | 9a46fc754a692f904543122476fef89a |
| Species | *Odoribacter laneus* | 99.24% | c51771361cc0d5110d17303bf367793a |
| Species | *Odoribacter laneus* | 99.06% | c5d0e70973214c5df921696bc73a3603 |
| Species | *Odoribacter sp.* | 99.81% | d8b3e5dd8412f8a442d2330fd262afde |
| Species | *Odoribacter sp.* | 99.84% | 8d6f42272782c04d18cabc67b4affcf2 |
| Species | *Odoribacter splanchnicus* | 93.47% | cc5dcf21555c7bdabc7b1fd3d454177c |
| Species | *Odoribacter splanchnicus* | 93.60% | 5ca49d21143ef23dfa5f0321aa7d6514 |
| Species | *Odoribacter splanchnicus* | 93.60% | 8ec9e58c15ef80f34d822329cbbf927f |
| Species | *Odoribacter splanchnicus* | 92.45% | de668defa0a319667d2ff83c25262895 |
| Species | *Odoribacter splanchnicus* | 94.29% | 9e410b129846947ca7735381b3df0599 |
| Species | *Odoribacter splanchnicus* | 92.45% | de3d9f662401e40fdc22c195675c017d |
| Species | *Odoribacter splanchnicus* | 92.81% | 4e09112f1cb6c0a7fa24268e33a92d59 |
| Species | *Odoribacter splanchnicus* | 92.99% | ddf72421683d68a75562d31b802fc3b1 |
| Species | *Odoribacter splanchnicus* | 86.98% | 9d8e7f8811b661305457b0b8508335b0 |
| Species | *Odoribacter splanchnicus* | 91.03% | a9481892dd545aa27d665c9eb469847d |
| Species | *Odoribacter splanchnicus* | 93.50% | 2d85f41f1c27d04f8ab7624a9bc0aa7a |
| Species | *Odoribacter splanchnicus* | 90.32% | 0951170cce8781736ddef2ba2c3b34d4 |
| Species | *Odoribacter splanchnicus* | 79.99% | 23bc32cb61d45de61028a5f1b48751fc |
| Species | *Odoribacter splanchnicus* | 94.11% | 9827b5372d4afaebd2e01174fad956ff |
| Species | *Odoribacter splanchnicus* | 94.05% | a73d0d3c40607a5495b3d7afcf44edb9 |
| Species | *Odoribacter splanchnicus* | 93.82% | a788450c203ff69ad2b67b886cfb0cd3 |
| Species | *Odoribacter splanchnicus* | 93.30% | 9fd615c9cc8d091c1fbebc198866c444 |
| Species | *Odoribacter splanchnicus* | 82.88% | 54c0d1d17590b41aff4733b02a9e7fd3 |
| Species | *Odoribacter splanchnicus* | 97.25% | ffef671711ff53e8daf956981d3726ef |
| Species | *Odoribacter splanchnicus* | 94.30% | 1ac1ae45d65040ebe62b6f2cc990fc89 |
| Species | *Odoribacter splanchnicus* | 90.55% | c1799c0febd59229998736f76f0b4e04 |
| Species | *Odoribacter splanchnicus* | 82.34% | 13eb52407812882c20a3c64eab118a20 |
| Species | *Odoribacter splanchnicus* | 71.29% | 9b9d62fcd93b36b44d8d89b07523c4a6 |
| Species | *Odoribacter splanchnicus* | 91.03% | 44fa39fdfd538ee0038d506bee626f2c |
| Species | *Odoribacter splanchnicus* | 86.56% | 1dac56970e69486deb1a6dee412b7ef9 |
| Species | *Odoribacter splanchnicus* | 82.88% | 9f060b9455c420f833d07d5404b69d8c |
| Species | *Odoribacter splanchnicus* | 93.49% | af05301a8366fc3c4349cfe5549c0554 |
| Species | *Odoribacter splanchnicus* | 97.25% | e2ae0c0fa7dc20c06289a5e4850c39cc |
| Species | *Odoribacter splanchnicus* | 96.09% | ec3337edc01e199dff5dfd70a82d8037 |
| Species | *Odoribacter splanchnicus* | 71.77% | a17e6d6aa7fade9677e571fc86cb319e |
| Species | *Odoribacter splanchnicus* | 91.89% | cbc27257cd00c814a23dd785df4cf8b5 |
| Species | *Odoribacter splanchnicus* | 91.43% | 3fab7c47443156862a14c2e00185fa4a |
| Species | *Odoribacter splanchnicus* | 94.75% | 85646029378d0c7ded60178e1f71be78 |
| Species | *Odoribacter splanchnicus* | 80.03% | 4e0fc1724a848e0cebae910873300cd4 |
| Species | *Odoribacter splanchnicus* | 90.60% | 8715a2d3670e64aa55e73d13df560b1a |
| Species | *Odoribacter splanchnicus* | 93.29% | 184b2caaf4b8e12b4b7dd42867107f0e |
| Species | *Odoribacter splanchnicus* | 93.52% | 7dc3fcc817f0a24e8126fc1422928f4e |
| Species | *Odoribacter splanchnicus* | 94.18% | 2ec6bb87ddf837ce7e3b03c4f9995e28 |
| Species | *Odoribacter splanchnicus* | 70.98% | df8068ffbafa2abdfeca32f3755394c8 |
| Species | *Odoribacter splanchnicus* | 73.27% | ed19f8da475e2dcd00cda91c9437c15c |
| Species | *Odoribacter splanchnicus* | 93.74% | 53433841791fc627c1f403b03237c856 |
| Species | *Odoribacter splanchnicus* | 83.44% | 038c60058ee0bc77b6f7e57f14d7ebfe |
| Species | *Odoribacter splanchnicus* | 97.25% | 11a1d79caf01be00dd7030771b3a113f |
| Species | *Odoribacter splanchnicus* | 81.42% | 6e7375aba20a0a1957df826e2cbf49d0 |
| Species | *Odoribacter splanchnicus* | 94.01% | 22a6349e1d0cef8d56c19d1fc6a8f955 |
| Species | *Odoribacter splanchnicus* | 85.66% | 5b009a37e9a7897ddc4c021ea52e1e1b |
| Species | *Odoribacter splanchnicus* | 78.88% | dc73d92b1ae50f19d2cae3844884aac2 |
| Species | *Odoribacter splanchnicus* | 94.26% | fbe567723cdce61c1c938bbff4f6a6d7 |
| Species | *Odoribacter splanchnicus* | 86.07% | 952ad024fada9ac92488ff784a96db8e |
| Species | *Odoribacter splanchnicus* | 86.56% | c9f3eeab233d4f128493fed67611a688 |
| Species | *Odoribacter splanchnicus* | 80.37% | cf441fe3bae2464ed12609528d791fa2 |
| Species | *Odoribacter splanchnicus* | 96.25% | ef02d5c40ec7eeb8a0d78301af76a246 |
| Species | *Odoribacter splanchnicus* | 94.28% | fb7bf768a7dc2db0e43b3aa80d0e5e39 |
| Species | *Odoribacter splanchnicus* | 71.81% | f1b9ab3ce4ac9735745b203049acd89c |
| Species | *Odoribacter splanchnicus* | 93.34% | 89bd719e631b95ab2cd10d45d40d782c |
| Species | *Odoribacter splanchnicus* | 94.28% | 7621989e2b1d828294475fa0fbc5c864 |
| Species | *Odoribacter splanchnicus* | 93.22% | 9f9d13030f33fc07255aa0f39ed07107 |
| Species | *Odoribacter splanchnicus* | 71.18% | e901c2ef1319519c76b6ccf84f2a9820 |
| Species | *Odoribacter splanchnicus* | 84.22% | bc0c836daf57a8ca208e393d5536c9b3 |
| Species | *Odoribacter splanchnicus* | 93.52% | 9c2cde448b0530a12cefa9d9f423a0cd |
| Species | *Odoribacter splanchnicus* | 98.65% | 85e84dca1d47d7403565895526c0c62d |
| Species | *Odoribacter splanchnicus* | 73.30% | b713d49cee2d264e56b5572ae08865e5 |
| Species | *Odoribacter splanchnicus* | 78.94% | 3cc6a9b00ef98d7ec2d36cc3edba6ff0 |
| Species | *Odoribacter splanchnicus* | 92.12% | 5926efa63b37cfd6c6bee573b258be1e |
| Species | *Odoribacter splanchnicus* | 72.02% | 80ddc1e3b5cb647d18a68a5a5235a45b |
| Species | *Odoribacter splanchnicus* | 89.06% | df4fbb2230ca31602f0536221cc2f87d |
| Species | *Odoribacter splanchnicus* | 73.32% | c738183b6fe3b99ce17003edca22cba8 |
| Species | *Odoribacter splanchnicus* | 94.07% | a8a9d49dfb421724c8faf65f0e61246e |
| Species | *Odoribacter splanchnicus* | 83.84% | 7cd9d8600df094c8e50efcc1505bf5eb |
| Species | *Odoribacter splanchnicus* | 88.40% | bbbbf5b75fc4b80ef15e9bd7f2612c2a |
| Species | *Odoribacter splanchnicus* | 89.95% | 16b2add3b587571f5c82ba7597b4d1b5 |
| Species | *Odoribacter splanchnicus* | 93.45% | cd61870d51f493b4eca0d86eb0db248b |
| Species | *Odoribacter splanchnicus* | 94.59% | 3b7b8a57a4325d1d3a592d7e0a4c1e69 |
| Species | *Odoribacter splanchnicus* | 86.52% | 1774170e745bb07b2749f83edbdf28e7 |
| Species | *Odoribacter splanchnicus* | 74.84% | 60d82804ef4bb582ee72f2c4b6403538 |
| Species | *Odoribacter splanchnicus* | 95.85% | 268a29b87f947be4466f7f6069e6d922 |
| Species | *Odoribacter splanchnicus* | 78.79% | e7b46fec7b58f7f17e8f3b9149d05f45 |
| Species | *Odoribacter splanchnicus* | 94.69% | 98f39cff6c6856fd1fa648a8be3d0290 |
| Species | *Odoribacter splanchnicus* | 70.57% | 2cc4a58117a11d508b32b8bbb7d377ba |
| Species | *Odoribacter splanchnicus* | 92.33% | 30b1ab7c0c09eec4e3105b274fc4304c |
| Species | *Odoribacter splanchnicus* | 93.27% | 2417f0d0802749566f7fddafb90b3e91 |
| Species | *Odoribacter splanchnicus* | 74.48% | 806fa5c5538c37468fe801ea064c1856 |
| Species | *Oerskovia turbata* | 92.57% | 9a5463ebdbba08440db851b085f43c25 |
| Species | *Oerskovia turbata* | 95.01% | ecbe33ae716019ef3d213e265838589c |
| Species | *Oerskovia turbata* | 78.49% | 91062e7fc372451f8774b956988ab9c6 |
| Species | *Orbus sasakiae* | 90.14% | 995c4679139a170b1f525546d56a411c |
| Species | *Oscillibacter sp.* | 73.02% | 7336ea38f59fd02664c948ccccacfc48 |
| Species | *Oscillibacter sp.* | 70.96% | f1172b72e1005e5c4ad0fa696b92c3dd |
| Species | *Oscillibacter valericigenes* | 80.62% | b9b0c67480666b32d32f4df04870cb6c |
| Species | *Oscillibacter valericigenes* | 82.41% | 9552c05318444cfb227a900422839922 |
| Species | *Oxalobacter vibrioformis* | 91.41% | 6761778f0cf0d4b4181dd7639ac286d7 |
| Species | *Oxalobacter vibrioformis* | 90.20% | 5b55aae7c292ded1d578ad7fde37ca74 |
| Species | *Paenibacillus antibioticophila* | 90.84% | b917bb904126a8611618e150c1986a7b |
| Species | *Paenibacillus doosanensis* | 76.72% | c6229c50156f9449f6cbbff39329eb40 |
| Species | *Paenibacillus marinisediminis* | 93.77% | 2657ed250e164bc97522a72a169ceca8 |
| Species | *Paenibacillus marinisediminis* | 99.49% | bc3e364e36baa04e18edf219c74fd08a |
| Species | *Paenibacillus marinisediminis* | 91.77% | 37dcad17ebccb105c27ed0d4489b201d |
| Species | *Paenibacillus purispatii* | 98.70% | f545ea867a4232c970bec19190176aa4 |
| Species | *Paenibacillus purispatii* | 91.87% | 1819b590c6823e00295c1db0523eb8ae |
| Species | *Paenibacillus purispatii* | 98.65% | 7162521771fc5692abb9a64319627c7e |
| Species | *Paenibacillus purispatii* | 98.67% | e25941ba00d858e532800a3e9223fc77 |
| Species | *Parabacteroides distasonis* | 85.86% | 8eaca96d6b94d1139e6b73673ade3800 |
| Species | *Parabacteroides distasonis* | 85.86% | aaadde7eb97ea9aee016a2c36a3184fa |
| Species | *Parabacteroides distasonis* | 80.77% | 4efeac63739f568c89967423bebf014f |
| Species | *Parabacteroides distasonis* | 86.63% | 3a2081cd304355f6187121e0835107ad |
| Species | *Parabacteroides goldsteinii* | 89.24% | b32b1c436383303d6136bb9e77ca3490 |
| Species | *Parabacteroides goldsteinii* | 89.25% | fd986a851fc5a0ba21127ca57e5ad00d |
| Species | *Parabacteroides goldsteinii* | 89.24% | e1839e3849a16d50d837400c2079c526 |
| Species | *Parabacteroides goldsteinii* | 89.25% | fa09927c5886fc468fb7f97f270fcd63 |
| Species | *Parabacteroides goldsteinii* | 89.24% | fe8cbb3d2f18c917e5ee1756fc56064a |
| Species | *Parabacteroides goldsteinii* | 98.95% | e8c197d2d747433e74fe05c0fbdaef6c |
| Species | *Parabacteroides goldsteinii* | 74.43% | 2a8f926304ad304a7ec90862af55727a |
| Species | *Parabacteroides goldsteinii* | 74.41% | f3f789335a7cc6c560a73be9633cf41c |
| Species | *Parabacteroides goldsteinii* | 71.31% | 32d04f2069462348d5d02d5220133c0f |
| Species | *Parabacteroides goldsteinii* | 98.29% | 16332f7135a94b51f5ee870f363151b7 |
| Species | *Parabacteroides goldsteinii* | 76.72% | 15c40eec040e41ff3486e262769ca6dd |
| Species | *Parabacteroides goldsteinii* | 89.24% | 9c75c289cc9f4f8fab937ae71c267db3 |
| Species | *Parabacteroides goldsteinii* | 71.11% | a1c7c3b95cb418b7f055693f98073878 |
| Species | *Parabacteroides goldsteinii* | 91.05% | 7ee6badb1a021f43bc9b94e59b12f3fa |
| Species | *Parabacteroides goldsteinii* | 98.28% | 46ae90ef4332094f5814df17914867b5 |
| Species | *Parabacteroides goldsteinii* | 91.42% | 08e5c1652d0802b5d02bf7b5b268bc4c |
| Species | *Parabacteroides goldsteinii* | 88.31% | dafb239d4b9ce17eee84777552401e1a |
| Species | *Parabacteroides goldsteinii* | 87.38% | 4bd7b09e05c4a3e279e4443be79da87c |
| Species | *Parabacteroides goldsteinii* | 75.84% | 7d306290140a7cd4303f51d2a7363093 |
| Species | *Parabacteroides goldsteinii* | 89.33% | 81fc87e8d297bfd2cdf2f99a773f0c96 |
| Species | *Parabacteroides goldsteinii* | 76.16% | 7b9dc8ee0b3cb301541af008e1f2e33e |
| Species | *Parabacteroides goldsteinii* | 89.13% | 7e0606b8b2863b13d247c4d4e4612a88 |
| Species | *Parabacteroides goldsteinii* | 98.95% | 819ca59944f89dd7ce856e12228acc5d |
| Species | *Parabacteroides goldsteinii* | 75.56% | 9ac3462ac71246f794be5c187b7bd4fd |
| Species | *Parabacteroides goldsteinii* | 71.98% | 225381ddf6880c311c7be2422b68f70e |
| Species | *Parabacteroides goldsteinii* | 74.85% | 9fb6d4ad3262349b00db6010c9e71cdf |
| Species | *Parabacteroides goldsteinii* | 89.24% | 79c95c6b195b2f979b8b6a009ea6dce6 |
| Species | *Parabacteroides goldsteinii* | 87.69% | d9cdd3585a6d16bc60aa69a136238270 |
| Species | *Parabacteroides goldsteinii* | 82.58% | c746754b01270a9016e523dcff4f0e56 |
| Species | *Parabacteroides goldsteinii* | 80.45% | 1768e282d1b9597d3f49f98c0f744875 |
| Species | *Parabacteroides goldsteinii* | 98.95% | 4d52a8fdf5f967e7682fa9c3ea0a5902 |
| Species | *Parabacteroides goldsteinii* | 84.00% | 18d8d5fb26f18c23d47f7cdb948b7834 |
| Species | *Parabacteroides goldsteinii* | 75.12% | 95021bf4e9715444e779b39fcf271271 |
| Species | *Parabacteroides goldsteinii* | 73.99% | 8ea11fd64556a161ea2ddb4eda0ae5f9 |
| Species | *Parabacteroides goldsteinii* | 82.76% | 036f04d19827c1c5a4a789a33863fff0 |
| Species | *Parabacteroides goldsteinii* | 73.09% | ef15c6e9f09491d42743761b72b22643 |
| Species | *Parabacteroides goldsteinii* | 98.91% | dfc59e870ba823c84a335d593d733bac |
| Species | *Parabacteroides goldsteinii* | 89.09% | 1af0434a7479ecbe59cf31c860d581a2 |
| Species | *Parabacteroides goldsteinii* | 74.19% | 022e2d67340612301076d95826c4ed72 |
| Species | *Parabacteroides goldsteinii* | 98.91% | a1dd5902f617dec55a8fa0eb0daea87e |
| Species | *Parabacteroides goldsteinii* | 73.78% | 36f278c0a886d66aee72b7f48338c1c0 |
| Species | *Parabacteroides goldsteinii* | 83.06% | d460443eb438e3c84fee282e718268d8 |
| Species | *Parabacteroides goldsteinii* | 80.45% | 27d68fecac0886c036f4a9c02c53c096 |
| Species | *Parabacteroides goldsteinii* | 81.24% | 944b6af93783da5161249ddaffc0bdef |
| Species | *Parabacteroides goldsteinii* | 83.47% | 9fbc2cc5dea66919af293a03e4b2cc92 |
| Species | *Parabacteroides goldsteinii* | 96.36% | a31cd20cd68adf3ea7e2a46b0af75831 |
| Species | *Parabacteroides goldsteinii* | 99.11% | 87fdb0b52c3ecf76227860df33436680 |
| Species | *Parabacteroides goldsteinii* | 90.78% | 6f19c2c7895c35600a324839e2715574 |
| Species | *Parabacteroides goldsteinii* | 90.78% | f57e76525cd1ddc5cd8118d8d823eef6 |
| Species | *Parabacteroides goldsteinii* | 95.58% | e7640f5af3acdc8f21ca95349ca4604f |
| Species | *Parabacteroides goldsteinii* | 87.81% | f28ff9a144a703537953a140a7961861 |
| Species | *Parabacteroides goldsteinii* | 90.69% | 204dd3185422578c856592ffa8cc4615 |
| Species | *Parabacteroides goldsteinii* | 98.70% | ab12abdfa69b3b67beeca7096221d016 |
| Species | *Parabacteroides goldsteinii* | 95.05% | 54756a7c94bdd5b6495aef94c6a8dd02 |
| Species | *Parabacteroides goldsteinii* | 90.68% | 960b4234d9604b51ba4eb59e891b24f1 |
| Species | *Parabacteroides goldsteinii* | 96.12% | 8bbf928d88002bb0bc808a0a6642eb99 |
| Species | *Parabacteroides gordonii* | 98.22% | e092f6361521068ddfa71a3845f229e2 |
| Species | *Parabacteroides gordonii* | 76.90% | 1368508bb2221ee74b051f4ac1d9ff3e |
| Species | *Parabacteroides gordonii* | 76.90% | 1e0db838c1c9607d237debf71cd2816c |
| Species | *Parabacteroides merdae* | 72.44% | a6b8f8eb89f38226184b10fa8baad67e |
| Species | *Parabacteroides merdae* | 74.70% | b8c4d43208e84c171c4a3f65a8269e80 |
| Species | *Parabacteroides merdae* | 75.85% | d28687c22e1f25ba0bf232e8d364288a |
| Species | *Parabacteroides merdae* | 72.44% | 70f135028ac986cc1911feef5f088a5f |
| Species | *Parabacteroides merdae* | 75.68% | 1064107489d25aac26c88e6c32a1e9c8 |
| Species | *Parabacteroides merdae* | 71.90% | 8e4e71c1f799b21e1cd574faca915ec9 |
| Species | *Parabacteroides merdae* | 71.90% | 3fd56dc1277390697703fead7b91bc14 |
| Species | *Parabacteroides merdae* | 72.44% | 41d390639a3813dcdfc00e764ffcc667 |
| Species | *Parabacteroides merdae* | 74.18% | dfd54d562975ec52b029f313cb01ec56 |
| Species | *Parabacteroides merdae* | 74.18% | e63d185ac6cf92192edf389f76321e87 |
| Species | *Parabacteroides merdae* | 71.32% | c0db0fa37ccceb213a01f7fefd512abb |
| Species | *Parabacteroides merdae* | 74.17% | a3169c59977a56515370fc113a6cbb79 |
| Species | *Parabacteroides merdae* | 75.54% | 19d16790162372c3c548730905364e75 |
| Species | *Parabacteroides merdae* | 71.33% | 4a95e57f23019de008df6b1f8dcc73c1 |
| Species | *Parabacteroides merdae* | 71.40% | 536bcebaaae7c4561ef55d0d102184ba |
| Species | *Parabacteroides merdae* | 76.38% | f245a49b387bc8e53f2bbe14758332b0 |
| Species | *Parabacteroides merdae* | 71.81% | 860aaf153932d2bcae60712e7e1f4386 |
| Species | *Parabacteroides merdae* | 71.80% | ea79f70f3aae8b5e2b2ba5c4a09acdef |
| Species | *Parabacteroides merdae* | 71.81% | 3b3dffe761bcb244a5c08e61d8ce8c48 |
| Species | *Parabacteroides merdae* | 71.86% | 2c99ceac6b72e89ae4b8070e224a94c6 |
| Species | *Parabacteroides sp.* | 98.09% | 2328ed27e6eacd15ea3d4955b194cf61 |
| Species | *Parabacteroides sp.* | 98.18% | f25c0a0b39c177f0852633c2de238728 |
| Species | *Parabacteroides sp.* | 98.96% | 0ba15fb3366f493a55808e7291827ea6 |
| Species | *Parabacteroides sp.* | 99.20% | f3c935c0e388b61f2f875456af8acfc4 |
| Species | *Parabacteroides sp.* | 98.51% | 51c4c42f12ee13fc4a47637b68166316 |
| Species | *Paracoccus marcusii* | 72.71% | ca51821302bd3bf27cef1f6287f1c893 |
| Species | *Paraeggerthella hongkongensis* | 95.85% | 6e9bea550986d173db1a36239fd1b2fe |
| Species | *Paraeggerthella hongkongensis* | 95.68% | 03663f2e98411faf2c05c60dcb2ff764 |
| Species | *Paraeggerthella hongkongensis* | 95.93% | d23fff0212b956734807ea59c68695b2 |
| Species | *Paraeggerthella hongkongensis* | 95.59% | b2f9cbcebc9a823241c8b3dd4920bb9c |
| Species | *Paraeggerthella hongkongensis* | 95.08% | 7c802651895539599d6692eb5a301b36 |
| Species | *Phascolarctobacterium faecium* | 99.58% | 5445a8a5aab7e5d5d89dbc5b78ec3a75 |
| Species | *Phascolarctobacterium faecium* | 99.61% | 7a94619f25176cf4931b6d000308160b |
| Species | *Porphyromonas canoris* | 99.96% | f283d82803079f724dd3abd680b52509 |
| Species | *Prasiolopsis sp.* | 99.92% | 59210bfbe5ce1611290ab1ef14016960 |
| Species | *Prasiolopsis sp.* | 99.99% | cd7d6563eefd837586ad468e4d6180bc |
| Species | *Prasiolopsis sp.* | 99.94% | 98eba5201a37be3450db76d86c6e9e91 |
| Species | *Pseudoclavibacter soli* | 90.01% | 4dc89e6fb6aae935662bbf5329bd94bd |
| Species | *Pseudoclavibacter soli* | 78.31% | ca2a3b05edbc5fc2ba1bc1f803e829d9 |
| Species | *Pseudoclavibacter soli* | 72.14% | 04a13ab682d940a9347fd09c5dfcef67 |
| Species | *Pseudoflavonifractor sp.* | 72.87% | 32739321d8a08cacd2d422fc904611d5 |
| Species | *Pseudoflavonifractor sp.* | 84.07% | 10636325e8808c1e7d06873aeec9ae50 |
| Species | *Pseudoflavonifractor sp.* | 85.42% | 7d48c1d1917d0300c759813ea9bd86d3 |
| Species | *Pseudoxanthomonas taiwanensis* | 98.25% | 96bbd871b8484fdbce8634bad7cb4493 |
| Species | *Raoultibacter massiliensis* | 95.03% | 3a528f39fc2dbcd754519b405244951d |
| Species | *Raoultibacter massiliensis* | 95.01% | aef79d9c9580297030e0bc71680fc507 |
| Species | *Raoultibacter massiliensis* | 95.03% | 048689321c244c678bbc02b70037ea05 |
| Species | *Raoultibacter massiliensis* | 90.81% | 0f0eade9e2723df13ee974d59f40bdd5 |
| Species | *Raoultibacter massiliensis* | 95.03% | 8f999cebb64a867880f549853c112455 |
| Species | *Raoultibacter massiliensis* | 77.08% | 1a6fd77f1fa8b1a22bd3203483558699 |
| Species | *Raoultibacter massiliensis* | 95.05% | 935d5a45bf48113f2cc24acf18c7ad6e |
| Species | *Raoultibacter massiliensis* | 94.01% | 2120f9f2f23f284fed36ad349804bcc3 |
| Species | *Raoultibacter massiliensis* | 90.12% | d9d9e8bf27ed638d3a29faf54e8716c5 |
| Species | *Raoultibacter massiliensis* | 93.41% | 01925031a5fce335af337017c9764870 |
| Species | *Raoultibacter massiliensis* | 90.07% | a830e677d6c98894fb7de27a08f84961 |
| Species | *Raoultibacter massiliensis* | 95.05% | 919f71f1e756fd084f9e18c9fc70b233 |
| Species | *Raoultibacter timonensis* | 91.77% | 9a9fd5ae09c97ca5f85f6fd544d60ca8 |
| Species | *Rikenella microfusus* | 96.59% | 49696ffc617b43e02023da034e92b66d |
| Species | *Rikenella microfusus* | 71.09% | 480ed4632ffdbf0e34406bb741401677 |
| Species | *Rikenella microfusus* | 74.91% | 0faa64047675feeb07ab754e864040e9 |
| Species | *Rikenella microfusus* | 70.92% | af4db378d4ec8b89d8c02eb1b79410e2 |
| Species | *Rikenella microfusus* | 96.07% | b7f6ecef38a70c97bb55992c9f761f91 |
| Species | *Rikenella microfusus* | 96.07% | fe1c5d4f2147230403712cda3290d882 |
| Species | *Rikenella microfusus* | 97.58% | 73a28f26c8284884f40a2623f055aa25 |
| Species | *Rikenella microfusus* | 90.19% | 7e66c026e25e2aae501c7ddb094a5746 |
| Species | *Rikenella microfusus* | 91.86% | 3f97c6a3988171b7b3dab2b013156eb3 |
| Species | *Rikenella microfusus* | 83.30% | 0529e4d63586aee5872eb8381a8eac59 |
| Species | *Rikenella microfusus* | 80.51% | e44754a6a734c6b649dfa1ca6d4f8ada |
| Species | *Rikenella microfusus* | 98.12% | 46c74b46461bbaa8c6145bfc5cdc8a29 |
| Species | *Rikenella microfusus* | 97.67% | ee89fb1784590ec656edce4c02845003 |
| Species | *Robinsoniella peoriensis* | 81.79% | 88bffcdbded3da50bcdd4f3a37ecab37 |
| Species | *Robinsoniella peoriensis* | 82.99% | b492ba67d0d3e2a8d1243e34057c93d5 |
| Species | *Robinsoniella peoriensis* | 86.28% | 136a7eae571a0bc117f3ab348daf5fdc |
| Species | *Robinsoniella peoriensis* | 80.67% | 7e9190c83934e43bd51559078a60ef71 |
| Species | *Robinsoniella peoriensis* | 71.35% | 9d2367768a08a8cb89ea2956ee387eb3 |
| Species | *Robinsoniella peoriensis* | 86.42% | 8fd1a39f026957488193a374cf95bb55 |
| Species | *Robinsoniella peoriensis* | 79.36% | 569c2ca35d053e919bbb504bfa72ad3e |
| Species | *Robinsoniella peoriensis* | 80.38% | 32ef823d50858fa9565bc957861984b6 |
| Species | *Robinsoniella peoriensis* | 79.93% | 1e117f169642f60083d22ed640b52d50 |
| Species | *Robinsoniella peoriensis* | 75.17% | e91051b6464edbc15b99e243fab89d1a |
| Species | *Robinsoniella peoriensis* | 85.09% | 11fd9ccf968a65011c41a56f2507a96c |
| Species | *Robinsoniella peoriensis* | 83.37% | 30b925e01a42ec03172c816169fbd39d |
| Species | *Roseburia hominis* | 79.30% | c8916f791913b2e2c3ec3e7118faaed8 |
| Species | *Ruminococcaceae bacterium* | 83.37% | 0e229021f15b6e4294c4fd7a930e7689 |
| Species | *Ruminococcaceae bacterium* | 99.99% | 10a10597425f6989da508663dfdf35c5 |
| Species | *Ruminococcaceae bacterium* | 89.24% | 7b90b2a118afae29dbbf9b8e954a5d96 |
| Species | *Ruminococcus bicirculans* | 74.78% | f70d61a956190744158a80ce73216cbc |
| Species | *Ruminococcus faecis* | 79.89% | 2db90cbd683a75b496297a3cda22925c |
| Species | *Sanguibacter gelidistatuariae* | 96.21% | 94ca0e18e3cb41ab08a0eae755ebc666 |
| Species | *Sanguibacter gelidistatuariae* | 95.63% | 07560189b9b7e89a72be1cfdacff845a |
| Species | *Sebaldella termitidis* | 100.00% | a23d91937b6960c49c40d6e9a06c4644 |
| Species | *Selenomonadales bacterium* | 99.95% | 04ee1b2fbd116b35eb08762569d38e43 |
| Species | *Selenomonadales bacterium* | 99.95% | c6cdb4dcff0c030da34b730596932afd |
| Species | *Selenomonadales bacterium* | 99.95% | 6dd776490dbf7795862650bcee00a711 |
| Species | *Selenomonadales bacterium* | 96.85% | 58de58c34bce4029f79b6733522aec84 |
| Species | *Selenomonadales bacterium* | 99.50% | 7774bda0412a902429302ea54d55c76a |
| Species | *Senegalimassilia anaerobia* | 97.09% | 83612f1715d55a247f81256f53eca7ad |
| Species | *Serratia symbiotica* | 91.49% | 235d57ac1fc7437ed418308e8984f64b |
| Species | *Sphingomonas aerophila* | 94.24% | 8adf7b042e2713f1319d27cca42481b0 |
| Species | *Staphylococcus carnosus* | 100.00% | 0ba001156c57d5dba7a68b431ddb5a56 |
| Species | *Staphylococcus carnosus* | 100.00% | 0748888e506686790039002b6f6ac9f9 |
| Species | *Streptococcus vestibularis* | 99.58% | bb58a05dc0516b99d4961f8bbdfeec1b |
| Species | *Streptomyces sp.* | 71.37% | cb0c99736b05badb14a69b17af3b1a7d |
| Species | *Streptomyces sp.* | 72.18% | 55e004c5634512d6c4760bbb3b7dfcbf |
| Species | *Streptomyces sp.* | 99.98% | 914032b162ff05f4a9949c16cee00dc0 |
| Species | *Streptomyces sp.* | 98.51% | c165ac55529bde3d19940b3b574de190 |
| Species | *Streptomyces sp.* | 94.08% | d53b6e96dbae1037f4847aacf6832413 |
| Species | *Streptomyces sp.* | 100.00% | 777d1457fd2b22fd76c62f837b66e983 |
| Species | *Streptomyces sp.* | 100.00% | eef22d407a7c91be44e4b12ca312b497 |
| Species | *Streptomyces sp.* | 99.99% | d88a3a54422b615dace023af818e78b5 |
| Species | *Streptomyces sp.* | 96.68% | ae451434099a9c60d2f9613ed399c97e |
| Species | *Streptomyces sp.* | 76.92% | 6271598096aed9bf1c26c31484bc3196 |
| Species | *Streptomyces sp.* | 99.70% | 8f4f8e8169d3999ceb821c09ec405bd2 |
| Species | *Streptomyces sp.* | 90.71% | 74dfa2bace0bfbfd2ebdc21e48072982 |
| Species | *Streptomyces sp.* | 98.51% | a5737b5ac669175a47ecbb9a00a1a7ae |
| Species | *Streptomyces sp.* | 100.00% | 3519b70afb36075fd5cd3a2f0bc926dc |
| Species | *Terrisporobacter mayombei* | 92.96% | 9da7b7b2fbe26fd07320a2ad7bdac53d |
| Species | *Tissierella carlieri* | 90.89% | feed718724792089945066d41f545d25 |
| Species | *Trebouxia sp.* | 98.28% | 2bf55179d10971c1a7d36d0c15a6a548 |
| Species | *Trebouxia sp.* | 91.31% | a436cb320d80c127b8b0ae6f3d7cfcab |
| Species | *Tyzzerella sp.* | 73.47% | de0d3a6332c613d42c1ff74e6f0bc958 |
| Species | *Tyzzerella sp.* | 75.19% | a86cd1b0f2808a20a9fbbec1eaa87a81 |
| Species | *Tyzzerella sp.* | 80.90% | 31277344a78c1fccfa8f37b90d760af4 |
| Species | *Tyzzerella sp.* | 74.31% | d822264edf9a42a84c4b30e71e9a0f5e |
| Species | *Vagococcus fluvialis* | 74.94% | 9e30822c297122ea922d039d2f180801 |
| Species | *Vagococcus fluvialis* | 74.66% | a9c2a69ea2f44a8964a763181b2742d1 |
| Species | *Vagococcus fluvialis* | 78.74% | 2933d640edd6f28e15b5fa32c57e2aae |
| Species | *Vagococcus fluvialis* | 74.06% | 1a0d50072d7714386911e5e8339008d5 |
| Species | *Vagococcus fluvialis* | 73.10% | 52ef861094a652a21e9d248f0b773915 |
| Species | *Vagococcus fluvialis* | 75.41% | e51ab3081a44b8515bf1239b3f41e6e7 |
| Species | *Vagococcus teuberi* | 98.57% | c05c6e95fd3ebd3fa4dc82820f842b3f |
| Species | *Vagococcus teuberi* | 98.57% | f354e872116988230db53261cfc7bb0d |
| Species | *Vagococcus teuberi* | 98.57% | 07dd49600004bd2980755c052ca13bd8 |
| Species | *Vagococcus teuberi* | 98.57% | e1ed52a33764d5c84a3e4ec96dbcd925 |
| Species | *Vagococcus teuberi* | 98.51% | ac46b4e3c226ac2e90baf8a28bf606ae |
| Species | *Vagococcus teuberi* | 98.58% | 3538faea7044eef45148f5ea262707a7 |
| Species | *Vagococcus teuberi* | 98.51% | f2d78399297681ac3b4d6554f3ca5f60 |
| Species | *Vagococcus teuberi* | 96.06% | 34f98d9e7d457bef709c142349d0a242 |
| Species | *Vagococcus teuberi* | 98.57% | 99d5da78d681dd40043e21a5beec83ba |
| Species | *Vagococcus teuberi* | 98.50% | de7e67e12a0e2505096a926e0b8c1abf |
| Species | *Vagococcus teuberi* | 98.51% | 125b21a752032b2211d1d024e4dcdc82 |
| Species | *Vagococcus teuberi* | 96.27% | a8ecf45eaa0a49d4babfff0da019f990 |
| Species | *Vagococcus teuberi* | 98.84% | e16f2c25fb349e0377a4c7b53699bcf0 |
| Species | *Victivallales bacterium* | 98.74% | 78bf44eaa75b57c3eca33cc56df2f5f4 |
| Species | *Victivallales bacterium* | 98.79% | 5dd3e670e857a3d4fea99dd11de1e592 |
| Species | *Victivallis vadensis* | 99.96% | 2dc0147f130a365b652e274b053df5dd |
| Species | *Victivallis vadensis* | 100.00% | c403751c87eabfa77d202984d39b2548 |
| Species | *Victivallis vadensis* | 100.00% | f836357305d22cb46cc34fe528430be2 |
| Species | *Victivallis vadensis* | 100.00% | 8ae361f8fce8e40092444e9389e151d9 |
| Species | *Victivallis vadensis* | 97.85% | 302db76010cbe6354e6a40007a88e182 |
| Species | *Victivallis vadensis* | 99.79% | c0fb6ec09277cd8dae54a5a79f91eab6 |
| Species | *Victivallis vadensis* | 100.00% | 9f7823ba31332821f9b65e60f808d3a4 |
| Species | *Victivallis vadensis* | 100.00% | d1cdf55071b1f47a15d4a9db7d2c5e50 |
| Species | *Victivallis vadensis* | 100.00% | 6c0de262dadfe5f496aa0ebc59e7684b |
| Species | *Victivallis vadensis* | 100.00% | 08183497f51119477631119c90d106c3 |
| Species | *Watanabea reniformis* | 97.82% | 83d69e81f5d2d4fd7317bb1178c77686 |
